# Abstracts from the Food Allergy and Anaphylaxis Meeting 2016

**DOI:** 10.1186/s13601-017-0142-2

**Published:** 2017-03-30

**Authors:** Guillaume Pouessel, Claire Claverie, Julien Labreuche, Jean-Marie Renaudin, Aimée Dorkenoo, Mireille Eb, Anne Moneret-Vautrin, Antoine Deschildre, Stephane Leteurtre, Linus Grabenhenrich, Margitta Worm, Sabine Dölle, Kathrin Scherer, Isidor Hutteger, Morten Christensen, Carsten Bindslev-Jensen, Charlotte Mortz, Esben Eller, Henrik Fomsgaard Kjaer, Leonor Carneiro-Leão, Jenny Badas, Alice Coimbra, Dikla Pivko Levy, Moshe Ben-Shoshan, Ayelet Rimon, Shira Benor, Nicolette J. T. Arends, Nikki Edelbroek, Hans de Groot, Joyce A. M. Emons, H. Kim A. Brand, Dirk Verhoeven, Leonieke N. van Veen, Nicolette W. de Jong, Geunwoong Noh, Eun Ha Jang, Mariona Pascal, Olga Dominguez, Mònica Piquer, Montserrat Alvaro, Rosa Jimenez-Feijoo, Jaime Lozano, Adriana Machinena, Maria del Mar Folqué, Maria Teresa Giner, Ana María Plaza, Paul Turner, Nandinee Patel, Marta Vazquez-Ortiz, Sarah Lindsley, Lucy Walker, Simon Rosenberg, Adriano Mari, Claudia Alessandri, Ivana Giangrieco, Lisa Tuppo, Chiara Rafaiani, Georg Mitterer, Michela Ciancamerla, Rosetta Ferrara, Maria Livia Bernardi, Danila Zennaro, Maurizio Tamburrini, Maria Antonetta Ciardiello, Christian Harwanegg, Antonio Fernandez, Regina Selb, Philippe Egenmann, Michelle Epstein, Karin Hoffmann-Sommergruber, Frits Koning, Martinus Lovik, E. N. Clare Mills, Javier Moreno, Henk van Loveren, Jean-Michel Wal, Susanne Diesner, Cornelia Bergmayr, Barbara Pfitzner, Vera Elisabeth Assmann, Philipp Starkl, David Endesfelder, Thomas Eiwegger, Zsolt Szepfalusi, Heinz Fehrenbach, Erika Jensen-Jarolim, Anton Hartmann, Isabella Pali-Schöll, Eva Untersmayr, Soren Wille, Peter Meyer, Caroline Klingebiel, Jonas Lidholm, Angelica Ehrenberg, Jonas Östling, Isabelle Cleach, Jean-Louis Mège, Joana Vitte, Roberta Aina, Pawel Dubiela, Sabine Pfeifer, Merima Bublin, Christian Radauer, Piotr Humeniuk, Stefan Kabasser, Riccardo Asero, Gador Bogas, Francisca Gomez, Paloma Campo, Maria Salas, Inmaculada Doña, Esther Barrionuevo, Maria Auxiliadora Guerrero, Cristobalina Mayorga, Ana Prieto, Domingo Barber, Maria Jose Torres, Annette Jamin, Andrea Wangorsch, Barbara Ballmer, Stefan Vieths, Stephan Scheurer, Danijela Apostolovic, Jelena Mihailovic, Maja Krstic, Maria Starkhammar, Tanja Cirkovic Velickovic, Carl Hamsten, Marianne van Hage, Francine C. van Erp, Edward F. Knol, Hannah M. Kansen, Bo Pontoppidan, Yolanda Meijer, Cornelis K. van der Ent, André C. Knulst, Rebekah Sayers, Helen Brown, Adnan Custovic, Angela Simpson, Claire Mills, Juliane Schulz, Network for Online Registration of Anaphylaxis (NORA), Jaap Akkerdaas, Muriel Totis, Annabelle Capt, Corinne Herouet-Guicheney, Ronald van Ree, Tushar Banerjee, Antima Banerjee, Mathilde Claude, Grégory Bouchaud, Roberta Lupi, Laure Castan, Olivier Tranquet, Sandra Denery-Papini, Marie Bodinier, Chantal Brossard, Rosella De Poi, Elisa Gritti, Emiliano De Dominicis, Bert Popping, Patrizia Polverino de Laureto, Kati Palosuo, Anna Kaarina Kukkonen, Anna Pelkonen, Mika Mäkelä, Nanju Alice Lee, Johanna Rost, Sridevi Muralidharan, Dianne Campbell, Sam Mehr, Catherine Nock, Joseph Baumert, Steve Taylor, Carla Mastrorilli, Salvatore Tripodi, Carlo Caffarelli, Serena Perna, Andrea Di Rienzo Businco, Ifigenia Sfika, Arianna Dondi, Annamaria Bianchi, Carlotta Povesi Dascola, Giampaolo Ricci, Francesca Cipriani, Nunzia Maiello, Michele Miraglia del Giudice, Tullio Frediani, Simone Frediani, Francesco Macrì, Chiara Pistoletti, Iride Dello Iacono, Maria Francesca Patria, Elena Varin, Diego Peroni, Pasquale Comberiati, Loredana Chini, Viviana Moschese, Sandra Lucarelli, Roberto Bernardini, Giuseppe Pingitore, Umberto Pelosi, Roberta Olcese, Matteo Moretti, Anastasia Cirisano, Diego Faggian, Alessandro Travaglini, Mario Plebani, Maria Carmen Verga, Mauro Calvani, Paolo Giordani, Paolo Maria Matricardi, Noe Ontiveros, Francisco Cabrera-Chavez, Julie Galand, Etienne Beaudouin, The Anaphylaxis Working Group of the French Allergology SocietyThe Anaphylaxis Working Group of the French Allergology Society, Florence Pineau, Shinobu Sakai, Kayoko Matsunaga, Reiko Teshima, Colette Larré, Sandra Denery, Sebastian Tschirner, Valérie Trendelenburg, Gabriele Schulz, Bodo Niggemann, Kirsten Beyer, Youcef Bouferkas, Younes Belabbas, Djamel Saidi, Omar Kheroua, Kamel Eddine El Mecherfi, Malika Guendouz, Abir Haddi, Hanane Kaddouri, Luis Amaral, Ana Pereira, Susana Rodrigues, Mareen Datema, Laurian Jongejan, Michael Clausen, Andre Knulst, Nikolaos Papadopoulos, Marek Kowalski, Frédéric de Blay, Aeilko Zwinderman, Karin Hoffman-Sommergruber, Barbara Ballmer-Weber, Montserrat Fernandez-Rivas, Shan Deng, Jia Yin, Charlotte Eisenmann, Maria Nassiri, Rabea Reinert, Johanna P. M. van der Valk, Roy Gerth van Wijk, Yvonne Vergouwe, Ewout W. Steyerberg, Marit Reitsma, Harry J. Wichers, Huub F. J. Savelkoul, Berber Vlieg-Boerstra, Anthony E. J. Dubois, Nicolette W. de Jong, Fabrícia Carolino, Ana Rodolfo, Josefina Cernadas, Dasha Roa-Medellín, Ana Rodriguez-Fernandez, Joaquín Navarro, Vicente Albendiz, María Luisa Baeza, Sonsoles Intente-Herrero, Andrea Mikkelsen, Kirsten Mehlig, Lauren Lissner, Linda Verrill, Stefano Luccioli, Jolanda van Bilsen, Frieke Kuper, André Wolterbeek, Tanja Rouhani Rankouhi, Lars Verschuren, Hilde Cnossen, Prescilla Jeurink, Johan Garssen, Léon Knippels, Jossie Garthoff, Geert Houben, Winfried Leeman, M. Eleonore Pettersson, Afke M. M. Schins, Gerard H. Koppelman, Boudewjin J. Kollen, Svitlana Zubchenko, Sarah Kuntz, Pablo Mérida, Montserrat Álvaro, Monica Piquer, Carmen Riggioni, Juan Heber Castellanos, Rosa Jimenez, Melanie Cap, Elodie Drumez, Stéphanie Lejeune, Caroline Thumerelle, Clémence Mordacq, Véronique Nève, Sonia Ricò, Margherita Varini, Rita Nocerino, Linda Cosenza, Antonio Amoroso, Margherita Di Costanzo, Carmen Di Scala, Giorgio Bedogni, Roberto Berni Canani, Paul J. Turner, Paloma Poza-Guedes, Ruperto González-Pérez, Inmaculada Sánchez-Machín, Victor Matheu-Delgado, Erik Wambre, Anne-Sofie Ballegaard, Charlotte Madsen, Juliane Gregersen, Katrine Lindholm Bøgh, Philippe Aubert, Michel Neunlist, Antoine Magnan, Daniel Lozano-Ojalvo, Alba Pablos-Tanarro, Leticia Pérez-Rodríguez, Elena Molina, Rosina López-Fandiño, Akila Rekima, Patricia Macchiaverni, Mathilde Turfkruyer, Sebastien Holvoet, Lénaïck Dupuis, Nour Baiz, Isabella Annesi-Maesano, Annick Mercenier, Sophie Nutten, Valérie Verhasselt, Ines Mrakovcic-Sutic, Srdan Banac, Ivana Sutic, Zdenka Baricev-Novakovic, Ingrid Sutic, Valentino Pavisic, Rosa Muñoz-Cano, Teodoríkez Jiménez-Rodríguez, Daniel Corbacho, Jordi Roca-Ferrer, Joan Bartra, Aleksandar Bulog, Vladimir Micovic, Lidia Markiewicz, Agata Szymkiewicz, Anna Szyc, Barbara Wróblewska, Bryan M. Harvey, Lucien F. Harthoorn, A. Wesley Burks, Georgios Rentzos, Anna-Lena Bramstång Björk, Ulf Bengtsson, Colin Barber, Chrystyna Kalicinsky, Christine Breynaert, Lieve Coorevits, Cornelia Jansen, Erna Van Hoeyveld, Kristin Verbeke, Anne-Marie Kochuyt, Rik Schrijvers, Diana Deleanu, Adriana Muntean, Maria Konstantakopoulou, Maria Pasioti, Anastasia Papadopoulou, Anna Iliopoulou, Nikolaos Mikos, Evangelia Kompoti, Eunice Dias de Castro, Borja Bartalomé, Kok Loong Ue, Elizabeth Griffiths, Stephen Till, Kate Grimshaw, Graham Roberts, Anna Selby, Indre Butiene, Jose Ignacio Larco, Ruta Dubakiene, Ana Fiandor, Alessandro Fiocchi, Nikos Papadopoulos, Sigurveig Sigurdardottir, Aline Sprikkelman, Anne-Fleur Schoemaker, Paraskevi Xepapadaki, Thomas Keil, Zizi Cojocariu, Beatriz Secades Barbado, Vasti Iancu, Esozia Arroabarren, Marta Goñi Esarte, Miren Arteaga, Mayra Coutinho Andrade, Denise Borges, Jorge Kalil, Pedro Giavina Bianchi, Rosana Camara Agondi, Rinkesh Kumar Gupta, Akanksha Sharma, Kriti Gupta, Mukul Das, Premendra Dwivedi, Rusudan Karseladze, Liana Jorjoliani, Lali Saginadze, Mariam Tskhakaia, Katia Basello, Gabriele Piuri, Attilio Francesco Speciani, Michela Carola Speciani, Carla Camerotto, Francesco Zinno, Olga Pakholchuk, Svitlana Nedelska, Stefano Pattini, Maria Teresa Costantino, Silvia Peveri, Danilo Villalta, Eleonora Savi, Andrea Costanzi, Vera A. Revyakina, Marina A. Kiseleva, Elena D. Kuvshinova, Inna A. Larkova, Anton A. Shekhetov, Diana Silva, André Moreira, José Plácido, Hanneke van der Kleij, Esther van Twuijver, Robbert Sutorius, Pieter-Jan de Kam, Jenny van Odijk, Helen Lindqvist, Elin Lustig, Amyra Ali Azamar Jácome, Karla Leversia Borjas Aguilar, Miguel García Domínguez, David Alejandro Mendoza Hernández, Cristiano Caruso, Cono Casale, Gian Lodovico Rapaccini, Antonino Romano, Italo De Vitis, Renata R. Cocco, Carolina Aranda, Marcia C. Mallozi, Jackeline F. Motta, Lilian Moraes, Antonio Pastorino, Nelson Rosario, Ekaterini Goudouris, Arnaldo Porto, Neusa F. Wandalsen, Emanuel Sarinho, Flavio Sano, Dirceu Solé, Constantinos Pitsios, Maria Petrodimopoulou, Ekaterini Papadopoulou, Maria Passioti, Meropi Kontogianni, Nino Adamia, Ekaterina Khaleva, Ana Prieto del Prado, George Du Toit, Edyta Krzych, Urszula Samolinska-Zawisza, Konrad Furmanczyk, Aneta Tomaszewska, Filip Raciborski, Agnieszka Lipiec, Piotr Samel-Kowalik, Artur Walkiewicz, Jacek Borowicz, Boleslaw Samolinski, Aimee Lou Nano, Marysia Recto, Maria Luisa Somoza, Natalia Blanca López, Diana Pérez Alzate, Francisco Javier Ruano, Maria Isabel Garcimartín, Elisa Haroun, Maria Vázquez de la Torre, Antonia Rojas, Montserrat López Onieva, Gabriela Canto, Alexandra Rodrigues, Andreia Forno, António Jorge Cabral, Rute Gonçalves, Ilya Vorozhko, Tatyana Sentsova, Olga Chernyak, Svetlana Denisova, Lidia Ilènko, Valery Muhortnich, Caroline Zimmermann, Alexander Rohrbach, Faisal R. Bakhsh, Kollen Boudewijn, Anne-Marie Oomkes-Pilon, Dorien Van Ginkle, Mira Šilar, Anja Jeverica, Tina Vesel, Tadej Avčin, Peter Korošec, Johanna van der Valk, Irene Berends, Nicolette Arends, Maurits van Maaren, Harry Wichers, Joyce Emons, Anthony Dubois, Nicolette de Jong, Oksana Matsyura, Lesya Besh, Chung-Hsiung Huang, Tong-Rong Jan, Gary Stiefel, Jean Tratt, Kerrie Kirk, Fabricia Carolino, Stefania Arasi, Lucia Caminiti, Giuseppe Crisafulli, Chiara Fiamingo, Jlenia Fresta, Giovanni Pajno, Ben Remington, Astrid Kruizinga, W. Marty Blom, Joost Westerhout, Sabina Bijlsma, Joe Baumert, Mark Blankestijn, Henny Otten, Rob Klemans, Anouska D. Michelsen-Huisman, Harmieke van Os-Medendorp, Astrid G. Kruizinga, Astrid Versluis, Gert van Duijn, H. Mary-Lene de Zeeuw-Brouwer, Jacqueline J. M. Castenmiller, Hub P. J. M. Noteborn, Geert F. Houben, Kristian Bravin, David Luyt, Bushra Javed, Phil Couch, Christopher Munro, Phil Padfield, Matt Sperrin, Aideen Byrne, Lizalet Oosthuizen, Carina Kelleher, Fiona Ward, Niamh Brosnan, Graham King, Eva Corbet, Josué Alejandro Huertas Guzmán, Montserrat Bosque García, Oscar Asensio, Laura Valdesoiro Navarrete, Helena Larramona, Xavier Domingo Miró, Katarzyna Pyrz, Moira Austin, Yanne Boloh, Philip Couch, Deirdre Galloway, Pilar Hernandez, Jonathan O’B. Hourihane, Fiona Kenna, Barbara Majkowska-Wojciechowska, Lynne Regent, Marina Themisb, Sabine Schnadt, Aida Semic-Jusufagic, Audrey Dunn Galvin, Tiina Kauppila, Mikael Kuitunen, Nikolaos A. Kitsioulis, Nikolaos Douladiris, Sofia Kostoudi, Ioanna Manolaraki, Dimitris Mitsias, Emmanouil Manousakis, Nikolaos G. Papadopoulos, Rebecca Knibb, Jennifer Hammond, Richard Cooke, Jaakko Yrjänä, Anna-Maija Hanni, Päivi Vähäsarja, Oona Mustonen, Teija Dunder, Petri Kulmala, Eva Lasa, Carmen D’Amelio, Sara Martínez, Alejandro Joral, Gabriel Gastaminza, Maria Jose Goikoetxea, David C. A. Candy, Marleen T. J. Van Ampting, Manon M. Oude Nijhuis, Assad M. Butt, Diego G. Peroni, Adam T. Fox, Jan Knol, Louise J. Michaelis, Ines Padua, Patricia Padrao, Pedro Moreira, Renata Barros, Hanan Sharif, Manzoor Ahmed, Nehad Gomaa, Joris Mens, Koen Smit, Frans Timmermans, Tomaž Poredoš, Anja Koren Jeverica, Marjeta Sedmak, Evgen Benedik, Meta Accetto, Mirjana Zupančič, Glauce Yonamine, Gustavo Soldateli, Bruna Aquilante, Antonio Carlos Pastorino, Cleonir Lui de Moraes Beck, Andrea Keiko Gushken, Mayra de Barros Dorna, Cristiane Nunes dos Santos, Ana Paula Moschione Castro, Abdulhadi Al-Qahtani, Rand Arnaout, Agha Rehan Khaliq, Rashid Amin, Farrukh Sheikh, Jorge Alvarez, Marta Anda, Miriam Palacios, Montserrat De Prada, Carmen Ponce, Bianca Balbino, Riccardo Sibilano, Thomas Marichal, Nicolas Gaudenzio, Hajime Karasuyama, Pierre Bruhns, Mindy Tsai, Laurent L. Reber, Stephen J. Galli, Ana Reis Ferreira, Josefina R. Cernadas, Aida del Campo García, Sara Pereiro Fernández, Nerea Sarmiento Carrera, Fernando Bandrés Sánchez-Cruz, José Ramón Fernández Lorenzo, Stephanie Claus, Claudia Pföhler, Franziska Ruëff, Regina Treudler, Mercedes Escarrer Jaume, Agustin Madroñero, Maria Teresa Guerra Perez, Juan Carlos Julia, Charlotte Hands Plovdiv, Lee Gethings, Jim Langridge, Karine Adel-Patient, Hervé Bernard, Ivona Barcievic-Jones, Raditsa Sokolova, Rumyana Yankova, Mariya Ivanovska, Marianna Murdjeva, Tatyana Popova, Svetlan Dermendzhiev, Martin Karjalainen, Ulrike Lehnigk, Duncan Brown, Julie C. Locklear, Julie Locklear, Ioana Maris, Jonathan Hourihane, Cristina Ornelas, Joana Caiado, Manuel Branco Ferreira, Manuel Pereira-Barbosa, Yolanda Puente, Juan Carlos Daza, Francisco Javier Monteseirin, Natalia Ukleja-Sokolowska, Ewa Gawronska-Ukleja, Magdalena Zbikowska-Gotz, Zbigniew Bartuzi, Lukasz Sokolowski, Aine Adams, Bernard Mahon, Karen English, Nelly Gourdon-Dubois, Laetitia Sellam, Bruno Pereira, Elodie Michaud, Khaled Messaoudi, Bertrand Evrard, Jean-Luc Fauquert, Francisca Palomares, Gador Gomez, Maria Jose Rodriguez, Luisa Galindo, Ana Molina, Lorella Paparo, Maurizio Mennini, Rosita Aitoro, Adam Wawrzeńczyk, Michał Przybyszewski, Anna Wawrzeńczyk, Hulya Ercan Sarıcoban, Meltem Ugras, Zerrin Yalvac, Bertine M. J. Flokstra-de Blok, J. L. van der Velde, Andrea Vereda, Clara Ippolito, Amaranta Traversa, Daniela Adriano, Daniela Manila Bianchi, Silvia Gallina, Lucia Decastelli, Melina Makatsori, Anne Miles, Sonja Posega Devetak, Iztok Devetak, Soraya Ainad Tabet, Jeanette Fisker Trandbohus, Pernille Winther, Hans-Jørgen Malling, Kirsten Skamstrup Hansen, Lene Heise Garvey, Chia-Chi Wang, Yin-Hua Cheng, Chun-Wei Tung, Mariola Dietrich, Ingo Marenholz, Birgit Kalb, Sarah Grosche, Katharina Blümchen, Rupert Schlags, Mareike Price, Sylke Rietz, Jorge Esparza-Gordillo, Susanne Lau, Young-Ae Lee, Ali Almontasheri, Mohammad Al Bahkali, Sahar Elshorbagi, Abdullah Alfhaid, Mashary Altamimi, Eman Madbouly, Hassan Al-Dhekri, Rand K. Arnaout, Maria Basagaña, Sira Miquel, Borja Bartolomé, Bettina Brix, Stefanie Rohwer, Sandra Brandhoff, Alena Berger, Waltraud Suer, Alf Weimann, Cristina Bueno, Laura Martín-Pedraza, Sara Abián, Pablo San Segundo-Acosta, Juan Carlos López-Rodríguez, Rodrigo Barderas, Eva Batanero, Javier Cuesta-Herranz, María Teresa Villalba, Magna Correia, Filipe Benito-Garcia, Cristina Arêde, Susana Piedade, Mário Morais-Almeida, James Hindley, Ross Yarham, Anna Kuklinska-Pijanka, David Gillick, Karine Patient, Martin D. Chapman, Katrine L. Bøgh, Ana Miranda, Eugénia Matos, Anna Sokolova, Huan Rao, Ivona Baricevic-Jones, Frances Smith, Wentong Xue, Helga Magnusdottir, Anna G. Vidarsdottir, Sigrun Lund, Anders Blom Jensen, Bjorn R. Ludviksson, Reyna Simon, Robert Elfont, Sean Bennett, Robert Voyksner, Maria de Lurdes Torre, Songül Yürek, Margaretha A. Faber, Annick Bastiaensen, Evelyne Mangodt, Athina van Gasse, Ine Decuyper, Vito Sabato, Margo M. Hagendorens, Chris H. Bridts, Luc S. De Clerck, Didier Ebo, Susanne Schwarz, Mandy Ziegert, Saskia Albroscheit, Christian Schwager, Skadi Kull, Jochen Behrends, Niels Röckendorf, Frauke Schocker, Andreas Frey, Arne Homann, Wolf-Meinhard Becker, Uta Jappe, Nesrine Zaabat, Sylvia Osscini, Chantal Agabriel, Benoît Sterling, Ania Carsin, Valérie Liabeuf, Monica Maćków, Alina Zbróg, Monica Bronkowska, Justine Courtois, Romy Gadisseur, Catherine Bertholet, Pierre Lukas, Etienne Cavalier, Philippe Delahaut, Birgit Quinting, Margareta Brandt Gertmo, Ewa Ternesten Hasseus, Vladyslava Barzylovych, Júlio Oliveira, Luis F. Ensina, Carolina S. Aranda, Leire Dopazo, Rebeca Lopez, Raquel Perez, Laura Santos-Diez, Agurtzane Bilbao, Juan Miguel Garcia, Ignacio García Núñez, María Ángeles Algaba Mármol, María José Barasona Villarejo, José Antonio Bácter Martos, Marina Suárez Vergara, José María Ignacio García, Agata Michalska, Grzegorz Sergiejko, Robert Zacniewski, Ileana-Maria Ghiordanescu, Cristina Deaconu, Mihaela Popescu, Roxana Silvia Bumbacea, Alkerta Ibranji, Elida Nikolla, Gjustina Loloci, Nanna Juel-Berg, Lau Fabricius Larsen, Lars Kjaergaard Poulsen, João Marcelino, Ricardo Prata, Ana Célia Costa, Fátima Duarte, Marta Neto, Jennifer Santos, Luís Câmara Pestana, Daniel Sampaio, Paola Minale, Paola Dignetti, Donatella Bignardi, Irena Nedelea, Florin-Dan Popescu, Mariana Vieru, Florin-Adrian Secureanu, Carmen Saviana Ganea, Miguel Vieira, José Pedro Moreira Silva, Timothy Watts, Sophia Watts, Marta Lomikovska, Marina Peredelskaya, Natalia Nenasheva, Ivana Filipovic, Zorica Zivkovic, Djordje Filipovic, Jennette Higgs, Amena Warner, Carla Jones

**Affiliations:** 1 Department of Pediatrics, Children’s Hospital, Roubaix, France; 2 Division of Pulmonology and Allergology, Department of Pediatrics, Faculty of Medicine and Children’s Hospital, Lille, France; 3 Allergy Vigilance Network, Vandoeuvre les Nancy, France; 4 Université Lille 2, CHU Lille, EA 2694 - Santé Publique: épidémiologie et qualité des soins, Lille, France; 5 Biostatistics Unit, Maison Régionale de la Recherche Clinique, CHRU Lille, Lille, France; 6 Department of Allergology, Emile Durkheim Hospital, Epinal, France; 7 Centre d’Epidémiologie sur les Causes Médicales de Décès INSERM, CHU de Bicêtre, Le Kremlin-Bicêtre, France; 8 Charité - Universitätsmedizin Berlin, Berlin, Germany; 9 University Hospital Basel, Basel, Switzerland; 10 Universitätsklinikum Salzburg, Salzburg, Austria; 11 Department of Dermatology and Allergy Centre, Odense Research Center for Anaphylaxis (ORCA), Odense University Hospital, Odense, Denmark; 12 Odense University Hospital, Odense, Denmark; 13 Serviço de ImunoalergologiaCentro Hospitalar de São João E.P.E., Porto, Portugal; 14 Tel Aviv Sourasky Medical Center, Tel Aviv, Israel; 15 Erasmus MC Sophia Children’s Hospital - Kinderhaven, Rotterdam, The Netherlands; 16 Pediatric Department, Reinier de Graaf Groep, Delft, The Netherlands; 17 Department of Allergy, Jeju Halla General Hospital, Jeju, Republic of Korea; 18 Department of Respiratory Medicine, Hanmaeum General Hospital, Jeju, Republic of Korea; 19 Immunology Department, CDB Hospital Clinic de Barcelona, Barcelona, Spain; 20 Department of Allergy and Clinical Immunology, Hospital Sant Joan de Déu, Esplugues de Llobregat, Spain; 21 Imperial College London, London, UK; 22 Illuminatis Ltd, London, UK; 23 Centri Associati di Allergologia Molecolare (CAAM), Rome, Italy; 24 Istituto di Bioscienze e Biorisorse, Consiglio Nazionale delle Ricerche, Naples, Italy; 25 MacroarrayDx, Vienna, Austria; 26 European Food Safety Authority, Parma, Italy; 27 University Hospitals of Geneva, Geneva, Switzerland; 28 Medical University of Vienna, Vienna, Austria; 29 Leiden University Medical Center (LUMC), Leiden, The Netherlands; 30 Norwegian University of Science and Technology (NTNU), Trondheim, Norway; 31 The University of Manchester (UNIMAN), Manchester, UK; 32 Consejo Superior de Investigaciones Científicas (CSIC), Madrid, Spain; 33 Maastricht University, Maastricht, The Netherlands; 34 Institut National de la Recherche Agronomique (INRA), Paris, France; 35 Department of Pathophysiology and Allergy Research, Center of PathophysiologyInfectiology and Immunology, Medical University of Vienna, Vienna, Austria; 36 Department of Pediatrics and Adolescent Medicine, Medical University of Vienna, Vienna, Austria; 37 Research Unit Microbe-Plant Interactions, Research Group Molecular Microbial Ecology, Department of Environmental Sciences, Helmholtz Zentrum München, German Research Center for Environmental Health, Neuherberg, Germany; 38 Scientific Computing Research Unit, Helmholtz Zentrum München, German Research Center for Environmental Health, Neuherberg, Germany; 39 Division of Immunology and Allergy, Food Allergy and Anaphylaxis Program, Department of Pediatrics, Hospital for Sick Children, Research Institute, Physiology and Experimental Medicine, University of Toronto, Toronto, ON Canada; 40 Priority Area Asthma & Allergy, Research Center Borstel, Airway Research Center North (ARCN), German Center for Lung Research (DZL), Borstel, Germany; 41 Messerli Research Institute of the University of Veterinary Medicine Vienna, Medical University Vienna and University of Vienna, Vienna, Austria; 42 Department of Pediatrics, Helsingborg Hospital, Helsingborg, Sweden; 43 Department of Pediatric Allergy, Skåne Universiy Hospital, Malmö, Sweden; 44 Laboratoire Montgrand, LBM Multisite SELDAIX - BIOPLUS, Marseille, France; 45 Thermo Fisher Scientific, Uppsala, Sweden; 46 Laboratoire d’Immunologie, Hôpital de la Conception, Assistance Publique Hôpitaux de Marseille, Marseille, France; 47 Aix-Marseille Université, Marseille, France; 48 Department of Pathophysiology and Allergy Research, Medical University of Vienna, Vienna, Austria; 49 Clinica San Carlo, Paderno Dugnano, Italy; 50 Allergy Unit, IBIMA-Regional University Hospital of Malaga, Malaga, Spain; 51 Research Laboratory, IBIMA-Regional University Hospital of Malaga, Malaga, Spain; 52 Institute for Applied Molecular Medicine (IMMA), School of Medicine, Universidad CEU San Pablo, Malaga, Spain; 53 Molecular Allergology, Paul-Ehrlich-Institut, Langen, Germany; 54 Allergy Unit, Department of Dermatology, University Hospital Zürich, Zurich, Switzerland; 55 Department of Medicine Solna, Immunology and Allergy Unit, Karolinska Institutet and Karolinska University Hospital, Stockholm, Sweden; 56 Center of Excellence for Molecular Food Sciences, Faculty of Chemistry, University of Belgrade, Belgrade, Serbia; 57 Department of Internal Medicine, Södersjukhuset, Stockholm, Sweden; 58 University Medical Centre Utrecht, Utrecht, The Netherlands; 59 University of Manchester, Manchester, UK; 60 Department of Dermatology and Allergology, Comprehensive Allergy Center Charité, Charité – Universitätsmedizin Berlin, Berlin, Germany; 61 Institute for Social Medicine, Epidemiology and Health Economics, Charité - Universitätsmedizin Berlin, Berlin, Germany; 62 The Allergy Vigilance Network, University Hospital Nancy, Nancy, France; 63 Academic Medical Center, Amsterdam, The Netherlands; 64 Bayer Crop Science, Valbonne, France; 65 Darlington Memorial Hospital, Darlington, UK; 66 INRA UR 1268 Biopolymers Interactions Assemblies, Nantes, France; 67 R&D Department Italy Mérieux NutriSciences, Resana, Italy; 68 Department of Pharmaceutical Sciences, Università di Padova, Padova, Italy; 69 Helsinki University Hospital, Skin and Allergy Hospital, University of Helsinki, Helsinki, Finland; 70 ARC Training Centre for Advanced Technology in Food Manufacture (ATFM), University of New South Wales, Sydney, Australia; 71 Children’s Hospital Westmead, Sydney, Australia; 72 Southern Cross University, East Lismore, Australia; 73 FARRP, University of Nebraska, Lincoln, NE USA; 74 Pediatric Department, Department of Clinical and Experimental Medicine, Azienda Ospedaliera-UniversitariaUniversity of Parma, Parma, Italy; 75 Department of Pediatric Pneumology and Immunology, Charité Medical University Berlin, Berlin, Germany; 76 The Italian Pediatric Allergy Network (I-PAN), Rome, Italy; 77 Pediatric Department and Pediatric Allergology Unit, Sandro Pertini Hospital, Rome, Italy; 78 Allergology Service, San Carlo Clinic, Paderno Dugnano, Milan, Italy; 79 Pediatric Unit, Department for Mother and Child, Ramazzini Hospital, Carpi, Italy; 80 Pediatric Unit, Mazzoni Hospital, Ascoli Piceno, Italy; 81 Pediatric Unit, Department of Medical and Surgical Sciences, University of Bologna, Bologna, Italy; 82 Pediatric Department, Second University, Naples, Italy; 83 Pediatric Department, La Sapienza University, Rome, Italy; 84 Pediatric Unit, Fatebenefratelli Hospital, Benevento, Italy; 85 Pediatric Highly Intensive Care Unit, Department of Pathophysiology and Transplantation, Università degli Studi di Milano, Fondazione IRCCS Ca’ Granda Ospedale Maggiore Policlinico, Milan, Italy; 86 Pediatric Intermediate Care Unit, Fondazione IRCCS Ca’ Granda Ospedale Maggiore Policlinico, Milan, Italy; 87 Pediatric Section, Department of Life and Reproduction Sciences, University of Verona, Verona, Italy; 88 Pediatric Department, Policlinico of Tor Vergata, Tor Vergata University, Rome, Italy; 89 Pediatric Unit, San Giuseppe Hospital, Empoli, Italy; 90 Pediatric Unit, Grassi Hospital, Rome, Italy; 91 Pediatric Unit, Santa Barbara Hospital, Iglesias, Italy; 92 Pulmonary Disease and Allergy Unit, G. Gaslini Hospital, Genoa, Italy; 93 Pediatric Unit, Crotone, Italy; 94 Department of Laboratory Medicine, University of Padua, Padua, Italy; 95 ASL Salerno, Salerno, Italy; 96 UOC Pediatria, San Camillo Forlanini, Rome, Italy; 97 Department of Statistical Sciences, Sapienza University of Rome, Rome, Italy; 98 University of Sinaloa, Culiacan, Mexico; 99 UR 1268 Biopolymères, Interactions, Assemblages, INRA, Nantes, France; 100 National Institute of Health Sciences, Tokyo, Japan; 101 Laboratory of Physiology of Nutrition and Food Safety, Department of BiologyFaculty of Natural and Life Sciences, University of Oran 1 Ahmed Ben Bella, Oran, Algeria; 102 LPNSA, Biology, Faculty of Natural and Life Sciences, University of Oran 1 Ahmed Ben Bella, Oran, Algeria; 103 CINTESIS, Faculdade de Medicina da Universidade do Porto, Porto, Portugal; 104 Serviço de Gastrenterologia, Centro Hospitalar de São João E.P.E, Porto, Portugal; 105 Academic Medical Centre, University of Amsterdam, Amsterdam, The Netherlands; 106 Landspitali University Hospital, Reykjavik, Iceland; 107 Centre for Paediatrics and Child Health, Institute of Human Development, University of Manchester, Manchester, UK; 108 Medical Univeristy of Łódź (MUL), Lodz, Poland; 109 University Hospital of Strasbourg, Strasbourg, France; 110 Phadia AB, Upsala, Sweden; 111 Paul-Ehrlich-Insitut, Federal Institute for Vaccines and Biomedicines, Langen, Germany; 112 Manchester Institute of Biotechnology, University of Manchester, Manchester, UK; 113 University Hospital Zürich, Zürich, Switzerland; 114 Hospital Clinico San Carlos, IdISSC, Madrid, Spain; 115 Peking Union Medical College Hospital, Beijing, China; 116 Department of Dermatology and Allergy, Allergie-Centrum-Charité, Charité-Universiätsmedizin Berlin, Berlin, Germany; 117 Department of Internal Medicine, Allergology, Erasmus Medical Center, Rotterdam, The Netherlands; 118 Center for Medical Decision Making, Department of Public Health, Erasmus MC, Rotterdam, The Netherlands; 119 Wageningen UR Food and Biobased Research, Wageningen, The Netherlands; 120 Laboratory of Cell Biology and Immunology, Wageningen University, Wageningen, The Netherlands; 121 Department of Paediatrics, Onze Lieve Vrouwe Gasthuis (OLVG), Amsterdam, The Netherlands; 122 Department of Pediatric Allergology, Diaconessenhuis Voorburg, RdGG, Delft, The Netherlands; 123 Department of Pediatric Pulmonology and Pediatric Allergology, University Medical Centre Groningen, GRIAC Research Institute, University of Groningen, Groningen, The Netherlands; 124 Serviço de Imunoalergologia, Centro Hospitalar São João, E.P.E., Porto, Portugal; 125 Hospital General Universitario Gregorio Marañón, Madrid, Spain; 126 Primary Care, Research and Development Centre, Gothenburg, Sweden; 127 Department of Public Health and Community Medicine, Section of Epidemiology and Social Medicine, Sahlgrenska Academy, University of Gothenburg, Gothenburg, Sweden; 128 Center for Food Safety and Applied Nutrition, FDA, College Park, MD USA; 129 TNO, Zeist, The Netherlands; 130 Triskelion, Zeist, The Netherlands; 131 Nutricia Research, Utrecht, The Netherlands; 132 Utrecht Institute for Pharmaceutical Sciences, Utrecht, The Netherlands; 133 Department of Pediatric Pulmonology and Pediatric Allergology, Beatrix Children’s HospitalUniversity of Groningen, University Medical Center Groningen, Groningen, The Netherlands; 134 GRIAC Research Institute, University of Groningen, University Medical Center Groningen, Groningen, The Netherlands; 135 Department of General Practice, University of Groningen, University Medical Center Groningen, Groningen, The Netherlands; 136 Danylo Halytsky Lviv National Medical University, Lviv, Ukraine; 137 Department of Pediatric Pneumology and Immunology, Charité Universitätsmedizin Berlin, Berlin, Germany; 138 Department of Pediatric Allergy and Clinical Immunology, Sant Joan de Déu Hospital, University of Barcelona, Barcelona, Spain; 139 Pediatric Pulmonology and Allergy Department, Hôpital Jeanne de Flandre, CHU Lille, Lille, France; 140 Biostatistics Department, CHU Lille, Lille, France; 141 Pediatric Pulmonary Function Testing Unit, Hôpital Jeanne de Flandre, CHU Lille, Lille, France; 142 Pediatric Clinic, Department of Clinical and Experimental Medicine, University of Parma, Parma, Italy; 143 University of Naples “Federico II”, Naples, Italy; 144 Section of Paediatrics, Imperial College, London, UK; 145 Hospital del Tórax, Santa Cruz de Tenerife, Spain; 146 Benaroya Research Instiute, Seattle, WA USA; 147 National Food Institute, Technical University of Denmark, Søborg, Denmark; 148 INSERMUMR1087, Institut du Thorax, Nantes, France; 149 CNRSUMR6291, Nantes, France; 150 Université de Nantes, Nantes, France; 151 INSERM UMR913, Institut des Maladies de l’Appareil Digestif (IMAD), Faculté de Médecine, Nantes, France; 152 DHU2020 Médecine Personnalisée des Maladies Chroniques, Nantes, France; 153 Service de Pneumologie, Institut du Thorax, CHU de Nantes, Nantes, France; 154 Instituto de Investigación en Ciencias de la Alimentación (CIAL CSIC-UAM), Madrid, Spain; 155 University of Nice Sophia Antipolis, TIM, EA 6302, Nice, France; 156 Institute of Biomedical Sciences - University of São Paulo, São Paulo, Brazil; 157 Nestle Research Center, Lausanne, Switzerland; 158 Sorbonne Universités, UPMC Univ Paris 06, INSERMInstitut Pierre Louis d’Epidémiologie et de Santé Publique (IPLESP UMRS 1136), Epidemiology of Allergic and Respiratory Diseases Department (EPAR), Medical School Saint-Antoine, Paris, France; 159 Department of Physiology and Immunology, Medical Faculty, Rijeka, Croatia; 160 Department of Pediatrics, Medical Faculty, Rijeka, Croatia; 161 Department of Public Health, Medical Faculty, University of Rijeka, Rijeka, Croatia; 162 Medical Faculty, University of Rijeka, Rijeka, Croatia; 163 Hospital Clinic, IDIBAPS-Universitat de Barcelona, Barcelona, Spain; 164 Hospital General Universitario de Alicante, Alicante, Spain; 165 Department of Family Medicine, Medical Faculty, University of Rijeka, Rijeka, Croatia; 166 Institute of Animal Reproduction and Food Research of Polish Academy of Sciences, Olsztyn, Poland; 167 Children’s Investigational Research Program, LLC (CHIRP), Bentonville, AR USA; 168 Nutricia Research, Nutricia Advanced Medical Nutrition, Utrecht, The Netherlands; 169 University of North Carolina, Chapel Hill, NC USA; 170 Section of Allergology, NÄL Hospital, Trollhättan, Sweden; 171 Intitute for Medicine, Sahlgrenska Academy, University of Gothenburg, Gothenburg, Sweden; 172 Department of Internal Medicine, Section of Clinical Immunology and Allergy, University of Manitoba, Winnipeg, MB Canada; 173 University Hospitals Leuven, Leuven, Belgium; 174 University of Medicine & Pharmacy Iuliu Hatieganu, Cluj-Napoca, Romania; 175 Allergology Department, Laiko” General Hospital, Athens, Greece; 176 Serviço de Imunoalergologia, Centro Hospitalar de São João, E.P.E., Porto, Portugal; 177 R&D Department, Bial Aristegui, Bilbao, Spain; 178 Department of Allergy, Guy’s and St Thomas’ NHS Foundation Trust, London, UK; 179 Department of Allergy, King’s College London, London, UK; 180 Experimental Sciences & Human Development in Health Academic Units, Faculty of Medicine, University of Southampton, Southampton, UK; 181 National Institute for Health Research Respiratory Biomedical Research Unit, Southampton, UK; 182 David Hide Asthma and Allergy Research Centre, St Mary’s Hospital, Newport, Isle of Wight UK; 183 Faculty of Health Sciences, Klaipeda University, Klaipeda, Lithuania; 184 La Paz Institute for Health Research, Madrid, Spain; 185 Children’s Hospital, Landspitali – The National University Hospital of Iceland, Reykjavik, Iceland; 186 Faculty of Medicine, Vilnius University, Vilnius, Lithuania; 187 Division of Allergy, Pediatric Hospital Bambino Jesu, Rome, Italy; 188 Institute of Social Medicine, Epidemiology and Health Economics, Charité Universitätsmedizin Berlin, Berlin, Germany; 189 Allergy Unit, 2nd Pediatric Clinic, University of Athens, Athens, Greece; 190 Department of Immunology, Landspitali – The National University Hospital of Iceland, Reykjavik, Iceland; 191 Department of Pediatric Respiratory Medicine and Allergy, Emma Children’s Hospital, Academic Medical Center Amsterdam, Amsterdam, The Netherlands; 192 Department of Immunology, Rheumatology and Allergy, Medical University of Lodz, Lodz, Poland; 193 Institute of Inflammation and Repair, Manchester Academic Health Science Centre, Manchester Institute of Biotechnology, University of Manchester, Manchester, UK; 194 Department of Paediatric Pneumonology and Immunology, Charité Universitätsmedizin Berlin, Berlin, Germany; 195 Complejo Hospitalario De Navarra, Pamplona, Navarra Spain; 196 Serviço de Imunologia Clínica e Alergia, Hospital das Clínicas, Universidade São Paulo, São Paolo, Brazil; 197 CSIR-Indian Institute of Toxicology Research, Lucknow, India; 198 Iv. Javakhishvili Tbilisi State University, Tbilisi, Georgia; 199 Iashvili Children’s Clinic, Tbilisi, Georgia; 200 Institute of Paediatrics, Tbilisi, Georgia; 201 GEK srl c/o Cryolab, Rome, Italy; 202 Inflammation Society, Orpington, UK; 203 GEK srl, Milan, Italy; 204 SMA srl, Milan, Italy; 205 Department of Biomedicine and Prevention, Faculty of Medicine, “Tor Vergata” University, Rome, Italy; 206 Zaporizhia State Medical University, Zaporizhia, Ukraine; 207 Dermatology Department, Allergy Unit, Azienda Ospedaliero-Universitaria Policlinico di Modena, Modena, Italy; 208 Centro Day Hospital Allergologia e Immunologia Clinica, ASST Carlo Poma Mantova, Mantova, Italy; 209 Allergy Unit, G. Da Saliceto Hospital, AUSL Piacenza, Piacenza, Italy; 210 Department of Laboratory Medicine, A.O.S. Maria degli Angeli, Pordenone, Italy; 211 Federal State Budgetary Scientific Institution, «Federal Research Centre of Nutrition Biotechnology and Food Safety», Moscow, Russia; 212 HAL Allergy B.V., Leiden, The Netherlands; 213 Department of Allergology, Sahlgrenska University Hospital, Gothenburg, Sweden; 214 Department of Clinical Nutrition, Sahlgrenska University Hospital, Gothenburg, Sweden; 215 Instituto Nacional de Pediatría, Mexico City, Mexico; 216 Allergy UnitFondazione Policlinico Gemelli, Rome, Italy; 217 Gastroenterology Unit, Fondazione Policlinico Gemelli, Rome, Italy; 218 Federal University of São Paulo, São Paulo, Brazil; 219 Federal University of Sergipe, Aracaju, Brazil; 220 Federal University of Mato Grosso, Cuiabá, Brazil; 221 Federal University of Paraná, Curitiba, Brazil; 222 Federal University of Rio de Janeiro, Rio de Janeiro, Brazil; 223 University of Rio Grande do Sul, Passo Fundo, Brazil; 224 Medical School of ABC, Santo André, Brazil; 225 Federal University of Pernambuco, Recife, Brazil; 226 Nipo-Brasileiro Hospital, São Paulo, Brazil; 227 Laikon General Hospital, Athens, Greece; 228 Harokopio University, Athens, Greece; 229 Guy’s and St Thomas’ NHS Foundation Trust, London, UK; 230 King’s College London, London, UK; 231 Unit of Environmental Hazard Prevention and Allergology, Warsaw Medical University, Warsaw, Poland; 232 University of the Philippines, Philippine General Hospital, Manila, Philippines; 233 Allergy Unit, University Hospital “Infanta Leonor”, Madrid, Spain; 234 Department of Pediatrics, Hospital Central do Funchal, Funchal Madeira, Portugal; 235 Federal Research Centre of Nutrition and Biotechnology, Moscow, Russia; 236 Pirogov Russian National Research Medical University, Moscow, Russia; 237 University Clinic of Respiratory and Allergic Diseases, Golnik, Slovenia; 238 Department of Allergology, Rheumatology and Clinical Immunology, University Children’s Hospital, University Medical Center Ljubljana, Ljubljana, Slovenia; 239 Department of Paediatric Allergology, Erasmus Medical Center Sophia Children’s Hospital, Rotterdam, The Netherlands; 240 National Institute of Infectious Diseases and Vaccinology, National Health Research Institutes, Miaoli, Taiwan; 241 Department and Graduate Institute of Veterinary Medicine, School of Veterinary Medicine, National Taiwan University, Taipei, Taiwan; 242 Leicester Royal Infirmary, Leicester, UK; 243 Leicester Partnership Trust, Leicester, UK; 244 Allergy Unit, Department of Pediatrics, University of Messina, Messina, Italy; 245 The Netherlands Organization for Applied Scientific Research (TNO), Zeist, The Netherlands; 246 University of Nebraska, Lincoln, NE USA; 247 Food Allergy Research & Resource Program (FARRP), Lincoln, NE USA; 248 Department of Dermatology/Allergology, University Medical Center Utrecht, Utrecht, The Netherlands; 249 NVWA, Netherlands Food and Consumer Product Safety Authority, Utrecht, The Netherlands; 250 University Hospitals of Leicester NHS Trust, Leicester, UK; 251 Institute of Inflammation and Repair, University of Manchester, Manchester, UK; 252 Institute of Population Health, University of Manchester, Manchester, UK; 253 Our Lady’s Children’s Hospital Crumlin, Dublin, Ireland; 254 School of Biological Sciences, Dublin Institute of Technology, Dublin, Ireland; 255 Allergy Department, Our Lady’s Children’s Hospital Crumlin, Dublin, Ireland; 256 Pediatric Pulmonology and Allergy Unit, Hospital de Sabadell, Corporació Parc Taulí, Barcelona, Spain; 257 School of Applied Psychology, University College Cork, Cork, Ireland; 258 Anaphylaxis Campaign (ACUK), Farnborough, UK; 259 Association Française pour la Prévention des Allergies (AFPRAL), Paris, France; 260 University of Manchester, University Hospital of South Manchester, Manchester, UK; 261 Anaphylaxis Ireland (AI), Cork, Ireland; 262 Asociación Española de Personas con Alergia a Alimentos y Látex (AEPNAA), Madrid, Spain; 263 Department of Pediatrics and Child’s Health, University College Cork, Cork, Ireland; 264 Deutscher Allergie- und Asthmabund e.V. (DAAB), Mönchengladbach, Germany; 265 University of Helsinki, Helsinki University Hospital, Skin and Allergy Hospital, Helsinki, Finland; 266 Allergy Department, 2nd Pediatric Clinic, University of Athens School of Medicine, Athens, Greece; 267 Aston University, Birmingham, UK; 268 PEDEGO Research Unit and Medical Research Center Oulu, University of Oulu and Oulu University Hospital, Oulu, Finland; 269 Pediatric Unit, Kontinkangas Healthcare and Wellness Center, Oulu, Finland; 270 Hospital Universitario Donostia, San Sebastián, Spain; 271 Clinica Universidad de Navarra, Pamplona, Spain; 272 Royal Alexandra Children’s Hospital, Brighton, UK; 273 Utrecht Institute for Pharmaceutical Sciences, Faculty of Science, Utrecht University, Utrecht, The Netherlands; 274 University Hospital Verona, Verona, Italy; 275 Guy’s & St Thomas’ Hospitals NHS Foundation Trust, London, UK; 276 Laboratory of Microbiology, Wageningen University, Wageningen, The Netherlands; 277 Great North Children’s Hospital, Royal Victoria Infirmary, Newcastle upon Tyne, UK; 278 Faculty of Nutrition and Food Sciences, University of Porto, Porto, Portugal; 279 Faculty of Medicine, University of Porto, Porto, Portugal; 280 Institute of Public Health, University of Porto, Porto, Portugal; 281 Research Centre in Physical Activity, Health and Leisure, University of Porto, Porto, Portugal; 282 Pediatric Department, University Hospital Sharjah, Sharjah, United Arab Emirates; 283 Department of Dermatology/Allergology, UMC Utrecht, Utrecht, The Netherlands; 284 Utrecht University Graduate School of Natural Sciences, Utrecht, The Netherlands; 285 Dutch Anaphylaxis Network, Dordrecht, The Netherlands; 286 University Children’s Hospital, University Medical Center, Ljubljana, Slovenia; 287 University Clinic of Respiratory and Allergic Diseases, Ljubljana, Slovenia; 288 Child Institute Clinical Hospital, University of São Paulo, São Paulo, Brazil; 289 King Faisal Specialist Hospital and Research Centre, Riyadh, Saudi Arabia; 290 Institut Pasteur, Paris, France; 291 Stanford University School of Medicine, Palo Alto, CA USA; 292 University of Liege, Liege, Belgium; 293 Tokyo Medical and Dental University, Tokyo, Japan; 294 Hospital Álvaro Cunqueiro, Vigo, Spain; 295 Department of Dermatology, Venereology and Allergology, Comprehensive Allergy Center, University Hospital, Leipzig, Germany; 296 Department of Dermatology and Allergology, Saarland University Hospital, Homburg, Germany; 297 Department of Dermatology and Allergology, Ludwig-Maximilians-University, Munich, Germany; 298 Allergy Unit, Department of Dermatology, University Hospital, Basel, Switzerland; 299 Juaneda Hospital, Palma de Mallorca, Spain; 300 Jerez, Spain; 301 Valencia, Spain; 302 Waters Corporation, Wilmslow, UK; 303 INRA-CRJJ, Jouey-en-Josas, France; 304 Department of Dermatology and Venereology, Medical University Plovdiv, Plovdiv, Bulgaria; 305 Department of Microbiology and Immunology, Medical University Plovdiv, Plovdiv, Bulgaria; 306 Department of Allergology and Occupational Medicine, Medical University Plovdiv, Plovdiv, Bulgaria; 307 Allergopharma GmbH & Co. KG, Reinbek, Germany; 308 Xcenda, LLC., Palm Harbor, FL USA; 309 EMD Serono, Inc., Rockland, MA USA; 310 Cork University Hospital, University College Cork, Cork, Ireland; 311 Imunoallergology Department, Centro Hospitalar Lisboa Norte, Hospital Santa Maria, Lisbon, Portugal; 312 Hospital Universitario Virgen Macarena, Seville, Spain; 313 University of Seville, Seville, Spain; 314 Immunoallergology Department, Centro Hospitalar de São João, E.P.E., Porto, Portugal; 315 Department of Allergology, Clinical Immunology and Internal Diseases, L. Rydygier Collegium Medicum, Bydgoszcz NCU, Bydgoszcz, Poland; 316 Division of Ergonomics and Exercise Physiology, L. Rydygier Collegium Medicum in Bydgoszcz NCU, Bydgoszcz, Poland; 317 Food for Health Ireland (FHI), Cellular Immunology Lab, Department of Biology, Institute of Immunology, Maynooth University, Maynooth, Ireland; 318 CHU Clermont-Ferrand, INSERM CIC 1405, Clermont-Ferrand, France; 319 Pediatric Allergy Unit, CHU Clermont-Ferrand, CHU Estaing, Clermont-Ferrand, France; 320 Unité de Biostatistiques, Direction de la Recherche Clinique (DRCI), CHU Clermont-Ferrand, Clermont-Ferrand, France; 321 Department of Immunobiology, UdA-INRA - CHU Montpied, CHU Clermont-Ferrand, Clermont-Ferrand, France; 322 IBIMA-HRUM, Malaga, Spain; 323 Department of Pediatric Allergy and Immunology, Yeditepe University Faculty of Medicine, Istanbul, Turkey; 324 Department of Pediatric Gastroenterology, Yeditepe University Faculty of Medicine, Istanbul, Turkey; 325 Department of Pediatrics, Yeditepe University Faculty of Medicine, Istanbul, Turkey; 326 UMCG, Groningen, The Netherlands; 327 Fundazion Dimenez Diaz, Madrid, Spain; 328 Istituto Zooprofilattico Sperimentale Piemonte, Liguria e Valle d’Aosta – SC Controllo Alimenti, Turin, Italy; 329 Department of Psychological Sciences, Birkbeck, University of London, London, UK; 330 Department of Pediatrics, General and Teaching Hospital Izola, Izola, Slovenia; 331 Faculty of Education, University of Ljubljana, Ljubljana, Slovenia; 332 Department of Pediatrics, Herlev and Gentofte Hospital, Copenhagen University Hospital, Copenhagen, Denmark; 333 Allergy Clinic, Herlev and Gentofte Hospital, Copenhagen University Hospital, Copenhagen, Denmark; 334 Kaohsiung Medical University, Kaohsiung, Taiwan; 335 National Taiwan University, Taipei, Taiwan; 336 Max-Delbrück-Center (MDC) for Molecular Medicine, Berlin, Germany; 337 Clinic for Pediatric Allergy, Experimental and Clinical Research Center, Charité - Universitätsmedizin Berlin, Berlin, Germany; 338 Department of Pediatric Pneumology and Immunology, Charité - Universitätsmedizin Berlin, Berlin, Germany; 339 Department of Allergy, Pulmonology and Cystic Fibrosis, Children’s Hospital, Goethe University, Frankfurt am Main, Germany; 340 Children’s Hospital, Wangen, Germany; 341 Clinic for Pediatric Pneumology, Allergology and Neonatology, University Children’s Hospital Hannover, Hannover, Germany; 342 Center for Pediatric and Adolescent Medicine, Elisabeth Children’s Hospital, Oldenburg, Germany; 343 Institute for Clinical Epidemiology and Biometry, University of Würzburg, Würzburg, Germany; 344 Department of Adult Allergy and Clinical Immunology, King Faisal Specialist Hospital and Research Centre, Riyadh, Saudi Arabia; 345 King Faisal Research Center, King Faisal Specialist Hospital and Research Centre, Riyadh, Saudi Arabia; 346 Department of Pediatric Allergy and Clinical Immunology, King Faisal Specialist Hospital and Research Centre, Riyadh, Saudi Arabia; 347 Allergy Unit, Hospital Universitari Germans Trias i Pujol, Universitat Autònoma de Barcelona, Badalona, Spain; 348 Bial-Aristegui Laboratories, Bilbao, Spain; 349 EUROIMMUN AG, Lübeck, Germany; 350 Department of Dermatology and Allergology, Comprehensive Allergy Center Charité, Charité Universitätsmedizin Berlin, Berlin, Germany; 351 Departamento de Bioquímica y Biología Molecular, Facultad de Ciencias Químicas, Universidad Complutense de Madrid, Madrid, Spain; 352 Departamento de Alergología, Fundación Jiménez Díaz, Madrid, Spain; 353 Allergy Center, CUF Descobertas Hospital, Lisbon, Portugal; 354 Indoor Biotechnologies Ltd, Cardiff, UK; 355 Hospital Professor Doutor Fernando Fonseca EPE, Amadora, Portugal; 356 Institute of Inflammation and Repair, Manchester Institute of Biotechnology, University of Manchester, Manchester, UK; 357 College of Food Science and Nutritional Engineering, China Agricultural University, Beijing, China; 358 Allergy Centre, University Hospital of South Manchester, Manchester, UK; 359 Department of Immunology, Landspitali University Hospital, Reykjavik, Iceland; 360 University of Iceland, Reykjavik, Iceland; 361 Children’s Hospital, Landspitali University Hospital, Reykjavik, Iceland; 362 Thermo Fisher Scientific, Copenhagen, Denmark; 363 Aimmune Therapeutics, Brisbane, CA USA; 364 LCMS Ltd., Durham, NC USA; 365 BIAL Industrial y Farmacéutica S.A., Zamudio, Spain; 366 Department of Immunology-Allergology-Rheumatology, Faculty of Medicine and Health Sciences, University of Antwerp, Antwerp University Hospital, Antwerp, Belgium; 367 Department of Pediatrics, Antwerp University Hospital, Antwerp, Belgium; 368 Division of Clinical and Molecular Allergology, Research Center Borstel, Priority Area Asthma and Allergy, Airway Research Center North (ARCN), German Center for Lung Research (DZL), Borstel, Germany; 369 Core Facility Fluorescence Cytometry, Research Center Borstel, Borstel, Germany; 370 Division of Mucosal Immunology and Diagnostics, Research Center Borstel, Priority Area Asthma and Allergy, Airway Research Center North (ARCN), German Center for Lung Research (DZL), Borstel, Germany; 371 Interdisciplinary Allergy Outpatient Clinic, Department of Internal Medicine, University of Luebeck, Luebeck, Germany; 372 Unité Allergologie et Exploration du Complément, Laboratoire d’Immuno-chimie et de Neuro-immunologie, Département d’Immunologie, Institut Pasteur d’Algérie, Dély-Brahim, Algeria; 373 Laboratoire d’Immunologie, Département de Pharmacie, Faculté de Médecine d’Alger, Ben Aknoun, Algeria; 374 Service de Pédiatrie Multidisciplinaire, Hôpital de la Timone, Assistance Publique Hôpitaux de Marseille, Marseille, France; 375 Service de Pédiatrie, Hôpital Nord, Assistance Publique Hôpitaux de Marseille, Marseille, France; 376 Service de Pneumologie Pédiatrique, Hôpital de la Timone, Assistance Publique Hôpitaux de Marseille, Marseille, France; 377 Service de Dermatologie et Vénéréologie, Hôpital de la Timone, Assistance Publique Hôpitaux de Marseille, Marseille, France; 378 Faculty of Health Science and Physical Education, Witelon State University of Applied Sciences, Legnica, Poland; 379 Faculty of Human Nutrition, University of Environmental and Life Sciences, Wroclaw, Poland; 380 CRIG Liège, Liège, Belgium; 381 CHU Liège, Liège, Belgium; 382 CER Group Marloie, Marloie, Belgium; 383 HELMo Liège, Liège, Belgium; 384 Immunoallergy Department, CUF Descobertas Hospital, Lisboa, Portugal; 385 Sahlgrenska Universityhospital, Gothenburg, Sweden; 386 Drug Allergy Diagnostics Centre, IPOG of NAMS, Kiev, Ukraine; 387 Clínica do Pulmão, Instituto Português de Oncologia do Porto, Porto, Portugal; 388 Hospital Universitario Cruces, Bilbao, Spain; 389 R&D Department, Bial-Aristegui, Spain; 390 Allergy and Pneumology Department, Hospital Quirón Campo de Gibraltar, Cádiz, Spain; 391 DCCU Écija, Osuna Primary Care Unit, Seville, Spain; 392 Allergy Department, Hospital Universitario Reina Sofía, Córdoba, Spain; 393 Department of Allergology, Clinical Immunology and Int. Diseases, L. Rydygier Collegium Medicum, Bydgoszcz NCU, Bydgoszcz, Poland; 394 Elias Emergency University Hospital, Bucharest, Romania; 395 Novo Medica Clinic, Bucharest, Romania; 396 Catholic University “Our Lady of Good Counsel”, Tirana, Albania; 397 Regional Hospital, Saranda, Albania; 398 “Mother Theresa” School of Medicine, Tirana, Albania; 399 Allergy Clinic, Copenhagen University Hospital, Gentofte, Denmark; 400 Department of Pediatrics, Herlev, Denmark; 401 Immunoallergology Universitary Department, Santa Maria Hospital, Lisbon Medical Academic Center, Lisbon, Portugal; 402 Psychiatric Department, Santa Maria Hospital, Centro Hospitalar Lisboa Norte, Lisbon, Portugal; 403 IRCSS San Martino, IST Genova UOC Allergologia, Genova, Italy; 404 Emergency Department, San Paolo Hospital Savona, Savona, Italy; 405 UMF CLUJ, Cluj-Napoca, Romania; 406 Carol Davila University of Medicine and Pharmacy, Bucharest, Romania; 407 Nicolae Malaxa Clinical Hospital, Bucharest, Romania; 408 Immunoallergology Department - Centro Hospitalar de Vila Nova de Gaia/Espinho (CHVNG/E), Vila Nova de Gaia, Portugal; 409 Guy’s Hospital, London, UK; 410 East Surrey Hospital, Redhill, UK; 411 Department of Clinical Immunology and Allergology of Danylo Halytsky, Lviv National Medical University, Lviv, Ukraine; 412 State Hospital 52, Kursk, Russia; 413 RMAPO, Moscow, Russia; 414 Faculty of Medical Science, Kragujevac, Serbia; 415 Children’s Hospital for Lung Diseases and Tuberculosis, Medical Center “Dr Dragiša Mišović”, Belgrade, Serbia; 416 Belgrade Institute for Emergency Medicine, Belgrade, Serbia; 417 Food To Fit Ltd, London, UK; 418 Allergy UK, Sidcup, UK; 419 University College Cork, Cork, Ireland; 420 Montreal Children’s Hospital, Montreal, QC Canada; 421 Department of Allergology, Rheumatology and Clinical Immunology, University Medical Center, University Children’s Hospital, Ljubljana, Slovenia; 422 Campden BRI, Chipping Campden, UK

## ORAL ABSTRACT SESSION 1: Food allergens • Anaphylaxis

### OP01 Fatal anaphylaxis is decreasing in France: analysis of national data, 1979–2011

#### Guillaume Pouessel^1,2,3^, Claire Claverie^4^, Julien Labreuche^5^, Jean-Marie Renaudin^3,6^, Aimée Dorkenoo^4^, Mireille Eb^7^, Anne Moneret-Vautrin^6^, Antoine Deschildre^2,3^, Stephane Leteurtre^4^

##### ^1^Department of Pediatrics, Children’s Hospital, Roubaix, France; ^2^Division of Pulmonology and Allergology, Department of Pediatrics, Faculty of Medicine and Children’s Hospital, Lille, France; ^3^Allergy Vigilance Network, Vandoeuvre les Nancy, France; ^4^Université Lille 2, CHU Lille, EA 2694 - Santé Publique: épidémiologie et qualité des soins, Lille, France; ^5^Biostatistic Unit, Maison Régionale de la Recherche Clinique, CHRU Lille, Lille, France; ^6^Department of Allergology, Emile Durkheim Hospital, Epinal, France; ^7^Centre d’Epidémiologie sur les Causes Médicales de Décès INSERM, CHU de Bicêtre, Le Kremlin-Bicêtre, France

**Correspondence**: Guillaume Pouessel - guillaume.pouessel@gmail.com

*Clinical and Translational Allergy* 2017, **7(Suppl 1)**:OP01

**Introduction**: Incidence of anaphylaxis is increasing. Data regarding anaphylaxis mortality are limited, but conflicting. Our objective was to document anaphylaxis mortality rate (deaths per million population), time trends and specificities according to triggers (iatrogenic, venom, food, unknown), age groups, sex and geographical regions (North and South) in France, between 1979 and 2011.

**Methods**: Data were obtained (1) from database of the National Mortality Center (CEPIDC) to collect cases in which anaphylaxis was included as a cause of death, sex, age, and geographic region of death, (2) from the database of the National Institute for Economical and Statistical studies (INSEE) to define the referent populations. We used a multivariable log-linear Poisson regression model to assess the impact of time period, age, sex and geographic region on anaphylaxis deaths.

**Results**: During the period study, 1603 deaths were collected: 1564 in adults and 39 in children (age <18 year). The overall prevalence of anaphylaxis fatalities was 0.84 per million population (95% IC 0.80–0.88), ranging from 0.08 per million (95% IC 0.05–0.10) in pediatric population to 1.12 per million (95% CI 1.06 to 1.17) in adult population. Annual percentage change for case fatality rate was −2.0% (95% CI −2.5 to −1.5; p < 10^−4^) indicating a decrease in case fatality rate during the study period. Anaphylaxis fatality rate was higher in men (1.08 per million [95% IC 1.00 1.16] than women (0.86 per million [95% IC 0.80–0.92]) (p < 10^−4^). Triggers of anaphylaxis fatalities were iatrogenic (63%), mostly drugs, venom (14%) and food (0.6%). Unspecified anaphylaxis was frequent (23%). The highest rate was in persons aged >70 years (3.50 per million population per year [95% IC 3.25–3.76]) and the lowest in the pediatric population (p < 10^−4^). Only venom-induced mortality rate was higher in South of France (0.16 per million [95% IC 0.13–0.19]) compared with the North (0.11 per million [95% IC 0.09–0.13]) (p = 0.004). Only 8 food-induced fatalities were recorded (age <35 years in 7 cases).

**Conclusion**: Overall anaphylaxis mortality rate is decreasing over the three last decades in France. We confirm that iatrogenic causes are the most frequent causes. Older age and male sex are risk factors of fatal anaphylaxis of any cause except for food-induced anaphylaxis.

### OP02 Diagnostic workup after severe anaphylaxis

#### Linus Grabenhenrich^1^, Margitta Worm^1^, Sabine Dölle^1^, Kathrin Scherer^2^, Isidor Hutteger^3^

##### ^1^Charité - Universitätsmedizin Berlin, Berlin, Germany; ^2^University Hospital Basel, Basel, Switzerland; ^3^Universitätsklinikum Salzburg, Salzburg, Austria

**Correspondence**: Linus Grabenhenrich - linus.grabenhenrich@charite.de

*Clinical and Translational Allergy* 2017, **7(Suppl 1)**:OP02

**Introduction**: After a severe anaphylactic reaction, a diagnostic workup is recommended to confirm or rule out the elicitor(s) in question. The type of diagnostic chosen is usually based on the elicitor and severity of the reaction and might follow local experiences. We aimed to describe elicitor-specific diagnostic habits in the workup of severe anaphylaxis, comparing European countries.

**Methods**: The Network for Online Registration of Anaphylaxis (NORA) collected details about elicitors, symptoms and severity, treatment and the diagnostic workup of patients who experienced at least one episode of severe anaphylaxis, as documented within medical records of participating tertiary referral centres.

**Results**: Between June 2011 and April 2016, the registry collected data for 6465 cases of severe anaphylaxis, 74% of which reported to know the elicitor, with a remaining 20% having only a suspicion and 6% cases of idiopathic anaphylaxis. The allergen was known and confirmed by a diagnostic test in 4410 (92% of known elicitors). Of these, 68% had a reaction to this allergen for the first time, and 32% reported at least one earlier reaction to the same allergen. In first-time reactors (n = 3001) 7% reported that the allergen was confirming by a diagnostic test already before this reaction, for food 14%, insects 3%, drugs 2%, and 80% for SIT-induced anaphylaxis. Of cases with recurrent anaphylaxis (n = 1409), 30% had a test confirming the allergen before the reported reaction, for food 44%, insects 16%, drugs 18%, and 91% for SIT-induced anaphylaxis. Of all diagnostically confirmed cases of food-induced anaphylaxis (n = 1555), 78% were assessed by a skin test (SPT, positive in 93%), 90% by specific IgE (sIgE, 94% positive), 27% tryptase (7% positive), and 13% underwent an oral food challenge (positive in 88%). Patients with anaphylaxis caused by drugs had the following tests (positives of these): SPT 88% (49%), sIgE 31% (46%), tryptase 48% (11%), and provocation 19% (68%). For reactions against insect venom: SPT 79% (84%), sIgE 98% (97%), and tryptase 93 (8%). Irrespective of the elicitor, SPTs were performed more often in Austria, Ireland and Greece (92, 96, and 99%, respectively), and less often in Italy (64%). Tryptase was almost never measured in Ireland, Greece and France, whereas determination of specific IgE was carried out similarly between European countries.

**Conclusion**: The choice of diagnostic measure depended on the elicitor and varied by country. Especially the assessment of tryptase is handled very differently between allergens in question and countries. These differences may indicate aspects of the diagnostic workup with a certain degree of ambiguity, which might benefit from further harmonization.

### OP03 Primary sensitisation versus co-sensitisation to hydrolysed wheat protein

#### Morten Christensen, Carsten Bindslev-Jensen, Charlotte Mortz

##### Department of Dermatology and Allergy Centre, Odense Research Center for Anaphylaxis (ORCA), Odense University Hospital, Odense, Denmark

**Correspondence**: Morten J. Christensen - morten.junker.christensen@rsyd.dk

*Clinical and Translational Allergy* 2017, **7(Suppl 1)**:OP03

**Introduction**: Wheat protein is responsible for various phenotypes of allergic diseases. More recently an increased number of immediate type 1 allergic reactions to hydrolyzed wheat proteins (HWP) have been reported.

The aim of this study was to characterize the clinical profile and evaluate patients with a case-history of anaphylaxis related to ingestion of a product containing HWP. Furthermore, to describe patients with other types of wheat allergy co-sensitized for HWP.

**Methods**: From May 2010 to August 2015 we investigated 56 patients (31 female, 25 male, mean age 39.0 years [1.5–77.2]) sensitized to commercialized HWP, either by specific immunoglobulin E (sIgE) (ThermoFischer, Uppsala, Sweden) and/or skin prick test (SPT). Based upon case-history patients were divided into 3 groups: (1) allergic reaction to ingestion of a HWP containing product (n = 9) (2) ingestion of a wheat product; WIA (n = 19), (3) ingestion of a wheat product in combination with exercise; WDEIA (n = 28). All patients were orally challenged with the incriminated food.

**Results**: The total positive rate of sIgE to HWP was 47/56 (83.9%), SPT 35/42 (83.3%) and BHR 22/42 (52.3%). Fourteen patients were triple positive to commercialized HWP of whom 7/9 patients in the HWP group. In total 9 (16%) patients were identified with a case-history of anaphylaxis related to a HWP containing product. Seven of 9 had a case-history to the same hydrolyzed wheat product (AMO Letbagt^®^). The average serum level of HWP-sIgE and the SPT were higher in patients with a case-history of HWP, respectively (median 5.3 kU/L ±6.8) (p < 0.05) and (median 6.0 mm ±4.1) (p < 0.05) compared to the WIA and WDEIA groups. A complete negative pattern was determined with specific wheat proteins normally associated with other phenotypes of wheat allergy, omega-5 gliadin (f416), gliadin (f98), High Molecular Weight (Tri a 26) and α-amylase trypsin inhibitor (Tri a 30). Basophil histamine release (BHR) for HWP was extremely positive in 8/9 HWP patients with activity retained to dilutions up to 10^−12^. The most striking finding was the ultrahigh sensitivity of BHR in diagnosing allergy to HWP. It is, however, interesting, that the HWP patient tolerates ingestion of unmodified wheat.

**Conclusion**: Reactivity to HWP seems to be confined to patients specifically sensitized to this heterogeneous group of products without concomitant allergy to normal wheat. Irrelevant co-sensitization is also seen in classical wheat allergy.

### OP04 Actual adrenalin treatment in a specialised clinical setting, compared to administration as recommended by a built-in algorithm in a severity scoring instrument in food allergy

#### Esben Eller, Henrik Fomsgaard Kjaer, Charlotte Mortz, Carsten Bindslev-Jensen

##### Odense University Hospital, Odense, Denmark

**Correspondence**: Esben Eller - esben.eller@rsyd.dk

*Clinical and Translational Allergy* 2017, **7(Suppl 1)**:OP04

**Introduction**: One of the most used severity scoring instruments, the Sampson 1–5, includes a built-in algorithm indicating symptoms which necessitate adrenalin administration. These include grade 5 anaphylactic symptoms, but also grade 3 and 4 such as laryngeal “puritus, tightness, or dysphagia” and lower respiratory symptoms such as “wheezing, dyspnea or cyanosis”. Our aim was to compare the recommended adrenalin administration with the actual administration in our clinic in relation to the underlying eliciting symptom.

**Methods**: Data from 2382 positive food challenges (mean age 11.6 years [range: 0.5–74.1y]) performed between Jan. 2000 and Dec. 2015 at the Allergy Centre, Odense, Denmark were included, and severity of reactions was assessed using the Sampson 1–5 severity instrument. All patients were evaluated by experienced specialists during challenge. Actual medications administered during the challenges, i.e. adrenalin, β2-agonist, corticosteroid, or antihistamine were compared with recommended adrenalin treatment according to the algorithm in Sampson 1–5.

**Results**: Out of 346 challenges scored as grade 4 anaphylaxis, 296 were terminated due to respiratory symptoms requiring adrenalin according to Sampson 1–5, i.e. “barky cough, hoarseness, difficulty swallowing” (laryngeal, n = 79), “wheezing, dyspnea, cyanosis” (lower resp. n = 181) or both (n = 36). Nine of the 115 patients with laryngeal symptoms were treated with adrenalin, all due to inspiratory stridor. No patients with lower respiratory symptoms received adrenalin, but the majority were treated with β2 agonists (188/217), whereas in 30 challenges, symptoms disappeared without treatment or only antihistamine for concomitant urticaria were used. Patients solely with laryngeal symptoms received β2-agonists in 16 challenges, but the majority of them (54/79) received no treatment or only antihistamine. The 36 patients with both laryngeal and lower respiratory symptoms were treated in same manner as patients with only lower respiratory symptoms, i.e. β2 agonist for their bronchial wheeze or asthma. Grade 5 anaphylaxis was seen in 11 challenges, 1 caused by non-adrenalin recommended “loss of bowel control”. In the remaining 10 cases, 7 patients were treated with adrenalin, either due to “hypotension < 90 mm Hg” (n = 3) or “unconsciousness” (n = 4). Three children fainted, but regained consciousness without administration of adrenalin. Grade 5 anaphylaxis should almost always be treated with adrenalin, whereas adrenalin only was administrated to inspiratory stridor and not to bronchial expiratory wheeze or asthma in grade 4 anaphylaxis. Respiratory signs were instead medicated according to symptoms, i.e. with β2-agonist to relieve bronchoconstriction. All patients were evaluated by experienced specialists, and therefore this practice should be addressed with care in less experienced settings.

**Conclusion**: Inspiratory stridor was the main cause of adrenalin treatment in grade 4 anaphylaxis, whereas the majority of lower respiratory symptoms were treated with inhalant β2 agonists, thereby overcoming the need for adrenalin. This needs to be considered in future treatment recommendations.

### OP05 Do patients know how to use adrenaline auto-injectors?

#### Leonor Carneiro-Leão^†^, Jenny Badas^†^, Luís Amaral, Alice Coimbra

##### Serviço de Imunoalergologia, Centro Hospitalar de São João, Porto, Portugal

^†^The first two authors have equal contribution.

**Correspondence**: Leonor Carneiro-Leão - leonorcarneiroleao@gmail.com

*Clinical and Translational Allergy* 2017, **7(Suppl 1)**:OP05

**Introduction**: Adrenaline auto-injectors (AAI) are the first line treatment for anaphylaxis in community settings. Two are currently available in Portugal (Anapen^®^ and Epipen^®^).

Our aim was to evaluate patient’s ability to properly use AAIs; impact of device switching and patients’ preferences.

**Methods**: Patients who had been prescribed an AAI in our department were invited to demonstrate correct technique of AAI by simulating adrenaline administration using training devices. First, simulation with their prescribed AAI; second, evaluation of device switching, without any previous training, by simulating injection with a different AAI (Epipen^®^ or Anapen^®^, as well as Emerade^®^-currently unavailable in Portugal). Finally, they were asked which device they liked the best.

**Results**: Thirty-two patients were enrolled, 16 (50%) females, with a mean (SD) age of 42.9 (±15.8) years; 18 (56%) with hymenoptera venom allergy and 14 (44%) food allergy. Anapen^®^ was prescribed to 15 (47%) and Epipen^®^ to 17 (53%). Six did not acquire any AAI; 21 (66%) admitted carrying it on a daily basis. Eleven (34%) could not demonstrate successful adrenaline administration with their prescribed AAI, 5 with Anapen^®^ and 6 with Epipen^®^. Nine (60%) of the 15 patients who were prescribed an Anapen^®^ could not administer adrenaline with an Epipen^®^; 11 (65%) of the 17 with a prescribed Epipen^®^ were unable to use an Anapen^®^. Only 2 (6%) were incapable of properly managing an Emerade^®^. The most common error in patients switching from Epipen^®^ to Anapen^®^ was not removing the needle cap (9 patients). In the group switching Anapen^®^ to Epipen^®^, the most common misuse was not massaging the injection site (10 patients); 6 tried to remove the orange tip as if it was a cap. The preferred AAI was Emerade^®^ in 20 (63%) and Epipen^®^ in 12 (37%).

**Conclusions**: Patients at-risk for anaphylaxis are provided with portable auto-injectors, educated and trained on their use. One-third of the patients did not always carry them. More than one-third was unable to successfully demonstrate adrenaline administration with their prescribed AAI. Almost two-thirds failed to simulate injection when switched to the alternative one available in Portugal without any training. Design appears to play a role in a successful switch since 94% of the patients changing from either Anapen^®^ or Epipen^®^ to Emerade^®^ were able to correctly use it. It was also the overall preferred auto-injector. These emergency medical devices should be patient friendly.

### OP06 Incidence, clinical features, triggers and management of anaphylaxis in the Pediatric Emergency Department of the Tel Aviv Medical Center

#### Dikla Pivko Levy^1^, Moshe Ben-Shoshan^2^, Ayelet Rimon^1^, Shira Benor^1^

##### ^1^Tel Aviv Sourasky Medical Center, Tel Aviv, Israel; ^2^Montreal Children’s Hospital, Montreal QC, Canada

**Correspondence**: Dikla Pivko Levy - diklapivko@gmail.com

*Clinical and Translational Allergy* 2017, **7(Suppl 1)**:OP06

**Introduction**: Anaphylaxis is a severe, life threatening systemic hypersensitivity reaction. The diagnosis of anaphylaxis is not always easy to make in the pediatric emergency department (ED) setting. Therefore, children are often dangerously underdiagnosed and undertreated. There is sparse information on the incidence and triggers of anaphylaxis in Israel.

Our objective was to assess the true incidence of anaphylaxis treated in the Pediatric ED, to identify triggers associated with anaphylaxis, to describe the management of anaphylactic reactions and identify potential gaps in diagnosis and treatment.

**Methods**: A retrospective chart review of cases presenting to the Pediatric ED of the Dana-Dweck Children’s hospital, at the Tel Aviv Sourasky Medical Center between January 1st 2013 to December 31th 2014, with a diagnosis of anaphylaxis or allergic reaction. The clinical features, causative agents, treatment administered and recommendations at discharge were recorded.

**Results**: During the study period, there were a total of 56,596 visits to the ED. 437 patients were diagnosed with an allergic or anaphylactic reaction. Of these 59 (13.5%) met the diagnostic criteria for anaphylaxis, but only 22 were given the correct diagnosis. The mean age of presentation was 6.9 years, with a male predominance of 66%. Food was the most common causative agent (78%). Specifically, exposure to treenuts (28% (and cow milk (24%) were responsible for a majority of the cases. The majority of children (78% (had known food allergies and presented with breathing difficulties (64%), followed by urticaria (62%). Twenty children (37.7%) were treated with IM adrenaline prior to ED arrival and only fifteen (26%) were treated with IM adrenaline in the ED. Most of the children (86%) were discharged home. Almost 30% were discharged without a prescription to an automated Adrenaline injector.

**Conclusion**: The rate of anaphylaxis in the study period was 0.1% of all visits to the pediatric ED. Most cases of anaphylaxis were underdiagnosed. As a result, treatment guidelines regarding the use of IM Adrenalin were not always followed and many children were discharged without a prescription for an adrenaline auto-injector.

## ORAL ABSTRACT SESSION 2: Clinical aspects • Diagnosis and treatment

### OP07 Almond allergy in a cohort of Dutch atopic children: results from 189 oral food challenges with almond

#### Nicolette J. T. Arends^1^, Nikki Edelbroek^1^, Hans de Groot^2^, Joyce A. M. Emons^1^, H. Kim A. Brand^1^, Dirk Verhoeven^2^, Leonieke N. van Veen^2^, Nicolette W. de Jong^1^

##### ^1^Erasmus MC Sophia Children’s Hospital - Kinderhaven, Rotterdam, the Netherlands; ^2^Pediatric Department, Reinier de Graaf Groep, Delft, the Netherlands

**Correspondence**: Nicolette J. T. Arends - n.arends@erasmusmc.nl

*Clinical and Translational Allergy* 2017, **7(Suppl 1)**:OP07

**Introduction**: Tree nut allergies are common in children, whereas most reported allergic reactions are caused by hazelnut and cashew nut [1,2]. Reactions to these nuts may vary from mild oral allergy symptoms to anaphylaxis. Not much is known about the frequency and severity of almond allergy in children. We therefore evaluated the results of oral food challenges with almond in a large group of Dutch atopic children.

**Methods**: All open and double-blind placebo-controlled food challenges (DBPCFC) with almond, performed between 2009 and 2015 in two Dutch outpatient clinics were evaluated retrospectively. Skin prick tests (SPT) with almond were performed in most children. Information about previous reactions, reasons for avoidance of almond and presence of atopic diseases (eczema, asthma, allergic rhinitis) were taken from the medical records.

**Results**: A total of 189 almond challenges were analyzed. Median age of the children was 7.5 years (range 2.0–17.8 years). Almond was removed from the diet for the following reasons: a previous reaction to almond (3.7%), previous reaction to another nut (30.7%), previous reaction to peanut (22.2%), other food allergies (23.8%), a positive test (sensitization) to almond (12.2%), eczema (3.7%), allergic parents (1.1%) and unknown (2.6%). A positive SPT almond was found in 148 children (78.3%). SPT was negative in 28 children (14.8%) and not performed in 13 children (6.9%). 97/101 DBPCFC’s were negative, 2/101 children had a mild reaction and 2/101 children had a doubtful reaction. 86/88 open challenges were negative, 1 child had a mild reaction and 1 child had a doubtful reaction. Reactions were treated with antihistamine. No correlation was found between the outcome of the challenge and SPT results. Sensitization to birch pollen was found in 109 children (57.7%). Sensitization to almond is frequently found in Dutch atopic children. This study shows that most of these sensitizations appear to be clinically irrelevant. Only 6/189 children (3.2%) had a mild reaction and no anaphylaxis was seen. Sensitization might be explained partly by cross-sensitization with birch pollen.

**Conclusion**: In a large cohort of Dutch atopic children, almond allergy is extremely rare and allergic reactions are only mild.

**References**

1. McWilliam V, Koplin J, LodgeC, Tang M, Dharmage S, Allen K. The prevalence of tree nut allergy: a systematic review. Curr Allergy Asthma Rep. 2015;15:54.
2. Grabenhenrich L, et al. Anaphylaxis in children and adolescents: The European Anaphylaxis Registry. J Allergy Clin Immunol 2016;137:1128–37.

### OP08 Oral tolerance induction using IFN-gamma in patients with anaphylactic food allergy (AFA), non-IgE-mediated food allergy (NFA) in atopic dermatitis (AD) and both AFA and NFA in AD

#### Geunwoong Noh^1^, Eun Ha Jang^2^

##### ^1^Department of Allergy, Jeju Halla General Hospital, Jeju, Korea Republic; ^2^Department of Respiratory Medicine, Hanmaeum General Hospital, Jeju, Korea Republic

**Correspondence**: Geunwoong Noh - admyth@naver.com

*Clinical and Translational Allergy* 2017, **7(Suppl 1)**:OP08

**Introduction**: Food allergy is assessed generally by IgE-mediated laboratory tests. For NFA, gastrointestinal allergy is mainly considered. However, NFA which appears as eczema in atopic dermatitis is also frequent. In this report, typical three groups for food allergy was presented, patients with anaphylactic food allergy (AFA) as a representative IgE-mediated food allergy, patients with NFA which are presented as eczema in AD and patients who had both AFA and NFA in AD. Oral immunotherapy (OIT) using IFN-gamma was conducted successfully in these three groups. The different diagnostic and therapeutic approaches according to the type of food allergy and the clinical and immunological significance are presented.

**Case report**: Two patients had AFA. Specific IgE for causative foods like milk or eggs are very high and skin prick test for causative foods is also strong positive. AFA was confirmed by oral food challenges. Patients received OIT using IFN-gamma according to the protocol and finally they got the tolerance for causative foods completely. Five patients had food allergy which symptoms is appearing as eczema as AD. Specific IgE and skin prick test for causative foods were negative. Oral food challenges were performed and the appearance of the symptoms and signs of AD is confirmed and the diagnosis was made as NFA as causes of AD. Patients received OIT using IFN-gamma according to the relevant protocol, successfully. Two patients had NFA in AD and AFA. The some foods caused AD by showing eczematous reaction and the other different food provoked anaphylactic reactions, together. The specific IgE and skin prick test for the causative foods is very high for the food allergens of AFA and those for the foods were negative for NFA in AD. OFC was done for the causative foods of AFA and NFA. Patients received OIT using IFN-gamma by the compatible protocol according to the types of food allergy successfully.

**Conclusion**: Food allergy may be assessed by differentiation as food allergies of IgE- and non-IgE-mediated type. The diagnostic and therapeutic approaches are different according to the types of food allergy. The immunopathogenesis and clinical approach should be done according to the differential diagnosis.

**Consent to publish**: Written consent provided for publication of this abstract.

### OP09 Predicting fish allergy outcome and assessing tolerance at home in a children’s population

#### Mariona Pascal^1^, Olga Dominguez^2^, Mònica Piquer^2^, Montserrat Alvaro^2^, Rosa Jimenez-Feijoo^2^, Jaime Lozano^2^, Adriana Machinena^2^, Maria del Mar Folqué^2^, Maria Teresa Giner^2^, Ana María Plaza^2^

##### ^1^Immunology Department, CDB Hospital Clinic de Barcelona, Barcelona, Spain; ^2^Department of Allergy and Clinical Immunology, Hospital Sant Joan de Déu, Esplugues de Llobregat, Spain

**Correspondence**: Mariona Pascal - mpascal@clinic.ub.es

*Clinical and Translational Allergy* 2017, **7(Suppl 1)**:OP09

**Introduction**: Fish allergy is relevant in our pediatric population on a Mediterranean diet. Specific IgE (sIgE) tests aid in diagnosis, but oral food challenge is the gold standard. We sought to: (1) analyze the efficiency of sIgE to Gad c 1 and fish whole extracts to avoid challenge and (2) to evaluate maintenance of tolerance of challenged patients at home.

**Methods**: Children sensitized to fish and/or Gad c 1 reporting or not clinical history of fish allergy were challenged (masked single blind, 16 g protein for ≤12 year-old children and 22 g for older ones). Clinical history was reviewed and sIgE to Gad c 1, fish extracts (ImmunoCAP, ThermoFisher) and challenge test outcomes were analyzed. Tolerance at home was investigated.

**Results**: 83 patients (67.5% male, median [range] age at OFC: 8[2–15] years-old) were analyzed. 9.6% were only sensitized. Among those reporting symptoms, 26% were anaphylaxis, 43.4% urticaria, 4.8% atopic dermatitis, 8.4% gastrointestinal symptoms, 2.4% dyspnea. All patients were challenged. A total of 221 challenges were done: 71 canned tuna (CT), 72 fresh tuna (FT), 48 hake and 30 sole. Challenge was positive (OFC+) in 2.2% of patients for CT, 12.5% FT, 47.9% hake and 40% for sole. Gad c 1 sIgE was significantly higher in OFC+ patients for FT, hake and sole (p = .0031, <.0001, .0003, respectively) comparing with OFC-ones. Similarly occurred with sIgE to the corresponding extracts in the case of hake and sole (p = .0014 and .0015, respectively). ROC-curve analysis of Gad c 1 and whole extracts tests provided sIgE cut-offs to predict OFC+: Gad c 1: >37.5 kU/L for OFC with FT (AUC:0.80, LH:6.9), >4.7 kU/L for OFC with sole (AUC:0.91, LH:6.6), >3.5 kU/L for OFC with hake (AUC:0.88, LH:7.1). Tuna extract sIgE >15.5 kU/L for OFC with FT (AUC:0.78, LH:6.4), hake extract sIgE >23.5 kU/L for OFC with hake (AUC:0.83, LH:6.6), sole extract sIgE >1.1 kU/L for OFC with sole (AUC:0.89, LH:7).The follow up of tolerance at home showed that 64 (77.1%) patients were not eating the challenged food at home, 25 (39%) mainly for fear or refusal. A total of 49 reactions, of which 17 (34.7%) anaphylaxis, occurred in 39 patients (7 children with several fish species).

**Conclusion**: Certain sIgE cut-offs for Gad c 1, tuna, hake and sole extracts may aid in fish allergy diagnosis and predicting OFC outcome in our pediatric population. A significant proportion of children that tolerate fish at challenge, suffer allergic reactions when eating fish at home, some of them severe.

### OP10 Development and validation of an app to monitor reactions during Oral ImmunoTherapy (OIT)

#### Paul Turner^1^, Nandinee Patel^1^, Marta Vazquez-Ortiz^1^, Sarah Lindsley^1^, Lucy Walker^2^, Simon Rosenberg^2^

##### ^1^Imperial College London, London, United Kingdom; ^2^Illuminatis Ltd, London, United Kingdom

**Correspondence**: Nandinee Patel - paulyt@doctors.org.uk

*Clinical and Translational Allergy* 2017, **7(Suppl 1)**:OP10

**Introduction**: Until recently, food allergy management involved complete allergen avoidance, however, data now implies that this may not be required - for example, with regard to extensively heated egg or cow’s milk e.g. in cakes/biscuits. In many countries, oral immunotherapy (OIT) is used as a treatment modality for food allergy, particularly to cow’s milk and increasingly, peanut. However, adverse events are common (occurring in up to 80% of patients). There is a need to develop systems to facilitate safe OIT and allow real-time communication between patients and their treating allergist. Almost 90% of patients/parents now own a smartphone, which can be used to facilitate communication between patients and the clinical team.

**Methods**: We adapted a digital App called “Tell the Doctor” to allow real-time reporting of symptoms (adverse events, AEs) occurring outside the hospital environment. The App is being tested in an OIT trial in 46 peanut-allergic children. Focus groups were held to collect feedback from study participants, their parents, and members of the study team.

**Results**: The app was welcomed by both study participants and their families. Feedback was obtained relating to the following areas:

- AE reporting was modified to allow rapid and instant alerts to the study team of any significant reactions occurring at home.
- Neurological/behavioural changes were separated from cardiovascular symptoms, as there was no evidence that the former are related to the latter.
- Study participants were asked to confirm OIT doses taken on a daily basis, thus confirming adherence to study protocol. Patients could also opt-in to a reminder service to prompt them to take both their OIT dose and any asthma preventer medicines by a time of their choosing.

**Conclusion**: The app facilitates real-time reporting of AEs during OIT studies and was preferred by participants and study team compared to delayed manual paper reporting. We expect electronic reporting to improve the data integrity of OIT-related AEs, and simplify AE reporting, thus improving safety. The advantages of using contemporary/popular communication modalities such as smartphone apps should be considered in the performance of OIT, whether in research or in clinical practice.

**Acknowledgements**: “Tell the Doctor” App funded by the Nominet Trust.

**Trial registration**: ClinicalTrials.gov Identifier: NCT02149719

**Consent to publish**: Informed consent was obtained, NHS HRA approval 15/LO/0287.

### OP11 Introducing FABER test for allergy diagnosis: food molecule- and extract-based allergenic preparations in the newest and broadest nanotechnology IgE test

#### Adriano Mari^1^, Claudia Alessandri^1^, Ivana Giangrieco^2^, Lisa Tuppo^2^, Chiara Rafaiani^1^, Georg Mitterer^3^, Michela Ciancamerla^1^, Rosetta Ferrara^1^, Maria Livia Bernardi^1^, Danila Zennaro^1^, Maurizio Tamburrini^2^, Maria Antonetta Ciardiello^2^, Christian Harwanegg^3^

##### ^1^Centri Associati di Allergologia Molecolare (CAAM), Rome, Italy; ^2^Istituto di Bioscienze e Biorisorse, Consiglio Nazionale delle Ricerche, Naples, Italy; ^3^MacroarrayDx, Vienna, Austria

**Correspondence**: Adriano Mari - adriano.mari@caam-allergy.com

*Clinical and Translational Allergy* 2017, **7(Suppl 1)**:OP11

**Introduction**: Multiplex tests allow to detect specific IgE to several different preparations at once. They allow patient’s profiling tailoring decisions for interventions. The last ten years have seen the availability of new technologies and when combined can lead to increase diagnostic information from allergy tests.

Our aim was to report about the FABER nanotechnology-based test in food allergy diagnosis.

**Methods**: FABER 244 IgE test is a new multiplexed in vitro test for specific IgE measurement having 122 molecular allergens and 122 allergenic extracts. Allergenic molecules and extracts, produced in house or obtained from top quality providers in the field, are coupled to chemically activated nanoparticles. Coupling is individually optimized to achieve maximum test performance providing high diagnostic accuracy for each spotted allergenic item. Once coupled they are arrayed to a solid phase matrix to form a one-step comprehensive array based testing solution, using 100 ul of patient serum or plasma.

**Results**: Extracts from 91 food-borne allergenic sources (fruits, vegetables, eggs, milks, meats, fishes, crustacean, mollusks, snails, mushrooms, anisakis) are arrayed together with 66 allergenic proteins obtained from the same sources. CCD-bearing proteins are included as markers to support test result interpretation, as well as allergenic molecular groups which cross-sectionally belong to food and inhalant sources. Extracts on FABER244 expand the panel overcoming missing of any not yet identified or available allergenic molecule, increasing diagnostic accuracy and comprehensiveness. Test interpretation is supported by CAAM Digital Reporting System (CDRS), a unique online tool available worldwide, allowing visualization on mobile devices of FABER test results. CDRS has been developed for patients to familiarize with the new extended molecule-based results. To be patient-friendly it uses local languages taking advantage of the Allergome platform as the multi-language source. Data on CDRS are shown with tables, graphs, images; comments are generated real time by experts using the Allergome and its external modules, InterAll and ReTiME.

**Conclusion**: FABER 244 is the most advanced in vitro test for specific IgE detection, including molecules and extracts. It makes available to the molecular allergist an unprecedented quantity of data. The inclusion of allergenic extracts is strategic to confirm or complement results obtained with the single allergenic molecules.

## ORAL ABSTRACT SESSION 3: Experimental aspects • Food allergens

### OP13 New developments in the allergenicity assessment of food derived from biotechnology

#### Antonio Fernandez^1^, Regina Selb^1^, Philippe Egenmann^2^, Michelle Epstein^3^, Karin Hoffmann-Sommergruber^3^, Frits Koning^4^, Martinus Lovik^5^, Clare Mills^6^, Javier Moreno^7^, Henk van Loveren^8^, Jean-Michel Wal^9^

##### ^1^European Food Safety Authority, Parma, Italy; ^2^University Hospitals of Geneva, Geneva, Switzerland; ^3^Medical University of Vienna, Vienna, Austria; ^4^Leiden University Medical Center (LUMC), Leiden, the Netherlands; ^5^Norwegian University of Science and Technology (NTNU), Trondheim, Norway; ^6^The University of Manchester (UNIMAN), Manchester, United Kingdom; ^7^Consejo Superior de Investigaciones Científicas (CSIC), Madrid, Spain; ^8^Maastricht University, Maastricht, the Netherlands; ^9^Institut National de la Recherche Agronomique (INRA), Paris, France

**Correspondence:** Antonio Fernandez - antonio.fernandezdumont@efsa.europa.eu

*Clinical and Translational Allergy* 2017, **7(Suppl 1)**:OP13

**Introduction**: The European Food Safety Authority (EFSA) and other international bodies (Codex) define approaches for allergenicity assessment of food and feed derived from biotechnology. As an outcome of the allergenicity assessment, risk assessors estimate whether the novel protein is likely to be allergenic and whether the food derived from biotechnology is likely to be more allergenic than that derived from its appropriate comparator(s). Because it is challenging to predict the allergenicity of novel proteins, a weight-of-evidence approach is used to provide the assessor with a cumulative body of evidence to (a) reduce the uncertainty linked to the allergenicity assessment and, (b) enhance the reliability of predictions regarding the allergenic potential of novel protein(s).

**Methods**: Currently, EFSA is developing supplementary guidance to better define and clarify specific aspects of the allergenicity assessment requirements. In particular, (i) non-IgE-mediated immune adverse reactions to foods; (ii) *in vitro* protein digestibility; and (iii) endogenous allergenicity, are addressed.

**Results**: Firstly, celiac disease is a well characterised non-IgE-mediated adverse immune reaction to food, and the food proteins involved, as well as the underlying molecular mechanisms, have been described in detail. Secondly, the outcome of *in vitro* protein digestibility studies is considered relevant information in the weight-of-evidence approach. To date, the “pepsin resistance test” is commonly accepted for the safety assessment considerations by risk assessors. However, EFSA has previously highlighted its limitations for the allergenicity assessment as well as for its capacity to reflect *in vivo* digestion conditions. Thirdly, high performance methodologies for protein identification and quantification are available for endogenous allergenicity.

**Conclusion**: Firstly, based on the current knowledge EFSA is working on defining a strategy to be followed for the assessment of novel proteins’ potential to cause celiac disease. Secondly, EFSA is proactively developing a complementary strategy in order to reduce the resulting uncertainty in the allergenicity assessment. This strategy will be based on state-of-the-art in science, aiming at proposing an enhanced and refined *in vitro* gastrointestinal digestion test where different physiological conditions will be taken into consideration and more informative read-out procedures will be recommended. Thirdly, high performance methodologies for protein identification and quantification will be proposed as complementary/alternative methods to those based on human sera for the assessment of endogenous allergenicity within the comparative assessment analysis.

### OP14 Why mice orally sensitised to OVA using antiacids react anaphylactic or not: possible explanation by colonisation with distinct bacterial strains

#### Susanne Diesner^1,2^, Cornelia Bergmayr^1^, Barbara Pfitzner^3^, Vera Elisabeth Assmann^1^, Philipp Starkl^1^, David Endesfelder^4^, Thomas Eiwegger^2,5^, Zsolt Szepfalusi^2^, Heinz Fehrenbach^6^, Erika Jensen-Jarolim^1,7^, Anton Hartmann^3^, Isabella Pali-Schöll^1,7^, Eva Untersmayr^1^

##### ^1^Department of Pathophysiology and Allergy Research, Center of Pathophysiology, Infectiology and Immunology, Medical University of Vienna, Vienna, Austria; ^2^Department of Pediatrics and Adolescent Medicine, Medical University of Vienna, Vienna, Austria; ^3^Research Unit Microbe-Plant Interactions, Research Group Molecular Microbial Ecology, Department of Environmental Sciences, Helmholtz Zentrum München, German Research Center for Environmental Health, Neuherberg, Germany; ^4^Scientific Computing Research Unit, Helmholtz Zentrum München, German Research Center for Environmental Health, Neuherberg, Germany; ^5^Division of Immunology and Allergy, Food Allergy and Anaphylaxis Program, Department of Pediatrics, Hospital for Sick Children, Research Institute, Physiology and Experimental Medicine, University of Toronto, Toronto ON, Canada; ^6^Priority Area Asthma & Allergy, Research Center Borstel, Airway Research Center North (ARCN), German Center for Lung Research (DZL), Borstel, Germany; ^7^Messerli Research Institute of the University of Veterinary Medicine Vienna, Medical University Vienna and University of Vienna, Vienna Austria

**Correspondence**: Eva Untersmayr - eva.untersmayr@meduniwien.ac.at

*Clinical and Translational Allergy* 2017, **7(Suppl 1)**:OP14

**Introduction**: In an oral mouse food allergy model, concomitant gastric acid suppression is associated with formation of antigen-specific IgE and anaphylaxis. Notably, we repeatedly observed non-responder animals protected from food allergy. Therefore, in this study we aimed to analyse the reasons for this protection.

**Methods and Results**: Out of 64 BALB/c animals being subjected to the oral ovalbumin (OVA) immunization protocol under gastric acid-suppression, 10 animals (16%) did not show any elevation of OVA-specific IgE or IgG1 titers indicating protection from allergic sensitization. In these animals, allergen challenges confirmed reduced antigen uptake and lack of anaphylactic symptoms, while in the non-protected allergic mouse group high levels of mouse mast cell protease-1 (mMCP-1) and a drop of core body temperature were elicited, indicative for anaphylaxis. Further, significantly lower numbers of CD4+ T cells and regulatory T cells were detected in the non-responders, as well as significantly lower levels of IL-4, IL-5, IL-10 and IL-13 in supernatants from stimulated splenocytes, but comparable levels of IL-22. This was accompanied by significantly increased numbers of total lymphocytes and reduced numbers of monocytes, erythrocytes and hematocrit in the peripheral blood of the non-responders. Comparison of microbiota finally revealed differences regarding the composition of bacterial communities on single bacterial Operational Taxonomic Unit (OTU) level between protected and allergic mice.

**Conclusion**: These data clearly indicate that protection from food allergy development was associated with significantly reduced Th2 cytokine levels and IL-10, and increased numbers of blood cells in the periphery after anaphylaxis. Most importantly, analysis of single bacterial OTUs indicated that a distinct microbiota composition was associated with a non-responding phenotype in this mouse model. The data propose that also microbiota might decide on the extent of a food-anaphylactic response.

**Acknowledgements**: Supported by FWF grants P21884, P21577 and KLI284 of the Austrian science fund FWF. HF is supported by the Deutsche Forschungsgemeinschaft (Cluster of Excellence “Inflammation at Interfaces” EXC 306).

### OP17 Allergy to fenugreek – A new food allergy in peanut allergic children in Sweden

#### Soren Wille^1,2^, Peter Meyer^1^

##### ^1^Department of Pediatrics, Helsingborg Hospital, Helsingborg, Sweden; ^2^Department of Pediatric Allergy, Skåne Universiy Hospital, Malmö, Sweden

**Correspondence**: Soren Wille - sorenwille@gmail.com

*Clinical and Translational Allergy* 2017, **7(Suppl 1)**:OP17

**Introduction**: To study an increasing number of peanut allergic children with allergic reactions after having eaten food with curry or other mixed spices. All were sensitized to fenugreek (*Trigonella foenum graeceum)*.

**Methods**: We have collected and reviewed data from 13 patients during the last 5 years from our outpatient allergy departments. Eleven had reacted to food with curry and 2 to food with fajitas spice mixture. All were sensitized to and regarded as allergic to fenugreek, an ingredient in both curry and the spice mixtures. Five reacted with an anaphylactic reaction. Six reported allergy symptoms such as urticaria, itching in the mouth, abdominal pain, vomiting and asthma.

**Results**: Mean age for the first reaction, when known, was 9 years. Seven boys and four girls. All 13 children were sIgE-positive to both fenugreek and to peanut as well as one or more of the recombinant peanut allergens Ara h 1, 2 or 3. Seven tested for Ara h 1 were all positive, in six of seven the dominant allergen was Ara h 1. The sIgE-results will be presented in detail. Our patients have not yet had a food provocation test. Fenugreek belongs to the Fabaceae plant family (legumes). The dried seeds are used whole or ground to a yellow powder after roasting, most commonly as a spice in curry and other mixed spices but also as an ingredient in a variety of food. In the literature fenugreek allergy was first described 1993, a case with occupational asthma. We have found only two reports about fenugreek food allergy. In Norway it is recommended to warn peanut allergic patients against food containing fenugreek and lupin. In Sweden there is no such warning either from our pediatric allergy society nor from The Swedish National Food Agency, but fenugreek as well as other peanut cross-reactive allergens are mentioned as a potential risk for allergic reactions. We plan to review of the history of reactions to curry or spices and add sIgE tests in follow up of our peanut allergic patients. The results may change our opinion about which advice to give to peanut allergic patients about fenugreek and mixed spices, eg curry.

**Conclusion**: For patients with fenugreek allergy and for cautious peanut allergic patients it is a problem that fenugreek is not on the list of ingredients that should be declared according to the food labelling directive from the European Union and The Swedish National Food Agency.

### OP18 Pru p 7 is a major peach allergen in patients from Southern France

#### Caroline Klingebiel^1^, Jonas Lidholm^2^, Angelica Ehrenberg^2^, Jonas Östling^2^, Isabelle Cleach^3^, Jean-Louis Mège^3,4^, Joana Vitte^3,4^

##### ^1^Laboratoire Montgrand, LBM Multisite SELDAIX - BIOPLUS, Marseille, France; ^2^Thermo Fisher Scientific, Uppsala, Sweden; ^3^Laboratoire d’Immunologie, Hôpital de la Conception, Assistance Publique Hôpitaux de Marseille, Marseille, France; ^4^Aix-Marseille Université, Marseille, France

**Correspondence:** Caroline Klingebiel - jvitte@hotmail.fr

*Clinical and Translational Allergy* 2017, **7(Suppl 1)**:OP18

**Introduction**: To assess frequency and magnitude of IgE sensitization to Pru p 7 sIgE in patients complaining of peach allergy.

**Methods**: Sera from 117 outpatients (median age 20 years, range 4–74; 44% males) having undergone sIgE work-up for peach extract and commercial component-resolved diagnostics (ImmunoCAP 250, Thermo Fisher Scientific, Uppsala, Sweden) between February 2012 and June 2016 were assayed for sIgE to recombinant Pru p 7. The positivity threshold was set at 0.10 kUA/L.

**Results**: Of the 117 patient sera analysed, 111 (95%) tested positive to peach extract, 73 of which (66%) displayed sIgE reactivity to rPru p 7. In 45/68 (66%) cases, sIgE reactivity to Pru p 7 was isolated (no detection of sIgE to rPru p 1, rPru p 3, or rPru p 4). Among the 6 peach negative sera, one was found positive to rPru p 7 (0.81 kUA/L). Quantitative analysis showed that levels of sIgE in the study population were higher to Pru p 7 than to peach extract (median 5.5 vs 1.3 kUA/L, range 0.10 to 30.7 vs 0.10 to 52.4, *p* = 0.003, coefficient of correlation -0.12). 38/111 (32%) sera with detectable sIgE to peach extract did not display sIgE to rPru p 7, and 3 of these sera (peach sIgE 0.18 to 0.20 kUA/L) did not display sIgE to any of rPru p 1, rPru p 3, rPru p 4, or MUXF3. Pru p 7 has been reported as a major allergen in peach allergic patients from Spain and Italy, and Pru p 7 sensitization appears to correlate with a specific clinical presentation in Japanese patients. Here, we bring evidence that Pru p 7 is a major allergen in peach-sensitized patients from Southern France, including those with low sIgE to peach extract and seemingly independent of sIgE reactivity to peach components currently available for diagnostic testing. Significant association with cypress pollinosis and severe peach-induced allergy have been observed in rPru p 7 sensitized patients.

**Conclusion**: Pru p 7 is needed for comprehensive component-resolved diagnostics of peach allergy in Mediterranean patients. Pru p 7 sensitization may be a prognostic factor of peach allergy severity. From a pathophysiological perspective, Pru p 7 may shed light on the as-yet obscure relationship between peach and cypress pollen allergy in the Mediterranean region.

## POSTER DISCUSSION SESSION 1: Food allergens • Anaphylaxis

### PD01 Comparison of the lipid-binding capacity and immunoreactivity of Pru p 3 and Mal d 3

#### Roberta Aina^1^, Pawel Dubiela^1^, Sabine Pfeifer^1^, Merima Bublin^1^, Christian Radauer^1^, Piotr Humeniuk^1^, Stefan Kabasser^1^, Riccardo Asero^2^, Karin Hoffmann-Sommergruber^1^

##### ^1^Department of Pathophysiology and Allergy Research, Medical University of Vienna, Vienna, Austria; ^2^Clinica San Carlo, Paderno Dugnano, Italy

**Correspondence:** Roberta Aina - roberta.aina@meduniwien.ac.at

*Clinical and Translational Allergy* 2017, **7(Suppl 1)**:PD01

**Introduction**: nsLTPs are pathogenesis-related proteins (PR-14) with antimicrobial activity, and represent important plant food allergens, especially in fruits. Even if nsLTPs from different Rosaceae fruits are highly homologous and cross-reactive, they possess different allergenic potentials, the strongest for Pru p 3 (peach), probably a primary sensitizer. This study aims at investigating the differences between Pru p 3 and Mal d 3 (apple), in relation to lipid-binding capacity and immunoreactivity, and to evaluate how LTP-ligand interaction may affect IgE reactivity.

**Methods**: Proteins were produced in *Pichia pastoris*, their expression was monitored in the culture supernatants by SDS-PAGE and immunoblotting with anti-nsLTP antiserum. rLTPs were purified by IEC chromatography and analysed by MALDI-TOF MS. To assess the LTP-ligand binding, ANS displacement assay was performed with 7 different fatty acids (from C12 to C18), either saturated or unsaturated, at different concentrations (10–100 μM). The IgE reactivity of recombinant allergens, alone or in complex with selected ligands, was analysed by ELISA assay using allergic patients’ sera.

**Results**: Both rLTPs migrate in SDS-PAGE between 10 and 15 kDa. MS analysis confirmed the identity of the purified proteins, providing 9.138 kDa (rPru p 3) and 9.553 kDa (rMal d 3) and were recognized by anti-nsLTP antiserum. ANS assay showed a higher fluorescence in Pru p 3 (+15%) compared to Mal d 3 and some differences in protein/ligands binding. However, both proteins had higher affinity for unsaturated fatty acids (e.g. ~50% fluorescence reduction with 10 μM oleic acid). ELISA test also evidenced higher IgE reactivity for rPru p 3 with respect to rMal d 3, as well as differences between the rLTPs alone and in complex with ligands. Our results suggest that Pru p 3 and Mal d 3 have different IgE reactivity and affinity for the fatty acids tested, but both preferentially bind unsaturated fatty acids. This interaction has an effect on IgE binding, but at different extent for Pru p 3 and Mal d 3. This may be due to specific differences in their lipid-binding region.

**Conclusion**: Our preliminary data support the hypothesis of a role of specific food matrix components (e.g. fatty acids), in modulating specific IgE responses to different food allergens with antimicrobial activity.

**Acknowledgements**: Supported by Marie-Curie project CARAMEL 626572, and FWF grants SFB-F4603 and W1248 to KHS, SP and PD, respectively.

### PD02 Apple, strawberry, hazelnut and tomato tolerance after one year of sublingual immunotherapy with LTP (Pru p 3) in patients with LTP-Syndrome

#### Gador Bogas^1^, Francisca Gomez^1^, Paloma Campo^1^, Maria Salas^1^, Inmaculada Doña^1^, Esther Barrionuevo^1^, Maria Auxiliadora Guerrero^1^, Cristobalina Mayorga^2^, Ana Prieto^1^, Domingo Barber^3^, Maria Jose Torres^1^

##### ^1^Allergy Unit, IBIMA-Regional University Hospital of Malaga, Malaga, Spain; ^2^Research Laboratory, IBIMA-Regional University Hospital of Malaga, Malaga, Spain; ^3^Institute for Applied Molecular Medicine (IMMA), School of Medicine, Universidad CEU San Pablo, Malaga, Spain

**Correspondence:** Francisca Gomez - paquigomez.p@hotmail.com

*Clinical and Translational Allergy* 2017, **7(Suppl 1)**:PD02

**Introduction**: One of the most frequent fruit and vegetable allergies in the Mediterranean area is non-specific-lipid transfer protein (nsLTP) syndrome where patients suffer allergies not only to peach but other plants-food related to nsLTPs. Specific immunotherapy (sIT) brings a new perspective to treat these patients however little is known whether sIT to one allergen can affect allergy to other plant-derived food. The aim was to evaluate the effect of sublingual immunotherapy (SLIT) with Pru p 3 (Pru p 3-SLIT) to other plants-derived-food in allergic patients to vegetable.

**Methods**: In a group of 36 patients with allergy to peach, 30 (83.3%) had allergies to other plants-food related. Plant-food allergies were evaluated by compatible clinical history, prick-prick to fresh fruit and ImmunoCAPIgE. After one year of treatment with (enriched-Pru p 3-SLIT) we evaluated reactivity to apple, hazelnut, strawberry and tomato by double blind placebo control food challenge (DBPCFC).

**Results**: In the total group of patients, 12 (33.3%) were allergic to apple, 4 (33.4%) had anaphylaxis, 5 (41.6%) urticaria and/or angioedema and three (25%) OAS. Four (11.1%) were allergic to hazelnut, from these 3 (75%) presented anaphylaxis and 1 (25%) urticaria and/or angioedema. Four (11.1%) to strawberry, all the patients presented OAS. Three (8.3%) were allergic to tomato, presenting anaphylaxis one patient (33.3%) and urticaria and/or angioedema 2 (66.75). Prick-prick with the culprit food was positive in all of the patients (100%). sIgE with apple, strawberry and tomato was positive in all the cases (100%). Hazelnut was positive in three (75%). After one year of SLIT all the patients tolerated tomato with peel and 100 gr of strawberries. Regarding reactivity to apple, 7 (58.3%) tolerated the whole apple with peel. Finally 2 patients (50%) tolerated 15 units of hazelnut. These results showed that a percentage of patients with clinical symptoms to other plants-food related to nsLTP that can tolerate this food after receiving enriched-Pru p 3-SLITduring one year. Enriched-Pru P 3-SLIT could be a good tool to improve the clinical symptoms in patients with LTP-Syndrome.

**Conclusion**: These data show clinical changes after the first year of treatment with enriched-Pru p 3-SLIT, not only to peach but also to other food allergens as apple, hazelnut, strawberry and tomato.

### PD03 Identification and implication of allergenic PR10 protein from walnut in birch pollen associated walnut allergy

#### Annette Jamin^1^, Andrea Wangorsch^1^, Jonas Lidholm^2^, Barbara Ballmer^3^, Stefan Vieths^1^, Stephan Scheurer^1^

##### ^1^Molecular Allergology, Paul-Ehrlich-Institut, Langen, Germany; ^2^Thermo Scientific, Uppsala, Sweden; ^3^Allergy Unit, Department of Dermatology, University Hospital Zürich, Zurich, Switzerland

**Correspondence**: Andrea Wangorsch - andrea.wangorsch@pei.de

*Clinical and Translational Allergy* 2017, **7(Suppl 1)**:PD03

**Introduction**: Beside hazelnut, the English walnut (*Juglans regia*) belongs to the most important allergenic tree nuts across Europe. So far, four walnut allergens (2S albumin, vicilin, nsLTP, 11S globulin) are listed in the official IUIS allergen database. Although an association of allergic reactions to walnut with birch pollen sensitization has been reported, no cross- reactive culprit walnut allergen has been described. The aim of the present study was to identify a Bet v 1-like protein in walnut and to investigate its allergenic properties.

**Methods**: Using a Bet v 1-homologous cDNA sequence from iron walnut leaves (KJ598787) as template, a cDNA encoding a corresponding protein from *Juglans regia* kernels (Jug r PR10) was cloned by RT-PCR and 5’RACE. Recombinant^®^ Jug r PR10 protein was expressed in *E. coli* and purified by a two-step chromatographic procedure. Purity and secondary structure were analyzed by SDS-PAGE and CD spectroscopy. Specific IgE levels to walnut extract, rBet v 1 and rJug r PR10 were measured by ImmunoCAP™ in birch pollen allergics with concomitant allergy to walnut (n = 15), confirmed by a positive open or double-blind placebo-controlled food challenge test. The presence of natural Jug r PR10 in walnut extract was analyzed by IgE immunoblot competition experiments using rJug r PR10 as inhibitor.

**Results**: Jug r PR10 (KX034087) was 100% identical in amino acid sequence to KJ598787, 67% to Bet v 1.01 and up to 74% to PR10 proteins from fruits, e.g. Mal d 1 (apple), Pru av 1 (cherry) and Pru p 1 (peach). Recombinant Jug r PR10 displayed secondary structures similar to those of Bet v 1. Walnut sensitization was detected in 40% (6/15) and 47% (7/15) of the patients studied, by ImmunoCAP and skin testing, respectively. In contrast, 93% (14/15) were reactive to rJug r PR10 and 100% to Bet v 1. The Bet v 1 and Jug r PR10 specific IgE values correlated strongly (r^2^ = 0.93), even though lower IgE levels were observed for Jug r PR10 (median 12.9 kU_A_/L) than for Bet v 1 (median 21.5 kU_A_/L). The presence of an IgE-reactive PR10 protein in walnut kernels was confirmed by immunoblot inhibition.

**Conclusion**: According to the established criteria, the PR10 protein from English walnut qualifies as major allergen. Low diagnostic sensitivity of walnut extract for patients with birch pollen associated walnut allergy might be due to small amounts of Jug r PR10 in walnuts. Recombinant Jug r PR10 may therefore become a useful tool for component-resolved diagnosis.

### PD04 Peptidomics of α-Gal carrying protein – Stability and allergenic properties

#### Danijela Apostolovic^1^, Jelena Mihailovic^2^, Maja Krstic^1,2^, Maria Starkhammar^3^, Tanja Cirkovic Velickovic^2^, Carl Hamsten^1^, Marianne van Hage^1^

##### ^1^Department of Medicine Solna, Immunology and Allergy Unit, Karolinska Institutet and Karolinska University Hospital, Stockholm, Sweden; ^2^Center of Excellence for Molecular Food Sciences, Faculty of Chemistry, University of Belgrade, Belgrade, Serbia; ^3^Department of Internal Medicine, Södersjukhuset, Stockholm, Sweden

**Correspondence**: Danijela Apostolovic - danijela.apostolovic@ki.se

*Clinical and Translational Allergy* 2017, **7(Suppl 1)**:PD04

**Introduction**: The mammalian carbohydrate galactose-α1,3-galactose (α-Gal) has shown to cause a novel form of food allergy, red meat allergy, where patients have severe allergic reactions several hours after red meat consumption. The number of diagnosed cases has increased significantly over the past few years and the α-Gal epitope is now an established clinically relevant glycan that should be taken into account in the diagnosis of food allergy. We have previously shown that red meat allergic patients have a selective IgE response to the pure α-Gal glycan that is unrelated to the carrier protein. The aim of this study was to explore the impact of digestion of α-Gal containing glycoproteins using a model system exposing the α-Gal containing protein bovine thyroglobulin to *in vitro* gastric digestion.

**Methods**: Bovine thyroglobulin was digested with pepsin. Digestion products were analyzed for stability and allergenic properties by SDS PAGE, immunoblot and ImmunoCAP using sera from ten red meat allergic patients, as well as with a peptidomics approach.

**Results**: During pepsinolysis of bovine thyroglobulin, a wide range of peptide bands could be observed during the first 10 min. Thereafter 14–17 kDa peptides remained stable during the whole gastric phase. The presence of the α-Gal epitope on the obtained peptides was demonstrated using an anti-α-Gal antibody as well as by IgE reactivity in sera from red meat allergic patients. The α-Gal peptides were able to inhibit at least 40% of the IgE-binding to bovine thyroglobulin (ImmunoCAP). The peptidomics approach showed that these peptides represent mostly internal and C-terminal parts of the protein, where a specific region from Arg_1617_ until Lys_2230_ contains complex type of glycans with the most potent IgE-binding α-Galactosyl residues. α-Gal containing food can elicit delayed severe allergic symptoms in red meat allergic patients. Here we show that peptides obtained after pepsinolysis of the model allergen, bovine thyroglobulin, contain α-Gal and remain stable during the whole gastric phase. Furthermore, these peptides contain specific α-Galactosyl recognition patterns and bind IgE from red meat-allergic patients.

**Conclusion**: The allergic response to α-Gal could depend on the type of α-Galactosyl residues on peptides obtained after gastric digestion, where complex type of glycans seems to have the most intense IgE-binding.

### PD05 Allergen-specific IgE and basophil responses to Ara h 2 and Ara h 6 are good predictors of peanut allergy in children

#### Francine C. van Erp^1^, Edward F. Knol^1^, Hannah M. Kansen^1^, Bo Pontoppidan^2^, Yolanda Meijer^1^, Cornelis K. van der Ent^1^, André C. Knulst^1^

##### ^1^University Medical Centre Utrecht, Utrecht, the Netherlands; ^2^Thermo Fisher Scientific, Uppsala, Sweden

**Correspondence**: Hannah M. Kansen - H.M.Kansen-2@umcutrecht.nl

*Clinical and Translational Allergy* 2017, **7(Suppl 1)**:PD05

**Introduction**: Double blind placebo-controlled food challenge (DBPCFC) is the gold standard to diagnose peanut allergy. In children sensitized to peanut, the detection of allergen-specific IgE (sIgE) and/or basophil sensitivity to Ara h 2 and Ara h 6 could be an alternative way to predict clinical peanut allergy and thereby avoid burdensome and expensive challenges in part of the patients.

We aimed to prospectively evaluate the most accurate diagnostic approach in children with suspected peanut allergy using sensitization tests and the Basophil Activation Test (BAT) to peanut components, with focus on Ara h 2 and Ara h 6.

**Methods**: In this cross sectional prospective diagnostic study (January 2012–May 2015), a total of 83 children (mean age 8.4 years) with suspected peanut allergy underwent diagnostic evaluation for peanut allergy including DBPCFC. The diagnostic value of sensitization tests and the BAT in predicting (severe) peanut allergy was evaluated.

**Results**: Peanut allergy was confirmed in 48 (58%) children, including 15 (18%) with severe allergy. Ara h 2 and h 6 showed high discriminatory capacity in sIgE and BAT. Ara h 6 had significant higher diagnostic value than Ara h 2 in the BAT. With sIgE to Ara h 2 we could classify 62% of children correctly as tolerant or allergic, when subsequently adding the BAT using Ara h 2 and Ara h 6 we could increase this to 80%.

**Conclusion**: This study shows that Ara h 2 and h 6 are both strong predictors of peanut allergy. A stepwise approach including sIgE to Ara h 2 and subsequently the BAT to Ara h 2 and Ara h 6 is able to predict peanut allergic status in the majority of children.

### PD06 How does thermal processing modulate the allergen profile and IgE reactivity of peanut?

#### Rebekah Sayers^1^, Helen Brown^2^, Adnan Custovic^1^, Angela Simpson^1^, Claire Mills^1^

##### ^1^University of Manchester, Manchester, United Kingdom; ^2^Campden BRI, Chipping Campden, United Kingdom

**Correspondence**: Rebekah Sayers - rebekah.sayers@postgrad.manchester.ac.uk

*Clinical and Translational Allergy* 2017, **7(Suppl 1)**:PD06

**Introduction**: Peanuts are rarely consumed in their native form and are most commonly, fried or roasted, conditions which favour the formation of Maillard reaction products (MRP’s). Quantitative proteomic profiling of these modified proteins will enable MRP formation to be monitored during processing and markers identified. Thermal processing is also thought to modulate the allergenic activity of peanuts by causing protein aggregation and decreasing solubility. The IgE reactivity of thermally processed peanut proteins will be assessed using a panel of peanut-allergic patient serum.

**Methods**: Raw and processed peanuts were extracted under harsh denaturing conditions and subjected to proteomic profiling using data-dependent acquisition (DDA) on an Orbitrap Elite mass spectrometer. Data was processed using Progenesis-QI and peptides identified using a curated peanut database and a predetermined set of variable Maillard modifications in Peaks. Serum samples from peanut allergic patients were obtained from the Manchester Respiratory, Allergy and Thoracic Surgery (ManARTS) Biobank and IgE reactivity assessed by immunoblotting, inhibition ELISA and histamine release.

**Results**: Mass spectrometric analysis revealed processing-induced modification of peanut allergens through the formation of Maillard reaction products and reduced solubility through aggregation. Extensive boiling (>2 h) had complex effects on allergen structure, and caused hydrolysis of allergens, and loss of Ara h 2 into the cooking water. Patients could be classified into those who were sensitised to several allergens and reacted towards aggregates, and those sensitised only to Ara h 2. Many of the latter patients were not reactive to boiled peanuts.

**Conclusion**: This work has identified a number of peptide markers in peanut which are characteristic of different types of thermal processing. It also highlighted the ability of extensively processed protein to retain IgE reactivity in certain sensitised individuals whilst boiled peanuts maybe less reactive in certain patients. Boiled peanuts may provide an alternative for oral immunotherapy with reduced side-effects, especially in patients only reactive to Ara h 2.

### PD07 Food induced anaphylaxis – Where did the food products come from and how much is consumed before reactions occur?

#### Sabine Dölle^1^, Linus Grabenhenrich^2^, Juliane Schulz^1^, Anne Moneret-Vautrin^3^, Margitta Worm^1^, Network for Online Registration of Anaphylaxis (NORA)

##### ^1^Department of Dermatology and Allergology, Comprehensive Allergy Center Charité, Charité - Universitätsmedizin Berlin, Berlin, Germany; ^2^Institute for Social Medicine, Epidemiology and Health Economics, Charité - Universitätsmedizin Berlin, Berlin, Germany; ^3^The Allergy Vigilance Network, University Hospital Nancy, Nancy, France

**Correspondence**: Sabine Dölle - sabine.doelle@charite.de

*Clinical and Translational Allergy* 2017, **7(Suppl 1)**:PD07

**Introduction**: Food is one of the most common elicitors of anaphylaxis. Even small amounts of a food allergen can cause severe allergic reactions. Based on data from the NORA network, we aimed to analyze the source and amount of food ingested causing the anaphylactic episode.

**Methods**: The European data from the Network for Online Registration of Anaphylaxis (NORA) was analyzed, restricted to cases of food-induced anaphylaxis.

**Results**: 2204 cases of food-induced anaphylaxis were registered between June 2011 and April 2016. The detailed questions for food induced anaphylaxis were answered in 1460 cases. Of these, 843 cases occurred to non-packed foods, and 617 cases to pre-packed foods. For 744 the source of food elicitor was unknown. The origin of non-packed products was known in 72%, mostly from supermarkets (n = 116), buffets (n = 108) and catered foods (n = 105). The origin was known in 78% for pre-packed foods namely cereal bar, peanut puffs or hazelnut spread. The responsible food allergen was listed in the list of ingredients in 89%. The amount of food causing the reaction was documented in 60% of all cases, more often when children were affected. In children, one tea spoon was the most frequently estimated amount, and a plate in adults.

**Conclusion**: Both, non- and pre-packed foods were frequent sources of allergens causing anaphylaxis. Despite, the allergen was explicitly stated in the list of ingredients in the majority of pre-packed foods, anaphylaxis occurred. Therefore, labelling alone is not sufficient to protect from severe reactions. Patients with food-induced anaphylaxis need detailed counselling about food allergen sources. Additionally, the differences in the amount of food allergen might not be age but rather depending on the major food allergen in this age group (children - peanut and adults - wheat).

**Acknowledgements**: Network for Online Registration of Anaphylaxis (NORA) participating centers can be found under www.anaphylaxie.net.

### PD08 New digestibility model(s) for investigating allergenicity of proteins

#### Jaap Akkerdaas^1^, Muriel Totis^2^, Annabelle Capt^2^, Corinne Herouet-Guicheney^2^, Ronald van Ree^1^

##### ^1^Academic Medical Center, Amsterdam, the Netherlands; ^2^Bayer Crop Science, Valbonne, France

**Correspondence**: Jaap Akkerdaas - j.h.akkerdaas@amc.uva.nl

*Clinical and Translational Allergy* 2017, **7(Suppl 1)**:PD08

**Introduction**: Gastric digestion assays have been part of the weight-of-evidence approach for evaluating the allergenic potential of proteins expressed in GM crops since protein stability in such assays was suggested to correlate with the allergenic status of proteins. EFSA has provided guidance that more physiologically relevant digestion assays should be evaluated for their potential to support the allergenicity risk assessment.

**Methods**: Nine proteins (shrimp and porcine tropomyosin, peanut and green pea albumin, peach and strawberry LTP, fish and bovine collagen, and carp parvalbumin) were subjected to 9 different gastric digestion conditions (pH 1.2/2.5/4.0, with three pepsin/protein ratios [PPR] 10:1/1:1/1:10), followed by duodenal digestion. Samples were taken at different time points and analyzed by SDS-PAGE and immunoblotting (mono-specific polyclonal rabbit antisera).

**Results**: The idea behind the four protein pairs was to pairwise compare an established strong allergen and a related non-allergen or weak allergen. Originally, we set out to do the same for fish parvalbumins, but purification of the hypothesized weak allergenic version from swordfish/tuna turned out to be extremely difficult due to the low content in the fish muscle (which is probably the more likely explanation of the low allergenicity of these fish). For three of the four pairs (tropomyosins, albumins and LTPs), the allergenic protein presented as the more stable during gastric digestion. The optimal conditions for this were pH1.2 and/or pH2.5 in combination with higher physiological PPRs (10 and/or 1). Gastric digestion at p 4.0 was clearly a less discriminative condition. Surprisingly, in case of the collagens, the allergenic one from fish was more labile than its bovine counterpart. Results from the duodenal digestion showed that, after gastric digestion at pH2.5 and/or 4.0 (less so after pH1.2), all proteins including all established allergens, were completely digested, also if they were resistant to preceding gastric digestion.

**Conclusions**: Gastric digestion at low (i.e. optimal) pH still remains the more appropriate but not perfect tool to use in building weight of evidence for the risk assessment of novel transgenes for GM crops. More physiological conditions, like higher gastric pH and/or inclusion of duodenal digestion may in fact be misleading with established strong allergens such as Ara h 2, Pru p 3 and Cyp c 1 being readily and completely digested.

### PD09 Increased parental anxiety and voluntary allergenic food avoidance (VAFA) in the siblings of the index case (IC) with single or multiple food allergies: exploring the parental response to the interventions for addressing their anxiety and successful introduction of new food

#### Tushar Banerjee, Antima Banerjee

##### Darlington Memorial Hospital, Darlington, United Kingdom

**Correspondence**: Tushar Banerjee - bnrjt07@hotmail.com

*Clinical and Translational Allergy* 2017, **7(Suppl 1)**:PD09

**Introduction**: It is known that parental anxiety & VAFA in siblings of an IC may affect the nutrition and quality of life (QOL). There is no evidence to support investigation and challenge for food allergy in the unaffected siblings. This study explores VAFA, parental anxiety and parental response to possible measures for facilitating new food introduction to the unaffected siblings of an IC.

**Methods**: The data was obtained from 40 families with proven food allergy in the IC attending a hospital in the UK. The parents were practicing VAFA in the unaffected siblings. The families with siblings suffering from eczema and asthma were excluded from the study. The families were offered 3 options:

1. Continue VAFA
2. Introduce suspected food in the hospital restaurant conveniently located near A&E and wait for 2 h
3. Give the suspected food at home during the day time.

**Results**: The parental anxiety score (AS) was measured in Likert Scale (0 to 10). Parents of IC with multiple food allergies and strong family history have higher AS to introduce suspected food in the siblings. Peanut, tree nut, milk and fish allergy caused maximum AS. 16 parents with AS >5 requested for allergy test before making the decision. All 16 parents choose to continue VAFA. Additionally, 5 parents in this group preferred VAFA to ensure allergen free household for safety of the IC & 4 witnessed anaphylaxis in the past (Table 1).

**Table 1:** See text for description

Options	Number of families	Anxiety score 5 to 10	Anxiety score 0 to 5
1. Continue VAFA	16	16	0
2. Introduce food in Hospital Restaurant	20	11	9
3. Introduce food at home	4	0	4

No allergic reaction was noted during food introduction. Parents reported reduction of AS post introduction. Parents felt less hesitant in introducing new food to the siblings in future.

**Conclusion**: This study highlights the effect of parental anxiety on VAFA. The novel innovative approach of introducing food within the hospital premises restaurant was perceived by parents as less risky and more reassuring. This option positively influenced parental decision to challenge and avoided unnecessary medicalisation of the problem in the unaffected siblings.

### PD10 Aggregation of ovalbumin and allergenicity

#### Mathilde Claude, Grégory Bouchaud, Roberta Lupi, Laure Castan, Olivier Tranquet, Sandra Denery-Papini, Marie Bodinier, Chantal Brossard

##### INRA UR 1268 Biopolymers Interactions Assemblies, Nantes, France

**Correspondence**: Mathilde Claude - mathilde.claude@nantes.inra.fr

*Clinical and Translational Allergy* 2017, **7(Suppl 1)**:PD10

**Introduction**: Allergen structure is often modified when heating foods. Aggregation is an irreversible modification of proteins with the formation of intermolecular bonds between unfolded proteins. Depending on the balance of attractive and repulsive interactions during heating, aggregates of various morphologies may be generated. This study investigates how different way of aggregating ovalbumin modulates its allergenicity by comparing two morphologies of aggregates obtained under opposite electrostatic conditions.

**Methods**: An ovalbumin solution was extensively heated (80°C for 6 h) under opposite electrostatic conditions to form small linear and large spherical-agglomerated aggregates. In a murine model of allergy, we compared the Ig production when sensitizing mice with the aggregates and the subsequent elicitation phase upon an oral challenge with native ovalbumin. The reactivity of specific IgE in mice sera was characterized by ELISA, Rat Basophil Leukemia assay and pepscan analysis.

**Results**: IgE production was significantly lower for the small aggregates than for the large aggregates, whereas IgG_1_ and IgG_2a_ productions didn’t change. In agreement with the IgE production, both symptoms upon oral challenge and basophil degranulation with native ovalbumin were reduced for mice sensitized with small compared to large aggregates. Pepscan analysis revealed two common linear IgE-epitopes but the aggregates were similarly or differently bound and cross-linked depending on the aggregate that had been used during sensitization. These results showed that small aggregates of ovalbumin formed under repulsive electrostatic conditions displayed a lower allergenic potential than the large aggregates. The way ovalbumin aggregated also modified the IgE repertory.

**Conclusion**: This work illustrates links between food structure and allergenic potential on parameters from the sensitization phase with some consequences on the elicitation phase of the allergic reaction. For the first time, we show that the physicochemical conditions when heating ovalbumin and consequently the aggregated structure are important parameters to consider in the context of allergy.

### PD11 Residual determination of milk and egg allergens in bakery products by LC-MS/MS

#### Rosella De Poi^1,2^, Elisa Gritti^1^, Emiliano De Dominicis^1^, Bert Popping^1^, Patrizia Polverino de Laureto^2^

##### ^1^R&D Department Italy Mérieux NutriSciences, Resana, Italy; ^2^Department of Pharmaceutical Sciences, Università di Padova, Padova, Italy

**Correspondence**: Rosella De Poi - rossella.depoi@studenti.unipd.it

*Clinical and Translational Allergy* 2017, **7(Suppl 1)**:PD11

**Introduction**: Food allergy is causing adverse health effects arising from specific immune-mediated responses, occurring reproducibly upon oral exposure to a given food. In the absence of a cure, sufferers have to rely on the accurate labeling of food to avoid allergens. Egg and cow’s milk proteins are common triggers of allergic reactions, especially in children. Following a multimethod comparative study about the latest approaches in food analysis using state-of-the-art technology (see our previous work), one specific goal of this study is to develop and validate a LC-MS/MS method for residual determination of cow’s milk and egg allergens in bakery products.

**Methods**: After sample homogenization, proteins are extracted, denatured and reduced by TCEP. Free thiol moieties are then alkylated and proteins digested by trypsin. The peptide mixtures obtained are then purified by Solid Phase Extraction and analyzed by LC-MS/MS using Sciex Q-Trap 6500 mass spectrometer. For our purpose, a Multiple Reaction Monitoring (MRM) method specific for milk β-lactoglobulin (β-lg) and egg ovalbumin was set up.

**Fig. 1:**
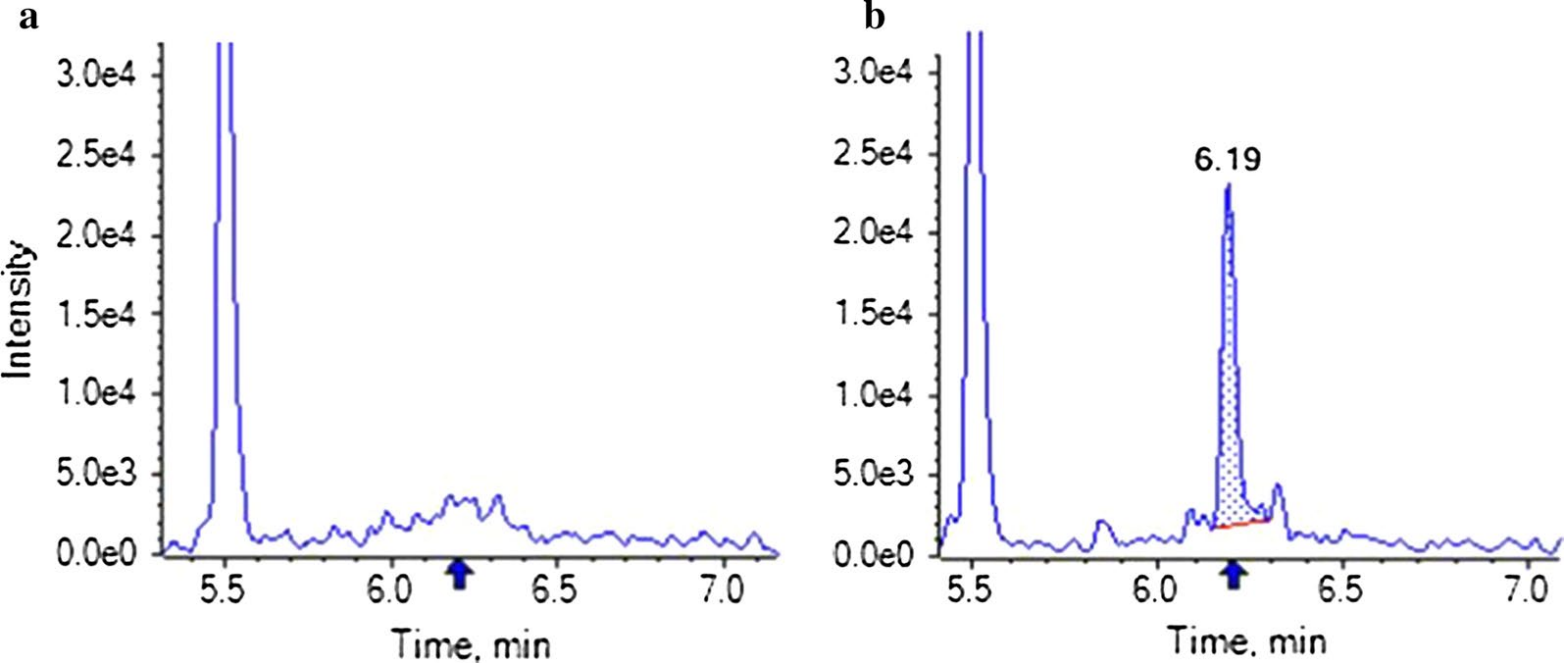
See text for description

**Results**: After spiking a mix of β-lg and ovalbumin into different bakery products, specific MRM signals were detected for both proteins. As an example, two XIC graphs for one of the MRM transitions investigated in β-lg are reported below (Fig. 1). The signal is clearly visible in the product spiked with as little as 0,05 mg/kg of β-lg (B), but is absent in the blank (A).

The efficacy of the MRM method was assessed by testing a certified material (cake mix, FAPAS) for milk’s presence. The material was previously used for a ring test involving different ELISA kits. As ELISA kits suffer from high kit-to-kit variability (as can be seen in many proficiency testing results) a quantitative comparison with the results obtained from ELISAs was not possible. In LC-MS/MS analysis, quantification by MRM is generally more accurate as it is not reliant upon biological interactions like antibodies.

**Conclusion**: The LC-MS/MS approach, combined with an efficient extraction method, shows high sensitivity and selectivity under all evaluated conditions, without producing false negatives for neither egg nor milk. The developed method provides a valid alternative to the ELISA kits on the market, bypassing issues associated with antigen-antibody interactions and retaining a low limit of quantification. The method will be validated and considered for accreditation.

### PD12 Predictive value of ovomucoid-specific IgE in the diagnosis of egg allergy in Finnish children

#### Kati Palosuo, Anna Kaarina Kukkonen, Anna Pelkonen, Mika Mäkelä

##### Helsinki University Hospital, Skin and Allergy Hospital, University of Helsinki, Helsinki, Finland

**Correspondence**: Kati Palosuo - kati.palosuo@fimnet.fi

*Clinical and Translational Allergy* 2017, **7(Suppl 1)**:PD12

**Introduction**: To calculate optimal cut-off values for egg white and Gal d 1,2,3, and 4 -specific IgE (sIgE) predicting positive oral challenges in 100 Finnish children with suspected egg allergy.

**Methods**: 100 patients (age 1–19 years, mean 9.2, median 9.6 years) with suspected egg allergy underwent double-blind, placebo-controlled (n = 60) or open (n = 40) food challenges with heated egg white. Serum IgE levels to egg white as well as the components Gal d 1 (ovomucoid), Gal d 2 (ovalbumin), Gal d 3 (conalbumin) and Gal d 4 (lysozyme) were measured by ImmunoCAP.

**Fig. 2:**
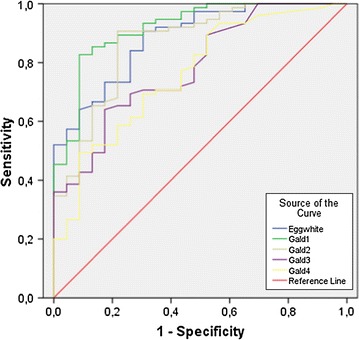
ROC curve. Diagonal segments are produced by ties

**Results**: Of the 100 challenges 75 were positive. Sensitization to Gal d 1 (ovomucoid) with a cut-off of value of >4.3 kU/L predicted a positive challenge with a specificity of 92% and sensitivity of 83%. The likelihood ratio was 10.3 In ROC analysis the area under curve was 0.92 (95% CI 0.86–0.98). Gal d 1 sIgE levels were significantly higher in the challenge positive (mean 38 kU/L, median 15.8 kU/L) than in the challenge negative group (mean 2.4 kU/L, median 0.5 kU/L), p < 0.0001. The diagnostic capacity of sIgE to egg white and Gal d 2, 3 and 4 was clearly weaker. In ROC analysis the AUC for egg white was 0.88, Gal d 2 0.87, Gal d 3 0.78 and Gal d 4 0.76 (Fig. 2).

Component-specific IgE levels can be useful in predicting outcomes of oral food challenges, but optimal cut-off levels vary in different populations. Sensitization is influenced by many factors including age and geography, whereas challenge outcome is influenced by referral base and challenge procedures. Thus, optimal cut-off levels need to be determined for each population separately. Sensitization to Gal d 1, which is relatively stable against heat and enzymatic digestion, predicts clinical reactivity to both raw and heated egg and is considered a risk factor for persistent egg allergy.

**Conclusion**: Ovomucoid-specific IgE is useful in distinguishing egg-sensitized patients with clinically reactive egg allergy from those tolerant to heated egg. The optimal cut-off point in a Finnish population of 100 children and adolescents was 4.3 kU/L.

### PD13 7S and 11S globulins are likely to be allergens in macadamia nuts

#### Nanju Alice Lee^1^, Johanna Rost^1^, Sridevi Muralidharan^1^, Dianne Campbell^2^, Sam Mehr^2^, Catherine Nock^3^, Joseph Baumert^4^, Steve Taylor^4^

##### ^1^ARC Training Centre for Advanced Technology in Food Manufacture (ATFM), University of New South Wales, Sydney, Australia; ^2^Children’s Hospital Westmead, Sydney, Australia; ^3^Southern Cross University, East Lismore, Australia; ^4^FARRP, University of Nebraska, Lincoln NE, USA

**Correspondence**: Nanju Alice Lee - alice.lee@unsw.edu.au

*Clinical and Translational Allergy* 2017, **7(Suppl 1)**:PD13

**Introduction**: Macadamia nut is reported to cause allergic reactions in sensitized individuals, and the prevalence is expected to increase along with the increase in world production and popularity of macadamia nuts as an ingredient in pre-packaged foods. Despite several reports documenting macadamia nut allergies, the eliciting allergens have never been identified due to a lack of genomic and proteomic data on macadamia nut. Identification and characterization of allergenic proteins are crucial for developing effective component-resolved diagnosis and management/treatment strategies.

We aimed to identify putative allergenic proteins in macadamia nut by combining patient IgE recognition with an allergenomics approach.

**Methods**: Five serum samples were collected from patients with clinical allergy. Immunoreactive proteins were identified by immunoblotting with the patient sera. A label-free shotgun proteomics approach was used to investigate the proteomic profile of macadamia nut. Briefly, the molecular weight distribution of proteins was determined by 1-dimentional and 2-dimensional gel electrophoresis. Following in-gel digestion with trypsin, proteins were subjected to liquid chromatography coupled tandem mass spectrometry.

**Results**: The label-free shotgun proteomics was used to predict putative macadamia nut allergens for the first time. The amino acid sequence homologies to 21 known allergens across different plant species were identified, which include lupin, latex, peanut, soy and rice allergens. The immunoblotting of the soluble proteins with the serum IgE revealed five reactive protein bands. The mass spectrometry analysis of the IgE-reactive proteins showed matches to Miamp1 protein, Miamp2, vicilin-like protein, 11S-legumin like protein and lactoylglutathione lyase amongst other proteins.

**Conclusion:**: The seed storage proteins belonging to 11S and 7S proteins are likely to be biologically active allergens in macadamia nuts.

### PD14 Pollen-food syndrome among Italian children: molecular endotypes

#### Carla Mastrorilli^1,2,3^, Salvatore Tripodi^3,4^, Carlo Caffarelli^1,3^, Serena Perna^2^, Andrea Di Rienzo Businco^4^, Ifigenia Sfika^4^, Riccardo Asero^5^, Arianna Dondi^6^, Annamaria Bianchi^7^, Carlotta Povesi Dascola^1^, Giampaolo Ricci^8^, Francesca Cipriani^8^, Nunzia Maiello^9^, Michele Miraglia del Giudice^9^, Tullio Frediani^10^, Simone Frediani^10^, Francesco Macrì^10^, Chiara Pistoletti^10^, Iride Dello Iacono^11^, Maria Francesca Patria^12^, Elena Varin^13^, Diego Peroni^14^, Pasquale Comberiati^14^, Loredana Chini^15^, Viviana Moschese^15^, Sandra Lucarelli^10^, Roberto Bernardini^16^, Giuseppe Pingitore^17^, Umberto Pelosi^18^, Roberta Olcese^19^, Matteo Moretti^10^, Anastasia Cirisano^20^, Diego Faggian^21^, Alessandro Travaglini^15^, Mario Plebani^21^, Maria Carmen Verga^11,22^, Mauro Calvani^23^, Paolo Giordani^24^, Paolo Maria Matricardi^2,3^

##### ^1^Pediatric Department, Department of Clinical and Experimental Medicine, Azienda Ospedaliera-Universitaria, University of Parma, Parma, Italy; ^2^Department of Pediatric Pneumology and Immunology, Charité Medical University Berlin, Berlin, Germany; ^3^The Italian Pediatric Allergy Network (I-PAN); ^4^Pediatric Department and Pediatric Allergology Unit, Sandro Pertini Hospital, Rome, Italy; ^5^Allergology Service, San Carlo Clinic, Paderno Dugnano, Milan, Italy; ^6^Pediatric Unit, Department for Mother and Child, Ramazzini Hospital, Carpi, Italy; ^7^Pediatric Unit, Mazzoni Hospital, Ascoli Piceno, Italy; ^8^Pediatric Unit, Department of Medical and Surgical Sciences, University of Bologna, Bologna, Italy; ^9^Pediatric Department, Second University, Naples, Italy; ^10^Pediatric Department, La Sapienza University, Rome, Italy; ^11^Pediatric Unit, Fatebenefratelli Hospital, Benevento, Italy; ^12^Pediatric Highly Intensive Care Unit, Department of Pathophysiology and Transplantation, Università degli Studi di Milano, Fondazione IRCCS Ca’ Granda Ospedale Maggiore Policlinico, Milan, Italy; ^13^Pediatric Intermediate Care Unit, Fondazione IRCCS Ca’ Granda Ospedale Maggiore Policlinico, Milan, Italy; ^14^Pediatric Section, Department of Life and Reproduction Sciences, University of Verona, Verona, Italy; ^15^Pediatric Department, Policlinico of Tor Vergata, Tor Vergata University, Rome, Italy; ^16^Pediatric Unit, San Giuseppe Hospital, Empoli, Italy; ^17^Pediatric Unit, Grassi Hospital, Rome, Italy; ^18^Pediatric Unit, Santa Barbara Hospital, Iglesias, Italy; ^19^Pulmonary Disease and Allergy Unit, G. Gaslini Hospital, Genoa, Italy; ^20^Pediatric Unit, Crotone, Italy; ^21^Department of Laboratory Medicine, University of Padua, Padua, Italy; ^22^ASL Salerno, Salerno, Italy; ^23^UOC Pediatria, San Camillo Forlanini, Rome, Italy; ^24^Department of Statistical Sciences, Sapienza University of Rome, Rome, Italy

**Correspondence**: Carla Mastrorilli - carla.mastrorilli@icloud.com

*Clinical and Translational Allergy* 2017, **7(Suppl 1)**:PD14

**Introduction**: Pollen food syndrome (PFS) is heterogeneous with regard to triggers, severity, natural history, comorbidities and response to treatment. Our study aims to classify different endotypes of PFS based on IgE sensitization to panallergens.

**Methods**: We examined 1271 Italian children (age 4–18 years) with seasonal allergic rhinoconjunctivitis (SAR). Foods triggering PFS were acquired by questionnaire. Skin prick tests were performed with commercial pollen extracts. IgE to panallergens: Phl p 12 (profilin), Bet v 1 (PR-10) and Pru p 3 (nsLTP), were tested by ImmunoCAP FEIA. An unsupervised hierarchical agglomerative clustering method was applied within PFS population.

**Results**: PFS was observed in 300/1271 children (24%). Cluster analysis identified five PFS endotypes linked to panallergen IgE sensitization: (1) Co-sensitization to ≥2 panallergens (“multi-panallergen PFS”); (2–4) sensitization to either profilin, or nsLTP, or PR-10 (“mono-panallergen PFS”); (5) no sensitization to panallergens (“no-panallergen PFS”). These endotypes showed peculiar characteristics: (1) “multi-panallergen PFS”: severe disease with frequent allergic comorbidities and multiple offending foods; (2) “Profilin PFS”: OAS triggered by *Cucurbitaceae*; (3) “LTP PFS”: living in Southern Italy, OAS triggered by hazelnut and peanut; (4) “PR-10 PFS”: OAS triggered by *Rosaceae*; (5) “no-panallergen” PFS: mild disease and OAS triggered by kiwifruit.

**Conclusions**: In a Mediterranean country characterized by multiple pollen exposures, PFS is a complex and frequent complication of childhood SAR, with five distinct endotypes marked by peculiar profiles of IgE sensitization to panallergens. Prospective studies in cohorts of PFS patients are now required to test whether this novel classification may be useful for diagnostic and therapeutic purposes in the clinical practice.

### PD15 Thermal treatment of bovine caseins could increase their sensitising potential

#### Noe Ontiveros, Francisco Cabrera-Chavez

##### University of Sinaloa, Culiacan, Mexico

**Correspondence**: Francisco Cabrera-Chavez - fcabrera@uas.edu.mx

*Clinical and Translational Allergy* 2017, **7(Suppl 1)**:PD15

**Introduction**: To evaluate the digestibility as well as the immunogenic and sensitizing potential of thermally treated bovine caseins (TT caseins).

**Methods**: Caseins were dry heated for 30 or 60 min at 140°C. Native and TT caseins were digested in simulated gastric fluid pH 1.2 (10 U of pepsin activity/µg of test protein). Samples of 200 µl were removed after incubation at 37°C. The reaction was quenched by addition of 70 µl of 200 mM NaHCO_3_, pH 11, and 70 µl 5× Laemmli buffer. The zero time points were prepared by quenching the pepsin in the solution before adding the test protein. The samples were subjected to SDS-PAGE electrophoresis using 10–20% polyacrylamide Tris-glycine gels. BALB/c mice (n = 6/group) were sensitized intraperitoneally without adjuvants through the administration of native or TT caseins in 250 µl of PBS. Sensitizations were performed at days 0, 3, 6, 9, 12, and blood was drawn on day 28. Casein-specific IgG and IgE antibodies were evaluated using ELISA.

**Results**: There was no effect of incubation of caseins in simulated gastric fluid for 60 min in the absence of pepsin. However, caseins, either native or TT caseins, were very unstable in the presence of pepsin, with a marked loss of full length protein after 0.5 min of incubation. Native and TT caseins were both immunogenic and allergenic at the dose tested (0.05 mg/mouse). Casein-specific IgG and IgE titers were higher in the group of mice sensitized with TT caseins for 30 min than the other groups (p < 0.05). There were no differences between the groups sensitized with native caseins and TT caseins for 60 min (p > 0.05). Specific IgE antibodies in sera from mice sensitized with native casein strongly recognized TT caseins and *vice versa* (p > 0.05). Extensively heated cow’s milk is an alternative to accelerate tolerance acquisition in some milk allergic cases. This is in line with our digestibility results, an indirect parameter of allergenicity, as TT caseins remains as digestible as native caseins. However, there was no reduction of the sensitizing potential of TT caseins. Although this potential was similar between TT caseins for 60 min and native caseins, longer thermal treatments are not suitable for the study because of the loss of protein solubility.

**Conclusion**: Thermal treatment of caseins alone does not reduce their allergenic potential highlighting that heat-induced interactions between milk proteins and other milk components are required to reduce their allergenic potential.

### PD16 The gaps in anaphylaxis diagnosis and management by French physicians

#### Guillaume Pouessel^1,2^, Julie Galand^1,2^, Julien Labreuche^3^, Etienne Beaudouin^4^, Jean-Marie Renaudin^4^, Anne Moneret-Vautrin^4^, Antoine Deschildre^2^, the Anaphylaxis Working Group of the French Allergology Society

##### ^1^Department of Pediatrics, Children’s Hospital, Roubaix, France; ^2^Division of Pulmonology and Allergology, Department of Pediatrics, Faculty of Medicine and Children’s Hospital, Lille, France; ^3^Biostatistics Unit, Maison Régionale de la Recherche Clinique, CHRU Lille, Lille, France; ^4^Department of Allergology, Emile Durkheim Hospital, Epinal, France

**Correspondence**: Guillaume Pouessel - guillaume.pouessel@gmail.com

*Clinical and Translational Allergy* 2017, **7(Suppl 1)**:PD16

**Introduction**: Anaphylaxis is still under-recognized and the treatment is often inadequate with underutilization of adrenaline even by medical personnel.

Our aim was to assess physician’s knowledge regarding diagnosis and management of anaphylaxis in children and to identify the reasons for the gaps.

**Methods**: Physicians were asked to respond a two-part questionnaire during continuing medical education: 1. A clinical scenario-based questionnaire involving a child experiencing a food-induced anaphylaxis with 5 of 9 true/false questions considered as key questions for an optimal management; 2. Demographic data and questions exploring determinants of an appropriate management.

**Results**: 318 physicians (GPs, 28%; pediatricians, 23%; allergists, 10%; school-mother and child care doctors, 19%; and junior doctors, 20%) were enrolled. They had private (29%), hospital (31%), or both practice (7%) or others (33%).

*Part 1:* 70% of participants agreed that the scenario was consistent with anaphylaxis but 24% refused because hemodynamic or respiratory disorders were missing; 31% chose to administer first adrenaline intramuscularly and 30% agreed with both diagnosis and treatment with adrenaline; 74% chose to administer first antihistamine and bronchodilators. Only 50% chose to call immediately the emergency number. Nearly a third estimated that a one-hour observation period was enough. Only 19% had all 5 key responses correct.

*Part 2:* A correct diagnosis was associated with pediatric specialty (p < 10^−4^) and hospital practice (p = 0.02); the use of adrenaline in the scenario with a correct diagnosis (p < 10^−4^), pediatric specialty (p < 10^−4^), a recent continuing medical education on food allergy (p = 0.005) and experience of adrenaline injection in real life (p = 0.02); all the 5 key responses correct with pediatric specialty (p < 10^−4^) and a recent continuing medical education (p = 0.04). In case of anaphylaxis in a child, 59% of physicians would immediately inject intramuscularly adrenaline, 22% only after calling an emergency physician, 18% only in case of vital disorders, 3% only in the presence of an emergency physician; 5% would refuse to inject adrenaline themselves (never done or feared side effects).

**Conclusion**: A large proportion of doctors seem to be unaware of the diagnosis criteria and the recent updated EAACI recommendations on anaphylaxis management. Medical specialty and continuing medical education improve anaphylaxis management.

### PD18 Relationship between deamidation intensity and allergenicity of acid hydrolysed wheat proteins preparations: from France to Japan

#### Olivier Tranquet^1^, Florence Pineau^1^, Roberta Lupi^1^, Shinobu Sakai^2^, Kayoko Matsunaga^2^, Reiko Teshima^2^, Colette Larré^1^, Sandra Denery^1^

##### ^1^UR 1268 Biopolymères, Interactions, Assemblages, INRA, Nantes, France; ^2^National Institute of Health Sciences, Tokyo, Japan

**Correspondence**: Olivier Tranquet - olivier.tranquet@nantes.inra.fr

*Clinical and Translational Allergy* 2017, **7(Suppl 1)**:PD18

**Introduction**: Hydrolyzed wheat proteins (HWP) were used as ingredients in food and cosmetics. From the 2000’s severe food allergy to HWP has been reported in individuals elsewhere tolerant to native wheat proteins. Denery et al. demonstrated that deamidation of wheat proteins, a consequence of acid hydrolysis, generate essential neo-epitopes in these particular allergy to wheat [1]. More recently in Japan, an acid-HWP preparation (a-HWP), named GluPearl 19S, elicited severe skin reactions and food allergy in more than 1800 individuals and was likely to contain deamidated gluten proteins [2]. Level of deamidation depends on treatment intensity; a-HWP preparations with either low or high level of deamidation can be found as ingredient [3]. This study aimed at exploring the impact of deamidation level of wheat proteins on the degranulation of basophils sensitized with IgE from patient allergic to a-HWP.

**Methods**: Impact of the deamidation level of gliadins and a-HWPs upon IgE reactivity of 8 a-HWP allergic patients was determined by ELISA. Impact of deamidation on basophil degranulation was also explored with humanized Rat Basophil Leukemia cells passively sensitized with IgE from patients and subjected to crosslinking with a set of deamidated samples. Finally IgE Repertoire specific to deamidated wheat protein was then explored by inhibition with INRA-DG1, a mouse monoclonal antibody specific for deamidated gliadins.

**Results**: Intensity of binding of patient IgE onto a-HWP and the degranulation potency were correlated with level of deamidation. Pre-incubation of deamidated gluten with INRA-DG1 mAb inhibited half of its degranulation capacity with patient IgE. These results suggested that the patient IgE repertoire specific for deamidated gluten proteins is likely to be limited to a very few specificities. GluPearl 19S, involved in the Japanese cases, was determined as highly deamidated. It was the most recognized sample among the 5 deamidated glutens tested in this study.

**Conclusion**: Although differences exist between French and Japanese cases (such as the tolerance of native wheat proteins), this result suggested that Japanese and French cases suffered from the same unconventional allergy to wheat.

**References**

1. Denery-Papini S, et al. Allergy 2012;67;1023–32.
2. Nakamura R, et al. Int Arch Allergy Immunol. 2013;160, 259–64.
3. Tranquet O, et al. J Agric Food Chem. 2015;63:5403–9.

### PD19 Hen’s egg allergen in house and bed dust is significantly increased after hen’s egg consumption

#### Sebastian Tschirner, Valérie Trendelenburg, Gabriele Schulz, Bodo Niggemann, Kirsten Beyer

##### Department of Pediatric Pneumonology and Immunology, Charité Universitätsmedizin Berlin, Berlin, Germany

**Correspondence**: Sebastian Tschirner - sebastian.tschirner@charite.de

*Clinical and Translational Allergy* 2017, **7(Suppl 1)**:PD19

**Introduction**: Cutaneous exposure to food allergens seems to be an important way of sensitization. It has been shown that household consumption of peanut is a risk factor for the development of peanut allergy. Furthermore, peanut protein was found in house dust. Yet there is only little data on other food allergens; therefore, we wanted to investigate whether hen’s egg (HE) protein could be found in domestic areas.

**Methods**: 8 households were included in the study. All households were asked to consume a meal of scrambled eggs in their habitual eating areas. Before and 48 h after hen’s egg consumption dust samples were collected in the habitual eating areas and from bed sheets, using a special vacuum cleaner device. HE protein was extracted and HE allergen levels were measured with a commercially available ELISA (limit of detection: 0.05 µg/g). Wilcoxon rank test was used to compare HE levels before and after HE consumption.

**Results**: HE protein was detectable in all 8 households in the habitual eating areas as well as on bed sheets. At baseline, there was a median of 13.05 µg/g HE protein (range 6.5–13.4 µg/g) in the habitual eating area and a median of 12.9 µg/g HE protein (range 2.0–18.4 µg/g) on the bed sheets. 48 h after consumption of scrambled egg significantly increased levels were measured with a median of 214.0 µg/g HE protein (range 17.0–2409.4 µg/g; p < 0.05) for the eating area and a median of 50.3 µg/g HE protein (range 5.9–247.0 µg/g; p < 0.05) in bed sheets. HE allergens were detectable in the house dust of all households not only in dining areas but also in the bed where HE is usually not consumed, indicating a spreading of food allergens. Furthermore, an increase in protein levels measured after consumption of a HE meal was shown. For infants, who spent most time in bed, house dust containing food allergens could be an important risk factor for food sensitization.

**Conclusion**: HE allergen was found in house and bed dust with high levels following HE consumption, indicating a potential risk factor for the development of HE sensitization. Nevertheless, further research is necessary to proof whether HE allergens in house and bed dust can cause sensitization and whether there is a correlation between allergen levels and the level of sensitization.

### PD20 Native gliadins stimulate the local anaphylactic response in Ussing chamber as studied in murine model of wheat allergy

#### Youcef Bouferkas, Younes Belabbas, Djamel Saidi, Omar Kheroua, Kamel Eddine El Mecherfi

##### Laboratory of Physiology of Nutrition and Food Safety, Department of Biology, Faculty of Natural and Life Sciences, University of Oran 1 Ahmed Ben Bella, Oran, Algeria

**Correspondence**: Youcef Bouferkas - youcef35@gmail.com

*Clinical and Translational Allergy* 2017, **7(Suppl 1)**:PD20

**Introduction**: The objective of this study is to investigate the impact of the native gliadins on the intestinal electrophysiological parameters as studied in murine allergy model.

**Methods**: Two groups of mice (n = 10 per group) were established: the first group (control) was sensitized with aluminum hydroxide (Alum) diluted in PBS, the second was sensitized with 10 µg of native gliadins adsorbed on alum (NG). Intraperitoneal (IP) sensitizations were performed at days 0, 10, 20 and 30. One week after the last boost, the jejunum fragments were withdrawn and used for histological analysis and the evaluation of the local anaphylactic responses in Ussing chamber by an *ex vivo* challenge allowing the contact of jejunums with sensitizing antigen and the measurement of electrophysiological parameters (short-circuit current (Isc) (µA/cm^2^) and conductance (G) (mmho/cm^2^).

**Results**: Intraperitoneal injection of native gliadins induced significant increase of the short circuit current (Isc µA/cm^2^) (*P* < 0.001) and conductance (G) values (*P* < 0.05). The histological observations of jejunum fragments of sensitized mice show an alteration in intestinal barrier (villous atrophy, Lymphocytes infiltration) compared to the control group. The Isc increase in sensitized mice is probably due to a secretory response and might well reflect local anaphylactic responses. The increase of conductance indicate that immunization alters the tight junctions and increases the paracellular permeability of the intestinal epithelium [1]. Several published studies show that food allergy in mice is characterized by villous atrophy and goblet cell hyperplasia, as well as infiltration of IgE-positive mast cells [2].

**Conclusion**: Our results suggest that the native gliadins modify permeability of the intestinal tract in native gliadin mice which confirm the allergenic potential of native gliadins

**References**

1. Terpend K, Blaton MA, Candalh C, et al. Intestinal barrier function and cow’s milk sensitization in guinea pigs fed milk or fermented milk. J Pedia Gastroenterol Nutr. 1999;28:191–8.
2. Grar H, Dib W, El Mecherfi K E, et al. Supplementation with β-carotene or vitamin E protects against increase in anaphylactic response in β-lactoglobulin-sensitized Balb/c mice: Ex vivo study. European Food Research & Technology 2015;241:393–8.

### PD21 Oral administration of royal jelly at different dose protect against increase in anaphylactic response in β-lactoglobulin sensitized Balb/c mice

#### Malika Guendouz, Abir Haddi, Omar Kheroua, Djamel Saidi, Hanane Kaddouri

##### LPNSA, Biology, Faculty of Natural and Life Sciences, University of Oran 1 Ahmed Ben Bella, Oran, Algeria

**Correspondence**: Malika Guendouz - guendouzmalika@hotmail.fr

*Clinical and Translational Allergy* 2017, **7(Suppl 1)**:PD21

**Introduction**: The objective of this work is to check if royal jelly could prevent the allergic response and reduce the clinical manifestations of mice to bovine milk proteins.

**Methods**: Forty female Balb/c mice at 4 weeks and fed with a standard diet were divided into 5 groups. Four groups received orally royal jelly for 7 days at doses of 0 g/kg (positive control), 0.5, and 1.5 g/kg and are then sensitized intraperitoneally with (*β*-lactoglobulin) *β*-Lg adsorbed on Alum. Group 5 received no treatment (negative control). At the end of the experiment, in vivo provocation test (observation of clinical signs 30 min after intraperitoneal injection: 1 mg *β*-Lg/mouse) and ex vivo provocation test in Ussing chamber by contacting the jejunum with the sensitizing antigen and measurement of electrophysiological parameters: short-circuit current (Isc, μA/cm^2^) were carried out.

**Results**: Our results show that the majority of mice pretreated with royal jelly dose showed no clinical signs after challenge compared with positive control mice (CL+). A secretory response reflecting local intestinal anaphylaxis was evident in sensitized mice, as indicated by an increase in Isc (p < 0.001). However, no significant changes in the values of the short circuit current (Isc) were observed after *β*-Lg challenge in mice that received royal jelly at 0.5, 1 and 1.5 g/kg and immunized with *β*-Lg. Oral administration of royal jelly at different dose prevents the appearance of clinical signs and the development of anaphylactic local. This effect is probably due to the presence of compound having immunomodulatory activity which may reduce T-cell proliferation, Th2 cytokine production, histamine release from mast cells and antibody IgE and IgG production (Vucevic et al. 2007).

**Conclusion**:

Oral administration of royal jelly decreases the allergic and inflammatory response in mice and can be considered as a preventive strategy against Cow‘s milk allergy (CMA).

**Reference**

1. Vucevic D, Melliou E, Vasilijic S, Gasic S, Ivanovski P, Chinou I, Colic M (2007) Fatty acids isolated from royal jelly modulate dendritic cell-mediated immune response in vitro. Int Immunopharmacol. 7:1211–20.

## POSTER DISCUSSION SESSION 2: Management of food allergy • Diagnosis and treatment • Epidemiology • Therapeutic options

### PD22 Sting reactions in beekeepers: characteristics and management

#### Luis Amaral^1^, Ana Pereira^2^, Alice Coimbra^1^

##### ^1^Serviço de Imunoalergologia, Centro Hospitalar de São João E.P.E., Porto, Portugal; ^2^CINTESIS, Faculdade de Medicina da Universidade do Porto, Porto, Portugal

**Correspondence**: Luis Amaral - luis.m.amaral@gmail.com

*Clinical and Translational Allergy* 2017, **7(Suppl 1)**:PD22

**Introduction**: Our objective was to describe what Portuguese beekeepers do after being stung. The secondary aim was to evaluate their knowledge on adrenaline auto-injectors (AAI), bee venom immunotherapy (VIT), and the medical specialty of Immunoallergology.

**Methods**: Cross-sectional study using a structured questionnaire including beekeepers present in an apiculture meeting. Data on demographic characteristics, number of stings, reaction description and conduct when stung (treatment, admission to ER) were collected. Awareness of AAI, VIT and the medical specialty of Immunoallergology was also questioned.

**Results**: A total of 114 beekeepers were included; 91 (80%) male with a median (interquartile range, IQR) age of 40 (17) years. The median (IQR) time of beekeeping was 3 (7.3) years. Nighty-nine (87%) were amateur beekeepers. All had been stung in the last 12 months; 45 (40%) had systemic reactions (11 anaphylaxis) and 41 had local reactions, including 22 large local reactions. Only 19 (42%) of those with systemic reactions went to the ER. Nine out of the 11 who had anaphylaxis went to the ER, but only one was treated with adrenaline. On discharge, an AAI was prescribed to one beekeeper, 4 reported to already have AAI and solely 2 were referred to an Allergist. Twenty-five (22%) used alternative treatments on the sting site, namely, metal, lemon or grape, ammonia, alcohol, vinegar and urine. Forty-one (36%) were aware of the existence of AAI, 35 (30%) of VIT and 32 (28%) of the medical specialty of Immunoallergology. Beekeepers with systemic reactions have demonstrated a better knowledge of AAI and VIT, p < 0.01 and p = 0.03, respectively. Awareness of AAI, VIT and Immunoalergology was not significantly different between gender, age and education groups.

**Conclusions**: This group of Portuguese beekeepers showed insufficient knowledge on proper management of sting reactions. Approximately one fifth used alternative treatments. Adrenaline underuse, underprescription, as well as an inefficient referral to a specialist, was evident in the ER. There is a rise in beekeeping in Portugal and so it is imperative to promote education on the risks, sting prevention measures and correct treatment of stings. The increasing awareness of the medical specialty of Immunoallergology and the existence of bee venom immunotherapy should assure a prompt referral in case of systemic reactions which is crucial in this population.

### PD23 Are atopy patch tests useful to identify food sensitisation in eosinophilic esophagitis?

#### Luis Amaral^1^, Leonor Carneiro-Leão^1^, Susana Rodrigues^2^, Alice Coimbra^1^

##### ^1^Serviço de Imunoalergologia, Centro Hospitalar de São João E.P.E., Porto, Portugal; ^2^Serviço de Gastrenterologia, Centro Hospitalar de São João E.P.E, Porto, Portugal

**Correspondence**: Luis Amaral - luis.m.amaral@gmail.com

*Clinical and Translational Allergy* 2017, **7(Suppl 1)**:PD23

**Introduction**: Eosinophilic esophagitis (EoE) is an inflammatory disease of the esophagus characterized by symptoms related to esophageal dysfunction, eosinophilic recruitment and infiltration of the esophageal epithelium. A combined mechanism of IgE dependent/cellular mediated hypersensitivity reactions to foods is thought to contribute to disease pathogenesis.

Our aim was to assess food sensitization in EoE patients with atopy patch tests (APT); A secondary aim was to find any relation between the results of APT, skin prick tests (SPT) and/or specific IgE (sIgE) with response to the six-food elimination diet (SFED).

**Methods**: A longitudinal study of adult patients diagnosed with EoE was carried out. Patch tests were performed with 9 foods: cow’s milk, hen’s egg, wheat, soy, peanut, walnut and hazelnut were applied undiluted, 1/10 dilution and 1 drop of allergen extract; shrimp and cod were applied raw, cooked and 1 drop of allergen extract. The APT were delivered using 8 mm Finn Chambers^®^. Occlusion time was 48 h and the results were read 20 min and 24 h after removal. Endoscopy results, SPT, sIgE, medical management and patients’ responses were collected.

**Results**: Twelve patients agreed to participate, 3 were female with a median (interquartile range, IQR) age of 23 (9) years age, 8 with allergic rhinitis and 2 asthma; 8 were sensitized to aeroallergens, 6 to house dust mites and 4 to pollens. Seven patients had positive food SPT and/or sIgE (3 cow’s milk, 2 wheat and 2 LTPs). Ten patients presented clinical and histological improvement with the SFED and in 8 symptoms and eosinophils recurred on food reintroduction (6 with cow’s milk and 3 with wheat). Only 2 patients presented positive APT: 1 to undiluted hazelnut, who was previously sensitized to LTP; and the other to undiluted soy, which was not clinically relevant since the patient frequently eats soy without any immediate symptoms or worsening of EoE.

**Conclusions**: Standardization on food APT is lacking. In this series, we did not observe any clinical utility for identifying food sensitivity with atopy patch tests in adult patients with Eosinophilic esophagitis.

### PD24 How well can hazelnut allergens distinguish between mild and severe hazelnut allergy?

#### Mareen Datema^1^, Laurian Jongejan^1^, Ronald van Ree^1^, Michael Clausen^2^, Andre Knulst^3^, Nikolaos Papadopoulos^4^, Marek Kowalski^5^, Frédéric de Blay^6^, Aeilko Zwinderman^1^, Jonas Lidholm^7^, Stefan Vieths^8^, Karin Hoffman-Sommergruber^9^, Clare Mills^10^, Barbara Ballmer-Weber^11^, Montserrat Fernandez-Rivas^12^

##### ^1^Academic Medical Centre, University of Amsterdam, Amsterdam, the Netherlands; ^2^Landspitali University Hospital, Reykjavik, Iceland; ^3^University Medical Center Utrecht, Utrecht, the Netherlands; ^4^Centre for Paediatrics and Child Health, Institute of Human Development, University of Manchester, Manchester, United Kingdom; ^5^Medical University of Lodz, Lodz, Poland; ^6^University Hospital of Strasbourg, Strasbourg, France; ^7^Phadia AB, Upsala, Sweden; ^8^Paul-Ehrlich-Insitut, Federal Institute for Vaccines and Biomedicines, Langen, Germany; ^9^Medical University of Vienna, Vienna, Austria; ^10^Manchester Institute of Biotechnology, University of Manchester, Manchester, United Kingdom; ^11^University Hospital Zürich, Zürich, Switzerland; ^12^Hospital Clinico San Carlos, IdISSC, Madrid, Spain

**Correspondence**: Mareen Datema - m.r.datema@amc.nl

*Clinical and Translational Allergy* 2017, **7(Suppl 1)**:PD24

**Introduction**: Component-resolved diagnosis (CRD) has been shown to improve hazelnut allergy diagnosis. Some allergens have been associated with severity of hazelnut allergy, however few studies have reported the classification accuracy of the markers in discriminating between mild and severe subjects, especially in adults.

Our aims were:

1. to analyze the association between sensitization to single hazelnut allergens and clinical symptom severity;
2. to evaluate their ability to discriminate between mild and severe hazelnut allergy.

**Methods**: Subjects (n = 731) from 12 European cities reporting reactions to hazelnut (83.6% adults) were included. In all subjects, sensitization against hazelnut extract and in 423/731, IgE against seven single components (rCor a 1, rCor a 2, rCor a 8, nCor a 9. nCor a 11, nCor a 12, rCor a 14) and CCD was measured. Additionally 124/731 underwent a DBPCFC of which 86 were reactive. Symptoms to hazelnut were categorized into mild, moderate and severe symptoms. Associations between sensitization to hazelnut and severity were analyzed using multinomial regression analyses. The discriminative ability of hazelnut allergens was evaluated by Receiving operating curve (ROC) analysis.

**Results**: Cor a 9 and Cor a 14 were both positively related with severe symptoms (OR 2.89[1.31–6.34] and OR 4.67[1.76–12.37] respectively) and Cor a 11 and Cor a 12 showed a positive trend in severity. Cor a 1 and 2 were negatively associated with severity (OR 0.36 [0.20–0.64]) and 0.50 [0.24–1.04], respectively). ROC analysis showed a poor to moderate ability to discriminate between reported mild and severe hazelnut with AUCs ranging from 0.50–0.62. However, AUCs for Cor a 9, Cor a 11 and Cor a 14 was significantly better in children, the DBPCFC group and using the combination of these allergens (AUC 0.70–0.86). Sensitivities for Cor a 9 and Cor a 14 were low (24%). Around 75% of the severe subjects with confirmed hazelnut allergy was not sensitized to these allergens.

**Conclusion**: Sensitization to Cor a 9 and Cor a 14 is associated with severity of hazelnut allergy however, the classification power was moderate in our adult dominated population. Unidentified allergens might elicit the severe symptoms but further research is needed to identify these allergens and to confirm their clinical relevance.

### PD25 The clinical utility of basophil activation testing in diagnosis of mugwort pollen-associated peach allergy

#### Shan Deng, Jia Yin

##### Peking Union Medical College Hospital, Beijing, China

**Correspondence**: Shan Deng - dengshan2000@126.com

*Clinical and Translational Allergy* 2017, **7(Suppl 1)**:PD25

**Introduction**: Our aim was to assess the performance of basophil activation testing (BAT) as a diagnostic marker for mugwort pollen-associated peach allergy.

**Methods**: Peach allergic (n = 89), peach-sensitized but tolerant (n = 52) and non-peach-sensitized nonallergic (n = 10) patients underwent sIgE to peach and its components. BAT was performed using flow cytometry.

**Results**: The patients with peach allergy had higher IgE levels for peach and Pru p 3 than peach sensitized population (*P* < 0.01). By stimulation with peach extract, BAT in peach-allergic patients showed a significant dose-dependent upregulation of CD63 compared with peach sensitized but tolerant and non-peach-sensitized nonallergic patients. While stimulated with Pru p 3, BAT could also discriminate between peach allergy and tolerance. Receiver operating characteristic curves showed basophil reactivity had larger area under the curve than IgE to peach (AUC 0.744, 95% CI 0.550–0.937, *P* = 0.039) and Pru p 3 (AUC 0.865, 95% CI 0.705–1.000, *P* = 0.002); while BAT stimulated with Pru p 3 had the largest area at 0.981 and stimulation with peach extract (100 ng/ml) at 0.942. Previous studies concerning pollen-associated food allergy have compared between healthy controls and food allergic patients, without addressing the possible effect of immunologic cross-reactivity on the performances of BAT. In this study, we performed a comparative analysis with peach-sensitized but tolerant patients. BAT stimulated with the major allergen is better than that with the crude allergen extract in discriminating between peach allergy and tolerance.

**Conclusion**: BAT stimulated with Pru p 3 is superior to other diagnostic tests in diagnosis of mugwort pollen-associated peach allergy.

### PD26 Usefulness of serum-levels of histamine, tryptase, Cys-LTs and 9α11β-PGF2 during oral food challenge

#### Charlotte Eisenmann, Maria Nassiri, Rabea Reinert, Sabine Dölle, Margitta Worm

##### Department of Dermatology and Allergy, Allergie-Centrum-Charité, Charité-Universiätsmedizin Berlin, Berlin, Germany

**Correspondence**: Charlotte Eisenmann - charlotte.eisenmann@charite.de

*Clinical and Translational Allergy* 2017, **7(Suppl 1)**:PD26

**Introduction**: Food Allergy (FA) is a common disease and it is estimated prevalence world-wide ranges between 2–10% with an increasing trend. Peanuts and tree nuts are frequent causes of FA and may albeit rare result in fatal reactions. The diagnosis of FA is based on the personal history, *in vivo* skin-testing (e.g. SPT), *in vitro* IgE-testing and oral challenges. The double-blind placebo controlled food challenge (DBPCFC) is the gold standard for diagnosing and categorizing FA. DBPCFC’s are time consuming and bear a risk for the patient. Moreover, sometimes the interpretation of test reactions is difficult. The aim of this study was to analyze mast cell mediators like histamine, tryptase, cys-LTs and 9α11β-PGF2 in serum samples from patients who underwent oral food challenges (OFC) and correlate these parameters with the reaction-severity.

**Methods**: 32 patients from the Allergy-Center with a history of FA to peanuts or tree nuts were recruited. 40 OFCs were performed. Reaction severity was determined by using the grading according to *Ring&Messmer*. Severe reactions were observed in 23 cases (grade = 2 in 10 patients, grade = 3 in 13 patients). Blood samples were collected before OFC (T1), 5–10 min after the reaction (T2) and 2 h post-onset (T3). Histamine (LDN, Nordhorn, Germany), cys-LTs (LTC4, LTD4, LTE4) and 9α11β-PGF2 (both Cayman Chemical, Ann Arbor, USA) were determined by ELiSA. Tryptase was kindly measured by Thermo Fisher Scientific, Freiburg, Germany.

**Results**: We observed a significant increase of tryptase, cyc-LTs and 9α11β-PGF2 after food provocation but not histamine in sera from patients with positive OFC. Tryptase levels correlated significantly with the severity of the reaction and a positive tendency of this correlation was also observed for histamine- and 9α11β-PGF2-levels, but not cys-LTs values.

**Conclusion**: The studied markers differ in their reliability. Histamine does not support evidence for a positive OFC. By contrast, tryptase but also 9α11β-PGF2 (alone or combined) may be used as supportive markers for diagnosing FA. Whether the total increase of the markers helps to predict severity in a given patient will need to be assessed in future studies.

### PD27 sIgE Ana o 1, 2 and 3 accurately distinguish tolerant from allergic children sensitised to cashew nuts

#### Johanna P. M. van der Valk^1^, Roy Gerth van Wijk^1^, Yvonne Vergouwe^2^, Ewout W. Steyerberg^2^, Marit Reitsma^3^, Harry J. Wichers^3^, Huub F. J. Savelkoul^4^, Berber Vlieg-Boerstra^5^, Hans de Groot^6^, Anthony E. J. Dubois^7^, Nicolette W. de Jong^1^

##### ^1^Department of Internal Medicine, Allergology, Erasmus MC, Rotterdam, the Netherlands; ^2^Center for Medical Decision Making, Department of Public Health, Erasmus MC, Rotterdam, the Netherlands; ^3^Wageningen UR Food & Biobased Research, Wageningen, the Netherlands; ^4^Laboratory of Cell Biology and Immunology, Wageningen University, Wageningen, the Netherlands; ^5^Department of Paediatrics, Onze Lieve Vrouwe Gasthuis (OLVG), Amsterdam, the Netherlands; ^6^Department of Pediatric Allergology, Diaconessenhuis Voorburg, RdGG, Delft, the Netherlands; ^7^Department of Pediatric Pulmonology and Pediatric Allergology, University Medical Centre Groningen, GRIAC Research Institute, University of Groningen, Groningen, the Netherlands

**Correspondence**: Johanna P.M. van der Valk - j.p.m.vandervalk@erasmusmc.nl

*Clinical and Translational Allergy* 2017, **7(Suppl 1)**:PD27

**Introduction**: The double-blind, placebo-controlled food challenge test (DBPCFC) is the gold standard in cashew nut allergy. This test is costly, time-consuming and not without side effects. Analysis of IgE-reactivity to cashew nut components may reduce the need for food challenge tests.

**Methods**: In a prospective and multicentre study, children with suspected cashew nut allergy underwent a DBPCFC with cashew nut. Specific IgE to total cashew nut and to the components Ana o 1, 2 and 3 were determined. A skin prick test (SPT) with cashew nut extract was performed. The association between the outcome of the food challenge test and specific IgE to Ana o 1, 2 and 3 was assessed with logistic regression analyses, unadjusted and adjusted for other diagnostic variables. Discriminative ability was quantified with a concordance index (c).

**Results**: 173 children (103 boys, 60%) with a median age of 9 years were included. 79% had a positive challenge test outcome. A steep rise in the risk of a positive challenge was observed for specific IgE to each individual component Ana o 1, 2 and 3 with estimated risks up to approximately 100%. Specific IgE to Ana o 1, 2 and 3 better distinguished between cashew-allergic and tolerant children (c = 0.87, 0.85 and 0.89 respectively), than specific IgE to cashew nut or SPT (c = 0.76 and 0.83 respectively). In the multivariable models inclusion of sIgE to Ana o 1, 2 or 3 increased the c-index of history and gender from 0.67 to 0.89 maximally, with increases up to 0.92 and 0.93, respectively when also specific IgE to cashew nut or both IgE and SPT were added.

**Conclusion**: The major cashew nut allergens Ana o 1, 2 and 3 are each individually predictive for the outcome of food challenge tests in cashew-allergic children.

### PD28 Non-ionic iodinated contrast media-induced anaphylaxis – Case series

#### Fabrícia Carolino, Ana Rodolfo, Josefina Cernadas

##### Serviço de Imunoalergologia, Centro Hospitalar São João, E.P.E., Porto, Portugal

**Correspondence**: Fabrícia Carolino - fabricia.c@sapo.pt

*Clinical and Translational Allergy* 2017, **7(Suppl 1)**:PD28

**Introduction**: To assess the prevalence and describe the cases of anaphylaxis to iodinated contrast media (ICM), evaluated in our Drug Allergy Unit (DAU) in the last 6 years.

**Methods**: Retrospective analysis of medical records of patients assessed in our DAU for suspected hypersensitivity reactions (HSR) to ICM between Jan/10 and Dec/15 (n = 54). Skin prick tests (SPT) were performed with potassium iodate (PI), iodopovidone, and a panel of available non-ionic ICM (iohexol, ioversol, iobitridol, iopromide, iomeprol, iodixanol, amidotrizoate, meglumine); intradermal tests (IDT) were also performed with ICM diluted 1/100–1/10, and undiluted (not in all cases).

**Results**: Eighteen (33.3%) patients presented with anaphylaxis (10 females, median age 65.5 years, interquartile range 43.0–72.0 years). The clinical manifestations were respiratory (n = 15, 83.3%), mucocutaneous (n = 12, 66.7%), cardiovascular (n = 11, 61.1%) and gastrointestinal (n = 5, 27.8%). No fatal outcomes occurred. Suspect ICM were iobitridol (n = 4, 22.2%), iopromide (n = 3, 16.7%), ioversol (n = 2, 11.1%), iomeprol (n = 1, 5.6%), iohexol (n = 1, 5.6%), and unknown in 7 (38.9%) cases. SPT with iodopovidone and PI were all negative. One patient (anaphylactic shock to iopromide) had a positive SPT (iopromide). In IDT, 4 patients had positive test results to ICM at 1/10 (including the suspect): (1) iopromide and iomeprol; (2) ioversol, iobitridol, iodixanol and amidotrizoate; (3) iobitridol and iopromide; (4) iohexol. The patient with anaphylactic shock to iopromide reacted at 1/100. Other 8 patients had positive IDT only to undiluted solution, with at least 1 of the ICM tested (including the suspect); 6 of these patients were tested to other undiluted ICM with negative results. Skin testing with a panel of ICM is recommended by the ENDA group in these patients, not only to confirm the culprit drug but to assess cross-reactivity and find safe alternatives. The ENDA position paper (2013) makes a weak recommendation to avoid undiluted ICM for skin tests as it may be irritative. Six of eight patients with a positive IDT to the undiluted suspected ICM had a negative test to at least two other undiluted ICM.

**Conclusion**: In this study we report 30% of ICM HSR fulfilling anaphylaxis criteria, and 70% of these patients had a positive diagnostic work-up. The authors raise the question of whether the IDT should routinely be performed including an undiluted solution, since some potentially allergic patients may be missed otherwise.

### PD29 Allergy to lipid transfer protein in pediatric population at the third level hospital in Madrid

#### Dasha Roa-Medellín, Ana Rodriguez-Fernandez, Joaquín Navarro, Vicente Albendiz, María Luisa Baeza, Sonsoles Intente-Herrero

##### Hospital General Universitario Gregorio Marañón, Madrid, Spain

**Correspondence**: Dasha Roa-Medellín - dasharoa@gmail.com

*Clinical and Translational Allergy* 2017, **7(Suppl 1)**:PD29

**Introduction**: Lipid transfer protein (LTP) Syndrome is a primary food allergy with high prevalence in South Europe population. Its clinical characteristics and prevalence are not well established in the paediatric population.

**Methods**: A retrospective-descriptive study from Jan 2013 to Dec 2015 of children with sensitization to Specific IgE to Pru p 3 and clinical manifestation in a third-level paediatric hospital was conducted. It was aimed to study and compare the clinical patterns of sensitization.

**Results**: Eighty-three allergic children with sensitization to Pru p 3 were found. Forty-nine male (59%) and thirty-four females: (41%) were assisted. The average age of first reaction: media: 5.3 years +/−3.6 STD. The history of atopic diseases were: rhinitis 39%, asthma 14% with a broad spectrum of pollen sensitization (Grass pollen: 55.4%, Olea 49.3%, plane tree 25%, Cypress 24%, mugwort 21%) and atopic dermatitis: 32%. The first food trigger involved were peach 36.2% (35), peanut 15.2% (14) walnut 12.2% (11), others fruits 11.2% (10), other legumes 9.2% (8) and other nuts 6.2% (5). The most common first clinical manifestation found was: urticarial 44.2% (43), following of oral allergy syndrome: 15.2% (14), anaphylaxis: 14.2% (13) and cutaneous rash: 12.3% (11), The exercise was a cofactor in a 3% of the population and NSAIDs were not involved. All patients were positive skin prick test and high levels of IgE specific levels to pru p3 median; 8.5 (14.58) IQR.

**Conclusion**: Severe plant food unrelated independent of peach are involved in LTP Syndrome. Grass and olea pollen are the main aeroallergen in our population. Peach and peanut are the main food elicitors. Urticarial symptoms are the most common clinical manifestation.

### PD30 Long term impact of cow’s milk allergy on children and their families – A 7-year follow-up

#### Andrea Mikkelsen^1,2^, Kirsten Mehlig^2^, Lauren Lissner^2^

##### ^1^Primary Care Research and Development Centre, Gothenburg, Sweden; ^2^Department of Public Health and Community Medicine, Section of Epidemiology and Social Medicine, Sahlgrenska Academy, University of Gothenburg, Gothenburg, Sweden

**Correspondence**: Andrea Mikkelsen - andrea.mikkelsen@vgregion.se

*Clinical and Translational Allergy* 2017, **7(Suppl 1)**:PD30

**Introduction**: Despite outgrown CMA, we have previously found that many families continue to experience nutritional related problems at a six-month follow-up [1]. Some may develop other food allergies or atopic diseases follow ing the atopic march, and some may fail to progress to unrestricted diet.

Our aims were to assess impact on nutrition related issues at a 7 year follow-up in relation to the development of CMA over time.

**Methods**: Families of children with CMA, who participated in the validation of the Food hypersensitivity famiLy ImPact questionnaire (FLIP), were re-approached 7 years later for follow-up and administered the FLIP.

**Results**: Of the original families (n = 94), at 7-year follow-up 84% (n = 79) agreed to participate. The children had a mean age 8.5 years (r = −11 years) and n = 30 (38%) were girls. The majority (n = 49.62%) no longer needed to follow a cow’s milk free diet. The remaining children (n = 0) were still following a special diet due to CMA exclusively (n = 7.9%) or in combination with other food allergy (n = 9.11%) and other food allergy excluding CMA (n = 14.18%). Most children were healthy (n = 67) but n = 8 (10%) had developed other atopic diseases or other non-atopic diseases (n = 3). These findings are in line with those from others [2]. A mixed linear model for the FLIP Nutrition subscale across three time points (baseline, 6-month and 7-year follow-up respectively) in relation to allergy status at 7-year follow-up (i.e. outgrown vs. persistent) showed only a marginally significant improvement in nutrition related issues for the group outgrown CMA (p = 0.07) at 7-year follow-up. For the group with persistent CMA there was no difference in the experienced impact. Despite outgrown CMA, n = 13 children in the group outgrown CMA had a restricted consumption of dairy due to fear of reactions (n = 4) or dislike of milk as a drink (n = 6), or both as a drink and when contained in food (n = 3). The latter group were still consuming non-dairy special products.

**Conclusions**: There is only a small improvement in nutrition related issues despite outgrown CMA at 7-year follow-up. Many families continue to serve a restricted diet despite outgrown CMA. Nutritional counselling should be considered to all families with children with persistent as well as outgrown CMA in order to ensure optimal nutritional intake, development of eating behaviour and progression to unrestricted diet preventing unnecessary limitations in daily life.

**Acknowledgements**: Financial support: FoU VG-region post-doctoral scholarship.

**References**

1. Mikkelsen A, et al. Monitoring the impact of cow’s milk allergy on children and their families with the FLIP questionnaire—a six-month follow-up study. Pediatr Allergy Immunol. 2015.
2. Spergel JM. Natural history of cow’s milk allergy. J Allergy Clin Immunol. 2013;131(3):813–4.

### PD31 Perception of allergen exposure risks in US adults with self-reported food allergies

#### Linda Verrill, Stefano Luccioli

##### Center for Food Safety and Applied Nutrition, FDA, College Park MD, USA

**Correspondence**: Stefano Luccioli - stefano.luccioli@fda.hhs.gov

*Clinical and Translational Allergy* 2017, **7(Suppl 1)**:PD31

**Introduction**: Self-reported food allergies (srFA) have increased in US adults over the past decade. Information on how this population perceives allergen exposure risks is lacking. We sought to identify adults with srFA and assess responses to questions about allergen advisory statements and thresholds.

**Methods**: We analyzed FDA 2016 Food Safety Survey data in 4619 adults to identify survey respondents with srFA, including reported doctor-diagnosed cases (ddFA). Respondents were asked about knowledge of thresholds or reaction risks and about consumption of products with certain advisory statements or trace allergen amounts.

**Results**: Weighted prevalence of srFA among survey respondents was 13.5% with ddFA representing 8.0%. The most common foods associated with srfA and ddFA were shellfish, milk, fruits, tree nuts, peanuts, wheat, fish and egg. Prior anaphylaxis history was reported in 24.8% of respondents. With regards to allergen exposure risk, only 33.2% of srFA respondents were aware of the concept of threshold levels, yet 59.0% reported belief that a level existed below which allergic reaction would not occur. 43.7, 39.0 and 48.8% of respondents reported having consumed or likely to consume products with advisory statements of “may contain”, “made with same equipment” or “produced in same facility” respectively, and 38.2% of respondents reported that they would consume products with trace amounts of the food allergen if there were assurances that allergen amount would not trigger reaction. No significant differences in responses were noted between srFA respondents with or without ddFA except ddFA respondents were more likely to view trace allergen amounts as posing higher risk for severe reactions. Respondents with prior anaphylaxis, shellfish or fish allergy were more likely to avoid products with one or more advisory statements while those with peanut, tree nut, milk or wheat allergy were more likely to have consumed these products. Survey data of adults with self-reported food allergies reveal different perceptions of thresholds and individual exposure risks. More education on understanding allergen exposure risks is needed for this population.

**Conclusion**: Survey data suggest that only one third of srFA respondents understand what allergen thresholds represent, yet over 40% report consuming foods with certain advisory statements. Differences in risk perception may depend on having a ddFA, a prior history of anaphylaxis or on type of food allergen.

### PD32 Tolerable dose during oral immunotherapy using IFN-gamma for anaphylactic food allergy: desensitisation and tolerance

#### Geunwoong Noh^1^, Eun Ha Jang^2^

##### ^1^Department of Allergy, Jeju Halla General Hospital, Jeju, Korea Republic; ^2^Department of Respiratory Medicine, Hanmaeum General Hospital, Jeju, Korea Republic

**Correspondence**: Geunwoong Noh - admyth@naver.com

*Clinical and Translational Allergy* 2017, **7(Suppl 1)**:PD32

**Introduction**: Oral immunotherapy using IFN-gamma (OIT) for anaphylactic IgE-mediated food allergy (AFA) has been done more than 10 years successfully. In this study, a tolerable dose was introduced during tolerance induction with interferon gamma, and its effectiveness and theoretical meaning was evaluated.

**Methods**: The study population comprised 30 AFA patients. Blood samples were taken for analysis, including complete blood count with differential counts of eosinophils, serum total IgE levels, and specific IgE for allergenic foods. Skin prick tests were conducted with the allergens. Oral food challenges were performed to diagnose AFA. Fifteen patients received OIT, 5 received classic OIT without tolerable dose, 5 received OIT without interferon gamma, and 5 were not treated (controls).

**Results**: Patients treated with OIT using IFN-gamma became tolerant to the allergenic food with and without introducing tolerable dose. The tolerable dose was introduced successfully in OIT using IFN-gamma. It was meaningless to introduce tolerable dose in OIT without IFN-gamma. Following the introduction of tolerable dose, the systemic reaction to oral intake subsided, but the local skin reaction to contact with the allergenic food persisted. Tolerable dose was successfully introduced during the oral immunotherapy using IFN-gamma. Conventional oral immunotherapy without using IFN-gamma, the concept of tolerable dose is meaningless because the therapeutic dose is the same or less than the tolerable dose. However, using IFN-gamma, therapeutic dose is escalated much more than that without using IFN-gamma. From the successful introduction of tolerable dose, the nature of tolerance induction was unveiled. Also, the exact meaning of desensitization and tolerance is clarified. By introducing tolerable dose, doctor and patient can confirm the progress of getting tolerance.

**Conclusion**: Introduction of tolerable dose improved the protocol for OTI using interferon gamma for AFA. In food allergy, the concept of tolerance and desensitization is clearly defined. Tolerance is the result of desensitization to allergens. The local skin reaction and systemic reaction to oral intake were dissociated following OIT for AFA.

### PD33 Risk-benefit assessment of nutritional immune interventions during early life

#### Jolanda van Bilsen^1^, Frieke Kuper^1^, André Wolterbeek^2^, Tanja Rouhani Rankouhi^1^, Lars Verschuren^1^, Hilde Cnossen^1^, Prescilla Jeurink^3^, Johan Garssen^4^, Léon Knippels^4^, Jossie Garthoff^3^, Geert Houben^1^, Winfried Leeman^1^

##### ^1^TNO, Zeist, the Netherlands; ^2^Triskelion, Zeist, the Netherlands; ^3^Nutricia Research, Utrecht, the Netherlands; ^4^Utrecht Institute for Pharmaceutical Sciences, Utrecht, the Netherlands

**Correspondence**: Jolanda van Bilsen - j.vanbilsen@tno.nl

*Clinical and Translational Allergy* 2017, **7(Suppl 1)**:PD33

**Introduction**: The immune health status is strongly determined during early life stages. Many immune-related diseases are thought to find their origin in adverse shifts in immune balances during pregnancy or the first 2–3 years of life, including atopic diseases. Therefore, immune health interventions during these stages of life may be most effective in reducing the loss of health, loss of quality of life and costs to society due to immune-related diseases and disorders. Several starting points for immune health interventions have been identified and are being developed into prophylactic or therapeutic approaches, particularly targeted at the early life stages. Unfortunately, there is no consensus on which parameters should be addressed to assess the safety and/or efficacy of the interventions and how all the available data should be interpreted at the end. Hence, it would be extremely helpful to address this issue by developing a pragmatic, flexible and science-based risk-benefit assessment.

**Methods**: We adapted the risk-benefit approach [1], to develop a framework for risk-benefit assessment of immune health interventions during early life stages. As case studies, we collected all available in vitro/vivo/silico and human data on galacto-oligosaccharides (GOS) and fructo-oligosaccharides (FOS).

**Results**: The severity of hazard and beneficial effects observed and the incidence at which such an effect may be considered acceptable, were used to weigh the risk and beneficial effects. Dose response relationships can be converted to 50% effect doses which, combined with the severity of the effect underlying the dose response curve and concurrent acceptable incidence of this effect, can be used for weighing benefit and risk. In the FOS/GOS case study, several prerequisites of (pre-)clinical data were identified. This risk-benefit framework enables us to evaluate all intervention data available and forms the basis to derive the optimal dose levels of intake.

**Conclusion**: This novel approach enables risk assessors to take the multitude of different types of data available covering toxicity and efficacy studies at early life stages into account, by ranking and weighing all available data. Ultimately, this assessment will help to determine optimal beneficial and safe dose levels of nutritional immune interventions during early life.

**Reference**

1. Renwick AG, Flynn A, Fletcher RJ, Müller DJG, Tuijtelaars S, Verhagen H. Risk-benefit 726 analysis of micronutrient. Food Chem Toxicol. 2004;42:1903–22.

### PD34 FABER 244 IgE test in food allergy. Diagnostic accuracy for LTP proteins

#### Claudia Alessandri^1^, Maria Antonetta Ciardiello^2^, Lisa Tuppo^2^, Ivana Giangrieco^2^, Danila Zennaro^1^, Rosetta Ferrara^1^, Maria Livia Bernardi^1^, Georg Mitterer^3^, Chiara Rafaiani^1^, Michela Ciancamerla^1^, Maurizio Tamburrini^2^, Christian Harwanegg^3^, Adriano Mari^1^

##### ^1^Centri Associati di Allergologia Molecolare - CAAM, Rome, Italy; ^2^Istituto di Bioscienze e Biorisorse, Consiglio Nazionale delle Ricerche, Naples, Italy; ^3^MacroarrayDx, Vienna, Austria

**Correspondence**: Adriano Mari - adriano.mari@caam-allergy.com

*Clinical and Translational Allergy* 2017, **7(Suppl 1)**:PD34

**Introduction**: FABER 244 is a new in vitro multiplex test for specific IgE detection using 122 molecular allergens and 122 allergenic extracts, coupled to chemically activated nanoparticles. Allergenic preparations, either produced in house or obtained from commercial providers, are individually coupled to nanobeads by means of optimized protocols to achieve maximum test performance.

Aim was to measure the diagnostic accuracy of FABER 244-122-122 01 by comparing with the ImmunoCAP ISAC 112 (TFS, Sweden) in patients allergic to LTP proteins shared by both tests.

**Methods**: A real life study as been set by analyzing the clinical records, and the FABER and ISAC test results from 94 consecutive patients referred to CAAM from March 2015 to August 2016. Data were extracted from the electronic medical record InterAll. The diagnostic accuracy measurement and test comparison were performed on 3 LTP proteins (Cor a 8, Jug r 3, Pru p 3) adopting the patients’ symptoms as gold standard and MedCalc as software for statistical analysis. ISAC test Limit of Detection (LoD) was set to 0.3 ISU whereas FABER LoD was set to 0.01 FIU. In the overall analysis attention has been put on additional information coming from non-shared LTPs, Tri a 14 and Ara h 9 for ISAC, and Act d 10, Pun g 1, Sola l 6, Tri a 7 k-LTP, Zea m 14 plus peanut and wheat extracts available on FABER.

**Results**: Both tests appear to be accurate and well aligned to each other for all three analyzed LTP proteins. In terms of sensitivity FABER performs better than ISAC on Cor a 8 (100 vs 73%), same as ISAC on Jug r 3 (100% both) and FABER better than ISAC on Pru p 3 (96 vs 91%). In terms of specificity FABER performs better than ISAC on Cor a 8 (96 vs 94%), same as ISAC on Jug r 3 (96%), whilst ISAC performs better on Pru p 3 (97 vs 92%). IgE detection by ISAC Ara h 9 and Tri a 14 was compensated by the peanut and wheat extracts, whereas info on additional food LTPs could be obtained from the FABER extended panel including 25 extracts from plant-derived foods bearing LTPs.

**Conclusions**: FABER test is a new in vitro test for specific IgE detection, including molecules and extracts. Considering the food allergen group belonging to the LTP, FABER appears to be accurate and in good agreement with ISAC results. The specific advantage of FABER relies on the chance of testing patients to a broader panel of LTP as well as to a large number of extracts, complementing the results on single allergenic molecules.

### PD35 Greater severity of reaction in high versus low fat matrix peanut challenges

#### M. Eleonore Pettersson^1,2^, Afke M. M. Schins^1,2^, Gerard H. Koppelman^1,2^, Boudewjin J. Kollen^3^, Anthony E. J. Dubois^1,2^

##### ^1^Department of Pediatric Pulmonology and Pediatric Allergology, Beatrix Children’s Hospital, University of Groningen, University Medical Center Groningen, Groningen, the Netherlands; ^2^GRIAC Research Institute, University of Groningen, University Medical Center Groningen, Groningen, the Netherlands; ^3^Department of General Practice, University of Groningen, University Medical Center Groningen, Groningen, the Netherlands

**Correspondence**: M. Eleonore Pettersson - m.e.pettersson@umcg.nl

*Clinical and Translational Allergy* 2017, **7(Suppl 1)**:PD35

**Introduction**: Previous studies have shown that the qualities of the matrix containing an allergenic food may impact the severity of reactions during DBPCFCs. The aim of this study was to examine the impact of the matrix fat content during DBPCFCs to peanut and its effect on the severity of the reaction and eliciting dose.

**Methods**: Data was collected from the food challenge unit database at the University Medical Center Groningen (November 2002–May 2014), where DBPCFCs were performed as part of routine clinical care. All children during this period with a positive DBPCFC to peanut were included in the analysis. The food challenges were included if they were performed with the two most frequently used recipes (n = 11 excluded). The recipes used were peanut in cookies and peanut in gingerbread, with a fat content of 23.9% and 5.9% respectively. In children with multiple DBPCFCs, only the first test was included (n = 37 tests excluded). 14 cases were excluded due to missing data. For the severity of reaction during the DBPCFC a previously published scoring system was used, with a severity index ranging from 0 to 12. The influence of the matrix on the severity of reaction and eliciting dose was analyzed by linear regression analysis, with correction for possible confounders. A variable was considered a confounder if it changed the beta coefficient by more than 10%. The alpha significance level was set at 0.05.

**Results**: 210 children were included in the analysis, 141 children were challenged with peanut in cookies (high fat) and 69 children with peanut in gingerbread (low fat). Children challenged with peanut in cookies had more severe reactions during the DBPCFC (β = 0.15, 95% CI 1.49–0.06, p = 0.03), compared to children challenged with peanut in gingerbread. However, there was no significant difference in eliciting dose (β = 0.03, 95% CI-31.91-51.11, p = 0.65). These results were not confounded by age, gender, sIgE, severity of the most severe previous accidental reaction, history of atopic dermatitis, asthma or allergic rhinoconjunctivitis.

**Conclusion**: Children receiving a high fat matrix peanut challenge have more severe reactions than children receiving this test in a low fat matrix. This supports the role of the food matrix as an augmentation factor which may enhance the severity of both diagnostic as well as accidental reactions. It also raises the possibility that matrix effects during oral immunotherapy with peanut could be a cause of adverse events during such treatment.

### PD36 The probability of distribution Alfa-gal associated allergy in Ukraine

#### Svitlana Zubchenko

##### Danylo Halytsky Lviv National Medical University, Lviv, Ukraine

**Correspondence**: Svitlana Zubchenko - maruniak.stepan@gmail.com

*Clinical and Translational Allergy* 2017, **7(Suppl 1)**:PD36

**Introduction**: Annually number of reports about anaphylaxis caused by food products increases. The development of IgE-mediated reactions to food that is well tolerated in the past, now, sometimes is a true diagnostic and therapeutic challenge for the patient and the doctor. An example of such allergies is delayed allergy to red meat that is associated with the formation of IgE antibody to the oligosaccharide galactose-alfa-1,3-galactose (Alfa-gal). During evolution human body formulated the immunological tolerance to Alfa-gal production through IgG. Factor launch IgE-mediated process are tick bites, which linked the spread of geographical features of this type of food allergy (FA). Proved that serious manifestations of anaphylaxis occur in conditions of consumption of fatty meat or large quantities of meat. Determined that Alfa-gal epitope transmitted through the milk that gives grounds to say about later reaction to this product. It is believed that the alternative cause of sensitization to Alfa-gal infection are different types of parasites.

The aim of our study was to determine the probability of distribution associated Alfa-gal allergy among Ukrainian patients.

**Results**: We conducted a comparative assessment of different components of the ticks saliva, on which was identified allergic reactions to Alfa-gal in the USA (Amblyomma americanum), Australia (Ixodes holocyclus) and Europe (Ixodes ricinus). The same ingredients not found in these types of ticks, but each of them has a specific immunotropic action. In particular, I. ricinus, prevalence of which in Ukraine in recent years increased by 14–18% containing biological substances that intensified alternative pathway of complement activation, Th2-response, inhibited proliferation of B lymphocytes.

**Conclusion**: Ukraine has a high probability of spreading Alfa-gal associated FA. The arguments are the same: rising prevalence of I. ricinus, Ukrainian traditional consumption of fatty foods and dairy products, high prevalence of parasitic invasions. There is a necessity of component diagnostics Alfa-gal, which today is not performed. In this case the only way to diagnose is carefully collected history.

### PD37 Natural history of hen’s egg allergy in early childhood

#### Sarah Kuntz, Valérie Trendelenburg, Bodo Niggemann, Kirsten Beyer

##### Department of Pediatric Pneumology and Immunology, Charité Universitätsmedizin Berlin, Berlin, Germany

**Correspondence**: Sarah Kuntz - sarah.kuntz@charite.de

*Clinical and Translational Allergy* 2017, **7(Suppl 1)**:PD37

**Introduction**: Hen’s egg (HE) allergy is the most common food allergy in early childhood. Although there is a good prognosis, reported data on the development of clinical tolerance vary between studies. The aim of our study was to analyze the natural history of HE allergy among children with challenge proven HE allergy.

**Methods**: Data of children undergoing double-blind, placebo-controlled food challenge (DBPCFC) in our center were prospectively recorded. Children who had a DBPCFC with raw HE between 01/2010 and 06/2014 were further evaluated. When data on repeated DBPCFC with either raw or baked egg of the children were not available in our data bank, parents were questioned by telephone with a standardized questionnaire whether their child tolerates raw and/or baked HE in their diet.

**Results**: 110 children with a positive DBPCFC to HE could be included (41 girls and 69 boys with a median age of 1.4 years (0.4 to 9.8 years) at the first DBPCFC with raw HE). After a median period of 34 months (9 to 96 months), 54/110 children (49%) tolerated raw HE (45 had a second DBPCFC to raw HE without reaction, 9 reported complete tolerance in the interview). Of the remaining 56 children without known tolerance to raw HE (still allergic n = 40, unknown n = 12), more than half (54%, n = 30) could tolerate heated HE after a median period of 26 months (20 had a negative DBPCFC to heated HE, 10 reported tolerance to heated HE in the telephone interview). Altogether 29/69 of the boys (42%) developed clinical tolerance to raw HE within a median period of 36 months (9 to 96 months), while 25/41 of the girls (61%) tolerated raw HE already after a median period of 26 months (11 to 66 months). Tolerance development to raw HE in this study was slightly longer than reported from the EuroPrevall birth cohort. This might be explained that re-challenges were not scheduled on yearly basis as in EuroPrevall but on request of the child’s physician.

**Conclusion**: Around half of the children with challenge proven HE allergy develop tolerance to raw HE within a median period of 3 years and even more to heated HE. Girls seem to have better chances to develop tolerance to HE in a shorter period of time than boys, but larger trials are necessary to confirm these observation.

### PD39 Comparing safety profile of boiled egg oral inmunotherapy as an alternative to raw egg

#### Pablo Mérida, Adriana Machinena, Montserrat Álvaro, Jaime Lozano, Monica Piquer, Carmen Riggioni, Juan Heber Castellanos, Maria Teresa Giner, Rosa Jimenez, Olga Dominguez, Ana Maria Plaza

##### Department of Pediatric Allergy and Clinical Immunology, Sant Joan de Déu Hospital, University of Barcelona, Barcelona, Spain

**Correspondence**: Pablo Mérida - p_merida@hotmail.com

*Clinical and Translational Allergy* 2017, **7(Suppl 1)**:PD39

**Introduction**: Improving safety of egg oral immunotherapy (OIT) is a source of concern. Our group has previously published data regarding safety in a protocol to raw egg (RE)-OIT, reporting adverse reactions in 7.6% of doses and adrenaline use in 26%. To evaluate the safety of boiled egg (BE)-OIT in egg allergic children and compare it with previous data on RE-OIT.

**Methods**: Medical records of children following the protocol from January 2015 to July 2016 were reviewed. Retrospective data were collected for demographics, adverse events at oral food challenge (OFC) and during induction phase. Specific-IgE (s-IgE) and skin prick test (SPT) were expressed with median and range (min-max). Written informed consent was signed. Open OFC with BE was performed, confirming allergy. Anaphylaxis was defined following EAACI position paper criteria.

**Results**: 21 patients were enrolled, 76.2% boys, median age 9 years (5–15). At OFC: All had adverse reactions and 9/21 presented anaphylaxis. Total IgE 1141 kU/L (97–3814), ovalbumin-sIgE 3.19 kU/L (0–667), ovomucoid-sIgE 4.47 kU/L (0.89–1480), egg white-sIgE 6.6 kU/L (0–1350); ovalbumin SPT 7.9 mm (2.8–14.7), ovomucoid 9.7 mm (4.2–16.9), egg white 10.4 mm (4–15.6). OIT induction phase: 57.1% (12/21) had adverse events: 42.8% gastrointestinal, 28.5% respiratory and 19% cutaneous. 61.9% (13/21) completed induction reaching at least 5.6 gr of BE (700 mg of protein). 3 patients were excluded for not reaching this dose. 1309 doses were administered and 28 reactions occurred (2.14% of OIT doses). 7/18 had anaphylaxis, 2 patients required adrenaline use (10.5%), 5 withdrew during induction phase (4 due to anaphylaxis and 1 for poor adherence). Egg OIT is still an experimental treatment and aims to modify clinical reactivity in persistently egg-allergic children. BE-OIT enhances children’s diet by including baked and various quantities of fully cooked egg thus improving families quality of life. However, complete tolerance to raw-egg is not attempted. Initial parameters at OFC were comparable to previous RE-OIT group. Adverse events are still a source of concern even with the modified BE-protocol.

**Conclusions**: Gastrointestinal symptoms were the most frequent adverse reactions and anaphylaxis the most common cause of withdrawal. Within similar populations, BE-OIT showed an improvement in safety profile with fewer adverse reactions and less use of adrenaline; proving to be a better OIT alternative for egg allergic children.

**Consent to publish**: Written informed consent was obtained.

## POSTER DISCUSSION SESSION 3: Clinical and experimental aspects • Molecular mechanisms

### PD40 Food allergy has no impact on asthma morbidity except if associated to atopic dermatitis

#### Melanie Cap^1^, Elodie Drumez^2^, Stéphanie Lejeune^1^, Caroline Thumerelle^1^, Clémence Mordacq^1^, Véronique Nève^3^, Antoine Deschildre^1^

##### ^1^Pediatric Pulmonology and Allergy Department, Hôpital Jeanne de Flandre, CHU Lille, Lille, France; ^2^Biostatistics Department, CHU Lille, Lille, France; ^3^Pediatric Pulmonary Function Testing Unit, Hôpital Jeanne de Flandre, CHU Lille, Lille, France

**Correspondence**: Antoine Deschildre - antoine.deschildre@chru-lille.fr

*Clinical and Translational Allergy* 2017, **7(Suppl 1)**:PD40

**Introduction**: Food allergy (FA) is often associated to asthma. We aimed to investigate the impact of FA on asthma control. Exacerbations, maintenance treatment, and lung function were also evaluated.

**Methods**: Longitudinal prospective study conducted between August 2015 and March 2016. Allergic asthmatic Children, ≥7 years old, with or without confirmed FA, were included during a scheduled visit. Asthma and FA characteristics were recorded. Asthma control was defined according to GINA criteria. Asthma Control Test (ACT) or pediatric-ACT, asthma exacerbation rate, oral corticosteroids courses (OCC), hospitalisations in the previous year, GINA maintenance treatment level and ICS dose, lung function (FEV1, FEV1/FVC, FeNO) were compared between children with FA and those without.

**Results**: 212 asthmatic children (mean age: 11.5 years), were included; 57 of them had FA (27%) (nuts = 26, peanut = 23, egg = 11, milk = 3, other = 20, multiple FA = 41). History of life threatening asthma was not different between the 2 groups (p = 0.4). History of food anaphylaxis was documented in 39 patients. 31 children (15%) were treated with Omalizumab, 10 of them with FA. Good control was observed in 38 children with FA (67%), and 94 children without FA (61%) (p = 0.42); there was no difference for ACT or pACT score (p = 0.26), exacerbation rate [mean: 1.2 (+/−2.04) if FA vs 2,22 (+/−3.65) if asthma only (p = 0.1)], OCC (p = 0.08), hospitalisations (p = 0.41), GINA treatment level (p = 0.17), ICS dose (p = 0.16). FEV1/FVC (mean: 84% if FA vs 83%; p = 0.12) and FeNO (median: 42 ppb if FA vs 24 ppb (p = 0.55)] were not different. The type of food allergen, the number of FA, the severity of food reaction had no effect on asthma control. The subgroup of patients with “asthma-atopic dermatitis-FA” (A-DA-FA) had a higher number of exacerbations (p = 0.009) and daily ICS (p = 0.017) compared to patients with asthma, FA and no AD. We did not observe that FA was associated to worse asthma control or morbidity as previously reported [1,2]. Regular follow-up in a tertiary care centre may explain the results. However, we observed that children with FA and AD had a more severe asthma than those with FA but no AD.

**Conclusion**: Our study does not provide evidence for increase asthma morbidity in children with any FA. The association of A-DA-FA characterized a specific phenotype.

**References**

1. Johnson J, et al. PloS One. 2015;10:e0124675
2. Friedlander JL, et al. J Allergy Clin Immunol Pract 2013;1:479–84.

### PD41 Skin prick test and development of tolerance in children with egg allergy

#### Carla Mastrorilli, Sonia Ricò, Margherita Varini, Carlotta Povesi Dascola, Carlo Caffarelli

##### Pediatric Clinic, Department of Clinical and Experimental Medicine, University of Parma, Parma, Italy

**Correspondence**: Carla Mastrorilli - carla.mastrorilli@icloud.com

*Clinical and Translational Allergy* 2017, **7(Suppl 1)**:PD41

**Introduction**: The aim of our study was to examine whether the loss of positive skin prick test (SPT) reactions to egg white or egg yolk predicted the development of spontaneous clinical tolerance in children with egg allergy.

**Methods**: We recruited children with egg allergy, as positive oral food challenge with egg (OFC) or suggestive history and positive SPT reactions to yolk egg and/or egg white. All children underwent SPTs with egg white and yolk extracts. Children whose SPT results to both egg white and egg yolk became negative during follow-up were included in group 1, those with persistent positive SPT results in group 2. The acquisition of tolerance to hen’s egg was ascertained by open OFC in both groups with escalating doses every 20 min of hard-boiled egg. The adverse reactions were considered immediate when they appeared within 2 h and delayed after 2 h. In case of negative TPO, the egg was administered at home for 4 days, with control of any adverse reactions. In case of unclear symptoms to TPO, it was performed a double-blind test, placebo-controlled trial.

**Results**: Seventy-five egg-allergic children (44 males), aged 3–78 months, were enrolled. Among them 23 were assigned to group 1 (negative SPT to yolk and egg white) and 52 in group 2 (positive SPT for yolk and/or egg white). In group 1, a child (4.3%) experienced positive OFC, in group 2, 13 children (25%) did not pass OFC (p < 0.03). In all cases manifestations were immediate, most frequently cutaneous (78%). Persistence of positive SPT was significantly associated with allergic symptoms (atopic dermatitis, oculorhinitis, other food allergies). There was no correlation between persistence of positive SPT, age at first or last egg reaction time interval between the last clinical response and OFC, time interval between the first and last SPT performance, type of symptoms. The diagnostic accuracy of SPT showed best results for sensitivity (0.92) and negative predictive value (0.95).

**Conclusion**: Children with egg allergy are more likely to tolerate the egg in case of negative SPT, compared to subjects with persistently positive SPT. So, loss of positive SPT may be a predictive marker of tolerance and reduce the need for OFC. However, 1 of 23 children with negative SPT had reactions during OFC (4%). Larger population studies are therefore needed to examine possible mild reactions at OFC in case of negative SPT.

### PD42 Extensively hydrolyzed casein formula containing lactobacillus rhamnosus GG prevents the occurrence of other allergic manifestations in subjects with cow’s milk allergy: 3-year randomised controlled trial

#### Rita Nocerino, Linda Cosenza, Antonio Amoroso, Margherita Di Costanzo, Carmen Di Scala, Giorgio Bedogni, Roberto Berni Canani

##### University of Naples “Federico II”, Naples, Italy

**Correspondence**: Rita Nocerino - ritanocerino@alice.it

*Clinical and Translational Allergy* 2017, **7(Suppl 1)**:PD42

**Introduction**: Children with cow’s milk allergy (CMA) have an increased risk to develop other allergic manifestations. We performed a randomized controlled trial (RCT) to test whether the early administration of an extensively hydrolyzed casein formula (EHCF) containing *L.rhamnosus* GG (LGG) can reduce the 3-year incidence of other allergic manifestations in these patients.

**Methods**: A parallel-arm RCT was performed in children aged 1 to 12 months with IgE-mediated CMA. Patients were allocated to one of two groups of dietary interventions: EHCF (Nutramigen^®^, Mead Johnson, Evansville, IN, USA) and EHCF+LGG (Nutramigen LGG^®^, Mead Johnson, Evansville, IN, USA). All subjects were evaluated during a 36 months follow-up. Other allergic manifestations (atopic eczema, allergic urticaria, asthma and oculorhinitis) were diagnosed using standard criteria by pediatricians blinded to group assignment. Tolerance acquisition was evaluated every 12 month by the result of oral challenge.

**Results**: 220 subjects (147 male, 67%) with a mean (SD) age of 5.7 (3) months were randomized, 110 to EHCF and 110 to EHCF+LGG. Binomial regression using intention-to-treat analysis revealed that the absolute risk difference (ARD) for the occurrence of at least one allergic manifestation over 36 months was −0.22 (95% CI −0.35 to −9%, p < 0.001) for EHCF+LGG vs. EHCF. The ARD for the occurrence of atopic eczema was −0.13 (95% CI −0.25 to −1%, p < 0.05), −0.14 (95% CI −0.25 to −2%, p < 0.001) for allergic urticaria, −0.11 (95% CI −0.22 to 0%, p > 0.05) for asthma, and −0.17 (95% CI −0.29 to −6%, p < 0.01) for oculorhinitis in EHCF+LGG vs. EHCF group. Binomial regression for repeated measures using per-protocol-analysis with Bonferroni’s correction for 3 comparisons (contrasts) revealed that the ARD for the acquisition of cow’s milk tolerance was 0.20 (95% CI 0.05 to 0.35, p < 0.01) at 12 months, 0.24 (95% CI 0.08 to 0.41, p < 0.01 at 24 months and 0.27 at 36 months (95% CI 0.11 to 0.43, p < 0.001) for the EHCF+LGG vs. the EHCF group.

**Conclusion**: Compared to EHCF, EHCF+LGG reduces the incidence of other allergic manifestations in children with IgE-mediated CMA. Moreover, the use of this hypoallergenic formula increases the rate of tolerance acquisition at 12, 24 and 36 months.

### PD43 How common is soya allergy in peanut-allergic children?

#### Nandinee Patel, Marta Vazquez-Ortiz, Sarah Lindsley, Paul J. Turner

##### Section of Paediatrics, Imperial College, London, United Kingdom

**Correspondence**: Nandinee Patel - nandinee7@gmail.com

*Clinical and Translational Allergy* 2017, **7(Suppl 1)**:PD43

**Introduction**: The prevalence of peanut allergy is around 2% in children. Current guidance from the European Medicines Agency (EMA) requires any medication containing soya-based products (including soya oil) to state that the product is contra-indicated in peanut-allergic individuals, since both are legumes. While clinical allergy to soya in peanut-allergic individuals is considered to be uncommon, only limited published data are available to substantiate this. We therefore sought to determine the rate of soya allergy in children with challenge-proven peanut allergy.

**Methods**: We performed open food challenges to soya (total 4.0 g protein) in children with peanut allergy proven through double-blind placebo-controlled food challenge (DBPCFC). All challenges were conducted according to PRACTALL consensus criteria. Where a participant experienced any symptoms during the soya challenge, DBPCFC to soya was undertaken to exclude placebo reactors. Local ethical and regulatory approval was granted, and informed consent was obtained. ClinicalTrials.gov Identifier: NCT02149719.

**Results**: 38 children (median age 13.5 years, range 8–17 years, M:F 1.3:1) with peanut allergy (confirmed by DBPCFC) participated in this study. Over half had a history of previous anaphylaxis to peanut, and 24% developed anaphylaxis during DBPCFC to peanut. Skin prick test to peanut extract ranged from 5 to 22 mm (median 10 mm), with a median peanut-specific IgE of 46 kU/L. SPT to soya extract was negative in 31/38 children; in the remaining 7 children, SPT varied from 2 to 4 mm. 37/38 (97%) tolerated the open challenge to soya; 1 child experienced oral pruritus which was reproduced during DBPCFC—interestingly, this child tolerated an open challenge to unroasted soya protein. One child developed oral symptoms to roasted but not unroasted soya; this child was also sensitised to birch pollen. There was no evidence of systemic soya allergy in this cohort of peanut-allergic children at challenge.

**Conclusion**: Co-allergy to soya is uncommon in peanut-allergic children. These data suggest that the EMA requirement for labelling of medicines containing low levels of soya as contraindicated in peanut-allergic individuals is unwarranted.

### PD44 The beta-phenotype: clinical and serological data of a new low-risk cow’s milk allergy phenotype

#### Paloma Poza-Guedes, Ruperto González-Pérez, Inmaculada Sánchez-Machín, Victor Matheu-Delgado

##### Hospital del Tórax, Santa Cruz de Tenerife, Spain

**Correspondence**: Paloma Poza-Guedes - pozagdes@hotmail.com

*Clinical and Translational Allergy* 2017, **7(Suppl 1)**:PD44

**Introduction**: Cow’s milk (CM) allergy is a rising problem worldwide. Poor prognostic risk factors such as casein sensitization are well described. Nowadays we have not yet clear clinical markers to differentiate in early stages patients with good prognosis. We analyze the clinical characteristics of patients who present only mild gastrointestinal symptoms.

**Methods**: We selected pediatric patients who refered only mild gastrointestinal symptoms after CM intake in the last 2 years. Skin prick test (SPT) with commercial extracts were performed with whole CM, casein (CAS), alphalactoalbumin (ALA) and betalactoglobulin (BLG). Measurement of total IgE and specific IgE against milk proteins in patients sera were performed (ImmunoCAP, Sweden). Families have been asked about their tolerance with both whole CM and dairy products. All patients performed an open challenge with CM to confirm clinic. Celiac disease and lactose intolerance was ruled out.

**Results**: From a total of 645 pediatric patients referring food adverse reaction, in 96 patients (4.3 ± 3 y.o.) have been confirmed mild gastrointestinal symptons after CM intake. The most prevalent symptom was abdominal cramps (n = 72), followed by abdominal bloating (47), mild chronic diarrhea (n = 38) or feed refusal (n = 21). A positive SPT to CM was achieved only in 35 patients (36.4%), with positive BLG in only 21 cases. Milk sIgE showed low rates generally, but BLG sIgE seems like the most prominent. It was noted that in a third of cases sIgE to CM was negative and in almost 25% only BLG showed positive sIgE. All patients identify CM such as the triggering food, although almost a third were still tolerating yogurt. Thus in the remaining patients we performed a subsequent open challenge test with yogurt, showing good tolerance nearly 80% of them. Interestingly only after 1 year, more than a half of patients reached whole CM tolerance. The broad dietary restriction in patients with CM allergy involves important limitations in everyday life. The identification of different clinical phenotypes may have relevant implications for the clinical management, and a potential better short-term prognosis.

**Conclusion**: We describe a new CM allergy phenotype characterized by mild abdominal symptoms and lower CM sIgE, showing BLG sIgE as a key biologic marker in our population. The identification of this syndrome may be clinically relevant in the management and prognosis of CM allergic patients.

### PD45 Mechanistic characteristics of peanut allergic children undergoing oral food challenge and oral immunotherapy

#### Erik Wambre

##### Benaroya Research Instiute, Seattle WA, USA

**Correspondence**: Erik Wambre - ewambre@benaroyaresearch.org

*Clinical and Translational Allergy* 2017, **7(Suppl 1)**:PD45

**Introduction**: Peanut OIT can dramatically improve the quality of life for allergic children but the molecular and immunological changes that occur during treatment are poorly understood.

**Methods**: Whole blood samples were collected pre- and post-oral food challenge to peanut at baseline and at 6 months from 15 peanut allergic patients participating in a double-blind, placebo-controlled, randomized trial of peanut Oral Immunotherapy (POIT). Basophil activation test (BAT) using CD203c expression was performed after stimulation with different concentration of peanut extract. Peanut-reactive CD4+ T cell response were monitored using the CD154-based assay following stimulation with pool of Ara h 1, 2, 3, 6, 8 and 9 peanut component. Sorted peanut-reactive CD4+ T cells were then run on the Fluidigm 96.96 dynamic array chip to assess changes in gene expression.

**Results**: Positive reactions to the BAT and presence of peanut-reactive effector TH2A cells lacking CD27 expression reflect the status of sensitization to peanut, proved by Oral food challenge. Oral food challenge to peanut drastically increase the frequencies of peanut-reactive CD4+ T cells before POIT but at a lower extent in the active group post treatment. Changes in the frequency, phenotype and molecular signature of peanut reactive T cells are predictive of early clinical responses induced by POIT. Our results also confirmed that allergen-specific TH2 cells exhibit an “exhausted”phenotype and are preferentially targeted by allergen immunotherapy.

**Conclusion**: These results reveal novel immunological and transcriptional signatures as surrogate markers of successful immunotherapy.

### PD46 Development of a food allergy skin sensitisation model in naive Brown Norway rats

#### Anne-Sofie Ballegaard, Charlotte Madsen, Juliane Gregersen, Katrine Lindholm Bøgh

##### National Food Institute, Technical University of Denmark, Søborg, Denmark

**Correspondence**: Katrine Lindholm Bøgh - kalb@food.dtu.dk

*Clinical and Translational Allergy* 2017, **7(Suppl 1)**:PD46

**Introduction**: Allergic sensitisation to foods may occur in infancy without prior oral exposure to the offending food. This has led to the assumption that food allergy sensitisation may occur through alternative routes, such as via the skin. Recently, concerns have been raised regarding the safety of use of cosmetic and personal care products containing hydrolysed wheat proteins. The aim of this study was to develop a skin sensitisation model in naïve Brown Norway (BN) rats.

**Methods**: A high IgE-responder BN rat strain bred on a gluten-free diet for several generations were used as an animal model and two different products, unmodified gluten and acid hydrolysed gluten were used as model proteins. Rat abdominal skin was shaved, lightly scratched with sandpaper and exposed to one of the products. The animal model was optimised for duration of skin exposure, the amount of product applied as well as for the post-immunisation regime. Skin conditions were evaluated by histology and water evaporation. At different time points sera were collected and analysed for the level, avidity and cross-reactivity of specific antibodies by different ELISAs. The antibody specificity was evaluated by immunoblotting and the functionality was examined by an ear swelling test.

**Results**: Both products were able to induce a specific immune response and sensitise through the slightly damaged skin without any use of adjuvant. This was evident before any post-immunisations. The sensitisation response depended on the duration of the skin application as well as on the amount of products applied to the skin. Differences in dose-response relationship were seen between products. The results confirm previous studies showing that acid hydrolysis induced new epitopes while maintaining original epitopes. Antibody avidity differed greatly between the products and showed that the shared epitopes induced antibodies with highest avidity. Oral and i.p. post-immunisation induced different outcomes, with a surprisingly higher response after oral compared to i.p. post-immunisations. The aim of the study, developing an animal model for studying food allergy skin sensitisation, was fulfilled. The model was able to detect differences in the induced response between the two products and further indicated homing of skin immune cells to the gut.

**Conclusion**: In BN rats non-tolerant to gluten, unmodified and acid hydrolysed gluten has sensitising capacity through the skin.

### PD47 The nature of wheat gliadins modifies the immune response in a mice model of food allergy

#### Grégory Bouchaud^1^, Laure Castan^1,2,3,4^, Mathilde Claude^1,4^, Philippe Aubert^3,5,7^, Michel Neunlist^3,5,7^, Antoine Magnan^2,3,4,6,7^, Marie Bodinier^1^

##### ^1^INRA, UR1268 BIA, Nantes, France; ^2^INSERM, UMR1087, Institut du Thorax, Nantes, France; ^3^CNRS, UMR6291, Nantes, France; ^4^Université de Nantes, France; ^5^INSERM UMR913, Institut des Maladies de l’Appareil Digestif (IMAD), Faculté de Médecine, Nantes, France; ^6^Service de Pneumologie, Institut du Thorax, CHU de Nantes, Nantes, France; ^7^DHU2020 Médecine Personnalisée des Maladies Chroniques, Nantes, Nantes, France

**Correspondence**: Grégory Bouchaud - Gregory.bouchaud@nantes.inra.fr

*Clinical and Translational Allergy* 2017, **7(Suppl 1)**:PD47

**Introduction**: Food allergies result from a complex immune response involving both innate and adaptive immune cells. Major proteins of wheat flour, gliadins, appear as important allergens and have a special role in wheat-dependent exercise-induced anaphylaxis. Allergen characteristics can influence the allergic response. In this context, chemically modified gliadins by industrial processes impact immune mechanisms orchestrating allergic reaction in an undefined manner. Our study investigates immune reaction development during food allergy with gliadins under native, deamidated or hydrolyzed forms.

**Methods**: Mice were sensitized with native (NG), deamidated (DG) or hydrolyzed gliadin (HG). Subsequently, mice were challenged by oral gavage with the corresponding allergens. Then, organs were collected at different time points and analyzed for immune and physiological parameters such as gastro-intestinal functions, cytokine secretion or allergenspecific IgE.

**Results**: Preliminary data clearly show an increase of specific IgE and IgG1 level in serum after challenge when mice were sensitized with DG compared with NG and a level comparable to un-sensitized mice with HG. This was accompanied with an increase of intestinal permeability and histological score reflecting intestinal integrity. Moreover, a more pronounced Th2-polarization together with a decrease in regulatory immunosuppressive response was observed in lymph nodes from mice sensitized with DG compared with NG sensitized mice.

**Conclusion**: Altogether, our data tend to demonstrate that industrial processes such as deamidation or hydrolysis impact food allergenicity through immune modulation and help us to develop tools to define how they can influence this reaction and encourage or decrease allergic reactions.

### PD48 Orally administered hydrolysed ovalbumin as an immunotherapeutic agent in a mouse model of egg allergy

#### Daniel Lozano-Ojalvo, Alba Pablos-Tanarro, Leticia Pérez-Rodríguez, Elena Molina, Rosina López-Fandiño

##### Instituto de Investigación en Ciencias de la Alimentación (CIAL, CSIC-UAM), Madrid, Spain

**Correspondence**: Daniel Lozano-Ojalvo - daniel.lozano@csic.es

*Clinical and Translational Allergy* 2017, **7(Suppl 1)**:PD48

**Introduction**: At present, the main treatment for egg allergic patients is based on food avoidance, which poses a risk, since egg is used as an ingredient in a wide range of food products. Oral immunotherapy (OIT) is a promising treatment option, although the use of intact allergens produces frequent side effects. In this respect, egg white protein hydrolyzates are thought to be safer to induce protective mechanisms leading to oral tolerance. The aim of this study was to determine the immunomodulatory effects of pepsin-hydrolyzed ovalbumin (OVA) administered as OIT in a BALB/c model of egg allergy.

**Methods**: BALB/c mice were orally sensitized during 6 weeks with 5 mg of raw egg white (EW) using cholera toxin as adjuvant. On week 7, mice underwent an immunotherapy protocol with either intact or pepsin-hydrolyzed OVA during 3 weeks and were subsequently challenged with EW. The severity of the anaphylactic response was evaluated (clinical signs and body temperature drop) and serum levels of *m*MCP-1 were determined by ELISA. Allergen-specific antibodies, IgE and IgG1, were monitored throughout the OIT. The expression of the genes TSLP, IL-33, IL-25, TGF-β and IL-10 was analyzed by RT-qPCR in the small intestine. Furthermore, cytokine responses were measured in allergen-stimulated splenocytes and changes in cellular populations (Th1, Th2 and T reg) were assessed in the mesenteric lymph nodes (MLNs) using flow cytometry.

**Results**: Mice orally treated with pepsin-hydrolyzed OVA were significantly protected from anaphylactic reactions compared with the groups of untreated mice and mice treated with intact OVA, which showed anaphylactic signs and a marked decrease of body temperature. Similarly, serum levels of *m*MCP-1 were lower in mice treated with the hydrolyzate. Desensitization of the allergic mice induced by the hydrolyzate was accompanied by a significant reduction in the levels of EW-specific IgE and IgG1. Administration of hydrolyzed OVA also downregulated the intestinal expression of TSLP, IL-33 and IL-25, and led to higher levels of IL-10 expression. However, the group that received intact OVA showed similar expression levels than untreated control mice. Desensitization by pepsin-hydrolyzed OVA was associated with a shift in the Th2 profile, as shown in *ex vivo* stimulated splenocytes and flow cytometry analysis of T cell subsets in the MLNs.

**Conclusion**: OIT with pepsin-hydrolyzed OVA desensitizes and prevents allergen-induced anaphylaxis in mice allergic to EW more effectively than the intact protein.

### PD49 Long term reduction in food allergy susceptibility in mice by combining breastfeeding-induced tolerance and TGF-β enriched formula after weaning

#### Akila Rekima^1^, Patricia Macchiaverni^2^, Mathilde Turfkruyer^1^, Sebastien Holvoet^3^, Lénaïck Dupuis^3^, Nour Baiz^4^, Isabella Annesi-Maesano^4^, Annick Mercenier^3^, Sophie Nutten^3^, Valérie Verhasselt^1,5^

##### ^1^University of Nice Sophia Antipolis, TIM, EA 6302, Nice, France; ^2^Institute of Biomedical Sciences - University of São Paulo, São Paulo, Brazil; ^3^Nestle Research Center, Lausanne, Switzerland; ^4^Sorbonne Universités, UPMC Univ Paris 06, INSERM, Institut Pierre Louis d’Epidémiologie et de Santé Publique (IPLESP UMRS 1136), Epidemiology of Allergic and Respiratory Diseases Department (EPAR), Medical School Saint-Antoine, Paris, France; ^5^The International Inflammation “in-FLAME” Network, Worldwide Universities Network

**Correspondence**: Akila Rekima - akila.rekima@unice.fr

*Clinical and Translational Allergy* 2017, **7(Suppl 1)**:PD49

**Introduction**: Inconsistencies in findings on food allergy prevention by breastfeeding may result from variations in duration of breastfeeding induced protection. Here, we assessed in mice how long food allergy was prevented by breastfeeding induced oral tolerance, and whether oral TGF-β supplementation after weaning would prolong it.

**Methods**: We first quantified ovalbumin (OVA) and OVA specific immunoglobulins levels (ELISA) in milk from the French EDEN birth cohort. Since OVA specific Ig were found in all milk samples, we assessed whether OVA-immunized mice exposed to OVA during lactation could prevent allergic diarrhea in their 6 and 13-week-old progeny. In some experiments, a supplement of TGF-β enriched formula was given after weaning.

**Results**: We found that, at 6 weeks, only 17% of symptom scores were ≥ 3 (diarrhea) during the last 3 oral OVA challenges in the group of mice breastfed by mothers immunized to OVA and exposed to OVA during lactation versus 43% in the group of mice breastfed by naïve mothers. However, at 13 weeks, the percentage of diarrhea increased to 28%. Supplementation with TGF-β after weaning allowed maintaining a strong protection from allergic diarrhea in 13-week-old mice breastfed by OVA-exposed mothers (13% of diarrhea only). This prolonged protection was only observed in mice rendered tolerant by breastfeeding and was associated with an improved gut barrier.

**Conclusions**: In mice, prevention of food allergy by egg antigen exposure through breast milk is of limited duration. Nutritional intervention by TGF-β supplementation after weaning could prolong beneficial effects of breast milk on food allergy.

### PD51 Cross-talk between Tregs and NKT cells in children with food allergy

#### Ines Mrakovcic-Sutic^1^, Srdan Banac^2^, Ivana Sutic^3^, Zdenka Baricev-Novakovic^3^, Ingrid Sutic^4^, Valentino Pavisic^1^

##### ^1^Department of Physiology and Immunology, Medical Faculty, Rijeka, Croatia; ^2^Department of Pediatrics, Medical Faculty, Rijeka, Croatia; ^3^Department of Public Health, Medical Faculty, University of Rijeka, Rijeka, Croatia; ^4^Medical Faculty, University of Rijeka, Rijeka, Croatia

**Correspondence**: Ines Mrakovcic-Sutic - ines.mrakovcic.sutic@medri.uniri.hr

*Clinical and Translational Allergy* 2017, **7(Suppl 1)**:PD51

**Introduction**: Understanding the mechanisms how the host recognizes countless foreign antigens and remains unresponsive to self, have opened many questions in the field of immunological tolerance. The most specific marker that distinguishes regulatory from conventional T cells is forkhead box transcription factor (Foxp 3). Predominant cell types that expressed Foxp 3 are CD4+CD25+ and characteristic of subpopulations which are Foxp 3 negative are their regulatory function lacked. Natural Tregs have the possibility to suppress multiple cell types involved in immunity and inflammation by inhibition the proliferation, immunoglobulin production, the blocking of NK and NKT-cell cytotoxicity, as well as, the function and maturation of dendritic cells. It seems that the efficacy of Treg-based therapy depends on the antigen specificity of these regulatory T cells. The most common use of Tregs is described in the prevention of allergic diseases, autoimmunity and in possibility to moderate transplantation tolerance. NKT cells represent a unique sublineage of innate lymphocytes, which share the properties of natural killer cells and conventional T cells. Hypothesis about regulatory network of Tregs and NKT cells was studied by flow cytometry, analyzing the characteristics of human Tregs and NKT cells of patients with food allergy, compared to healthy volunteers.

**Methods**: A total of 30 children with food allergy were investigated. None of them was taking any systemically administered medications for at least 3 months before testing. Venous blood samples were taken and peripheral blood leukocytes (PBL) were isolated. Phenotype of lymphocytes was analyzed using intracellular and surface immunofluorescence and flow cytometric analysis (FACSCalibur).

**Results**: Our preliminary study was shown a significant increased in a percentage of regulatory T lymphocytes and NKT cells in peripheral blood of people with described food allergy. Tregs have the ability to suppress allergic immune response. NKT cells perform an important subpopulation of cells which can play both roles: as effectors and as regulatory cells in a wide range of disease settings.

**Conclusion**: We can talk about the new kind of cells NKT-reg cells, whose monitoring may lead to important early diagnosis and/or prognosis of food allergies.

**Acknowledgements**: This work was supported by grants from University of Rijeka (13.06.1.1.14 and 13.06.1.1.15).

### PD52 PGE2 diminishes basophil activation in patients with food anaphylaxis dependent of nonsteroidal anti-inflammatory drugs

#### Mariona Pascal^1^, Rosa Muñoz-Cano^1^, Teodoríkez Jiménez-Rodríguez^2^, Daniel Corbacho^1^, Jordi Roca-Ferrer^1^, Joan Bartra^1^

##### ^1^Hospital Clinic, IDIBAPS-Universitat de Barcelona, Barcelona, Spain; ^2^Hospital General Universitario de Alicante, Alicante, Spain

**Correspondence**: Mariona Pascal - mpascal@clinic.ub.es

*Clinical and Translational Allergy* 2017, **7(Suppl 1)**:PD52

**Introduction**: A direct effect of nonsteroidal anti-inflammatory drugs (NSAIDs), but not selective cyclooxygenase (COX)-2 inhibitors, on basophils using a human model of peach lipid transfer protein (Pru p 3) allergy enhanced by NSAIDs has been recently reported. In patients with food dependent exercise induced anaphylaxis, the administration of misoprostol, a prostaglandin (PG) E1 analog, was reported to inhibit the allergic response. We sought to evaluate the effect of PGE2 in an in vitro model of basophils of patients with Pru p 3 allergy enhanced by NSAIDs.

**Methods**: A basophil activation test, measured as expression of CD63 (Flow2CAST™, Bühlmann Laboratories AG^®^, Switzerland) is performed strictly following the manufacturer’s procedure. Stimuli used are: Pru p 3 (1.120 ng/mL), anti-IgE, lysine acetylsalicylate (L-ASA; 3.38 mM) and PGE2 (10–4 M) at several concentrations and in combinations.

**Results**: A BAT to 6 consecutive patients with Pru p 3 allergy enhanced by NSAIDs has been performed. In all of them, we observe the enhancement of basophil activation caused by the L-ASA in combination with the allergen compared to the activation caused by the allergen alone, as previously described by our group. PGE2 not only diminishes the effect of L-ASA on basophil activation (median CD63+ basophils (%) after stimuli Pru p 3+L-ASA vs Pru p 3+L-ASA+PGE2: 60.25 vs 44.60, p = 0.031) but also the activation caused by the antigen alone although not statistically significant (median CD63+ basophils (%) after stimuli Pru p 3 vs Pru p 3+PGE2: 58.2 vs 37.3, p = 0.063). In 4 of these patients the same experiment was done but using anti-IgE instead of Pru p 3 and the same effect of PGE2 was observed (median CD63+ basophils (%) after stimuli anti-IgE+L-ASA vs anti-IgE+L-ASA+PGE2: 83.7 vs 62.3, p = 0.016; anti-IgE vs anti-IgE+PGE2: 76.3 vs 58.2, p > 0.05).

**Conclusion**: Preliminary results show that PGE2 diminishes basophil activation in vitro in our patients with food anaphylaxis dependent of NSAIDs. Confirmation of this observation is required with a larger cohort of patients.

### PD53 Heterogeneity of specific CD4^+^ T cell responses to peanut allergic components

#### Erik Wambre

##### Benaroya Research Institute, Seattle WA, USA

**Correspondence**: Erik Wambre - ewambre@benaroyaresearch.org

*Clinical and Translational Allergy* 2017, **7(Suppl 1)**:PD53

**Introduction**: We recently described a unique Th2 cell subset specifically involved in all allergy disease (TH2A cell subset). We investigate the cellular and molecular mechanisms behind clinical heterogeneity of peanut allergic responses.

**Methods**: We combined a CD154-based assay and a single-cell transcriptomes analysis to assess ex vivo and at a single cell level the specific CD4^+^ T cell responses to each peanut allergic components (Ara h) in adults with or without peanut allergy.

**Results**: Pathogenic responses (Th2 response) were specifically associated with short life terminally differentiated allergen-specific CD4^+^ T cells, which dominate in allergic subjects but are absent in non-allergic subjects. Protective responses in non-atopic individuals were associated with peanut-specific TH1/TH17 cell responses. Within the peanut allergic group, we observed inter-individual variations of the specific immune response to each peanut allergic component. No direct linkage between CD4^+^ T cell response and IgE responses against each individual peanut allergic component.

**Conclusions**: Ability to identify immunogenicity and type of response elicited by each peanut allergic component appears to be critical to future success in vaccine development against peanut allergy. Understanding the type of cellular response and the role of genetic restriction may allow to target immune response to critical peanut allergen and to uncover the optimal type of cellular immune response necessary for protection.

### PD54 The role of enzymes matrix metalloproteinases 2 and 9 in the pathogenesis of food allergies

#### Ines Mrakovcic-Sutic^1^, Srdan Banac^2^, Valentino Pavisic^1^, Aleksandar Bulog^3^, Ivana Sutic^4^, Vladimir Micovic^3^, Ingrid Sutic^5^, Zdenka Baricev-Novakovic^4^

##### ^1^Department of Physiology and Immunology, Medical Faculty, Rijeka, Croatia; ^2^Department of Pediatrics, Medical Faculty, Rijeka, Croatia; ^3^Department of Public Health, Medical Faculty, University of Rijeka, Rijeka, Croatia; ^4^Department of Family Medicine, Medical Faculty, University of Rijeka, Rijeka, Croatia; ^5^Medical Faculty, University of Rijeka, Rijeka, Croatia

**Correspondence**: Ines Mrakovcic-Sutic - ines.mrakovcic.sutic@medri.uniri.hr

*Clinical and Translational Allergy* 2017, **7(Suppl 1)**:PD54

**Introduction**: Interactions between immune and inflammatory responses may play a crucial role in the development and progression of allergic diseases, autoimmune and chronic progressive inflammatory disease. The matrix metalloproteinases (MMPs) play a key role in angiogenesis together with migration and/or invasion of endothelial cells in surrounding stroma and tissues. MMPs are involved in degrading of extracellular matrix (ECM), which consequently lead to facilitate invading of endothelial cells and in the same time stimulate the releasing of extracellular matrix-sequestered proangiogenic factors (ECM-sequestered proangiogenic factors), integrins, adhesion receptors and different growth factors and receptors. The members of the matrix metalloproteinase (MMP) family are involved in angiogenesis and vascular remodeling, consequently leading to the progression of numerous vascular diseases such as atherosclerosis, varicose veins, hypertension, abdominal aortic aneurysm, preeclampsia, etc. The aim of this study was to examine the values of enzyme matrix metalloproteinase-2 and 9 in urine from children with described food allergies.

**Methods**: We analyzed 30 patients with children with described food allergies. The method of enzyme immunoassay (ELISA) was used to determine enzymes expression of matrix metalloproteinase-2 and 9 (MMP-2 and 9).

**Results**: The children with described food allergies had a statistically significantly increased level of MMP-2 and 9 in the urine in comparison with healthy volunteers.

Matrix metalloproteinases (MMPs) play a key role in the physiology of connective tissue development, in morphogenesis and in wound healing and their unregulated activity has been implicated in numerous disease processes including arthritis, tumor cell metastasis and atherosclerosis.

**Conclusion**: Our data has showed a large increase in the enzyme MMP-2 and 9 in the urine of children with described food allergies, which may be an easy marker for the monitoring of the development of food allergies in children.

**Acknowledgements**: This work was supported by grants from University of Rijeka (13.06.1.1.14 and 13.06.1.1.15).

### PD55 *Lactobacillus casei* and *Lactobacillus delbrueckii* ssp. *Bulgaricus* as sources of IgG and IgE-reactive proteins

#### Lidia Markiewicz, Agata Szymkiewicz, Anna Szyc, Barbara Wróblewska

##### Institute of Animal Reproduction and Food Research of Polish Academy of Sciences, Olsztyn, Poland

**Correspondence**: Lidia Markiewicz - l.markiewicz@pan.olsztyn.pl

*Clinical and Translational Allergy* 2017, **7(Suppl 1)**:PD55

**Introduction**: Secretion of antibodies belonging to IgG, IgA and IgM class is a natural response of host organism to any protein antigens including those introduced with food. Production of IgE may, however, indicate on sensitivity of the immune system to a particular protein. Bacteria present in food continuously influence the host body interacting with the immune system. Last reports indicate on possible adverse, IgE-dependent reaction of human immune system with proteins from lactic acid bacteria (LAB), namely *Lactobacillus casei*. The aim of the study was to investigate whether proteins from LAB species commonly used in food industry react with human both IgG and IgE antibodies.

**Methods**: Whole cell extracts of two LAB strains *Lactobacillus delbrueckii* subsp. *bulgaricus* 151 (L151) and *Lactobacillus casei* LcY (LcY) were a source of proteins. Two pooled human sera were used as a source of human primary antibodies: serum A obtained by pooling seven sera from allergic patients and serum B obtained by pooling sera from ten allergic patients. As a control, a pool of sera from six healthy participants with negative serum parameters for allergy diagnostic tests, with no allergic manifestations and with defined negative family histories of atopy was used. Bacterial proteins were separated (Tricine SDS-PAGE), then transferred onto nitrocellulose membranes. The membranes were probed with human sera (primary antibodies) and then with fluorescently labelled secondary antibodies: goat anti-human IgG antibodies already conjugated with IRDye^®^ 800CW (Li-COR) and mouse monoclonal anti-human IgE antibodies (Sigma) labelled using the IRDye^®^680RD Protein Labelling Kit (Li-COR). Signal detection was carried out with the Odyssey Infrared Imaging System.

**Results**: Analyses carried out with pooled serum A showed only one protein fraction (*ca.* 36 kDa) in LcY strain reacting with both IgG and IgE. With the use of pooled serum B, however, IgG and IgE reactive proteins were detected in both LcY and L151 strain. Differences in profiles of immunoreactive proteins obtained with the examined sera indicated that bacterial proteins are characterized by different immunological determinants with different affinity to human antibodies.

**Conclusions**: Immunoreactivity of bacterial proteins, which may be in contact with the human immune system, and studies on immunoreactivity of proteins from bacteria used in food production technology and those comprising the human microbiome should be continued.

### PD57 Mineral status of infants requiring dietary management of cow’s milk allergy by using an amino acid-based formula

#### Bryan M. Harvey^1^, Lucien F. Harthoorn^2^, A. Wesley Burks^3^

##### ^1^Children’s Investigational Research Program, LLC (CHIRP), Bentonville AR, USA; ^2^Nutricia Research, Nutricia Advanced Medical Nutrition, Utrecht, the Netherlands; ^3^University of North Carolina, Chapel Hill NC, USA

**Correspondence**: Lucien F. Harthoorn - lucien.harthoorn@nutricia.com

*Clinical and Translational Allergy* 2017, **7(Suppl 1)**:PD57

**Introduction**: Cow’s milk allergy (CMA) is the most common food allergy in infancy. Fundamental to the management of food allergy is complete elimination of the offending proteins. However, due to dietary elimination CMA patients are at risk for inadequate nutritional intake. Management approaches in infants and young children include the use of hypoallergenic formulas that need to be fully tolerated, support normal growth and also assure adequate nutritional status in these patients. Dietary management of CMA with a hypoallergenic amino acid-based formula (AAF) has been proven to be effective and safe. Data on mineral status after dietary management by AAF are however scarce.

**Methods**: In a prospective, randomized, double-blind controlled study, full term infants with diagnosed CMA received an AAF (n = 110) with or without synbiotics (neutral and acidic oligosaccharides, *Bifidobacterium breve* M-16 V) for 16 weeks. Primary outcomes were growth and formula tolerance and have been reported previously [1,2]. Mineral status was assessed by analyses of blood samples obtained at baseline and 16 weeks, which included calcium, phosphorus, chloride, sodium, potassium, magnesium and total iron. Total protein, albumin, prealbumin, hemoglobin and ferritin were also determined. Formula intake was recorded through diaries at weeks 0, 4, 8 and 16 during the study.

**Results**: Average age of infants at inclusion was 4.5 ± 2.4 months (mean ± SD). Median study product intake ranged from 704 ml/day in the first week to 789 ml/day at week 16. At baseline, averages (mean, median) of blood levels of calcium, phosphorus, chloride, sodium, potassium, magnesium and iron were within reference ranges. After 16 weeks on AAF, the averages of all mineral blood levels were again within the specified reference ranges set for the corresponding infant ages. Also the averages of total protein, albumin, prealbumin, hemoglobin and ferritin were within reference ranges. For some minerals, a number of individual values at baseline were below references, i.e. calcium (n = 1), phosphorus (n = 1), chloride (n = 1), and sodium (n = 1), whereas at week 16 none of these minerals had individual values below reference ranges.

**Conclusion**: This study shows that an AAF with or without synbiotics, which have been reported previously to be equally tolerated and to support normal growth [1,2], are effective in managing an adequate mineral status in CMA infants.

**References**

1. Harvey BM, Langford JE, Harthoorn LF, Gillman SA, Green TD, Schwartz RH, Burks AW. Effects on growth and tolerance and hypoallergenicity of an amino acid-based formula with synbiotics. Pediatr Res. 2014;75(2):343–51.
2. Burks AW, Hathoorn LF, Van Ampting MT, et. al. Synbiotics-supplemented amino acid-based formula supports adequate growth in cow’s milk allergic infants. Pediatr Allergy Immunol. 2015; 26:316–22.

## POSTER DISCUSSION SESSION 4: Case reports

### PD58 Desensitisation of serious milk allergy in adult patient and monitoring with basophil activation test and IgG4

#### Georgios Rentzos^1,2^, Anna-Lena Bramstång Björk^1^, Ulf Bengtsson^2^

##### ^1^Section of Allergology, NÄL Hospital, Trollhättan, Sweden; ^2^Intitute for Medicine, Sahlgrenska Academy, University of Gothenburg, Gothenburg, Sweden

**Correspondence**: Georgios Rentzos - grentzos@gmail.com

*Clinical and Translational Allergy* 2017, **7(Suppl 1)**:PD58

**Introduction**: Serious life-threatening food allergy is still difficult to treat and usually results in a social life with restrictions. Pre-treatment with Anti-IgE has lately been suggested in order to start desensitization for food allergy in some patient cases. The conventional allergy tests though, are not always enough for monitoring the treatment with Anti-IgE and desensitization process. Here, we present a case report of desensitization against serious allergy to milk and monitoring with basophil activation test (BAT) and IgG4.

**Case report**: A 70-years old female patient with serious allergy to milk, allergic asthma who was previously treated with immunotherapy for allergy to pollen and animal dander, was referred for allergological assessment and for possible treatment with desensitization to milk allergy. The patient reacted previously with anaphylaxis due to ingestion of traces of milk protein, even due to airborne milk protein and with contact urticaria due to milk. In 2015 the patient started pre-treatment with Anti-IgE, in a dose of 450 mg every 2 weeks, and was monitored with BAT before eventually desensitization. Before the treatment with Anti-IgE, BAT with prick-test extract for milk (PT) (Soluprick, ALK) was 1.148 units (U) and with fresh milk 467.29 U. After six months of treatment with Anti-IgE, BAT analysis was found very low, compared to the values before starting pre-treatment with Anti-IgE (BAT with PT-extract for milk at 1.5U and with fresh milk at 1.8 U), indicating a starting point for desensitization against milk allergy. In addition, sIgE tests showed for milk 580 kU/L, IgG4 for casein 1.55 kU/L after six-months of treatment with Anti-IgE and before starting the desensitization. The patient started desensitization with slow build-up by ingesting fresh milk once a week with target of reaching the cumulative dose of 200 ml after 16 weeks. After 12 weeks of desensitization the patient reacted with mild skin redness in the face and neck when she ingested the dose of 50 ml of fresh milk, and therefore the patient started continuous treatment with H1-antagonstist and anti-leucotrienes daily along with Anti-IgE treatment. New BAT analyses with PT-extract and fresh milk was turned out negative after 12 weeks of ongoing desensitization while IgE for milk showed 390 kU/L and IgG4 for casein 2.51 kU/L. The patient reached the dose of 200 ml fresh milk after 16 weeks and continued consuming daily dairy products without any further reactions by now.

**Conclusion**: In some patients with serious life-threatening food allergy, pre-treatment with Anti-IgE is required and BAT is suggested as monitoring tool for indicating the starting-point for the desensitization process. BAT along with IgG4 may be used in order to monitor successfully the patient’s acquired tolerance for the food allergen during the desensitization and during the follow-up later on, even when Anti-IgE medication would have been suspended.

**Consent to publish**: The patient has given consent for presentation and publication of the case.

### PD60 A novel combination of an IgE mediated adult onset food allergy and a suspected mast cell activation syndrome presenting as anaphylaxis

#### Colin Barber, Chrystyna Kalicinsky

##### Department of Internal Medicine, Section of Clinical Immunology and Allergy, University of Manitoba, Winnipeg MB, Canada

**Correspondence**: Colin Barber - umbarbec@myumanitoba.ca

*Clinical and Translational Allergy* 2017, **7(Suppl 1)**:PD60

**Introduction**: Food allergy is reported to affect approximately 2% of the adult population, with the majority having had onset during childhood. There is an increasing recognition of adult onset food allergy, however these typically are associated with pollen-plant association, and are more often food- dependent, exercise-induced, anaphylaxis (FDEIAn). According to recent consensus statements, mast cell activation syndromes are divided into 3 subtypes: Primary Mast Cell Activation Syndromes (MCAS), Secondary MCAS, and idiopathic MCAS. A mast cell activation disorder requires clinical symptomatology that is in keeping with the disorder, a transient, measurable increase in either serum tryptase or other markers of mast cell mediators and a response to agents that interfere with mast cell mediators. Recent attempts have been made to standardize an approach to suspected mast cell disorders, in order to appropriately classify individuals with evidence of mast cell activation that did not meet diagnostic criteria for systemic mastocytosis.The combination of adult onset food allergy with an underlying mast cell disorder has not been described previously in the literature, and this case report demonstrates the investigation of an elderly gentleman who presented with first onset anaphylaxis due to food ingestion with evidence of a suspected underlying mast cell activation syndrome.

**Case report**: A 75-year-old male presented to the local emergency department exhibiting symptoms consistent with anaphylaxis. When found by his son in law he was reported to be flushed and unresponsive. On arrival of emergency medical personnel he was found to be hypotensive with blood pressure values of 80/60, hypothermic at 34.7°C orally, and hypoxemic with a sPO2 of 88% by pulse oximetry. He received a 500 cc IV normal saline bolus with EMS. In the emergency department a blood pressure of 71/44 was recorded. He was treated with 3L of IV crystalloid, epinephrine 1:1000 0.3 mg IM once, diphenhydramine 25 mg IV with a second 50 mg IV dose, ranitidine 150 mg IV once, and methylprednisolone 250 mg IV once with improvement. He suffered a type 2 MI related to anaphylaxis associated hypotension that was manifested by tropinemia.

He had no previous history of anaphylaxis, atopy, lymphoproliferative disorder or other neoplasm. His daily medications included aspirin 81 mg daily, and this in addition to his other regular medications were continued post reaction. He did not take additional doses of aspirin, over the counter or herbal products on the day of reaction. There was no family history of atopy. The Allergy and Clinical Immunology Service was contacted by the emergency physician, at which time the food intake history was unclear, and given his profound hypotension at presentation a concern of a MCAS was raised. This is consistent with suggestions to investigate for MCAS in patient’s presenting with anaphylaxis with profound cardiovascular derangement and lacking documented uritcaria, even if likely attributed to an IgE mediated reaction. He was therefore discharged on cetirizine 10 mg daily, prednisone 50 mg orally for five days, diphenhydramine 25–50 mg orally q6 h as needed, and an epinephrine auto-injector. He was subsequently followed in the Adult Allergy and Clinical Immunology outpatient clinic.

Between the anaphylactic episode and his appointment at the Allergy Clinic [approximate time 1 month] he consumed Atlantic cod without reaction. A food history revealed his initial event had developed 3 h following ingestion of a mixed fish and shellfish stew, which he had consumed without reaction on a regular basis. Due to the concern of a potential underlying MCAS and the severity of his initial reaction, it was felt safest to continue H1 receptor antagonist therapy, as such skin prick testing was deferred for use of ImmunoCAP^Ⓡ^ [serum specific IgE (Phadia, Sweden)] for shellfish, finned fish and hymenoptera venom. A serum tryptase was evaluated. He was given an epinephrine auto-injector to be used in the event of subsequent anaphylactic reaction. At follow up his serum specific IgE was high positive for Shrimp at 13.7 kUA/L, and Crab at 7.3 kUA/L, moderately positive for Lobster at 2.9 kUA/L and Clam at 0.9 kUA/L, while testing negative with values <0.35 kUA/L for Salmon, Walleye Pike and Whitefish. A serum tryptase level was 15 ng/ml [with a normal range from 1 to 11.4 ng/ml], an elevation not diagnostic of systemic mastocytosis, but suggestive of a mast cell activation disorder. A creatinine obtained demonstrated a value of 92, corresponding to an eGFR of >60 ml/min, ruling out reduced renal clearance as a cause of accumulation of serum tryptase.

To further evaluate for a potential mast cell disorder, a 24 h urine methylhistamine level was obtained, demonstrating a value of 103 ug/g Cr (normal range 30–200 ug/g Cr). A serum protein electrophoresis demonstrated no evidence of an M protein. He was seen by the Adult Hematology/Oncology Service at CancerCare Manitoba to definitively exclude a diagnosis of systemic mastocytosis, for which a bone marrow biopsy and c-KIT testing were completed. His c-KIT was negative. A bone marrow biopsy demonstrated normal trilineage hematopoiesis with normal differentiation and maturation without definitive morphological evidence of mastocytosis or lymphoma, specifically revealing no large lymphoid aggregates, abnormal plasma cells, or spindle cells suggestive of mastocytosis. Accompanying flow cytometry demonstrated revealed a sample composed of 23% lymphocytes, of which 84% were T cells, 8% NK cells, and there was a CD4/8 ratio of 0.9. Remaining cells were polyclonal B cells without evidence of lymphoma, plasma cell neoplasm, or mastocytosis. Tryptase was consistently elevated at 17 ng /ml on repeat testing.

Given his elevated tryptase, he was maintained indefinitely on cetirizine, and continued to avoid both fish and shellfish, but did require emergency department monitoring following administration of his epinephrine auto injector in January 2015 following ingestion of a perogy, of which the precise constituents were unknown, and development of a diffuse urticarial rash. He was treated with a 3 day course of 50 mg of oral prednisone.

He fulfills the proposed diagnostic criteria for diagnosis of a suspected MCAS based on guidelines published by Valent and colleagues, however we acknowledge the challenge of establishing the diagnosis in the context of a documented IgE mediated food allergy, and he may be best classified as a Secondary MCAS [IgE-dependent disease related].

**Conclusions**: The combination of food allergy with a mast cell activation syndrome with onset in the elderly population represents a novel combination that is not well described. It is unclear whether the patient in this case had an underlying mast cell disorder that was previously quiescent and was detected only due to his presentation, or whether this was related to a newly acquired population of non-clonal mast cell hyperplasia secondary to an as yet undiscovered or subclinical triggering source such as malignancy, infection, subclinical thrombosis, an underlying autoimmune condition, increased stimulatory cytokines, or increased vasoactive peptides; as all are postulated triggers of mast cell activation.

The increasing prevalence of food allergy worldwide, and the heightened propensity for life threatening anaphylactic reactions in those with underlying mast cell reactivity highlights the importance of an educated, stepwise, evidence -based diagnostic approach to older adults presenting with anaphylaxis. This further supports the importance of a high level of suspicion for clonal mast cell disorders in patient’s presenting with anaphylaxis with profound cardiovascular manifestations and limited cutaneous manifestations. A case such as this emphasizes the importance of an approach with thorough investigation of all potential allergen exposures, including those to which a patient has been exposed on multiple occasions without systemic or local reaction.

It could be theorized that new sensitization to previously tolerated food antigens with resultant anaphylaxis could be associated with underlying mast cell activation disorders in adults without a history of atopy, particularly when presenting with profound hypotension. Further research is needed into the incidence of adult onset IgE mediated food allergies, anaphylaxis, and the rate at which it is associated with underlying mast cell activation, particularly given the association with MCAS and a heightened risk for a more severe anaphylactic reaction.

**Consent to publish**: The individual described in the above case report has completed and signed a consent form for publication and presentation, currently stored at the Allergy and Immunology clinical offices in the Health Sciences Centre, Winnipeg, Manitoba, Canada. A copy can be made available if required.

### PD61 A natural red pigment as a hidden allergen in delayed idiopathic anaphylaxis: carmine-induced food allergy

#### Christine Breynaert, Lieve Coorevits, Cornelia Jansen, Erna Van Hoeyveld, Kristin Verbeke, Anne-Marie Kochuyt, Rik Schrijvers

##### University Hospitals Leuven, Leuven, Belgium

**Correspondence**: Christine Breynaert - christine.breynaert@uzleuven.be

*Clinical and Translational Allergy* 2017, **7(Suppl 1)**:PD61

**Introduction**: Carmine (E120) is a red dye extracted from dried female cochineal arthropods (Dactylopius coccus cacti) commonly used in foods, drugs and cosmetics. Reports of IgE-mediated carmine allergy are limited.

**Fig. 3:**
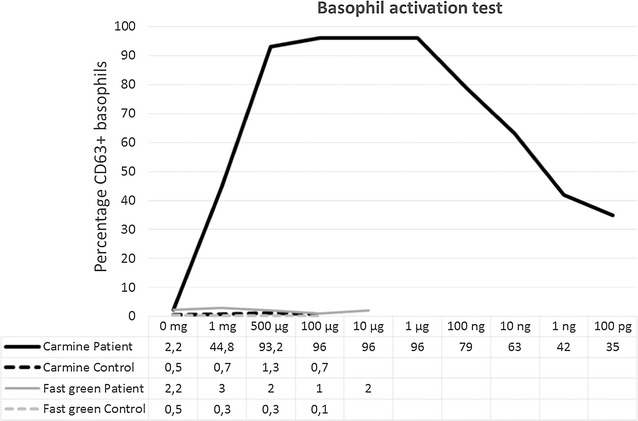
Basophil activation test

**Case report**: A 56-year-old female experienced 2 anaphylactic reactions after eating chicken tikka masala and after a croissant with wild berries topping. She showed immediate nausea, vomiting and diarrhea followed by urticarial rash and facial edema several hours later. Afterwards, patient had, with exception of the wild berries topping, the same food again but without complaints. Skin and immunoCAP (Thermofisher-Phadia) testing were negative with all food allergens relevant to the history, including Tri a19 and galactose-alpha-1.3-galactose. Idiopathic anaphylaxis was diagnosed and an adrenalin auto-injector was prescribed. Several months later, after performing an urea breath test for Helicobacter pylori with a Fortimel^®^ energy strawberry (Nutricia) drink, which contains as potential allergens milk, rape seed oil, sunflower seed oil, soy lecithin and carmine acid, patient experienced immediate epigastric pain followed by dyspnea, facial edema and generalized urticaria. Skin testing with Fortimel^®^ energy strawberry was clearly positive (negative in 5 controls) and negative for milk and soy milk. Specific IgE showed positivity for cochineal extract (2.77 kU/L) (retrospective serum analysis one month before: IgE 1.92 kU/L). Flow cytometric analysis of activated peripheral blood basophils (CD63^+^CD123 ^+^HLA-DR^−^), including a positive control (anti-IgE), a negative control (without allergen), carmine (Sigma-Aldrich) and fast green (E143), showed clear CD63 positivity after stimulation with carmine and not with fast green (Fig. 3). This response was absent in the control patient.

**Conclusions**: A rare case of carmine-induced food allergy is described and confirmed by sIgE, skin prick test and basophil activation test. Carmine, used as a natural red dye, can cause severe allergic reactions at very low concentrations, with an uncharacteristic time delay between exposure and clinical manifestations, potentially hours later. Unclear episodes of anaphylaxis may be due to sensitization to carmine. Carmine should be included in the allergy work-up of idiopathic (food-induced) anaphylaxis as it can act as a hidden allergen.

**Consent to publish**: Informed consent of the patient is obtained.

### PD62 Oral immunotherapy to wheat in allergic asthmatic female – Case presentation

#### Diana Deleanu, Adriana Muntean

##### University of Medicine & Pharmacy Iuliu Hatieganu, Cluj-Napoca, Romania

**Correspondence**: Diana Deleanu - deleanudiana@yahoo.com

*Clinical and Translational Allergy* 2017, **7(Suppl 1)**:PD62

**Introduction**: Immunological adverse reaction to wheat may be either IgE-mediated, either T-cell-dependent. In patients with IgE -mediated allergy to wheat protein there is no specific therapym, except oral immunotherapy (OIT). There are few data regarding OIT with wheat protein in wheat allergic patients.

**Case report**: A 32 years old female with a history of allergic rhinitis of 12 years developed asthma after 4 years. The patients had a severe persistent rhinitis with a step 4 partially controlled asthma, sensitized to house dust mites and grass pollen. In the last 5 years she developed a severe chronic urticaria, and in the last 2 years her asthma worsened having frequent exacerbations. She was evaluated for the cause of urticaria and of asthma exacerbations. A wheat allergy was confirmed with skin prick test (12 mm diameter), specific IgE (18.6 kUI/l) and a double-blind placebo-controlled food challenge (DBPCFC). An elimination diet was introduced for 2 months. Her urticaria and asthma symptoms improved, but she was unsatisfied with her diet. The patient underwent wheat OIT with cooked pasta. Up-dosing phase started with 1 g pasta in the allergy office and was given at home daily for 6 days. The increasing of the dose was performed weekly in the allergy department, for 8 weeks, by double the quantity up to 120 g. The dose was maintained daily for 6 months. During maintained period, the patients did not have urticaria and asthma exacerbation. She reduced the dose of glucocorticosteroids (nasal and inhaled). At the end of the maintained period, the patient underwent DBPCFC with no symptoms. Specific IgE to wheat decreased to 3.2 kUI/l. She was allowed to eat bread and other foods containing wheat proteins, with no urticaria and no asthma exacerbation, during the 6 months followed up.

**Conclusion**: A wheat OIT was safe and produced an improvement in allergic rhinitis, asthma and urticaria female patient allergic to wheat. Wheat OIT may induce tolerance to wheat proteins.

**Consent to publish**: Our patient gave consent for presentation and publication.

### PD63 Anaphylaxis to termites’ ingestion in a 30 year-old-woman

#### Maria Konstantakopoulou, Maria Pasioti, Anastasia Papadopoulou, Anna Iliopoulou, Nikolaos Mikos, Evangelia Kompoti

##### Allergology Department, ^“^Laiko” General Hospital, Athens, Greece

**Correspondence**: Maria Konstantakopoulou - mariakonstantakopoulou@windowslive.com

*Clinical and Translational Allergy* 2017, **7(Suppl 1)**:PD63

**Introduction**: Termites are insects in the *Isoptera* suborder, *Blattodea* family, with 3106 described species. They live in worm regions, including Africa, South America, South Europe and Australia. Besides their use as a dietary supplement in non developed regions, they have become popular in western countries as delicacy.

**Case report**: A 30-year-old Caucasian woman presented at the emergency department with cough, wheezing and urticaria immediately after termites’ ingestion, accompanied by epigastric pain and dizziness 30 min later. It was the first time she consumed termites. The patient had a history of two anaphylactic reactions after honeycomb ingestion. Between the first two episodes she was consuming honey, honeycomb and propolis with no reaction, but subsequently she has banned them. “Slight discomfort” with crustaceans’ consumption was also reported. Skin prick tests to food and aeroallergens were positive to grasses, Bermuda grass, *Blatella germ, Derm. Pter., Derm. Far.,* shrimp, crab and shellfish. Additionally prick to prick tests performed with raw and boiled termites turned out positive contrary to 5 negative controls. Total IgE was slightly elevated and baseline tryptase was normal. Specific IgE antibodies for shrimp, fish, grape, latex, honey bee venom and tropomyosin were negative, unlike Bermuda grass, *Blat. germ*, *Derm. pter*. and *Derm. far*. which were positive.However our patient has neither personal or family history of atopic diseases. According to history and laboratory evaluation, we speculated that the common allergen is a minor panallergen, like a calcium-binding protein, common in pollens (Bermuda) Cyn d 7 but also in crustaceans Cra c 4, cockroach Bla g 6, mite Der f 17 and honey bee (calcium binding protein 39), although, to our knowledge, no cross reactivity between animals and plants has been described. Unfortunately, for lack of means, we couldn’t identify the common allergen. Accidental exposure in related species could also be a reason for sensitization.

**Conclusion**: This is the first case report of an anaphylactic reaction to termites’ ingestion. It is challenging how the food consumption from different cultures and ambient could increase the appearance of new food allergic reactions.

**Consent to publish**: Consent was obtained from the patient for publication of this case report.

### PD66 Anaphylaxis to banana: a pediatric case report

#### Ana Rodolfo^1^, Eunice Dias de Castro^1^, Borja Bartalomé^2^, Alice Coimbra^1^

##### ^1^Serviço de Imunoalergologia, Centro Hospitalar de São João, E.P.E., Porto, Portugal; ^2^R&D Department, Bial Aristegui, Bilbao, Spain

**Correspondence**: Alice Coimbra - aipre@hotmail.com

*Clinical and Translational Allergy* 2017, **7(Suppl 1)**:PD66

**Introduction**: Food allergy is the most common cause of anaphylaxis in children. Banana is not considered a highly allergenic food and it is usually one of the first fruits introduced in the diet of infants.

**Case report**: A 2-year-old boy had an anaphylaxis episode immediately after eating a banana. He presented with oral discomfort, facial and tongue angioedema, emesis and rash on his legs. On route to the emergency department, his aunt, an Allergist, gave instructions to administer dimethindene and betamethasone with symptom improvement. He remained in observation at the hospital and an adrenaline auto-injector, antihistamine and oral steroid were prescribed on discharge. He had eaten bananas before, but always in small quantities because he did not like bananas and he would refuse to eat them. He tolerates apple, pear, mango, pineapple, melon, clementine and vegetables. His medical history includes atopic dermatitis and the use of a helmet during six months for the correction of plagio-brachycephaly under the supervision of neurosurgery. Skin prick tests (SPT) with commercial extracts were positive for kiwi and banana; and negative for *D. pteronyssinus*, grass pollens, Pru p 3 and latex. The skin prick-prick tests with banana were also positive. Total IgE and basal trytpase were normal; specific IgE to banana 2.85 kU/L and negative to latex. Sodium dodecyl sulfate polyacrylamide gel electrophoresis (SDS-PAGE) immunoblotting with banana extract and the patient’s serum was performed and an IgE-binding band with a molecular mass of 20 kDa was detected which suggests sensitization to banana.

**Conclusion**: Although banana allergy is well established in adults, often associated with latex allergy in the latex-fruit syndrome, there are few case reports of anaphylaxis to banana in children. Banana pulp is rich in thaumatin-like protein (TLP), an allergen with 20 kDa of molecular mass; it is probably the sensitizer in this case. Curiously, SPT with kiwi extract was positive although he never ate kiwi. This may be explained by the cross-reactivity described among the TLPs.

**Consent to publish**: We hereby declare that the boy’s parents authorized the authors to publish these findings.

### PD67 Omalizumab used as treatment in lipid transfer proteins allergy

#### Kok Loong Ue^1^, Elizabeth Griffiths^1^, Stephen Till^1,2^

##### ^1^Department of Allergy, Guy’s and St Thomas’ NHS Foundation Trust, London, United Kingdom; ^2^Department of Allergy, King’s College London, London, United Kingdom

**Correspondence**: Kok Loong Ue - kokloong.ue@gstt.nhs.uk

*Clinical and Translational Allergy* 2017, **7(Suppl 1)**:PD67

**Introduction**: Omalizumab (anti-IgE antibody) is an effective treatment for asthma and chronic spontaneous urticaria, but relatively few reports exist of use for prevention of uncontrolled systemic allergic reactions in adults. We report the case of an adult patient with allergy to lipid transfer proteins (LTP) who was successfully treated with omalizumab.

**Case report**: A 34-year-old Caucasian female started to experience anaphylactic reactions to red wine in 2009, with abdominal cramps, vomiting, urticaria, lips and tongue angioedema. She subsequently experienced pharyngeal discomfort with difficulty in breathing after eating grapes. Over the next 3 years she progressed to similar reactions after eating numerous fruits and vegetables. We first became involved in her case in 2012. She underwent investigation with skin tests to a wide range of fruits/vegetables, component and ISAC testing. A diagnosis of LTP allergy was made. She was provided with an anaphylaxis management plan. She continued to have recurrent and frequent reactions despite extensive dietitian input and then antihistamine prophylaxis, with repeated use of adrenaline injector and emergency department attendances. Her dietary intake became heavily restricted and quality of life was significantly affected, both as a result of allergic reactions but further compounded by new depressive and anxiety symptoms. A decision was made to undertake a trial of subcutaneous omalizumab therapy. She underwent a positive baseline double blinded placebo controlled food challenge (DBPCFC) with raisin, developing nasal congestion and abdominal pain that was reproduced when re-challenged with the same dose (20 mg) but not placebo. Treatment was then initiated with omalizumab (300 mg once monthly), in accordance with the asthma dosage recommendation for weight and total IgE. The DBPCFC was repeated after 6 months of omalizumab and a final dose of 600 mg of raisins was fully tolerated. She has subsequently undergone multiple open oral food challenges to previous trigger foods, which have now been reintroduced into her diet. She continues to receive regular omalizumab and has not experienced a recurrence of systemic allergic reactions.

**Conclusions**: There is currently little data to confirm omalizumab use in patients with anaphylaxis to foods outside of the oral immunotherapy setting. To our knowledge this is the first reported case of omalizumab being used to successfully manage systemic allergic reactions to LTP.

**Consent to publish**: Written informed consent was obtained from the patient for publication of this abstract and any accompanying images.

### PD68 Risk factors for egg allergy in Europe: EuroPrevall birth cohort

#### Kate Grimshaw^1^, Graham Roberts^1,2,3^, Anna Selby^1^, Indre Butiene^4^, Jose Ignacio Larco^5^, Michael Clausen^6^, Ruta Dubakiene^7^, Ana Fiandor^5^, Alessandro Fiocchi^8^, Linus Grabenhenrich^9^, Nikos Papadopoulos^10,11^, Sigurveig Sigurdardottir^12^, Aline Sprikkelman^13^, Anne-Fleur Schoemaker^13^, Marek Kowalski^14^, Paraskevi Xepapadaki^10^, Thomas Keil^9^, Claire Mills^15^, Kirsten Beyer^16^

##### ^1^Experimental Sciences & Human Development in Health Academic Units, Faculty of Medicine, University of Southampton, Southampton, United Kingdom; ^2^National Institute for Health Research Respiratory Biomedical Research Unit, Southampton, United Kingdom; ^3^David Hide Asthma and Allergy Research Centre, St Mary’s Hospital, Newport, Isle of Wight, United Kingdom; ^4^Faculty of Health Sciences, Klaipeda University, Klaipeda, Lithuania; ^5^La Paz Institute for Health Research, Madrid, Spain; ^6^Children’s Hospital, Landspitali – The National University Hospital of Iceland, Reykjavik, Iceland; ^7^Faculty of Medicine, Vilnius University, Vilnius, Lithuania; ^8^Division of Allergy, Pediatric Hospital Bambino Jesu, Rome, Italy; ^9^Institute of Social Medicine, Epidemiology and Health Economics, Charité Universitätsmedizin Berlin, Berlin, Germany; ^10^Allergy Unit, 2nd Pediatric Clinic, University of Athens, Athens, Greece; ^11^Centre for Paediatrics and Child Health, Institute of Human Development, University of Manchester, Manchester, United Kingdom; ^12^Department of Immunology, Landspitali – The National University Hospital of Iceland, Reykjavik, Iceland; ^13^Department of Pediatric Respiratory Medicine and Allergy, Emma Children’s Hospital, Academic Medical Center Amsterdam, Amsterdam, the Netherlands; ^14^Department of Immunology, Rheumatology and Allergy, Medical University of Lodz, Lodz, Poland; ^15^Institute of Inflammation and Repair, Manchester Academic Health Science Centre, Manchester Institute of Biotechnology, University of Manchester, Manchester, United Kingdom; ^16^Department of Paediatric Pneumonology and Immunology, Charité Universitätsmedizin Berlin, Berlin, Germany

**Correspondence**: Graham Roberts - g.c.roberts@soton.ac.uk

*Clinical and Translational Allergy* 2017, **7(Suppl 1)**:PD68

**Introduction**: Hen’s egg is the second most common allergen with a mean incidence of allergy of 1.23% (95% CI 1.27–3.47) in the EuroPrevall birth cohort. There are little data on the risk factors for egg allergy. In this study we aimed to assess the risk factors for egg allergy in the Europrevall birth cohort with a particular focus on eczema.

**Methods**: The EuroPrevall birth cohort was established across nine European countries and children were followed up to 2 years. Questionnaires were undertaken at 12 and 24 months. Children with suspected egg allergy were invited for skin prick testing, measurement of specific IgE and double-blind, placebo-controlled challenge (DBPCFC) to hen’s egg if allergy was suspected. Each egg allergy case (positive DBPCFC or egg induced anaphylaxis) was allocated two age-matched controls. Statistical analysis was undertaken in SPSS version 22 (IBM, New York, USA) and STATA SE 13 (StataCorp, College Station, USA).

**Results**: 12,049 infants were recruited into the EuroPrevall birth cohort and 9336 (77.5%) were followed until 2 years. 86 infants had egg allergy (98% via DBPCFC) and were matched with 140 controls. Cases were evaluated at a mean age of 11 months. Infants with egg allergy were significantly more likely to have eczema than controls (76 vs 39%, p < 0.001). Cases were significantly more likely to report rhinitis (26 vs 9%, p < 0.001), to have mould in their home (15.5 vs 6.5%, p = 0.038) and to have received any skin cream, lotion or powder (87.2 vs 70.9%, p = 0.001); they were less likely to be Caucasian (83 vs 93%, p = 0.027). Factors that were independently associated with egg allergy in this analysis were current eczema (adjusted OR 24.08, 95% CI 7.41–78.22), current rhinitis (2.68, 1.02–7.04), antibiotics in the first week of life (7.71, 2.15–27.64) and male gender (2.44, 1.16–5.26). Increasing eczema severity was associated with an increasing risk of egg allergy and eczema was reported to have started an average (SE) of 3.6 (0.5) months before egg allergy was evaluated.

**Conclusions**: Eczema, rhinitis, antibiotics in the first week of life and male gender are the key risk factors for developing egg allergy. The onset of eczema is temporarily related to developing egg allergy with infants with more severe eczema being more likely to develop egg allergy. This provides potential preventative strategies for egg allergy.

## POSTER SESSION 1: Clinical and therapeutic aspects • Diagnosis and treatment • Epidemiology

### PP001 Registration of allergies in primary health care history

#### Zizi Cojocariu, Beatriz Secades Barbado, Vasti Iancu, Esozia Arroabarren, Marta Goñi Esarte, Miren Arteaga

##### Complejo Hospitalario de Navarra, Navarra, Spain

**Correspondence**: Zizi Cojocariu - zizicojocariu@hotmail.com

*Clinical and Translational Allergy* 2017, **7(Suppl 1)**:PP001

**Introduction**: The prevalence of the allergologic diseases (AD) is increasing, being an important health problem. WHO classifies the AD between the first 6 pathologies in the world. These, restrict the daily activities of the patient, their life quality and it have a high social and economic impact. Also of the correct diagnose and treatment of the AD, it’s important to do an adequate record in the patient’s history being the mistakes at this level can cause mistakes relates with drug prescription and increase the costs.

**Methods**: The aim is to evaluate the quality of the computerized record of the allergies in the medical clinic history by the physicians in the health centers of Pamplona, reviewing the clinic history of the patients and by random selection, choose 1 patient of 4, between 15/02/2016 and 14/04/2016.

**Fig. 4:**
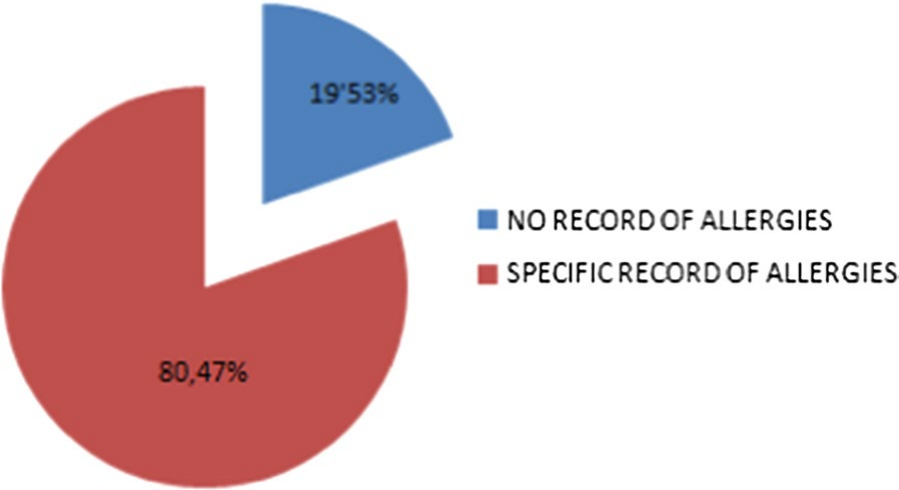
Total allergies registration

**Fig. 5:**
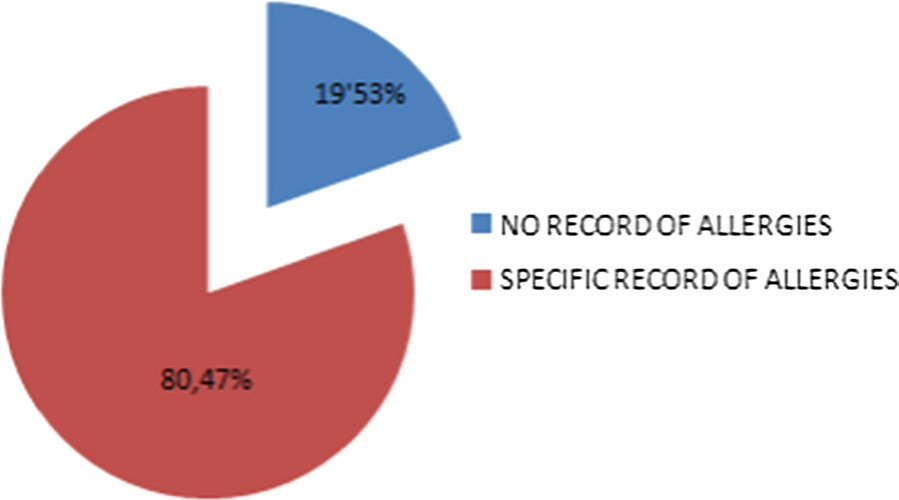
Total allergies registration

**Results**: Have been analyzed 1408 medical histories. Of these, 275 (19.5%) had no record of allergies. In 1133 (80.4%) it had a specific record of allergies, 824 (72.7%) without known allergies. In 309 patients have had recorded allergies and/or secondary effects (Fig. 4).In the analysis of the histories with positive data, in 142 (45.9%) it was diagnosed with a specific allergy (allergologic report or suggestive clinical data) and 167 (54.1%) had diagnosed with secondary known effects or they were registered improperly (recorded without any documents that support it). In these 167 medical histories, 289 records were documented (1.7 average/patient), clinical histories with more than one improperly registration, not mandatorily related between them. Among the wrong registered records (without documents, side known effects or intolerance), 69 (49.6%) were antibiotics, 36 (25.9%) NSAIDs, 34 (24.4%) other allergens (Fig. 5).

Given the clinical, social and economical importance it is a significant fact that 19.5% of the clinical histories revised there is no sign of allergy. In the ones that’s is registered, almost half of them have their record incomplete or insufficient, being the most frequent the antibiotics and NSAIDs, which condition the prescription and it can lead to a rise of the costs, having to use alternative drugs.

**Conclusion**: The interest of the physicians by allergies it seems is not the expected; a proper register of the allergies/intolerances, as well as a allergologic diagnose when it is indicated, it is fundamental for a good medical practice, quality of the prescription, higher security for the patient and decrease the costs.

### PP002 Food allergens sensitisation in atopic asthmatic patients

#### Mayra Coutinho Andrade, Denise Borges, Jorge Kalil, Pedro Giavina Bianchi, Rosana Camara Agondi

##### Serviço de Imunologia Clínica e Alergia, Hospital das Clínicas, Universidade São Paulo, São Paolo, Brazil

**Correspondence**: Mayra Coutinho Andrade - maycoutinho@hotmail.com

*Clinical and Translational Allergy* 2017, **7(Suppl 1)**:PP002

**Introduction**: Sensitization to food allergens in patients with persistent allergic asthma followed in a tertiary clinic center.

**Methods**: A cross-sectional study was conducted with asthma patients in a regular follow-up in a tertiary clinic in São Paulo, Brazil. The study included patients older than 18 years with allergic asthma, with or without suggestive history of food allergy. Sensitization to aeroallergens, and food allergens was assessed by the skin prick test and/or specific serum IgE. Food allergens included: milk, egg, wheat, soy, shrimp, fish and peanuts. Data was also researched through clinic’s registration system, total IgE levels and severity of asthma, according to Gina 2015.

**Results**: We studied 41 patients with a mean age of 39.5 years and 73% were women. When assessing the severity of asthma, 39% had severe asthma, 39% moderate, and 20% mild asthma. Fourteen percent of the patients had suggestive history of food allergy. The mean total serum IgE was 1171.8 IU/mL. Thirty-nine percent of the patients had sensitization to some of the tested food allergens. Of these, milk and eggs accounted for 25% each, followed by shrimp—20%. Wheat and peanuts accounted for 10% each of them, and soy and fish, 5% each of them. The group sensitized to food allergens had a higher total IgE level (1597 IU/mL), when compared to the group that was not sensitized to food allergens (912 IU/mL). It is well established in the literature that asthma is associated to different phenotypes, and therefore may be associated with varying degrees of treatment difficulties. It has been reported in the literature that food allergy might be a risk for complicated or poorly controlled asthma, not only in children but in adults as well, and was associated with asthma morbidity, including risk of hospitalizations and oral steroid use. Nevertheless, the prevalence of food allergy in adults with asthma is still not known. This study showed that nearly 40% of the allergic asthmatic patients with or without history of food allergy, had sensitization to major food allergens. These patients sensitized to food allergens also had higher levels of total serum IgE when compared with non-sensitized patients.

**Conclusions**: Due to the difficulty of control in some cases of asthma, different risk factors should be investigated, including food allergy. More studies should be performed to research these findings in large population. And it would have implications for the management of asthmatics, particularly those with severe disease.

### PP003 Red gram (*Cajanus cajan*) proteins induce symptoms of atopic dermatitis in BALB/c mice following intraperitoneal route of exposure

#### Rinkesh Kumar Gupta, Akanksha Sharma, Kriti Gupta, Mukul Das, Premendra Dwivedi

##### CSIR-Indian Institute of Toxicology Research, Lucknow, India

**Correspondence**: Rinkesh Kumar Gupta - rinkeshgupta9@gmail.com

*Clinical and Translational Allergy* 2017, **7(Suppl 1)**:PP003

**Introduction**: Legumes play a major role in the induction of food allergic manifestations. Allergy to red gram has been reported in allergic rhinitis as well as BALB/c mice. Information about the atopic dermatitis (AD) like symptoms induced by red gram is yet to be explored. In this study, attempts have been made to elucidate the AD like symptoms induced by red gram proteins following intraperitoneal route of exposure.

**Methods**: To establish the correlation between red gram allergy and AD, we have studied the coexistence of AD and food allergy in red gram allergic patients (n = 210) and healthy individuals (n = 10 as a control) at the Department of Respiratory Medicine, King George Medical University (KGMU), India. Further, BALB/c mice were sensitized with red gram crude protein extract (CPE) via intraperitoneal route to explore the red gram induced AD like symptoms. Serum levels of specific IgE and IgG1 antibodies were measured by indirect ELISA. Anaphylactic signs and symptoms were evaluated 40 min after the challenge by two investigators using the scoring. Histopathological responses were analyzed in the skin of treated mice. Further, mast cell count was performed in the skin. Moreover, expression levels of cytokines, transcription factors and filaggrin were studied in the skin of treated mice.

**Results**: To observe the clinical prevalence of AD in red gram sensitized patients, 210 allergic patients were screened. Out of which 3.33% patients showed possible symptoms of AD, followed by positive SPT for red gram allergen. In the Balb/c mice, elevated levels of specific IgE and IgG1 in the serum along with symptoms of anaphylaxis were found in the treated mice when compared to their respective controls. Further, histopathological analysis of skin revealed characteristics of AD in the treated groups. Significantly enhanced numbers of mast cells were observed in the skin of treated mice as compared to control. Mixed profiles of GATA-3, T-bet, IL-4 and IL-13 at the mRNA were found in the skin of treatment groups when compared to their respective controls. Similarly, enhanced expressions of GATA-3, T-bet as well as filaggrin were observed at the protein levels in the skin of treated mice. We have endeavored to understand the allergenic potential of red gram in the context of AD because in our previous study, we have observed the health concern related to skin in the pigeon pea’s allergic patients. Increased levels of specific IgE and IgG1 antibodies were evident in treated group as compared to control group; illustrate the allergenic potential of red gram CPE. In our study, treated group mice have shown significantly fall in body temperature (4–5 °C) as well as symptoms of systemic anaphylaxis upon challenge with red gram CPE. Histopathological results showed the AD like pathological changes, including epidermal thickening, mixed inflammatory cellular infiltration along with hyperplasia indicating that these changes may be evidences for AD. In this study, elevated levels of mast cells were found in the skin, suggesting the massiveness bearing of red gram specific IgEs bound to mast cell receptors. Further, treated skin also over expressed with IL-4 and IL-13 and associated transcription factors like GATA-3 and reduced expression of T-bet at mRNA level, suggesting that local skin expression of Th2 cytokines have crucial role in the development of AD. Present study showed decreased level of filaggrin in the skin, supporting our finding related to the prevalence of AD induced by red gram.

**Conclusion**: Little is known regarding the association between food allergy and atopic dermatitis. In this report, intraperitoneal exposure of red gram proteins may lead to symptoms of atopic dermatitis in BALB/c mice.

### PP004 Food allergy in young children age and the evaluation of clinical effectiveness of hypo-allergic formula

#### Rusudan Karseladze^1^, Liana Jorjoliani^1^, Lali Saginadze^2^, Mariam Tskhakaia^3^

##### ^1^Tbilisi State University, Tbilisi, Georgia; ^2^Iashvili Children’s Clinic, Tbilisi, Georgia; ^3^Institute of Paediatrics, Tbilisi, Georgia

**Correspondence**: Rusudan Karseladze - rusakars@yahoo.com

*Clinical and Translational Allergy* 2017, **7(Suppl 1)**:PP004

**Introduction**: The Atopic Dermatitis caused by nutrition allergy is noticed in most of children till one year and from the etiological spectrum a nutritious sensitization frequently is caused by cow’s milk protein.

Our aim was the evaluation of clinical effectiveness of specialized, hydrolyzed cow’s milk albumin in children of early age diseased by atopic Dermatitis (AD).

**Methods**: There were under observation 20 children from 30 day up to one year, with AD. The including criteria were age, artificial feeding and existence of AD. The excluding criteria were: an individual intolerance of formula and mother’s refusal. The basis of diagnostics was a diagnostic algorithm of AD by Hanifin and Rajka [1]. A heaviness of AD is evaluated by means of standard criteria according to universally recognized SCORAD system.

**Results**: 16 children—first group with mild form of AD, in which SCORAD index amounted to 35.5–65.5 score,14 one-second group with moderate form-SCORAD index was 65.5–78.5 score. In 52% of observed children has been diagnosed IgE-depended allergy, in 20%—an increase of specific IgG4 antibodies level towards cow’s milk albumin, but in 28% a mixed variant. Hence is follows, that for the purpose of diet-therapy, the children with AD have been taking specialized hypo-allergic formulas, which is made on basis of hydrolyzed cow’s milk protein. The medicinal effectiveness of formula has been evaluated according to SCORAD index.

The results of research show, that in both groups have been noted a decreased of subjective showings in particular of itch and sleep disorder. In children who taking a hypo-allergic formula, are noted a good indicator of body’s weight and surplus of length, which points in favour of good endurance of formula. In 82% of diseased after 1–3 weeks from the beginning of diet-therapy, is noted a clinical improvements-decreased of skin’s inflammatory process has been proved according to subjective (itch, insomnia) and objective (erythema, dryness, humidity, excoriation) showings, which are fairly shown under SCORAD index.

**Conclusion**: A convincing and reliable results of a conducted research give a opportunity to make a conclusion, that to use a formula of diet-therapy in children with AD is expedient in specialized arsenal of children’s nutrition.

**Reference**

1. Hanifin JM, Rajka G. Diagnostic features of atopic dermatitis. Acta Derm Venereol Suppl (Stockh) 1980;92:44–7.

### PP007 BAFF and PAF: assessing long term stability in dried blood samples

#### Katia Basello^1^, Gabriele Piuri^2^, Attilio Francesco Speciani^3^, Michela Carola Speciani^4^, Carla Camerotto^4^, Francesco Zinno^5^

##### ^1^GEK srl c/o Cryolab, Rome, Italy; ^2^Inflammation Society, Orpington, United Kingdom; ^3^GEK srl, Milan, Italy; ^4^SMA srl, Milan, Italy; ^5^Department of Biomedicine and Prevention, Faculty of Medicine, “Tor Vergata” University, Rome, Italy

**Correspondence**: Katia Basello - katia.basello@gek-group.com

*Clinical and Translational Allergy* 2017, **7(Suppl 1)**:PP007

**Introduction**: The aim of this study is to evaluate the degradation over time of B Cell Activating Factor (BAFF) and Platelet-Activating Factor (PAF) in collected dried blood, evaluating the differences between two kinds of sample collectors: Whatman 903 Neonatal card (CARD) and Copan Nylon Swab (SWAB).

**Methods**: A dried blood spot is a common, moderately-invasive method for collecting patient blood from finger pricks. The blood samples are collected from 18 individuals. The collection of the samples is carried out in triplicate for both devices. Samples were stored at room temperature in the dark until serum extraction and analysis. Samples are assayed by Enzyme-Linked Immuno-Sorbent-Assay (ELISA) for BAFF (R&D Systems) and PAF (Elabscience). Serum extraction and ELISA assay were carried out 2 days (T2), 7 days (T7), 14 days (T14), 21 days (T21) and 28 days (T28) after collection. Results were presented as mean ± SEM.

**Results**: PAF values were stable over the time up to 21 days both for CARD and SWAB. At 28 days there was a reduction of up to 54.3 ± 6.76% for SWAB and up to 36.6 ± 5.5% for CARD (p < 0.001). As regards BAFF, values were stable until T21 while at T28 there was a decrease of up to 77.1 ± 6.3% for SWAB and up to 63.7 ± 4.5% for CARD (p < 0.001). According to Finkelman, there is a pathway of activation of immune system mediated by IgG, FcγRs, macrophages, and PAF. BAFF is a member of the tumor necrosis factor superfamily and an important regulator of peripheral B cell survival, maturation and immunoglobulin class-switch recombination. Many studies suggest that BAFF might modulate immune inflammation and could probably be one of the cornerstones of this IgG pathway of immune reaction. BAFF and PAF have both already been linked in non-atopic subjects to food reactions, supporting the possibility that these inflammatory molecules could be involved in non-IgE-mediated allergic reactions.

**Conclusion**: The data presented in this study should be considered for both clinical and research applications. The higher degradation of PAF compared to BAFF could be attributed to the different chemical nature of these two inflammatory molecules, lipid and protein respectively. The degradation over time of BAFF and PAF also depends on the type of technological support used for the collection of the specimen. Samples should be analysed as soon as possible and no later than 21 days from the time of collection to ensure greater stability of the values.

### PP010 Diagnostic approaches to the differentiating food allergies from food intolerances

#### Olga Pakholchuk

##### Zaporizhia State Medical University, Zaporizhia, Ukraine

**Correspondence**: Olga Pakholchuk - bessikalo2009@live.ru

*Clinical and Translational Allergy* 2017, **7(Suppl 1)**:PP010

**Introduction**: Literature reviews showed that FA clinical manifestations on the skin of various degrees of severity related to foods ingestion can arise as a result of a number of disorders and only some of which can be defined as allergic.

The aim of the research was to identify diagnostic approaches to the differentiating food allergies from food intolerances in children.

**Methods**: Skin elements were identified in complex with anamnesis data and results of the laboratory data. Hypersensitivity to food was detected by the skin tests (patch and prick), levels of the specific IgE and/or oral challenge test. Gut permeability was identified with 6-h lactulose urinary excretion.

**Results**: 56 patients (age 1–36 months old) with food allergy with typical allergic symptoms related to food intake were included into the study in the outpatient department. Major part of the parents (67%) couldn’t clearly identify the causative product. Only 15 children (26%) had positive specific IgE to milk and/or egg. Skin tests were positive in 10 of the patients (17.8%). Oral challenge test with milk and/or egg was positive only in 7 children (12.5%). Skin lesions vary from urticaria to papulation. Commonly non-immune reactions were nonspecific, not itching and not intense. Nevertheless of the type of the food intolerance mechanism skin dryness was presented in the major part of the patients. Moreover lactulose was detected in urine in 76.7% of cases both in group with immune and nonimmune reactions. There was no association of the lactulose level with type of the skin lesions. But intestinal permeability correlated with skin dryness (p < 0.05).

**Conclusion**: The study revealed that gut permeability in children with skin manifestations of food allergy often overlapping with symptoms found in nonimmune disorders such as disorders of absorption. Skin dryness can be its’ phenotypical marker. Gut permeability is the purpose for additional dietary corrections in children with FA.

### PP011 Validation of food hypersensitivity phenotypes using longitudinal observation and atopic sensitization data in the first 6 years of life

#### Olga Pakholchuk, Svitlana Nedelska

##### Zaporizhia State Medical University, Zaporizhia, Ukraine

**Correspondence**: Olga Pakholchuk - bessikalo2009@live.ru

*Clinical and Translational Allergy* 2017, **7(Suppl 1)**:PP011

**Introduction**: The aim of this study is to validate food hypersensitivity (FH) phenotypes in the survey using data of the 6-year examination and allergic sensitization assessment at 1, 3, and 6 years.

**Methods**: 196 children with FH symptoms diagnosed on the 1^st^ and 2^nd^ year of life were recruited in the outpatient department and completed 6-year follow-up. Skin prick testing and specific IgE detection was performed at 1, 3 and 6 years (n = 98, 53, 24, respectively). Using questionnaire and clinical examination and laboratory data at 6 m, 12 m, 2, 3 and 6 years we classified children into FH phenotypes: transient early, persistent, late-onset.

**Results**: It was found that non-immune FH mechanisms were commonly seen in patients with late-onset and persistent phenotype (p < 0.05). Children with transient FH symptoms both were as allergic, as non-allergic. Early onset phenotype patients more often had clear association with causative product and in almost ¾ developed food tolerance.

**Conclusion**: Immune FH more often manifests during first year of life. Late onset, especially after 2 y.o. as usual associated with food intolerance reactions, particularly if clear cause of the reactions is not seen.

### PP012 Using Component Resolved Diagnosis (CRD) in food allergy improves diagnostic-therapeutic appropriateness and therefore optimises financial resources

#### Stefano Pattini^1^, Maria Teresa Costantino^2^, Silvia Peveri^3^, Danilo Villalta^4^, Eleonora Savi^3^

##### ^1^Dermatology Department, Allergy Unit, Azienda Ospedaliero-Universitaria Policlinico di Modena, Modena, Italy; ^2^Centro Day Hospital Allergologia e Immunologia Clinica, ASST Carlo Poma Mantova, Mantova, Italy; ^3^Allergy Unit, G. Da Saliceto Hospital, AUSL Piacenza, Piacenza, Italy; ^4^Department of Laboratory Medicine, A.O.S. Maria degli Angeli, Pordenone, Italy

**Correspondence**: Stefano Pattini - pattini.stefano@gmail.com

*Clinical and Translational Allergy* 2017, **7(Suppl 1)**:PP012

**Introduction**: The aim of this study is to evaluate the percentage change of the diagnostic-therapeutic choice in complex polysensitized patients affected by food allergy, after using CRD compared to a first level survey (medical history and skin tests), along with an economic analysis of the patient’s overall management according to the two different approaches (assessment of higher costs and savings).

**Methods**: In this multicenter study in real life, 187 patients polysensitized to skin tests and with clinical symptoms related to an IgE mediated food allergy were recruited. Each patient was submitted to skin prick tests (SPT) with a standard panel of food allergens and to a blood sample in order to detect specific IgE against food recombinant molecules (CRD), which were chosen according to medical history and positivity to SPT, by ImmunoCAP method (Thermo Fisher Scientific^®^, Uppsala, Sweden) or, when not available, by Microarray ISAC 112 allergens system (Thermo Fisher Scientific^®^, Uppsala, Sweden). Then the first diagnostic-therapeutic hypothesis, based only on medical history and skin tests, was recorded for each patient and, after that, the second diagnostic-therapeutic choice (final) was made, the one which would be implemented considering the CRD outcome. Therefore, an evaluation of the change of the diagnostic-therapeutic choice was carried out, in percentage, between the first hypothesis and the final choice, analyzing statistically the results by the agreement coefficient (k index) and the Chi square test. Finally, a detailed analysis was conducted on the economic impact of a molecular approach to the overall management of the allergic patient in order to evaluate whether the increase in the diagnostic costs would be compensated and eventually exceeded by savings coming from the increasing diagnostic-therapeutic appropriateness.

**Results**: A change in the prescription of self-injected adrenaline has been observed in about 50% of patients (k index 0.56) and an overall saving of financial resources along with a higher diagnostic-therapeutic appropriateness has been pointed out too.

**Conclusion**: In this study there is only moderate agreement concerning prescription of self-injected adrenaline before and after performing CRD: as a result, it is highlighted the usefulness of CRD, at least in complex polysensitized patients, in indicating risk assessment and therefore the correct therapy of food allergy, thus resulting in a cost-saving approach.

### PP013 Food IgG antibodies: study of long-term stability of dried blood samples

#### Gabriele Piuri^1^, Katia Basello^2^, Attilio Francesco Speciani^3^, Andrea Costanzi^2^, Francesco Zinno^4^

##### ^1^Inflammation Society, Orpington, United Kingdom; ^2^GEK srl c/o Cryolab, Rome, Italy; ^3^GEK srl, Milan, Italy; ^4^Department of Biomedicine and Prevention, Faculty of Medicine, “Tor Vergata” University, Rome, Italy

**Correspondence**: Gabriele Piuri - gabriele.piuri@me.com

*Clinical and Translational Allergy* 2017, **7(Suppl 1)**:PP013

**Introduction**: A dried blood spot is a common, moderately-invasive method for collecting patient blood from finger pricks. The aim of this study is to evaluate the long-term stability in the collection and detection of food-specific IgG antibodies comparing two different kinds of sample collectors: Whatman Neonatal card 903 (CARD) and Copan Nylon Swab (SWAB). The blood samples are tested by IgG Enzyme-Linked Immuno-Sorbent-Assay (ELISA) for precision and accuracy.

**Methods**: The blood samples are collected from 18 individuals (10 females and 8 males). The collection of samples is carried out in triplicate for both devices. Serum samples are assayed for food-specific IgG. The specific IgG antibody responses are analyzed on a customized ELISA plate (Immunolab GmbH) wich detects total IgG for 33 food antigens in serum. Samples were stored at room temperature in the dark. Serum extractions and ELISA assays were carried out after 2 days (T2), 7 days (T7), 14 days (T14), 21 days (T21) and 28 days (T28). Since the Kolmogorov–Smirnov normality test revealed non-normal distribution of the parameters, results were presented as median and interquartile range (IQR).

**Results**: Over time, analyses show progressively smaller results both as regards the CARD and the SWAB (p < 0.001). While between T2 and T7 there were no statistically significant changes, in T14 there was a significant reduction of −17% (−0.45 to +0.03) to the initial value that is maintained up to T21. Between T21 and T28, there were no statistically significant differences regarding CARD, while for SWAB there was a further reduction of values of up to −29% (−0.46 to −0.08). As discussed by Ligaarden, the concentration of specific IgG for food reflects the use of that food in the diet. In addition, Finkelman showed a IgG-dependent mechanism of anaphylaxis which involves IgG, FcγRs on macrophages, basophils and neutrophils, complement-derived peptides C3a e C5a, B cell Activating Factor (BAFF) and Platelet Activating Factor (PAF). For a more accurate study of the possible interaction between these mechanisms associated with IgG, it is important to know precisely the advantages and disadvantages of the most used methods in this area of investigation.

**Conclusion**: Considering the deterioration of biological samples over time it is important that the dosage of food IgG antibodies in dried blood is carried out as quickly as possible and in any event within 21 days of collection.

### PP014 Diagnostic algorithm for gluten intolerance in children

#### Vera A. Revyakina, Marina A. Kiseleva, Elena D. Kuvshinova, Inna A. Larkova, Anton A. Shekhetov

##### Federal State Budgetary Scientific Institution «Federal Research Centre of Nutrition, Biotechnology and Food Safety», Moscow, Russia

**Correspondence**: Vera A. Revyakina - 5356797@mail.ru

*Clinical and Translational Allergy* 2017, **7(Suppl 1)**:PP014

**Introduction**: The gluten intolerance is associated with celiac disease and Allergy to gluten, the criteria for the differential diagnosis of which is still insufficiently developed.

The aim of this study is the development of a diagnostic algorithm for gluten intolerance in children for the purpose of adequate diet therapy application to prevent development of complications and severe forms of the disease.

**Methods**: Under supervision there were 42 children with chronic diarrhea and symptoms of impaired intestinal absorption. The diagnostic algorithm consisted of the following stages. The first stage—the detection of the risk groups for gluten enteropathy. These include: (1) immediate relatives of patients with celiac disease; (2) close relatives with allergic diseases; (3) children with gastrointestinal symptoms resistant to standard therapy; (4) children, lagging in physical development and with symptoms of impaired intestinal absorption; (5) patients with dermatitis herpetiformis and diseases that can be associated with celiac disease. At the second stage children included in the risk group were subjected to screening diagnostics. We used the BIOCARD TM Celiac Test (Finland), which allows to determine antibodies to tissue transglutaminase (IgA). Patients with a confirmed diagnosis of celiac disease and positive (BIOCARD TM Celiac Test) test was not included in further examination. The third stage of gluten intolerance diagnostics was in-depth examination of children with gastrointestinal and skin manifestations of Allergy, including anamnesis and clinical symptoms, determination of total IgE, IgG, IgA, IgM and specific IgG and IgE antibodies in the blood serum.

**Results**: Three groups of patients were identified. The first group consisted of 23 children with positive allergen-specific IgE antibodies to gluten and wheat and to cow’s milk protein in serum. In 10 children from the second group IgE and IgG antibodies to gluten and wheat serum were identified at the same time. They had gastrointestinal symptoms within a few hours after eating a certain amount of foods containing gluten (individual for each child). The third group of patients included 9 children with IgG antibodies to gluten and wheat serum. Clinically they mainly had skin rashes.

**Conclusion**: The staged diagnostic algorithm for gluten intolerance in children allows to determine the mechanism of gluten intolerance and to prescribe required elimination diet and treatment aimed at prevention of serious complications.

### PP015 Accuracy of skin prick test to access plant food allergy

#### Diana Silva, André Moreira, José Plácido, Alice Coimbra

##### Serviço de Imunoalergologia, Centro Hospitalar de São João E.P.E., Porto, Portugal

**Correspondence**: Diana Silva - disolha@gmail.com

*Clinical and Translational Allergy* 2017, **7(Suppl 1)**:PP015

**Introduction**: Plant food allergy diagnosis is challenging due to the different implicated foods, clinical manifestations and geographic diversity. We aimed to assess the diagnostic accuracy of single food allergenic proteins combined with plant and food skin prick test extracts (SPT) to evaluate plant food allergy.

**Methods**: This is a cross-sectional retrospective study. All SPT performed in an allergy department of Porto, Portugal from 09/2014 to 12/2015 (n = 3141 patients) were included and those evaluating sensitization with extracts to fresh fruits (soy, watermelon, banana, apple, peach, kiwi, avocado, fig), nuts (walnut, hazelnut, almond, pinenut, peanut and chestnut), lipid transfer proteins (LTP), profilin and latex were selected (n = 189 patients). Clinical and demographic data, sIgE, SPT with fresh foods, and open food challenges (OFC) results were reviewed from patients records. Plant food allergy syndromes were identified and the accuracy of the skin tests were calculated using medical diagnosis as the gold standard. Continuous data are presented as median and interquartile range and categorical as n(%). Mann-whitney and Chi square were used to compare differences between groups.

**Results**: Of all the tests, 89(48%) were performed due to plant food allergy suspicion and in 58(65%) diagnosis was confirmed: profilin mediated allergy (n = 17); LTP allergy syndrome (n = 20); both profilin and LTP (n = 2); birch-plant food syndrome (n = 4), latex-fruit (n = 4); and to specific foods (peanut n = 3; walnut n = 1; banana n = 1; kiwi n = 4; wheat n = 1; blackberry n = 1). Most were women 41(71%), with a median age of 22 [18;32] years, 43(74%) had rhinitis and 15(26%) asthma. Oral allergy symptoms (OAS) were the most common complaint 27(47%), followed by anaphylaxis 15(26%) and urticaria/angioedema 14(24%). LTP mediated syndrome had more anaphylaxis and a lower number of OAS compared with profilin mediated syndrome (10 vs 1, p = 0.003; 3 vs 14, p < 0.001). The sensitivity and specificity of using combined SPT extracts was 83.0 CI 95% [69.7;91.5] and 95.7[76.0;99.8], respectively. Using only LTP and profilin improved specificity 100.0[82.2;100.0] but not sensitivity 71.7[57.4;82.8].

**Conclusion**: LTP and profilin plant food mediated allergy syndrome were the most frequently encountered in our population. LTP sensitization was associated with more severe reactions. SPT with extracts including LTP and profilin were useful as a first line diagnostic tool to identify plant food allergy syndromes.

### PP016 Double blind placebo controlled food challenge (DBPCFC)

#### Hanneke van der Kleij, Esther van Twuijver, Robbert Sutorius, Pieter-Jan de Kam

##### HAL Allergy B.V., Leiden, the Netherlands

**Correspondence**: Hanneke van der Kleij - hvdkleij@hal-allergy.com

*Clinical and Translational Allergy* 2017, **7(Suppl 1)**:PP016

**Introduction**: In Phase II/III clinical development, the Double Blind Placebo Controlled Food Challenge (DBPCFC) is the generally accepted primary measure to evaluate the effectivity of immunotherapy with relevant food allergens. The DBPCFC is a challenge procedure with increasing doses of food allergen to determine the reactivity and threshold dose to food a specific food allergen and serves as the gold standard to confirm diagnosis of food allergy. HAL Allergy is developing a chemically modified peanut allergen preparation for immunotherapy and requires a standardised, pharmaceutical-grade oral challenge test to determine efficacy during clinical development.

**Methods**: A systematic literature search was performed to grade (scale 0–11) the relevance and quality of food challenge tests being published to define a standardised food challenge protocol, an adequate methodology for standardised DBFPCFC. Grading was performed independently by two viewers.

**Results**: Peanut material should be roasted and ground into a powder. The allergens will be administered to the patient in a matrix. Matrices (such as vanilla, cinnamon and cocoa) need to be capable of blinding the sensory properties of the food in a small volume compatible with use in the target patient group. Hypoallergenic pudding, applesauce or oatmeal are very suitable vehicles. The amount of incremental steps in a DBPCFC, with a dosing interval of preferably 20 min, should not exceed 10 dosages. For our peanut immunotherapy clinical studies, a dosing schedule ranging from 0.1–300 mg is proposed for the DBPCFC before treatment and from 1 to 3000 mg (or possibly 6000 mg) of peanut protein post-treatment. Placebo and active should be administered on separate days in random order, with at least 48 h between challenges. DBPCFCs need standardisation regarding the interpretation of objective and subjective symptoms. Standard criteria to define a positive challenge outcome do not exist and the variability in symptom assessment is relatively high.

**Conclusions**: The literature search has resulted in recommendations for the design of the food challenge protocol to be used in our peanut immunotherapy in upcoming Phase II/III clinical studies, especially with regards to dosing schedules, matrix, physical and psychological health, contraindications and other logistic considerations. Further research is needed to standardize signs and symptoms to define a positive food challenge.

### PP017 New validated recipe for wheat for double blind placebo controlled food challenge in adults

#### Jenny van Odijk^1^, Helen Lindqvist^2^, Elin Lustig^2^

##### ^1^Department of Allergology, Sahlgrenska University Hospital, Gothenburg, Sweden; ^2^Department of Clinical Nutrition, Sahlgrenska University Hospital, Gothenburg, Sweden

**Correspondence**: Jenny van Odijk - jenny.van.odijk@vgregion.se

*Clinical and Translational Allergy* 2017, **7(Suppl 1)**:PP017

**Introduction**: DBPCFC (Double Blind Placebo Controlled Food Challenge) is considered the golden standard for investigation of food hypersensitivity especially if allergy tests are negative and the patient history is vague. Many recipes used for this purpose includes elimination diet products and /or are not validated in adult subjects with regard to taste, volume and blinding procedure.

Our aim was to develop an allergen adapted and blinded recipe for wheat protein for DBPCFC use in adult patients, using ordinary staple foods. Another aim was to evaluate the recipes with regard to taste and acceptance.

**Methods**: The study consisted of three parts:

1. *Development of recipe*

For the development of recipes, several food items were tested in different amounts to mimic a smoothie drink. Standard heat treated wheat flour was used as a base for the food challenge. Rice-, coconut-, oat- or soydrinks were tested as base flavoured with either fruit purées, cacao or vanilla, both for active and placebo drink development.

2. *Acceptance test*

An acceptance test was performed in a small group of eight students and employees at Gothenburg University, during a strict setting. All subjects filled in taste and acceptance evaluation using a 9- grade hedonic scale.

3. *Triangle test*

Triangle test took place in a separate setting. Thirty-eight adults (non smokers and non allergic) took part in the test. The test was performed following standard proceedings. The critical value for a statistical difference (n = 38): 19 correct answers; p = 0.05).

**Results**: Development of recipe: a recipe using coconut milk, wheat flour or gluten free mix, milk free chocolate powder, vanilla flavoring was considered usable for further testing and evaluation. The challenge drink contains 220 kcal and measures around 270 ml in portion size, comparable to a commercial smoothie drink.

Acceptance test: Both taste and volume got acceptable results in the test. The mean value for taste was 5.6 out of 9 and for acceptable volume 7.9 out of 9. Triangle test: Out of 38 subjects, 16 pointed out the odd sample in the test i.e. p < 0.05 and the challenge drink and the control drink were thus not possible to identify in a blinded test.

**Conclusions**: This study has shown that it is possible to develop a blinded recipe for use of DBPCFC using an “adult portion” of wheat protein. The recipe has acceptable portion size, few ingredients, contains ordinary staple foods and has low cost. Further recipes for other allergenic foods will be developed using the same model.

### PP019 Pollen-food syndrome in a pediatric population attending an Allergy Clinic in Mexico City

#### Amyra Ali Azamar Jácome, Karla Leversia Borjas Aguilar, Miguel García Domínguez, David Alejandro Mendoza Hernández

##### Instituto Nacional de Pediatría, Mexico City, Mexico

**Correspondence**: Amyra Ali Azamar Jácome - amyra.aaj@gmail.com

*Clinical and Translational Allergy* 2017, **7(Suppl 1)**:PP019

**Introduction**: The aim of this study was to explore the epidemiological characteristics and sensitization patterns in children with pollen food syndrome (PFS) at a specialist allergy clinic in Mexico City.

**Methods**: We enrolled in this study every patient aged between 6 and 18 years reviewed at the Pediatric National Institute Allergy clinic between March 2015 and March 2016. Diagnosis of PFS was made according to PFS diagnostic questionnaire and algorithm by *Skypala* et al. Skin prick testing (SPT) to pollens and prick by prick testing (PPT) to fresh foods were performed in a subset of patients with PFS. Those negative to PPT, underwent open oral food challenge (OFC).

**Results**: Within the studied population prevalence of PFS was 5.4%. Their average age was 10+/−3, 60% between 6 and 10 years, 35% from 11 to 14 years and only 5% between 15 and 18 years; 45% were women and 55% men. Apple (55%), peach (50%) and banana (50%), were the foods most commonly implicated. Rosaceae family was responsible in 75% of the cases, followed by Musaceae with 50%, Lauraceae with 45% and Cucurbitaceae and Fabaceae with 40%. All but one patient had allergic rhinitis, 50% had asthma, 35% atopic dermatitis, 25% had all three and 10% had other type of food allergy. In the subset of patients with PPT to fresh foods, 90% had a positive result and 10% had a negative one, these patients underwent open OFC which turned out positive. Sensitization to *Quercus robur and Alnus glutinosa* were the most frequents (40%), followed by *Fraxinus excelsior* (35%) and *Betula verrucosa* (30%). There is little knowledge about FPS in Mexico and there are no previous studies in pediatric population. We found that FPS affects males and females equally, being more frequent between 6–10 years. When compared with international literature, the prevalence is similar and we also found apple and peach as the most frequent foods implicated. Although, we also found banana as a frequent food responsible for FPS, similar to what is reported in the Mexican study performed in adults. Contrary to studies performed worldwide, we found birch sensitization in only 30% in FPS, being more frequent other *Alnus glutinosa* and *Quercus robur*.

**Conclusions**: It appears to be a significant difference in sensitization patterns to aeroallergens in Mexican pediatric population with FPS. Foods implicated seem to be similar to national and international reports. More studies are required to known the real characteristics of FPS in Mexico.

### PP020 Prevalence of allergy to wheat in patients who report a history of reactions after ingesting food which contains gluten

#### Cristiano Caruso^1^, Cono Casale^2^, Gian Lodovico Rapaccini^2^, Antonino Romano^1^, Italo De Vitis^2^

##### ^1^Allergy Unit, Fondazione Policlinico Gemelli, Rome, Italy; ^2^Gastroenterology Unit, Fondazione Policlinico Gemelli, Rome, Italy

**Correspondence**: Cristiano Caruso - cristiano.caruso@policlinicogemelli.it)

*Clinical and Translational Allergy* 2017, **7(Suppl 1)**:PP020

**Introduction**: The association between food allergy and celiac disease (CD) and Non celiac Gluten sensitivity (NCGS) is still to be clarified. Gluten-related disorders have gradually emerged as an epidemiologically relevant phenomenon with a global prevalence that is estimated around 5%, drawing the attention of the scientific community. Epidemiological studies estimate a worldwide prevalence of CD of approximately 1:100 individuals, with a considerable proportion of patients remaining undiagnosed and untreated. According to a study performed by the National Health and Nutrition Examination Survey in the United States, the prevalence of self-prescribed GFD in an unselected population of subjects aged 6 years or older was 0.5%. Epidemiological studies report a prevalence of WA in American population of around 0.4% untill 0.6%. The diagnosis of WA is classically based on skin prick tests (SPT), in vitro specific Immunoglobulin E (sIgE) assays and functional assays. SPTs and sIgE in vitro assays are the first-level diagnostics for WA. However, they are affected by a low predictive value. In particular, their low sensitivity can be explained by the fact that the commercial test reagents are mixtures of water/salt-soluble wheat proteins that lack allergens from the insoluble gluten fraction.

**Case report**: In our unit of celiac disease and related conditions to gluten we visited in one year about 400 patients. Of these 113 they were not celiac but were investigated for suspected non-celiac gluten sensitivity. After in vitro tests for the exclusion of celiac disease, the same patients underwent allergologic workup consists of: skin prick tests for foods including wheat (Alk-abello), LTP (lipid transfer protein) (peach Alk abello), alpha amylase, wheat flour, barley, corn, rice, grass pollen and histamine. Also all they performed patch tests for suspected allergy to nickel, if they reported reactions after a few hours of ingestion of gluten. Molecular-based allergy (MA) diagnostics could overcome some limitations of sIgE in vitro assays using wheat flour extracts. We have used omega-5 gliadin (Tri a 19) and nsLTP (Tri a 14), gliadin, wheat, gluten that are available in the ImmunoCAP™ assay, whereas the alpha-amylase/trypsin inhibitor (Tri a aA/TI) is available only in the microarray ISAC™ assay. The sIgE to omega-5 gliadin assay is highly reliable and now widely used to identify the patients with WDEIA.

**Conclusion**: Of a total of 104 patients with a history of immediate reactions and not immediate after ingesting gluten, we found wheat protein sensitization in 14 patients (13%). In addition of 300 celiac patients we also found 5 patients with allergy to wheat or wheat protein (1.5%) different percentage than that reported in the literature.

**Consent to publish**: Authors confirm that the individual(s) described have authorized them to publish the findings related with these cases.

### PP021 Clinical profile of food allergic patients in Brazil – Evaluation of a questionnaire

#### Renata Cocco^1^, Carolina Aranda^1^, Marcia C. Mallozi^1^, Jackeline F. Motta^2^, Lilian Moraes^3^, Antonio Pastorino^1^, Nelson Rosario^4^, Ekaterini Goudouris^5^, Arnaldo Porto^6^, Neusa F. Wandalsen^7^, Emanuel Sarinho^8^, Flavio Sano^9^, Dirceu Solé^1^

##### ^1^Federal University of São Paulo, São Paulo, Brazil; ^2^Federal University of Sergipe, Aracaju, Brazil; ^3^Federal University of Mato Grosso, Cuiabá, Brazil; ^4^Federal University of Paraná, Curitiba, Brazil; ^5^Federal University of Rio de Janeiro, Rio de Janeiro, Brazil; ^6^University of Rio Grande do Sul, Passo Fundo, Brazil; ^7^Medical School of ABC, Santo André, Brazil; ^8^Federal University of Pernambuco, Recife, Brazil; ^9^Nipo-Brasileiro Hospital, São Paulo, Brazil

**Correspondence**: Renata Cocco - renata.cocco72@gmail.com

*Clinical and Translational Allergy* 2017, **7(Suppl 1)**:PP021

**Introduction**: The prevalence of food allergy (FA) is increasing and many factors may contribute to these results. The aim of this study was to perform a clinical examination of patients with FA in Brazil.

**Methods**: Cross-sectional study applying a written questionnaire (WQ) to parents of children presenting with food allergy in ten Pediatric Allergy Centers in Brazil.

**Results**: We evaluated 120 WQ from children aged 1–18 years-old. Median age for onset of symptoms was 7.8 months and 56.5 months by the time of WQ performance. The most frequently referred foods were: cow’s milk (86%), egg (32%), wheat (4.1%), soybean (3.3%), peanuts (2.5%) and seafood (2.5%). Allergies to a single food were observed in 71.6% and 28.3% described multiple food allergies. The most prevalent symptoms were urticaria (44%), atopic dermatitis (AD) (29%), anaphylaxis (22.5%), eosinophilic esophagitis (2.5%) and wheezing (1.7%). The severity of food allergy was mild in 33.3%, moderate in 29.1% and severe in 37.5%; 17.5% were hospitalized due to food allergy. Food restriction was applied in 100% of patients. Regarding to other allergies, 47.5% of patients referred asthma/rhinitis associated. Skin prick test was performed in 37.5% of children, with positive results in 88.8%. Oral food challenge was performed on 36% and 70% failed the test. Patients with AD performed the Scoring Atopic Dermatitis (SCORAD), averaging 40. Risk factors significantly associated with severe food allergy were family history of rhinitis (OR = 4.1; 95% CI = 1.41–4.5), asthma (OR = 2.5; 95% CI = 1.5–3.7) and smoking (OR = 1.4; 95% = = 1.1–2.0). In this sample, the symptoms began in the first year of life in congruence with most studies. The most reported food was cow’s milk and egg, as well as reported in the literature, especially in children. Despite the WQ had been applied to patients up to 18 years, the mean age of them was 4 years old. The major limitation of this study was that the WQ was completed in specialized services with more severe patients.

**Conclusions**: Due to the increasing prevalence of FA, knowledge about food-related allergies, diagnostic tests and possible risk factors in specific population are important keys to create strategies for prevention and possible treatment options beyond food exclusion.

### PP024 Foods reported as responsible for OAS in Greece

#### Anna Iliopoulou^1^, Constantinos Pitsios^2^, Maria Petrodimopoulou^1^, Maria Konstantakopoulou^1^, Ekaterini Papadopoulou^1^, Maria Passioti^1^, Nikolaos Mikos^1^, Meropi Kontogianni^2^, Evangelia Kompoti^1^

##### ^1^Laikon General Hospital, Athens, Greece; ^2^Harokopio University, Athens, Greece

**Correspondence**: Anna Iliopoulou - iliopoulou.anna@gmail.com

*Clinical and Translational Allergy* 2017, **7(Suppl 1)**:PP024

**Introduction**: Oral allergy syndrome (OAS) is a clinical expression of food allergy mainly caused by foods of plant origin. The culprit allergens are cross-reacting proteins, like profilin, that are also present in pollen, triggering allergic respiratory symptoms. The aim of our study was to detect the foods of plant origin that are referred as responsible for OAS in Greek adults.

**Methods**: 264 atopic adult patients, with allergic respiratory symptoms and/or food allergy were interviewed in our study. A complete record of their atopic medical history was obtained; symptoms of allergic rhinitis, asthma and signs of atopic dermatitis, as well as their family history of atopy. Information regarding any symptoms of food allergy and the type of reaction (OAS or systemic reaction) were collected. The foods that patients considered responsible were registered.

**Results**: 79/264 of our atopic patients (30%) referred symptoms of OAS, while 50/264 patients (18.9%) had experienced systemic reactions. Foods of plant origin implicated in OAS symptoms were: peach; 23/79 (29%), walnut; 21/79 2(6.5%), kiwi; 17/79 (21.5%), banana; 14/79 (17.7%), hazelnut; 9/79 (11,4%), peanut; 8/79 (10%), eggplant/apricot; 7/79 (8.8%), melon; 6/79 (7.5%), strawberry/almond/cherry; 5/79 (6%), cabbage/tomato/watermelon/apple/pineapple/grape/orange/pistachio/spinach; <5/79 (3–5%). Finally, less common foods, each referred only once by our patients, were: sesame/sunflower seed/pear/cashew nut/brazil nut/lemon/cucumber/bell pepper/mustard/chestnut/fig/mango/papaya/plum/onion/lentil/peas. Our results in Greek adults show some similarities but also some diversities in comparison with the relative ones from other Mediterranean countries. Similarities are found between Greek and Spanish populations, which refer fruits of the Rosaceae family as the most common cause of OAS, followed by kiwi, peach alone, melon and banana. On the contrary, Italian studies mention melon as the most common culprit food, followed by watermelon, tomato, banana and pineapple.

**Conclusions**: Peach, walnut, kiwi, banana and hazelnut are the top 5 foods referred as responsible for OAS in Greece.

### PP025 Prevalence of childhood food allergy in Batumi

#### Liana Jorjoliani, Rusudan Karseladze, Lali Saginadze, Nino Adamia

##### Iv. Javakhishvili Tbilisi State University, Tbilisi, Georgia

**Correspondence**: Liana Jorjoliani - lia-zhorzholiani@mail.ru

*Clinical and Translational Allergy* 2017, **7(Suppl 1)**:PP025

**Introduction**: To investigate the prevalence of food allergies and to identify the most common causes of the allergies in the population of preschool children from the city of Batumi.

**Methods**: The study was conducted with children population, in Batumi. The random sampling was applied in the study to ensure representative sample and the cross section method of epidemiological survey was used. At the first stage of the survey 840 children aged from 6 months to 7 years, were questioned. Data were collected through the face-to-face and phone interviews. At the second stage of the survey, the subjects of study were the part of the population, who revealed the clinical signs of food allergy for the last 12 months. The causal factors were revealed on the basis of answers to the questionnaire, anamnesis data, specific IgE measurements and skin prick tests. The risk factors were studied on the basis of case control studies.

**Results**: Skin manifestation (76%) of food allergy was significantly (p < 0.05) higher than the rate of gastrointestinal symptoms (24%) in the studied population. The most common causes of food allergies in the study population were different food addi-tives (29.2%), fish (22.6%), eggs (22.3%), milk (18.6%), honey (13.7%;) and nut (4.3%). Such factors as inheritance from mother (OR 13.69; 95% CI 7.08–27.04), excess weight of newborn (OR 1.08; 95% CI 0.30–3.82) and bottle feeding (OR 5.29; 95% CI 3.30–8.51) are associated with higher risks of food allergies. IgE mediated reactions to the food allergens were identified in 98% of the patients. The prevalence of parentally reported allergy was 15.8%, while proven allergy cases were 6.1% positive on the basis of IgE/STP. Thus, the allergy presence is significantly overestimated by parent (p < 0.01). This problem can be explained by lack of information and education about food allergy in parents. Allergy to nut is significantly higher in children between 3 and 5 years than in children of younger age, while allergy to milk and eggs was same in all ages.

**Conclusions**: In the childhood, allergic skin manifestation of food allergy is high. According to the data on obtained in the study, management of the risk factors as well as education of parents and caregivers play significant role in the focused and effective prevention of disease.

### PP026 Evaluation of using an adrenaline in children during an oral food challenge

#### Ekaterina Khaleva^1^, Ana Prieto del Prado^1^, George Du Toit^1,2^

##### ^1^Guy’s and St Thomas’ NHS Foundation Trust, London, United Kingdom; ^2^King’s College London, London, United Kingdom

**Correspondence**: Ana Prieto del Prado - anaanap@hotmail.com

*Clinical and Translational Allergy* 2017, **7(Suppl 1)**:PP026

**Introduction**: Oral food challenges (OFC) are the gold standard for the diagnosis of food allergy. They are also the method of choice to assess the resolution of food allergies. In clinical practice, most centres use open OFC for diagnostic purposes. OFC’s are logistically demanding and not without risk. It has therefore been suggested that between 30–40% of OFC’s should result in a positive challenge; of these positive reactions only a few should result in anaphylaxis requiring treatment with adrenaline. The aim of this study was to study in detail the clinical characteristics of anaphylaxis and time after first exposure to first symptoms in comparison with allergic background of the child.

**Methods**: A chart review study of 3979 patients undergoing the food challenge was conducted at tertiary Allergy Centre. We identified all patients for whom adrenaline was administered for food-induced anaphylaxis between 2008 and 2016 year and analyzed demographic characteristics such as age, gender, type of food given in the challenge, time after first exposure to first symptoms, treatment required, skin prick test, specific lgE and allergic co-morbidities.

**Results**: 32 patients had anaphylaxis for which adrenaline administration was required. Of the patients who developed these severe symptoms, 22 (68.75%) males and 10 (31.25%) females, median age was 4.9 years. Six (18.75%) of these reactions occurred to cow’s milk of which 4 (66,6%) were to baked milk and 2 (33,4%) to fresh milk. Ten (31.25%) children reaction to nuts, 1 (3.13%) to soya, 3 (9.38%) to egg, 4 (12.5%) to sesame, 4 (12.5%) children to wheat. Children, who were allergic to nuts reacted sooner after exposure than that recorded for other foods. The most common co-morbidities were other food allergies in 23 cases (71.9%), 17 (53.2%) with eczema, 10 (31.3%) with asthma, 7 (21.9%) with allergic rhinitis.Of those children for whom adrenaline was administered, five required repeat adrenaline administration.

**Conclusions**: This audit reveals that anaphylaxis during OFC’s in a bust tertiary Allergy Centre seldom results in the need for adrenaline administration for the treatment of allergic reactions. However, all foods are capable of producing anaphylaxis in this setting of which milk was the commonest cause. Reactions can occur soon after allergen exposure, especially when the challenge is undertaken to nuts. It is difficult to predict who is most at risk for severe allergic reactions; all children in this audit had allergic co-morbidities and food allergy was the most frequent one. Children who have both asthma and a food allergy are at greater risk for anaphylaxis. Severe reactions requiring treatment with adrenaline was common, but few children required multiple doses of adrenaline. OFC’s, which serve as the gold standard diagnostic modality are generally safe but severe reactions do rarely occur for which adrenaline treatment is required.

### PP027 Almond and peanut allergy: how commonly sensitised and how commonly reactive?

#### Sofia Kostoudi, Nikolaos Douladiris, Nikolaos Kitsioulis, Emmanouil Manousakis, Nikolaos G. Papadopoulos, Paraskevi Xepapadaki

##### Allergy Unit, 2^nd^ University Pediatric Clinic, University of Athens, Athens, Greece

**Correspondence**: Nikolaos Kitsioulis - drnok21@gmail.com

*Clinical and Translational Allergy* 2017, **7(Suppl 1)**:PP027

**Introduction**: Tree nut and peanut allergy are responsible for a large number of severe IgE-mediated reactions. Almond is widely used in manufactured and homemade sweets or as a snack, while peanuts, alone or in other products are increasingly consumed in the Greek diet.

We aimed to assess sensitization and reactivity patterns to peanut and almond in children with a reported immediate reaction after consuming nuts or peanuts (cashew, pistachio, walnut, hazelnut, peanut and almond).

**Methods**: We retrospectively studied data on sensitization patterns by means of skin prick tests (SPTs) and specific (s) IgE in children with symptoms suggestive of IgE mediated allergic reaction following consumption of tree nuts or peanut, confirmed by positive IgEs and/or SPTs, visiting our Unit between 2012 and 2015.

**Results**: 204 children were included, 136 males, mean aged 8.2 ± 3.2 years, in whom 264 reactions were recorded. The most common provocative agent was pistachio (24.9%) and cashew (24.5%), while hazelnut and walnut were associated with 16.1% and 15.4% of the reactions respectively. Almond was the least reported (2.7%, n = 7), followed by peanut (13.6%, n = 36). Among the 7 patients with a reported reaction to almond sIgE was 27.4 kU/L (SE = 12.31), while SPT max diameter was 5 mm [3.22, 6.77]. 93 of the sensitized children to almond (with no reported reaction), had sIgE of 2.6 kU/L [1.75, 3.45], while SPT max diameter was 5.16 mm [4.68, 5.64]. Open food challenges (OFCs) in this population were negative. 36 children with a reported peanut reaction, had sIgE to peanut 45.93 kU/L, 95% CI [31.75, 65.11] and SPT and max diameter was 10.8 mm [9.12, 12.49]. 53 of the sensitized children to peanut (with no reported reaction), had sIgE of 2.32 kU/L [1.57, 3.07] while SPT max diameter was 4.6 mm [3.96, 5.25]. Open food challenges (OFCs) in this population were negative. To notice, a large proportion (50%, n = 102) of our study population was sensitized to almond; although small number reported reaction. Regarding peanut, 48.5% (n = 99) of the total population were sensitized with slightly more than one third of that group (36.7% of the peanut sensitized children) reporting immediate type of reaction.

**Conclusions**: Almond and peanut have a considerably high sensitization rate among children visiting our Unit with reaction to nuts, however true reactors were only few. Peanut reactions are less frequent than these to tree nuts, however cannot be overlooked.

### PP028 The popularity of the use of probiotics in patients with allergic diseases

#### Edyta Krzych, Urszula Samolinska-Zawisza, Konrad Furmanczyk, Aneta Tomaszewska, Filip Raciborski, Agnieszka Lipiec, Piotr Samel-Kowalik, Artur Walkiewicz, Jacek Borowicz, Boleslaw Samolinski

##### Unit of Environmental Hazard Prevention and Allergology, Warsaw Medical University, Warsaw, Poland

**Correspondence**: Edyta Krzych - edyta.krzych-falta@wum.edu.pl

*Clinical and Translational Allergy* 2017, **7(Suppl 1)**:PP028

**Introduction**: The aim of the study was an attempt to assess the frequency of the use of probiotics in the group of subjects in the light of the Epidemiology of Allergic Diseases in Poland project.

**Methods**: The study group consisted of 4.783 subjects aged 6–7 years, 13–14 years and adults (20–44 years) from the eight largest Polish urban centres. The method that was used was the ECRHS II and ISSAC survey questionnaire as well as additional studies including those concerned with the problems related to the range of probiotics’ uses.

**Results**: The probiotics used were very popular in the group of subjects with diagnosed allergic diseases and were not only combined with antibiotic therapy but also supplemented with kefir and yogurt. The protective action could be observed especially at the age above 14 years. The preventive effect was not observed at the age of early childhood.

**Conclusions**: Probiotics have relatively often used in the population under study and a health-improving effects was mainly observed at the age over 14 years old.

### PP029 Prevalence of food sensitisation, IgE-mediated and Non-IgE-mediated food allergy among pediatric patients diagnosed with autism spectrum disorders

#### Aimee Lou Nano, Marysia Recto

##### University of the Philippines, Philippine General Hospital, Manila, Philippines

**Correspondence**: Aimee Lou Nano - aimeenanomd@gmail.com

*Clinical and Translational Allergy* 2017, **7(Suppl 1)**:PP029

**Introduction**: Autism spectrum disorders and food allergies are conditions with increasing prevalence. Studies have investigated the link between intake of certain food among ASD patients and onset of adverse reactions. Results of these studies are varied and conflicting. This is the first local study on the prevalence of food allergies among these patients.

This study aims to determine the prevalence of food sensitization, IgE-mediated and non-IgE-mediated food allergies among pediatric ASD patients. It also aims to determine the prevalence of perceived food allergies, and its triggers and the types of reactions of perceived food allergies and on open food challenge.

**Methods**: This is a cross-sectional, prospective study. Pediatric patients diagnosed with Autism Spectrum Disorders were enrolled in the study. Excluded were: children with uncontrolled asthma, with a recent anaphylactic reaction, and those on chronic high dose steroid therapy. Required sample size is 92. Complete history and PE were obtained and previous reactions to food and suspected allergens were duly noted. All patients underwent skin prick testing (SPT) to cow milk, wheat, soy and other perceived food allergens. Those with perceived food allergies underwent open food challenge to specific food allergens. Frequencies and proportions were determined to analyze the different variables.

**Results**: Data were gathered from 84 patients diagnosed with ASD. 32/84 (38%) have perceived food allergies, mostly to milk (31.3%), chocolate (25%), and egg (18.8%). Most commonly perceived allergic reactions to food allergens were: hyperactivity (53.1%), loose stools (25%), pruritus (15.6%), and wheals (12.5%). A total of 17 (20.2%) patients had (+) skin prick test result, hence food sensitization, to at least 1 food allergen, mostly to soy (41.2%) and milk (35.3%). Of these patients, 8 had perceived food allergies. 6 of these patients underwent open food challenge and all of them had (−) results.

**Conclusions**: Prevalences of perceived food allergies and food sensitization are higher among ASD patients in this study compared to the general population. The most common perceived food allergens are similar with that of other children. Notably, the common perceived allergic reactions to food were behavioral or gastrointestinal symptoms, which may be non-IgE mediated or not food allergies at all. Hence, ASD patients with adverse food reactions are recommended to undergo complete, systematic evaluation and possible restrictive diets should be based on well-documented food allergies

### PP031 Food allergy in children of immigrants from Latin America born in Spain in an area of Madrid

#### Maria Luisa Somoza, Natalia Blanca López, Diana Pérez Alzate, Francisco Javier Ruano, Maria Isabel Garcimartín, Elisa Haroun, Maria Vázquez de la Torre, Antonia Rojas, Montserrat López Onieva, Gabriela Canto

##### Allergy Unit, University Hospital “Infanta Leonor”, Madrid, Spain

**Correspondence**: Maria Luisa Somoza - mlsomoza@yahoo.com

*Clinical and Translational Allergy* 2017, **7(Suppl 1)**:PP031

**Introduction**: In 2015, the 11% of the total population in Madrid was foreign, being the Latin American origin the most frequent followed by Asia, East Europe and in fourth place by other EU countries. In our working area, the foreign population average was even higher: 19%. Racial disparities in food sensitization have been described.

The aim of this work was to study the clinical characteristics of food allergy in children of immigrants from Latin American countries born in Spain.

**Methods**: In our study population 474 children reported allergy to food (cow’s milk and hen’s egg allergy not included) between 2012 and 2016. We analyze all the children from Latin American origin who were born in Spain evaluated in our center. Clinical history, skin tests and quantitation of specific IgE antibodies were performed as well as oral food challenges.

**Results**: A group of 29 children were included with an age range of 1–14 y.o., (Mean 7). Most of them were male (69%).Familiar atopy history: Rhinoconjunctivitis/Asthma was the most frequent entity (66%) followed by Atopic Dermatitis (16%). Most of the patients reported a personal history of Rhinoconjunctivitis/Asthma (51%) caused by pollen in 59% of cases; and AD (48%). Concerning food allergy, Latex-fruit group was the most frequent (25%), being Melon the most common, followed by Banana and Kiwi. Treenuts were the second family involved (21%), being Walnut the most common, followed by Peanut. Fruits belonging to the Rosaceae family were also involved (19%), being Peach the most frequent followed by Cherry. Shellfish (17%) was the forth family, Prawn was the most common, and Fish (11%) the fifth family, Hake was the most common. Finally, Legumes (7%) were the sixth group involved in allergy episodes, being Bean the most frequent. Describing Clinical Manifestations, Urticaria/Angioedema was the most frequent entity (59%), OAS the second (35%) followed by Anaphylaxis (4%).

**Conclusions**: In this study, Rhinoconjunctivitis/Asthma was the most frequently reported entity of personal history of atopy, whereas in Spanish children Atopic Dermatitis is the most common. Latex-fruit group was the most frequently involved food in allergy episodes in contrast to what occurs in the Spanish native children, where the fruits of the Rosaceae family are the most common.

### PP032 Eosinophilic esophagitis – The importance of food restriction

#### Alexandra Rodrigues, Andreia Forno, António Jorge Cabral, Rute Gonçalves

##### Department of Pediatrics, Hospital Central do Funchal, Funchal Madeira, Portugal

**Correspondence**: Alexandra Rodrigues - alexandrabrod@gmail.com

*Clinical and Translational Allergy* 2017, **7(Suppl 1)**:PP032

**Introduction**: The eosinophilic esophagitis (EE) is a chronic inflammatory disease, with an immunological component. The relation with food allergy has been reported in a crescent number of studies, demonstrating the key role that food restriction has on symptomatic improvement in these patients.

**Methods**: Retrospective analysis of clinical records of pediatric patients diagnosed with EE, confirmed histologically in our hospital from 2009 to 2015.

**Results**: 7 cases of EE were diagnosed with an average age of 14.5 years, the number of male patients was superior (n = 6). The clinical presentation was complaints of dysphagia and food impaction in all patients, with mean age of presentation at 12.5 years. Patients underwent upper gastrointestinal endoscopy, and the biopsies demonstrated in all patients, the presence of numerous eosinophils (>15 per high power field) in the lamina propria and in transepithelial migration. There was an increase of total IgE (172–877 kU/l) in 4 patients, 3 of whom had positive phadiatop to inhalant allergens. The inhalant allergens were negative in the remaining patients. Food allergens and specific IgE for food were negative. Skin tests (milk, egg, soy, wheat, peanuts, hake, shellfish) were positive for wheat in just one patient, all the others were negative. It was empirically established a restrictive diet in 2 patients, since there was no clinical improvement with drug therapy alone (fluticasone 500 μg bid). The exclusion of cow’s milk, beef, egg and soy in a patient’s diet and cow’s milk, soy and egg on another patient’s diet resulted in a significant clinical and histological improvement.

**Conclusions**: EE pathogenesis seems to be related to atopy and its control should include a dietary component as an adjunct to the pharmacological therapy instituted. Although skin tests may be negative, dietary restriction of certain foods can improve symptoms and histology on these patients.

### PP033 Goat milk formula effectiveness in healthy infants with asymptomatic sensitization

#### Ilya Vorozhko^1^, Tatyana Sentsova^1^, Olga Chernyak^1^, Svetlana Denisova^2^, Lidia Ilènko^2^, Valery Muhortnich^1^

##### ^1^Federal Research Centre of Nutrition and Biotechnology, Moscow, Russia; ^2^Pirogov Russian National Research Medical University, Moscow, Russia

**Correspondence**: Tatyana Sentsova - bio45@inbox.ru

*Clinical and Translational Allergy* 2017, **7(Suppl 1)**:PP033

**Introduction**: Numerous discussions related to the justification for the use of certain formulas in infants with asymptomatic sensitization. The problem is that a balanced and rational nutrition in realization of metabolic programming is necessary for proper growth and development of the child, on the other hand - there is a risk of transition latent sensitization in clinical manifestations of food allergy.

Our aim was to study goat milk formula effectiveness in healthy infants with asymptomatic sensitization.

**Methods**: The study included 110 healthy children aged between 2 and 8 months of age who were bottle-fed. The duration of observation was 6–8 months. Prior to transfer to artificial feeding all children were breast-fed. Children received casein dominant formula based on whole goat milk. ELISA. used to identified allergen-specific IgE antibodies to the protein of cow milk, casein, ß-lactoglobulin, α-lactalbumin and goat milk protein in coprofiltrates.

**Results**: Frequency of latent sensitization to cow milk protein and its fractions, as well as to a protein of goat milk in healthy children was maximal at 2–3 months of age. Observation of the children who received casein based formula showed positive dynamics, which is expressed in reducing allergen-specific IgE antibodies to cow’s milk and its fractions, as well as the goat’s milk to 7–8 months of life.

**Conclusion**: Using goat milk casein based formula was effective in artificial feeding in children with asymptomatic sensitization.

### PP034 CCL17 and CCL20 levels in serum of children with sensitization to hen’s egg in regard to the outcome of the oral food challenge

#### Caroline Zimmermann, Valérie Trendelenburg, Gabriele Schulz, Alexander Rohrbach, Bodo Niggemann, Kirsten Beyer

##### Department of Pediatric Pneumology and Immunology, Charité Universitätsmedizin Berlin, Berlin, Germany

**Correspondence**: Kirsten Beyer - Kirsten.beyer@charite.de

*Clinical and Translational Allergy* 2017, **7(Suppl 1)**:PP034

**Introduction**: Food-specific IgE (sIgE) determines the sensitization to food but oral food challenges are necessary to determine the clinical relevance of a food allergy in most cases. Therefore the identification of other biomarkers could be of great interest. Previously, chemokine levels have been correlated with the severity of eczema and the sensitization status to food allergens. The aim of this study was to analyze if chemokine levels could predict a positive or negative outcome of a food challenge with hen’s egg.

**Methods**: 37 children sensitized to hen’s egg were included in the study. They underwent an oral food challenge with hen’s egg to proof its clinical relevance. Serum chemokines (CCL17 and CCL20) as well as total and sIgE were measured using an ELISA kit (R&D Systems) and the ImmunoCAP250 System^®^ (Phadia) respectively. For statistical analysis the Mann-Whitney-U-test and the Spearman rank correlation were computed.

**Results**: The median age of the children (59.5% male and 40.5% female) was 2.6 years (range 0.8–14.9 years). 89.2% (33/37) of the children had eczema. 19 children (51.4%) had a positive hen’s egg challenge and therefore a clinically relevant hen’s egg allergy whereas 18 children (48.6%) were clinically tolerant. Median sIgE in the allergic patients was 1.9 kU/L (range 0.43–33.9 kU/L) and in the tolerant ones 0.6 kU/L (range 0.03–4.76 kU/L). Hen’s egg allergic children showed significantly higher serum levels of CCL17 (median 655.4 pg/ml; range 183.6–1394.4 pg/ml) compared to tolerant ones (median 501.6 pg/ml; range 172.8–1027.8 pg/ml) (p = 0.030). Furthermore there was a significant correlation of CCL17 with sIgE (r_s_ = 0.377, p = 0.014) and with the ratio of sIgE/total IgE (r_s_ = 0.455, p = 0.004). Regarding the chemokine levels of CCL20 there was no significant difference seen. Our previous studies have shown a strong correlation between serum levels of CCL17 and the severity of eczema in food-sensitized infants. In this study the serum levels of CCL17 could be significantly correlated to the level of hen’s egg-specific IgE and were significantly elevated in children with clinically relevant hen’s egg sensitization in comparison to clinically tolerant ones.

**Conclusions**: CCL17 might not only be a biomarker for severe eczema but also for clinically relevant hen’s egg allergy. However larger studies are necessary to prove these findings. Furthermore, determination of CCL17 will not be able to replace oral food challenge tests.

### PP035 The influence of the order of food challenge days on DBPCFC outcomes in children

#### Faisal R. Bakhsh, Kollen Boudewijn, Anne-Marie Oomkes-Pilon, Dorien Van Ginkle, Anthony E. J. Dubois

##### Department of Pediatric Pulmonology and Pediatric Allergology, University Medical Center Groningen, GRIAC Research Institute, University of Groningen, Groningen, the Netherlands

**Correspondence**: Faisal R. Bakhsh - faisal.r.b@hotmail.com

*Clinical and Translational Allergy* 2017, **7(Suppl 1)**:PP035

**Introduction**: The double-blind placebo-controlled food challenge (DBPCFC) is the most reliable diagnostic test in food allergy for several reasons including reduction of patient and operator or observer bias, thus decreasing the chance of false-positive outcomes. In DBPCFC, the order of the challenges with verum or placebo food are randomized. It is currently unknown whether there is a significant difference when initiating the DBPCFC with a placebo challenge or with an active food challenge.

The study aims to explore whether there is a significant difference when initiating the DBPCFC with a placebo challenge or with an active challenge DBPCFC in children

**Methods**: In this study, a total of 1680 patients who underwent DBPCFC were analyzed. Differences between test day reaction frequencies were assessed with the Mcnemar test. Subjective and objective reactions were considered together and separately. Events occurring on placebo days were also assessed separately.

**Results**: There was no significant difference between the frequency of reactions on test day 1 and day 2 (p-value = 0.160). This was also the case for subjective and objective reactions considered separately (p = 0.640 and 0.193, respectively). When considering only placebo challenges, 58 patients experienced events on the first day and 47 patients on the second day. This difference was significant (p = 0.000).

**Conclusions**: Patients tend to experience spurious events more often during the first challenge day than the second challenge day, perhaps because of pre-conceived notions about the test and greater anxiety. However, this has no significant impact on the overall diagnostic accuracy of the test, even in patients where the diagnosis is made on the basis of subjective reactions.

### PP037 Basophil activation test and cow’s milk or egg clinical tolerance

#### Mira Šilar^1^, Anja Jeverica^2^, Tina Vesel^2^, Tadej Avčin^2^, Peter Korošec^1^

##### ^1^University Clinic of Respiratory and Allergic Diseases, Golnik, Slovenia; ^2^Department of Allergology, Rheumatology and Clinical Immunology, University Children’s Hospital, University Medical Center Ljubljana, Ljubljana, Slovenia

**Correspondence**: Mira Šilar - mira.silar@klinika-golnik.si

*Clinical and Translational Allergy* 2017, **7(Suppl 1)**:PP037

**Introduction**: Outgrowing of cow’s milk or egg allergy might be related to differences in IgE-dependent basophil function. We sought to investigate if the level of allergen specific basophil response is associated with the clinical tolerance among a cohort of children with cow’s milk or egg allergy.

**Methods**: Sixty children were classified as milk or egg-reactive and milk or egg- outgrowing (tolerant) based on the results of oral food challenges (38 milk and 22 in egg group). Serum was analysed for allergen-specific IgE and IgG levels, basophil response was assessed in whole blood stimulated with serial dilutions of milk or egg white and yolk protein (all 0.33–33.3 ng/ml) and with anti-FcεRI stimuli. Skin prick tests (SPTs) to commercial extracts were also performed.

**Results**: Milk-reactive subjects’ basophils showed significantly higher response (median fourfold, at 33.3 ng/ml) to milk allergen stimulation than basophils from milk- tolerant individuals. There was also a significantly higher response to anti-FcεRI stimuli (twofold). On the other hand, in egg group no significant differences in egg white, yolk or anti-FcεRI basophil response, in sIgE to egg white or yolk, and in SPT were demonstrated between egg-reactive versus egg-tolerant children. However in egg-tolerant children we demonstrated a significantly higher level of specific IgG to egg white.

**Conclusions**: We showed that children with outgrowing versus reactive cow’s milk allergy can be distinguished by basophil activation test. These observations could not be confirmed for children with egg allergy.

### PP038 Epinephrine treatment of anaphylactic reactions during food challenges in children

#### Johanna van der Valk^1^, Irene Berends^1^, Roy Gerth van Wijk^1^, Nicolette Arends^2^, Maurits van Maaren^1^, Hans de Groot^3^, Harry Wichers^4^, Joyce Emons^2^, Anthony Dubois^5^, Nicolette de Jong^1^

##### ^1^Department of Internal Medicine, Allergology, Erasmus Medical Center, Rotterdam, the Netherlands; ^2^Department of Paediatric Allergology, Erasmus Medical Center Sophia Children’s Hospital, Rotterdam, the Netherlands; ^3^Department of Pediatric Allergology, Diaconessenhuis Voorburg, RdGG, Delft, the Netherlands; ^4^Wageningen UR Food and Biobased Research, Wageningen, the Netherlands; ^5^Department of Pediatric Pulmonology and Pediatric Allergology, University Medical Center Groningen, GRIAC Research Institute, University of Groningen, Groningen, the Netherlands

**Correspondence**: Johanna van der Valk - j.p.m.vandervalk@erasmusmc.nl

*Clinical and Translational Allergy* 2017, **7(Suppl 1)**:PP038

**Introduction**: The primary aim of this study is to evaluate the percentage of anaphylactic reactions treated with epinephrine during a food challenge test and to identify associated factors for the administration of epinephrine.

**Methods**: Children who underwent a food challenge test with peanut, hazelnut, cow’s milk, hen’s egg or cashew nut in the period 2005 to 2015 at the department of Allergology Erasmus Medical Center Rotterdam in the clinical setting or in a research setting (IDEAL-study, collaboration of three tertiary care centers for food allergy, trial number NTR3572) were evaluated. The children with reactions meeting the criteria for anaphylaxis according to the EAACI Guidelines for Food Allergy and Anaphylaxis and/or who were treated with epinephrine were included. Possible factors associated with the administration of epinephrine such as age, gender, symptoms consistent with asthma, history of an allergic reaction to the tested allergen and the type of symptoms during the anaphylactic reaction were investigated with logistic regression analysis.

**Results**: A total of 92 children (40 boys, 44%) with a median age of 7 years (range 1–17 years) who met the criteria for anaphylaxis (n = 85) or who were treated with epinephrine (n = 7) were included in this study. Thirty-three children (39%) with anaphylaxis were treated with epinephrine. Factors significantly associated with epinephrine treatment were younger age (p = 0.001) and lower airway symptoms (p = 0.001). Gastro-intestinal symptoms and a history of an allergic reaction to the tested allergen were significantly associated with the lack of epinephrine treatment (p < 0.001 and p = 0.043, respectively). The most severe allergic type of reaction during a food challenge test is anaphylaxis, for which the recommended treatment is epinephrine. However, only about half of the children with anaphylaxis received epinephrine during the food challenge tests in our study. Treatment of anaphylaxis during food challenge test does not correspond to the indication for such treatment as delineated in the EAACI Guidelines. Further analysis is warranted to ascertain the cause of this discrepancy.

**Conclusions**: Anaphylaxis occurring during challenge tests seems to be undertreated with epinephrine.

### PP039 Analysis of dietary programmes for infants with allergy signs

#### Oksana Matsyura, Lesya Besh

##### Danylo Halytsky National Medical University, Lviv, Ukraine

**Correspondence**: Oksana Matsyura - omatsyura@gmail.com

*Clinical and Translational Allergy* 2017, **7(Suppl 1)**:PP039

**Introduction**: Nowadays allergies are among the most common of medical disorders. It is estimated that more than one in every five people suffer from some form of allergy in the world. Allergic pathology in children is usually manifested by food allergy (FA). This problem starts in infancy and is most likely to arise when atopic diseases run in the family. Food allergens are major components of sensitization structure in children under one year. Our purpose wat to select high-adapted nutrition mixtures for infants with allergic manifestations.

**Methods**: 116 artificially fed infants with the signs of allergic constitutional dermatitis and children’s eczema. Programme contemplated estimating efficiency of the nutrition mixture. “NAN Lactose free” was administered as main nutrition for 78 and “NAN Soy”—for 38 infants. If they proved ineffective “Acidophilic NAN” and “Alfare” were given. Adequacy of nutrition was determined by the dynamics of skin status and simultaneous analysis of the character of sleep, presence of gastro-intestinal changes, microbiological profile of feces, and specific allergodiagnosis *in vitro*.

**Results**: Soy-bean nutrition proved effective in 56%. “NAN Soy” combined with “Acidophilic NAN” led to stabilization of intestinal motility in 80% of infants with a good tolerance to this mixture. Application of “NAN Lactose free” caused positive dynamics in 63%. The rest (37%) were transferred successfully to a combined nutrition “NAN Soy” + “Acidophilic NAN”. In case “NAN Soy” and “NAN Lactose free” (33%) were ineffective, mixture “Alfare” or its combination with “Acidophilic NAN” were tested. Positive dynamics of FA course was observed in all infants.

**Conclusions**: Thus, at the absence of breast milk, infants with FA manifestations are expedient to feed with the following mixtures:

- with complete protein hydrolysis (Alfare);
- acidophilic (Acidophilic NAN);
- alactic (NAN Lactose free);
- soy-bean (NAN Soy).

### PP040 Oral administration with *Lactobacillus reuteri* attenuated food allergic responses and enhanced the reactivity of regulatory T cells in BALB/c mice

#### Chung-Hsiung Huang^1,2^, Tong-Rong Jan^2^

##### ^1^National Institute of Infectious Diseases and Vaccinology, National Health Research Institutes, Miaoli, Taiwan; ^2^Department and Graduate Institute of Veterinary Medicine, School of Veterinary Medicine, National Taiwan University, Taipei, Taiwan

**Correspondence**: Chung-Hsiung Huang - 030506@nhri.org.tw

*Clinical and Translational Allergy* 2017, **7(Suppl 1)**:PP040

**Introduction**: Our aim was to investigate the anti-allergic and immunomodulatory effects of *Lactobacillus reuteri* in a murine model of food allergy.

**Methods**: BALB/c mice were sensitized with ovalbumin (OVA) plus alum and subsequently challenged with OVA by gavage to induce food allergy. The mice were daily administered with *L. reuteri* (1 x 10^9^ CFU/mouse) and/or MRS broth (as vehicle) throughout the entire period of experiment. Allergic diarrhea was monitored after each OVA challenge, and the mice were sacrificed post the last OVA challenge to collect serum samples, spleen and duodenal tissues for immunological and histopathological analysis.

**Results**: *L. reuteri* administration attenuated the occurrence of diarrhea, intestinal mast cell activation, and serum IgE production. Furthermore, both the production of IFN-γ and IL-4 by splenocytes was suppressed by *L. reuteri*. Concordantly, a decreased expression of IL-4, IFN-γ, GATA3 and T-bet were observed in the duodenum. However, the expression of IL-10, TGF-β and Foxp3 was augmented. These findings demonstrate that oral administration with *L. reuteri* attenuated allergic responses and down-regulated both T helper (Th)1 and Th2 immune responses, which was closely associated with the enhanced reactivity of regulatory T cells.

**Conclusions**: *L. reuteri* may be used as a functional probiotic for managing intestinal disorders associated with exaggerated immune responses, especially food allergy.

### PP041 Questionnaire Survey of the infrastructure for managing allergy in schools around Leicester, UK

#### Gary Stiefel^1^, Jean Tratt^2^, Kerrie Kirk^1^

##### ^1^Leicester Royal Infirmary, Leicester, United Kingdom; ^2^Leicester Partnership Trust, Leicester, United Kingdom

**Correspondence**: Gary Stiefel - garystiefel@icloud.com

*Clinical and Translational Allergy* 2017, **7(Suppl 1)**:PP041

**Introduction**: The children’s allergy service in Leicester continues to work with local authorities (LAs) to ensure there are guidelines in managing allergy, including emergency action plans. The aim of this survey was to ascertain whether school staff had the appropriate infrastructure in place to manage children with allergies.

**Methods**: Two LAs were sent a link to an online questionnaire to all schools under their jurisdiction (377 schools). The schools were requested to circulate the questionnaire to all members of staff to complete.

**Results**:

- 234 staff members completed the survey. Off those 166 taught children <11 yrs old.
- 86.2% of staff state there is a school policy relating to managing allergy.
- The responsibility for checking the expiry dates of Adrenaline autoinjectors (AAIs) lies with parents (37.9%), administration staff (23.7%), teacher (12.9%), other (23.8%), Unsure/no procedure (1.7%). 85.1% of respondents maintain a clear log with 65.8% checking expiry dates at least once a term.
- AAIs are kept with the child (19.2%), unlocked central location (36.2%), locked central location (25.8%), Classroom (37.1%)
- AAIs are taken on the school playing fields 52% of the time, but 96.5% of the time for visits off the school premises.
- Schools request the following number of AAIs per child; one AAI (55.8%), two AAI (24.3%) and leave it for the doctor to decide (19.9%).

**Conclusions**:

- Most schools have policies in place relating to managing allergy in schools.
- More guidance surrounding storage of AAI and checking expiry date may prevent out of date AAIs as well as AAIs being locked away, which in an emergency could prove problematic.
- Schools appear to be influencing AAI prescription practices by specifying numbers of AAIs they require.
- Ongoing partnership between the allergy clinic, LAs and school nurses are required to further improve the infrastructure for managing allergy in schools.

### PP042 Do school staff receive training and feel confident in the recognition and management of allergic reactions?

#### Gary Stiefel^1^, Jean Tratt^2^, Kerrie Kirk^1^

##### ^1^Leicester Royal Infirmary, Leicester, United Kingdom; ^2^Leicester Partnership Trust, Leicester, United Kingdom

**Correspondence**: Gary Stiefel - garystiefel@icloud.com

*Clinical and Translational Allergy* 2017, **7(Suppl 1)**:PP042

**Introduction**: The children’s allergy service in Leicester has worked with local school nurses to produce educational material on the recognition and treatment of allergic reactions. School nurses deliver this package to school staff. The aim of this survey was to ascertain whether staff felt they were able to manage children presenting with an allergic reaction.

**Methods**: 377 schools under the jurisdiction of two local education authorities were sent a link to an online questionnaire. The schools were requested to circulate the questionnaire to all members of staff to complete.

**Results**:

- Training for the management of allergic reactions was provided by a LEA school nurse (75.1%), first aid training provider (16.7%) and other including no training (8.2%)
- 75.4% of school staff had received training in recognition and management of allergic reactions within the last 12 months, 14.2% within 12–24 months and 10.4% >24 months.
- 91.1% felt confident in the recognition of an allergic reaction, while 89.3% felt confident in being able to manage an allergic reaction, including the use of an AAI.
- 30.4% and 22.9% respectively felt they needed more training on recognition of an allergic reaction and administration of an AAI.

Nearly all members of staff completing the survey had received training within the last 2 years and feel confident in the recognition and management of allergic reactions. Significant numbers of staff feel further training would be beneficial.

**Conclusions**: Continued resources are required to maintain staff training in managing allergy. Additional training resources such as DVD, trainer pens & useful websites should be provided with the current training package to provide ongoing training.

## POSTER SESSION 2: Management of food allergy

### PP043 Food avoidance after negative challenge in children

#### Luis Amaral, Leonor Carneiro-Leão, Fabricia Carolino, Diana Silva, Alice Coimbra

##### Serviço de Imunoalergologia, Centro Hospitalar de São João E.P.E., Porto, Portugal

**Correspondence**: Luis Amaral - luis.m.amaral@gmail.com

*Clinical and Translational Allergy* 2017, **7(Suppl 1)**:PP043

**Introduction**: A negative oral food challenge (OFC) should normally be followed by its reintroduction in the diet. However, this fails in a subset of children.

Aims: (1) To analyse the rate of occurrence and the reasons for failure of reintroduction of the implicated food after negative OFC in children; (2) To determine the proportion of food avoidance in children who did not undergo OFC.

**Methods**: A retrospective study of children with an OFC scheduled between January 2012 and December 2015 was performed. Clinical records were reviewed and the food consumption status (FCS) was obtained using a short structured phone or paper enquiry answered by the caregivers.

**Results**: Data of 127 children (59% male; median age [interquartile range, IQR] 5 [7] years); 64% atopic (48% allergic rhinitis; 38% asthma and 27% atopic eczema); was collected. A total of 235 OFC were scheduled during this period and only 186 were performed and 137 were negative. The major foods tested were with cow’s milk (29%), hen’s egg (26%), fish (14%) and crustaceans (11%). Data on FCS was obtained in 97.3% of the total 235 OFC scheduled during the study period. The mean time (±std) between the OFC and the assessment of FCS was 13 ± 6.9 months. Food avoidance was maintained in 26.3% of the 137 negative OFC. Food rejection was the main reason (44%), followed by the fear of reaction by caregivers (35%). Food avoidance was related with the suspected food group (p < 0.01). Further comparison of children’s FCS revealed no significant differences with respect to gender (p = 0.651), age (p = 0.07), clinical manifestation of the index reaction (p = 0.627) or the presence of allergic comorbidities (p = 0.133). Eviction of the suspected food was maintained in 61.2% of the 49 OFC scheduled that were not performed, in contrast to the 37.1% when an OFC was performed, independently of its result; p < 0.001.

**Conclusions**: Avoidance of the offending food is still the mainstay of management and so, children with suspected food allergy often present with a long-lasting elimination diet. Despite a negative challenge outcome and advice to re-introduce the food in the diet, 26.3% of the children were still avoiding the implicated food. Almost two thirds (61.2%) of the patients who did not undergo OFC maintained avoidance of the suspected food. These data highlight the importance of performing an OFC in preventing unnecessary restrictive diets as well as alert to the need of assessing food consumption after a negative OFC.

### PP044 Omalizumab combined with oral immunotherapy for the treatment of severe cow’s milk allergy: our 2-year-long experience

#### Stefania Arasi, Lucia Caminiti, Giuseppe Crisafulli, Chiara Fiamingo, Jlenia Fresta, Giovanni Pajno

##### Allergy Unit, Department of Pediatrics, University of Messina, Messina, Italy

**Correspondence**: Stefania Arasi - stefania.arasi@yahoo.it

*Clinical and Translational Allergy* 2017, **7(Suppl 1)**:PP044

**Introduction**: Oral immunotherapy (OIT) combined with omalizumab (O) can be an effective and safe approach treatment to rapid desensitization in children with severe cow’s milk allergy (CMA). We report our experience.

Objective: To evaluate the safety and efficacy of OIT combined with O.

**Methods**: Volunteers with severe CMA that had previously interrupted a conventional OIT because of dose—related severe anaphylactic reactions were enrolled. Informed written consent was obtained by the respective parents before the treatment. As off label treatment, O was administered according to the package insert for asthma treatment. Each pts undertook s.c. injections of O for 8 weeks prior to and 8 weeks following the initiation of OIT. On the initial escalation day, dosing began at 1.5 ml of cow’s milk (CM, 5 mg of proteins) and were slowly increased until the pt reached a final dose of 200 ml or if any adverse event (AEs). The highest tolerated dose (i.e. with no clinical reactivity) determined the pt’s starting daily home dose. The pts returned to the clinic every 2 weeks for a dose escalation visit. OIT protocol did not advance according to a fixed calendar, but, rather were individualized according to pts’ allergic reactions and safety outcomes. After the up-dosing regimen the maintenance phase was performed (200 ml of CM daily and dairy products ad libitum).

**Results**: We have enrolled 6 children (n male = 4), aged 9.8 ± 2.31 yrs (mean ± SD) suffering from severe CMA. 4 children had concomitant allergic asthma (A) and one of the latter atopic dermatitis, too. *Efficacy*: 2 pts reached the maintained dose of CM 200 ml after 17 weeks; 1 reached the dose of 150 ml of CM and dairy products after 7 months. All of them are going on daily maintenance intake since 12 months. 3 pts interrupted OIT during the build- up phase: 1 for severe AE (2 ml CM); 1 for concomitant severe A; 1 for personal problem. *Safety*: 2 pts developed severe anaphylactic reactions after dose intake: 1 at the increasing dose of 2 ml [rhinitis (R), cough, A, angioedema-urticaria (U), hypotension]; 1 at the maintenance dose of 150 ml (U&A). All of them have history of A. The same 2 pts developed mild AEs during the induction phase of OIT: 1 reacted at the 8 ml dose (R) and 25 mL dose of CM (U); another at 150 ml of CM (R&U).

**Conclusions**: This combined approach could allow for a more successful and safer desensitization in children with high-risk CMA that had failed a conventional OIT protocol. However, in pts with concomitant A, it could be less effective.

### PP046 Risk assessment of unintended allergens in food: can we use food consumption data from the general population?

#### Ben Remington, Astrid Kruizinga, Marty Blom, Geert Houben

##### The Netherlands Organization for Applied Scientific Research (TNO), Zeist, the Netherlands

**Correspondence**: Ben Remington - ben.remington@tno.nl

*Clinical and Translational Allergy* 2017, **7(Suppl 1)**:PP046

**Introduction**: Risk is a function of hazard and exposure. For food products, allergens are a well-known hazard and exposure scenarios are calculated based on the concentration of unintended allergen in combination with the amount of food consumed. At the moment, food consumption data from the general population are used for calculating exposure scenarios in food allergen risk assessments. A reasonable assumption is that if a food product is chosen to be consumed, the population distributions of amounts of a food product consumed at one eating occasion are comparable between the allergic population and the general population. A second assumption is that the frequency of consumption for certain product categories may differ between the allergic and general populations. However, structured data are lacking to underpin these assumptions and research initiatives are needed to fill in the current data gaps.

**Methods**: Based on the US National Health and Nutrition Examination Survey, a statistical comparison was made between the food consumption distribution at a single eating occasion of allergic and non-allergic individuals. Two allergic identifiers were studied, serum IgE to the specific food (NHANES 2005–2006) and self-indicated food allergy (NHANES 2009–2010). The food allergens studied were: shrimp, wheat, cow’s milk, egg, fish, shellfish, peanut and other nuts. Food consumption was compared at the food group level. A total of 103 food groups were defined. The statistical difference was tested with ANOVA or Chi squared test.

**Results**: Overall, the results indicated that the criteria for allergy were not strong enough in the NHANES survey and that non-allergic individuals were mixed with the allergic population.

**Conclusion**: It was not possible to make a conclusion about the validity of the assumptions applied in risk assessment when using food consumption data from the general population. More comprehensive and structured studies are needed to ascertain the differences in consumption patterns of allergic and non-allergic individuals. Ideally, any future study should identify clinically-diagnosed food-allergic individuals and measure their food consumption patterns using the same method as the general population.

### PP047 Food allergy population thresholds: 2011–2016 update of international DBPCFC database for food allergen risk assessment

#### Ben Remington^1^, Joost Westerhout^1^, Marty Blom^1^, Astrid Kruizinga^1^, Sabina Bijlsma^1^, Steve Taylor^2^, Geert Houben^1^, Joe Baumert^2^

##### ^1^The Netherlands Organization for Applied Scientific Research (TNO), Zeist, the Netherlands; ^2^University of Nebraska, Lincoln NE, USA

**Correspondence**: Ben Remington - ben.remington@tno.nl

*Clinical and Translational Allergy* 2017, **7(Suppl 1)**:PP047

**Introduction**: In early 2011, the VITAL^®^ (Voluntary Incidental Trace Allergen Labeling) Scientific Expert Panel of The Allergen Bureau of Australia & New Zealand (ABA) reviewed individual NOAELs and LOAELs obtained from over 1800 clinical food challenges in an effort to guide advisory labeling decisions for use on food labels. Reference Doses were established for 11 allergenic foods including peanut, cow’s milk, egg, hazelnut, soybean, wheat, cashew, shrimp, sesame seed, mustard and lupine (in terms of mg of total protein). Reference Doses were not established for fish, celery or other tree nuts due to a lack of sufficient quality data at the time. As part of the VITAL^®^ update process, the threshold database was supplemented with additional data collected from 2011 to 2016. The results of that update are presented here.

**Methods**: Individual NOAELs and LOAELs from published clinical literature were reviewed and verified by scientists at the Food Allergy Research & Resource Program (FARRP) at the University of Nebraska and at TNO in the Netherlands. Publications from 2011 to 2016 were selected based upon previously outlined criteria and focused on low-dose oral food challenges. Additional unpublished clinical records from TNO and FARRP collaborators were also used to supplement the published data. Where possible, population based threshold distributions were updated.

**Results**: Over 1300 data points were added from data made available during 2011–2016. The TNO-FARRP allergen threshold database now contains over 3100 individual NOAELs and LOAELs from clinical food challenges for 32 allergens, including 15 priority allergens from different international labelling regulations. However, limited data restricted distribution calculations for a number of non-priority foods. In general, the newly calculated population threshold distributions did not significantly vary for a majority of allergens between the 2011 and 2016 datasets.

**Conclusions**: The results from this study indicate that the population thresholds generated for most VITAL^®^ allergens are stable and were not significantly altered with the addition of new data. These results should enable government and food industry risk managers to proceed with allergen risk management systems based on the VITAL^®^ reference doses.

### PP049 Threshold dose distribution in walnut allergy

#### Mark Blankestijn^1^, Ben Remington^2^, Geert Houben^2^, Joe Baumert^3^, Henny Otten^1^, André Knulst^1^, Rob Klemans^1^, Steve Taylor^3^

##### ^1^University Medical Center Utrecht, Utrecht, the Netherlands; ^2^The Netherlands Organization for Applied Scientific Research (TNO), Zeist, the Netherlands; ^3^Food Allergy Research & Resource Program (FARRP), Lincoln NE, USA

**Correspondence**: Mark Blankestijn - m.a.blankestijn@umcutrecht.nl

*Clinical and Translational Allergy* 2017, **7(Suppl 1)**:PP049

**Introduction**: Our aim was to determine eliciting doses (EDs) in walnut allergic adults and to compare with previously established threshold data in peanut and tree nuts.

**Methods**: Prospectively, adult subjects with a suspected walnut allergy were included and underwent a low-dose double-blind, placebo-controlled food challenge (DBPCFC). Individual no observed and lowest observed adverse effect levels (NOAELs/LOAELs) were determined and Log-Normal, Log-Logistic and Weibull models were fit to the data. Estimated eliciting dose (ED) values were calculated for the ED5, ED10 and ED50, the dose respectively predicted to provoke an allergic reaction in 5, 10 and 50% of the walnut allergic population.

**Results**: Fifty-seven subjects were challenged and 33 subjects were confirmed to be walnut allergic. Objective symptoms occurred in 20 of the positive challenges (61%), varying from angioedema of the lip to severe dyspnea. The lowest cumulative LOAEL for objective symptoms was 0.31 mg of walnut protein, leading to repeated coughing in one subject. Data from 13 subjects with only subjective symptoms were right censored. The cumulative eliciting doses in the three distribution models ranged from 3.1 to 4.1 mg for the ED05, from 10.6 to 14.6 mg walnut protein for the ED10 and from 590 to 625 mg of walnut protein for the ED50.

**Conclusions**: Population EDs for walnut are slightly higher compared to those previously found in peanut and hazelnut allergy. Additionally, previous ED estimates for cashew from a limited number subjects (31) was also higher when compared to hazelnut (202 subjects), indicating that threshold levels for hazelnut could be used as a conservative estimate for risk assessment of other tree nuts where little or no food challenge data is available.

### PP050 Unexpected allergens and food products causing allergic reactions in daily life

#### W. Marty Blom^1,2^, Anouska D. Michelsen-Huisman^2^, Harmieke van Os-Medendorp^2^, Astrid G. Kruizinga^1^, Astrid Versluis^2^, Gert van Duijn^3^, H. Mary-Lene de Zeeuw-Brouwer^1^, Jacqueline J. M. Castenmiller^4^, Hub P.J.M. Noteborn^4^, André C. Knulst^2^, Geert F. Houben^1,2^

##### ^1^The Netherlands Organization for Applied Scientific Research, Zeist, the Netherlands; ^2^Department of Dermatology/Allergology, University Medical Center Utrecht, Utrecht, the Netherlands; ^3^Triskelion, Zeist, the Netherlands; ^4^NVWA, Netherlands Food and Consumer Product Safety Authority, Utrecht, the Netherlands

**Correspondence**: W. Marty Blom - marty.blom@tno.nl

*Clinical and Translational Allergy* 2017, **7(Suppl 1)**:PP050

**Introduction**: Unexpected allergic reactions to food are frequently occurring, with even severe and fatal reactions.

Our aim was to analyze the type of food products and possible presence and levels of allergens involved in unexpected reactions in daily life of patients.

**Methods**: A prospective cohort study in adults with a doctor diagnosed food allergy. Patients reporting an unexpected allergic reaction were asked to provide information on the food product, the label and estimated consumed amount, and a sample to determine the possible culprit allergenic substances and their concentrations. This was combined with individual patient food allergies and products were analysed using ELISA or qPCR for different allergens. Results were analyzed and compared with the Reference Doses as established by Taylor et al (Food and Chemical Toxicology 63, 2014, 9–17).

**Results**: Patients experiencing an unexpected allergic reaction reported a very diverse range of food products. A significant part of the unexpected reactions, 78% (118 out of 151), was attributed to a specific product by the patients. In 22% the reaction was caused by a composite meal. A total of 53 food samples were received and analyzed for 28 different allergens, ranging from 1 to 15 allergens per product, and on average 5–6 measurements per product. In 40% of the products 1 to 4 allergens were detected per product, other allergens analyzed were not present or under the limit of detection. In 60% of the received samples no unexpected allergenic substance was detected. Levels ranged from 0.2 ppm up to 5000 ppm allergen protein/kg food product. The levels of peanut, hazelnut, sesame, cow’s milk and hen’s egg were all above action levels determined using the Reference Doses. Walnut, pecan nut, cashew nut and celeriac were also detected. The severity of the reactions for the 21 products ranged from mild to moderate (Muller score 0–3). In part of the unexpected reactions it was difficult to attribute to a specific food product or allergen. Reasons could be that meals consisted of multiple products, allergens might not be present or are not equally distributed in the food, or the wrong product was provided by patients.

**Conclusions**: Levels of unexpected allergen and estimated doses taken by patients did not exceed the Reference Doses for precautionary labeling as described in Taylor et al 2014.

### PP051 Home based oral immunotherapy with baked egg protocol

#### Kristian Bravin, David Luyt

##### University Hospitals of Leicester NHS Trust, Leicester, United Kingdom

**Correspondence**: Kristian Bravin - kristian.bravin@uhl-tr.nhs.uk

*Clinical and Translational Allergy* 2017, **7(Suppl 1)**:PP051

**Introduction**: Hen’s egg (HE) allergy is one of the commonest food allergies in young children with a natural history to resolve, although recent evidence suggests a greater prevalence and more persistent nature. Treatment strategies of allergen avoidance anticipating that the child will grow out of the allergy are being replaced by interventions like oral immunotherapy (OI). The recipes used in HE OI are however in forms of egg not commonly ingested and are therefore considered experimental and not suitable for widespread clinical application.

**Methods**: We developed a recipe of baked egg biscuits for use in HE OI and implemented a home-based slow up-dosing programme. Five recipe stages were provided to create enough doses to allow gradual increases in allergen exposure. Eligible subjects were recruited at routine allergy clinic visits. Persisting HE allergy was confirmed mostly by recent contact symptoms or, if not, by a positive baked egg challenge. As allergic symptoms are frequent in OI, families had ready access to dietetic advice on dosing and symptom treatment.

**Results**: Fifteen subjects (9 boys) age from 6 to 17 years (median 11 years 2 months) were treated. Eight achieved full tolerance (equivalent to one egg), 5 became partially tolerant allowing them to include dietary traces and 2 failed. Allergic symptoms during OI were mild with oral pruritus, abdominal pain, nausea and vomiting and urticaria or eczema flares, successfully treated with antihistamines.

**Conclusions**: We have demonstrated the efficacy and safety of a HE OI programme using an easy-to-make baked egg recipe. As the biscuit form of egg presentation is widely ingested, we propose the recipe to enable HE OI be more widely clinical available.

### PP052 Development of a controlled vocabulary for oral food challenges in the EuroPrevall and iFAAM data dictionary to allow semantic interoperability in Allerg-e-lab

#### Bushra Javed^1,2^, Phil Couch^3^, Christopher Munro^3^, Phil Padfield^1,2^, Matt Sperrin^3^, Angela Simpson^1,2^, Clare Mills^1,2^

##### ^1^Manchester Institute of Biotechnology, University of Manchester, Manchester, United Kingdom; ^2^Institute of Inflammation and Repair, University of Manchester, Manchester, United Kingdom; ^3^Institute of Population Health, University of Manchester, Manchester, United Kingdom

**Correspondence**: Bushra Javed - bushra.javed@postgrad.manchester.ac.uk

*Clinical and Translational Allergy* 2017, **7(Suppl 1)**:PP052

**Introduction**: Allerg-e-Lab is a semantic web service initiative for the documentation and exchange of information related to food allergies. The data dictionary—controlled medical vocabulary is essential to identify and characterise the semantic relationship of variables and is key to interpreting data. This study will analyse and compare existing standard terminologies used to describe oral food challenges, and develop a structured approach to collating meta data required for modelling dose distributions from clinical data from double blind placebo controlled food challenges.

**Methods**: Data harmonization - integrating data into the Allerg-e-Lab is a five step process which is being based on the DataSHaPER approach (quality, quantity, and harmony) used for bio clinical studies. The data annotation tools (software and user interface) are being used to annotate data, attaching metadata, and referring to terms from a controlled vocabulary for importing EuroPrevall and iFAAM study data sets into the Allerg-e-Lab platform.

**Results**: The initial focus for developing the EuroPrevall-iFAAM data dictionary has been to develop a controlled medical vocabulary which describes the protocols and clinical symptoms recorded during food challenges undertaken in the EuroPrevall-iFAAM studies. Differences in protocols and definitions between the two projects will be described, and their application to developing data sets to allow comparison in the development of dose distributions based on pooled data described.

**Conclusions**: The EuroPrevall-iFAAM data dictionary will be implemented within the Allerg-e-lab environment and used to investigate the effect of age on threshold dose distributions for peanut, egg and milk from the EuroPrevall and iFAAM projects.

### PP053 Introducing egg containing foods step by step

#### Aideen Byrne^1^, Lizalet Oosthuizen^1^, Carina Kelleher^1^, Fiona Ward^1^, Niamh Brosnan^2^

##### ^1^Our Lady’s Children’s Hospital Crumlin, Dublin, Ireland; ^2^School of Biological Sciences, Dublin Institute of Technology, Dublin, Ireland

**Correspondence**: Aideen Byrne - aideen.byrne@olchc.ie

*Clinical and Translational Allergy* 2017, **7(Suppl 1)**:PP053

**Introduction**: The Irish Food Allergy Network (IFAN) created the IFAN egg ladder, along which increasingly lightly cooked egg can be introduced into the diet of egg allergic children. Our hospital allergy clinic has been providing, step by step instructions and recipes to parents of egg allergic children, considered appropriate for home introduction. The purpose of this study was to retrospectively examine the success and safety of this practice.

**Methods**: 139 children, who commenced home introduction of baked egg were identified as suitable candidates. A retrospective chart review was performed and a questionnaire was completed by parents via telephone interview.

**Results**: 75 cases were included in the study. Only 5 had not introduced any form of egg; 4 of these due to refusal by the child. Almost 50% deviated from provided instructions. 41 (54.7%) reported a reaction during home challenge. 35 (46.7%) had immediate reactions with n = 17 given an anti-histamine, and 1 patient seen by a GP. 6 reported worsening eczema. Only 1 subject had a severe reaction. 60% of patients introduced egg in forms less baked than cake or muffin, such as pancake, egg pasta, egg noodle and hardboiled egg. 54.7% stated that finding time to bake goods was a barrier to introduction. More regular ingestion of egg was associated with greater success. The number of egg products in the diet was directly associated with increased peace of mind socialising, reduced worry and increased meal options. Home introduction of egg containing products at an earlier age may be important. The process is associated with allergic reactions, primarily mild. Increased egg introduction appears to improve aspects of QoL. Simplifying challenges to include bought produce needs to be considered.

**Conclusions**: Home introduction of egg containing food in a step by step manner is achievable and allows for provision of options for children, more appealing to parents than cake.

### PP054 Primary prevention of nut allergy in children – Is there a “LEAP” between research and reality?

#### Graham King, Eva Corbet, Aideen Byrne

##### Allergy Department, Our Lady’s Children’s Hospital Crumlin, Dublin, Ireland

**Correspondence**: Aideen Byrne - aideen.byrne@olchc.ie

*Clinical and Translational Allergy* 2017, **7(Suppl 1)**:PP054

**Introduction**: The recently published *Consensus Communication on Early Peanut Introduction* recommends *at*-*home* introduction of peanut into diets of skin prick test negative, high risk (egg allergy and/or severe eczema) infants. The communication states that, if deemed appropriate, in-office observed peanut ingestion can be performed before initiating at-home ingestion. We sought to examine the success of home introduction of peanut in our clinical cohort.

**Methods**: Our study was a two cycle audit; the first cycle consisted of verbal instruction to all parents of non-sensitised high risk children to introduce at least 6 g of peanut protein per week with a follow up phone-call; the second cycle (for parents of children who had not started peanut) included a *repeat* skin prick test and a meeting with a member of the clinical team to address parental concerns. Written instructions were also provided. Parents were subsequently contacted by phone one month later.

**Results**: 25 of 38 families (66%) introduced peanut and 13(34%) did not; Infants successfully commenced on peanut consumed a median 2.5 g of peanut protein per week. 2 parents who had their own diagnosis of peanut allergy requested hospital observed introduction. The remaining 11 non-ingestors returned to clinic. Repeat skin test was negative in each case. Each agreed to home introduction. 10 were contactable. 5(50%) had introduced peanut. Post-intervention a median 4 g of peanut protein was consumed per week. Nearly 80% (79%) of parents introduced peanut at home without an initial hospital challenge. Medians of 2.5 g & 4 g of peanut protein were consumed per week in our study; the LEAP study reported a median 7.7 g consumption. Reasons (Table 2) for non-introduction show *peanut refusal* as a major issue. The LEAP study addressed this with regular telephone calls, food frequency questionnaires and extra clinics. Our limited resources hinder such high level of supervision.

**Table 2:** Reasons given for not introducing peanut at home (13 patients)

Patient	Initial reason	Post intervention reason
1	Child refused peanut butter	Refusing peanut butter (even hidden)
2	Child refused peanut butter	Acid reflux and so excluded all dairy/egg/nut from diet
3	Child refused peanut butter	
4	Refused peanut butter and sibling has nut allergy	
5	Parents prioritised home based graded baked milk exposure	
6	Parents prioritised home based graded baked egg exposure	Refusing peanut butter (even hidden)
7	Parents prioritised home based graded baked egg exposure and suspected eczema worsening	
S	Did not understand advice given at initial clinic visit	
9	Did not understand advice given at initial clinic visit	
10	Sibling has peanut allergy	Parental decision to maintain nut free house
11	Parent has peanut allergy (*subsequently requested clinic based introduction*)	
12	Parent has suspected peanut allergy (*subsequentlyrequested clinic based introduction*)	
13	Maternal anxiety	Parental decision

**Conclusions**: Initiation of peanut ingestion by home introduction is successful in most cases. Clear written instructions, including tips on how to conceal are likely to improve compliance. Early follow up to identify those unable to introduce is necessary. Those with family members with peanut allergy are less likely to introduce without prior hospital observed introduction. Ongoing adherence is unlikely to be achieved in the clinical setting at levels equivocal to the research setting.

**References**

1. Du Toit G, Roberts G, Sayre PH, Bahnson HT, Radulovic S, Santos AF, et al. Randomized trial of peanut consumption in infants at risk for peanut allergy. N Engl J Med. 2015;372: 803–13.
2. Fleischer DM, Sicherer S, Greenhawt M, Campbell D, Chan E, Muraro A, et al. Consensus communication on early peanut introduction and the prevention of peanut allergy in high-risk infants. J Allergy Clin Immunol. 2015; 136: p. 258–61.

### PP055 Patient-perceived impact of food allergy in daily life

#### Fabrícia Carolino, Luís Amaral, Alice Coimbra

##### Serviço de Imunoalergologia, Centro Hospitalar São João, E.P.E., Porto, Portugal

**Correspondence**: Fabrícia Carolino - fabricia.c@sapo.pt

*Clinical and Translational Allergy* 2017, **7(Suppl 1)**:PP055

**Introduction**: Our aim was to assess the perceived impact in daily life of food allergy (FA) in our population.

**Methods**: A medical records review of all patients assessed in our Food Allergy Unit from 2013 to 2015 (n = 187) was performed and those with confirmed FA after diagnostic work-up (n = 51, 27.3%) were selected. These food-allergic patients or their caregivers were then interviewed by telephone; only 21 (41.2%) patients responded and these were included in the final analysis.

**Results**: A total of 21 food-allergic patients (66.7% females, mean ± SD age 28.1 ± 15.7 years; 7 aged under 18 years) were included. The main culprit food groups were crustacean (25.8%), fish (19.4%), fruits (19.4%), and tree nuts (16.1%); 9 patients were allergic to foods from more than one group. Reactions were immediate in 20 (95.2%) cases and 12 (57.1%) presented as anaphylaxis. Two (9.5%) patients confessed to be not fully avoiding foods from the sensitizing group and one had recurrent allergic reactions (oral allergy syndrome); 7 (33.3%) patients admitted not always carrying their emergency medication and 1 had already needed it. Eleven (52.4%) patients stated that they had changed their vacation plans due to FA. Five (23.8%) referred to never or rarely reading food labels and 4 (19.0%) patients had a food allergic reaction following accidental exposure. Five (23.8%) referred to have increased monthly expenses due to FA and another 10 (47.6%) felt somehow impaired by their FA. Interestingly, all patients mentioned to be more confident with the diagnosis and management after an Allergist’s evaluation. Food allergy continues on the rise. The negative practical and psychological impairment associated with FA in both adults and allergic-children caregivers has been recently assessed. A negative impact on daily life was also evident in our patients and this needs to be addressed in order to improve disease management in all aspects. In this group, half of them admitted to changing vacation plans; almost one quarter had increased expenses because of their FA; almost one-fifth had a reaction following accidental exposure and almost half admitted feeling somehow impaired by their FA. All of the patients stated feeling more confident after consultation with a specialist.

**Conclusions**: The scope of an Allergist should also include helping patients cope with FA and reduce its interference in daily life.

### PP060 A pan-allergen patient with eosinophilic esophagitis

#### Josué Alejandro Huertas Guzmán, Montserrat Bosque García, Oscar Asensio, Laura Valdesoiro Navarrete, Helena Larramona, Xavier Domingo Miró

##### Pediatric Pulmonology and Allergy Unit, Hospital de Sabadell, Corporació Parc Taulí, Barcelona, Spain

**Correspondence**: Josué Alejandro Huertas Guzmán - jahg17@yahoo.com

*Clinical and Translational Allergy* 2017, **7(Suppl 1)**:PP060

**Introduction**: The treatment of food allergy is the exclusion diet. Oral immunotherapy (OIT) can be a therapeutic alternative, however described immediate adverse reactions during its implementation. The eosinophilic esophagitis (EoE) is a chronic disease: Esophageal dysfunction and infiltration Eosinophilic ≥ 15 eosinophils (eos/hpf) field in 4 fields. Some recent studies describe it as secondary adverse long-term reaction to the OIT.

**Case report**: 7 year old male patient with an atopic dermatitis history, debuts with urticaria and angioedema after egg tortilla and fish ingestion at 12 months of life. 3 years old: IgE 360UI/ml, Ovomucoid 1.61 kU/L, Egg white 14.5 kU/L, Egg yolk 1.59 kU/L, Ovalbumin 15.61 kU/L, Cod 10.4 kU/L, Tuna 21.4 kU/L, Hake 7.24 kU/L, Sole 6.47 kU/L, Four-spot megrim 7.06 kU/L. Prick: Egg white 6/5, Egg yolk 3/5, Egg 3/5, Ovalbumin 2/5, Ovomucoid 3/5, Cod 7/5, Sole 6/5, Hake 5/5. Oral challenge (PPO) to boiled egg was negative, so diet is indicated with egg cooked 3 times a week and without fish. Later the patient concerns hives after eating Peach, practiced prick: LTP 5/6, Profilina 0/6, Egg white 8/6, Egg yolk 5/6, Egg 2/6, Ovalbumin 2/6, Ovomucoid 4/6, Peach 2/6, Cod 8/6, Sole 7/6, Hake 6/6. IgE 404 IU/ml, Prup3 LTP Peach 4.49 kU/L, Ovomucoid 0.83 kU/L, Egg White 8.95 kU/L, Ovalbumin 8.62 kU/L, Egg Yolk 1.59 kU/L, Four-spot megrim 3.82 kU/L, Cod 5.1 kU/L, Hake 4.4 kU/L, Tuna 5.35 kU/L, Sole 5.52 kU/L. Practiced PPO Omelette was positive, so begins desensitization with egg white, diet without peach and fish. At 7 years old, patient describes food impactation, so endoscopy was performed > 40 (eos/hpf). Treatment: Fluticasone 250mcg BID, Omeprazol, diet without egg, fish and peach.

**Conclusion**: The present case can suggest the implication between EoE and the reintroduction of allergic food so recommendation of stretch following of this patients should be performed after an OIT.

**Consent to publish**: Patient consented to the publication of this abstract.

### PP061 A reporting system for adverse reactions to food in Europe: a community-based Allergic REACTions (AlleREACT) intervention

#### Katarzyna Pyrz^1^, Moira Austin^2^, Yanne Boloh^3^, Philip Couch^4^, Deirdre Galloway^5^, Pilar Hernandez^6^, Jonathan O’B. Hourihane^7^, Fiona Kenna^5^, Marek Kowalski^8^, Barbara Majkowska-Wojciechowska^8^, Clare Mills^4^, Christopher Munro^4^, Lynne Regent^2^, Marina Themisb^4^, Sabine Schnadt^9^, Aida Semic-Jusufagic^4^, Angela Simpson^4^, Audrey Dunn Galvin^1^

##### ^1^School of Applied Psychology, University College Cork, Cork, Ireland; ^2^Anaphylaxis Campaign (ACUK), Farnborough, United Kingdom; ^3^Association Française pour la Prévention des Allergies (AFPRAL), Paris, France; ^4^University of Manchester, University Hospital of South Manchester, Manchester, United Kingdom; ^5^Anaphylaxis Ireland (AI), Ireland; ^6^Asociación Española de Personas con Alergia a Alimentos y Látex (AEPNAA), Madrid, Spain; ^7^Department of Pediatrics and Child’s Health, University College Cork, Cork, Ireland; ^8^Medical Univeristy of Łódź (MUL), Lodz, Poland; ^9^Deutscher Allergie- und Asthmabund e.V. (DAAB), Mönchengladbach, Germany

**Correspondence**: Katarzyna Pyrz - kpyrz@ucc.ie

*Clinical and Translational Allergy* 2017, **7(Suppl 1)**:PP061

**Introduction**: Mild to severe adverse reactions to food in the community are under-researched, and bio-psychosocial risk factors are poorly understood. An online system has been developed to allow for real-time reporting of incidents for food allergic patients in seven European countries under auspices of the *iFAAM (Integrated Approaches to Food Allergen and Allergy Risk Management)* project.

Our aim was to develop and validate a real-time, multilingual, online-based reporting system to capture real-world circumstances and determinants of food-allergic incidents across Europe.

**Methods**: The *Allergic REACTions (AlleREACT)* is an international community-based intervention using a prospective multi-site design. Adults, adolescents and parents of children with food allergies in the UK, Ireland, Germany, Spain, France, Belgium and Poland will report food allergic incidents using a validated online reporting tool between April-December 2016.

**Results**: Following experts’ consultations and Patient Organisations’ (POs) workshops, a short version of a previously validated reporting tool ‘Allergic Reactions in the Community’ (AlleRiC) was adapted for international use. The new measure has already been tested online and piloted in the English language by adults and parents of children with food allergies (N = 37). Translation and back-translation have been completed in German, Spanish, French and Polish and the system has now been launched. The resulting tool “*AlleREACT’*” is a comprehensive (42 items), user-friendly and takes only 10 min to complete.

**Conclusions**: AlleREACT builds on e-Health and community-based research frameworks. It will provide novel findings in areas not previously researched in depth, and promote understanding of food allergic incidence with implications for risk assessment and risk management across Europe. It is intended that the tool will be used by patient groups and individuals within the community and managed by POs from participating countries.

### PP062 Outcome of oral immunotherapy in cow’s milk allergy in Finland after a follow-up of 7–11 years

#### Tiina Kauppila, Anna Kaarina Kukkonen, Mikael Kuitunen, Anna Pelkonen, Mika Mäkelä

##### University of Helsinki, Helsinki University Hospital, Skin and Allergy Hospital, Helsinki, Finland

**Correspondence**: Tiina Kauppila - tiina.k.kauppila@helsinki.fi

*Clinical and Translational Allergy* 2017, **7(Suppl 1)**:PP062

**Introduction**: Our aim was to evaluate the outcome of oral immunotherapy (OIT) in cow’s milk allergy 7–11 years after starting the OIT.

**Methods**: A real-life follow-up study on OIT to milk was started in 2005 by a protocol presented in detail earlier [1]. By March 2009, 67 children had participated in OIT (Table 3). Information about current milk consumption and side effects to milk were collected from 63 patients between January to April 2016 with a questionnaire, phone calls, or from medical records (Table 4).

**Table 3:** See text for description

Baseline characteristic before milk OIT (n = 67)	
Male/Female	37 (55%)/30 (45%)
Age (y), median (InterQuartile Range, IQR)	9.9 (5.8–17.4)
Median age when cow’s milk allergy was diagnosed (months)	7
Asthma, [n (%)]	51 (76)
Allergic rhinitis, [n (%)]	41 (61)
Atopic dermatitis, [n (%)]	30 (45)
Baseline milk IgE (kU_A_/L), median (IQR)	17.2 (0.2–1390.0)
Baseline milk skin prick test score (mm), median (IQR)	10.0 (4.0–20.0)

**Table 4:** See text for description

Long-term follow-up data collection (n = 67)	
Median follow-up time (years)	8.7 (7.0–11.1)
Questionnaire	35 (52.2%)
Phone	15 (22.4%)
Medical records (recent 6 months)	13 (19.4%)
Lost in the follow up	4 (6%)

**Fig. 6:**
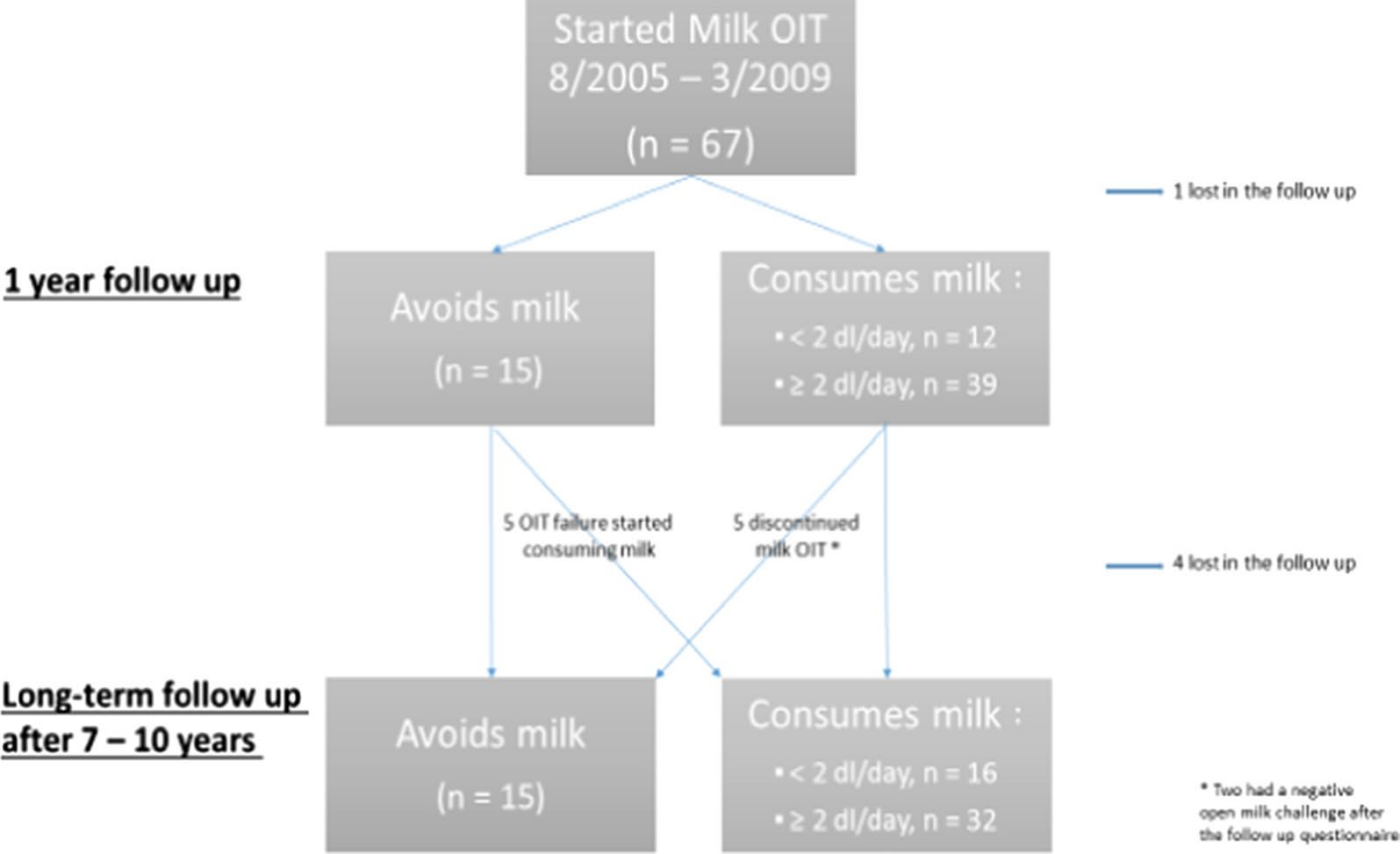
See text for description

**Results**: At the 1-year follow up visit, 51 (76%) patients consumed milk daily (Fig. 6) and 23 (45%) of them reported side effects to the milk. The side effects were mostly mild and they required no medication or only antihistamine. One patient had an anaphylactic reaction during the OIT build-up phase. After the reaction, the patient continued OIT and is now consuming 5 dl milk a day without reporting any side effects. At the 7–11 years follow-up, 48 (72%) patients consumed milk daily (Fig. 6) and 18 (38%) of them reported side effects to milk during the last 12 months. Side effects were mostly mild requiring no medication, or only antihistamine, but two patients reported using adrenalin to allergic reaction after consuming milk. Information about side effects was not available for 6 (13%) patients. Five patients, who were previously consuming milk, had discontinued its consumption because of the symptoms. However, two of them recently had a negative oral food challenge (OFC) to milk. Five patients who previously failed in milk OIT, and were on a milk-avoidance diet, consumed milk at the long-term follow up. They had started using small amounts of cheese daily by escalating doses or had a negative OFC to milk or had started milk OIT again. Our results suggest that performing a long-term follow-up after milk OIT is important, irrespective of the outcome of the OIT.

**Conclusions**: After milk OIT, the milk consumption can change over time and allergic reactions may appear even after 7 years or more of daily milk consumption.

**Reference**

1. Kuitunen M, Englund H, Remes S, Moverare R, Pelkonen A, Borres M.P, Mäkelä M. High IgE levels to α—lactoglobulin and casein predict less successful cow’s milk oral immunotherapy. Allergy 2015;70:955–62.

### PP063 A comparative audit of milk and fish induced Food Protein Ιnduced Εnterocolitis Syndrome (FPIES) management

#### Nikolaos A. Kitsioulis^1^, Nikolaos Douladiris^1^, Sofia Kostoudi^1^, Ioanna Manolaraki^1^, Dimitris Mitsias^1^, Emmanouil Manousakis^1^, Nikolaos G. Papadopoulos^1,2^, Paraskevi Xepapadaki^1^

##### ^1^Allergy Department, 2nd Pediatric Clinic, University of Athens School of Medicine, Athens, Greece; ^2^University of Manchester, Manchester, United Kingdom

**Correspondence**: Nikolaos A. Kitsioulis - drnok21@gmail.com

*Clinical and Translational Allergy* 2017, **7(Suppl 1)**:PP063

**Introduction**: FPIES is a non-IgE-mediated food allergy, often unrecognized, attributed to a variety of foods, with cow’s milk (CM) and fish to be considered two of the most common culprit foods.

Our aim was to evaluate diagnostic and management procedures in children with FPIES attributed to CM and fish, in a reference Allergy Unit of a tertiary Hospital in Greece.

**Methods**: This is a retrospective study on records between 10/2010 and 10/2015. Diagnosis was based on self-reported clinical history, however oral food challenges (OFCs) were performed in case of unclear history. Age at diagnosis and resolution of the syndrome were recorded. For the assessment of potential tolerance, OFCs were performed, in a wide range of time, in dependence of patients’ accidental exposures, and not according to conservative approach (every 18–24 months) as recommended.

**Results**: 47 patients (23 males) were diagnosed with FPIES, 27 to CM and 20 to fish (median age: 5 and 18 months, IQR 2–7 and 12–22.5 respectively). We performed 45 OFCs in total (18 to fish), 41 to evaluate potential tolerance (17 to fish). As regards OFCs performed to assess potential tolerance, 11 out of 17 (64.7%) OFCs to fish were positive, while corresponding ratio to CM was 4/24 (16.7%). Median time between diagnosis and OFC was 24 months in total (IQR 16.5–36.7), while regarding patients with negative OFC outcome (tolerance), the corresponding time was 22 months (IQR 15.9–30.4). In children still with active disease at re-evaluation, it was noted that in respect to fish even though they have been challenged in older age (fish 73.3 ± 25.9 months vs CM 22.6 ± 6.8 months, p = 0.002), they had in their majority positive OFCs. A significant association regarding the interval between diagnosis and OFC with OFC outcome (fish 51.1 ± 25.5 months vs CM 14.7 ± 2.2 months, p = 0.015) was also noted. Out of 41 children with FPIES diagnosis at presentation, 63.4% (n = 26) were tolerant by means of OFCs (6 to fish). In respect to the interval from diagnosis to tolerance, correlation with food type was noted (fish median 27.7 months, IQR 22–73.5 vs CM median 20 months, IQR 15.1–26.9, p = 0.044).

**Conclusions**: There is heterogeneity in the resolution of FPIES depending on the implicated food. FPIES caused by fish has a delayed remission. Ιt is more cost-effective for both the patients and the medical staff to avoid early OFCs, compared to time of diagnosis, as regards fish or more delayed concerning CM.

### PP064 Coping with food allergy by children and adolescents – A systematic review

#### Rebecca Knibb, Jennifer Hammond, Richard Cooke

##### Aston University, Birmingham, United Kingdom

**Correspondence**: Rebecca Knibb - r.knibb@aston.ac.uk

*Clinical and Translational Allergy* 2017, **7(Suppl 1)**:PP064

**Introduction**: Children and adolescents with food allergy are the group most at risk of serious and fatal reactions from accidental ingestion of an allergen. Ways in which they cope with their food allergy could explain this increased risk. A systematic review was conducted of published papers looking at coping strategies used by children and adolescents with food allergy to explore current knowledge and identify gaps for future research.

**Methods**: Electronic searches were conducted using the following databases: MEDLINE, PsycINFO, SCOPUS, Science Direct, Web of Science. Papers including data from participants aged 8–16 years old with a food allergy or hypersensitivity were retrieved and analysed. Thematic analysis was used to synthesise the findings.

**Results**: Twelve studies were selected from 4672 papers after a review of abstracts and full texts. These papers underwent data extraction, quality appraisal and thematic analysis. Six key themes were identified: (1) Coping with risk (2) Using auto-injectors (3) Education, knowledge and understanding (4) Social support (5) Taking responsibility and (6) Coping with emotions. Adaptive coping strategies were identified such as problem-solving and planning; maladaptive coping strategies such as mental or behavioural disengagement were also apparent. Education and knowledge regarding allergies and its treatment varied and influenced how well patients coped, as did attitudes towards the allergy. Social and peer support was important for coping but can be limited for children and adolescents with food allergy. Many felt that their needs were not fully understood, even at school, and some felt pressured by peers or social situations to ignore the risks. Coping with food allergy by children and adolescents is a complex multifaceted process and the type of strategy used is dependent on the individual’s perception of risk, the situation or environment they are in, the influence and attitude of others, and the individual’s age and gender. Participants in the review were mainly teenagers; therefore further research on how younger children cope with food allergy is needed.

**Conclusions**: Ways of teaching adaptive coping strategies from point of diagnosis need to be investigated. The impact of social influences should be considered when supporting this population particularly as they get older, with age-appropriate strategies that facilitate confidence in being able to cope with and manage their food allergy.

### PP065 INTO-study – Randomized controlled study on the INduction of TOlerance in babies

#### Jaakko Yrjänä^1^, Anna-Maija Hanni^2^, Päivi Vähäsarja^2^, Oona Mustonen^1^, Teija Dunder^1^, Petri Kulmala^1^

##### ^1^PEDEGO Research Unit and Medical Research Center Oulu, University of Oulu and Oulu University Hospital, Oulu, Finland; ^2^Pediatric Unit, Kontinkangas Healthcare and Wellness Center, Oulu, Finland

**Correspondence**: Petri Kulmala - petri.kulmala@oulu.fi

*Clinical and Translational Allergy* 2017, **7(Suppl 1)**:PP065

**Introduction**: This general population based RCT aims at answering two major hypotheses: First, systematic early introduction of solid foods decreases the incidence of food allergy and dietary restrictions by the age of one year. Second, stimulation with the symptom-eliciting food rather than avoidance will induce tolerance in babies with non-severe allergic symptoms.

**Methods**: All new born babies living in the city of Oulu are recruited to the study (n = 1380) at their first health nurse visit (at or before 1 month of age) in the local primary care child health clinics. Families in the intervention group will get an instruction booklet including information on early systematic introduction of solid foods starting at the age of 4 months, with foodstuff from all major groups in diet by the age of 6 months (vegetables and fruits, wheat and other grains, meat, fish, egg, dairy products). Furthermore, the booklet includes information and instructions on food related symptoms and atopic eczema. Babies with mild symptoms are encouraged to continue the symptom-eliciting food. All families fill out monthly internet-based questionnaires on food diary, symptoms, diagnoses and health care visits until the age of 1 year. Dietary restrictions and food allergy diagnoses are verified at the age of one year. The primary outcome measures will be the incidence of diet restrictions, parent-reported and doctor-verified food allergies and atopic eczema by the age of 1 year. The secondary outcomes will be the need for health services and the family experienced distress during the first year of life.

**Results**: The study has started in March 2014. Currently, 1317 babies have been recruited and 645 children have completed the entire study by the end of April 2016. According to the preliminary data from questionnaires at the age of 6 months, 70.6% of 425 children in the intervention group and 74.1% of 444 children in the control group were still on breast-feeding (*p* = *0.256*). Furthermore, 45.9% of children in the intervention group and 23.3% in the control group (*p* < *0.001*) had 2 or more potentially allergenic food (wheat, egg, fish or fermented milk products) in their diet before the age of 6 months.

**Conclusions**: The study protocol seems to be feasible at a population level and does not affect the rate of breastfeeding during the first 6 months of babies. INTO-study will provide invaluable data on early-feeding strategy in primary and secondary prevention of food allergy.

### PP066 Adherence and safety in home doses in oral specific tolerance induction

#### Eva Lasa^1^, Carmen D’Amelio^2^, Sara Martínez^1^, Alejandro Joral^1^, Gabriel Gastaminza^2^, Maria Jose Goikoetxea^2^

##### ^1^Hospital Universitario Donostia, San Sebastián, Spain; ^2^Clinica Universidad de Navarra, Pamplona, Spain

**Correspondence**: Eva Lasa - evamarialasa@gmail.com

*Clinical and Translational Allergy* 2017, **7(Suppl 1)**:PP066

**Introduction**: Egg and milk allergy is the main food allergy in the paediatric population in our country. In recent years, as an alternative to egg and milk avoidance a novel and promising treatment has appeared consisting in induction of oral tolerance. Allergenic food doses are increased progresively from a minimun amount of egg/milk. Doses are increased in the hospital and the same dosis are maintained at home. The procedure is not free of risk even when doses are taken at home.

We aim to evaluate the adherence to desensitized food doses and patient safety at home.

**Methods**: One-hundred and twenty patients treated by specific oral tolerance induction (SOTI) to milk or egg from two hospitals were invited to fill an anonymous survey via web. Similar recommendations were given to the patients under SOTI in these two hospitals and full-time access to anallergist was available by e-mail or telephone. Survey asked about adherence to treatment, cofactors avoidance and confidence about treating reactions at home.

**Results**: 109 patients answered the survey. 70% of the patients never forgot to take the dose. Whereas 82% never forgot to take premedication (antihistamines), 40% forgot to take asthma medication. Most of the patients always avoided cofactors (89% avoided fasting, 90% NSAIDs, 78% exercise) and 88% of the patients were correctly monitored after taking the doses always. However, regarding reactions at home, at least in one reaction, 53% of the patients did not know if the reaction suffered should be treated and 26% did not know the type of medication required for that reaction. Most of the patients showed good adherence to the treatment and correct cofactors avoidance however, patients do not feel confident when a reaction occurs.

**Conclusions**: Patients need to be teached in managing reactions before starting a food SOTI.

### PP067 Dietary management of non-IgE mediated cow’s milk allergic infants with a synbiotics-supplemented amino acid-based formula: effects on faecal calprotectin, eosinophilic cationic protein and α1-antitrypsin concentrations

#### David C. A. Candy^1^, Marleen T. J. Van Ampting^2^, Johan Garssen^2,3^, Manon M. Oude Nijhuis^2^, Assad M. Butt^1^, Diego G. Peroni^4^, Adam T. Fox^5^, Lucien F. Harthoorn^2^, Jan Knol^2,6^, Louise J. Michaelis^7^

##### ^1^Royal Alexandra Children’s Hospital, Brighton, United Kingdom; ^2^Nutricia Research, Nutricia Advanced Medical Nutrition, Utrecht, the Netherlands; ^3^Utrecht Institute for Pharmaceutical Sciences, Faculty of Science, Utrecht University, Utrecht, the Netherlands; ^4^University Hospital Verona, Verona, Italy; ^5^Guy’s & St Thomas’ Hospitals NHS Foundation Trust, London, United Kingdom; ^6^Laboratory of Microbiology, Wageningen University, Wageningen, the Netherlands; ^7^Great North Children’s Hospital, Royal Victoria Infirmary, Newcastle upon Tyne, United Kingdom

**Correspondence**: Marleen T.J. Van Ampting - marleen.vanampting@nutricia.com

*Clinical and Translational Allergy* 2017, **7(Suppl 1)**:PP067

**Introduction**: This study investigated the effects of a new amino acid-based formula (AAF) with pre-and probiotics (synbiotics) in infants with suspected non-IgE mediated CMA.

**Methods**: In a prospective, randomised, double-blind controlled study (registered as NTR3979), infants with suspected non-IgE mediated CMA were enrolled to receive either an AAF (control n = 36) or an AAF with synbiotics (oligofructose, long-chain inulin, Bifidobacterium breve M-16 V) (test n = 35) for 8 weeks. Faecal samples were collected at baseline and weeks 8, 12 and 26 after initiation of dietary management. Faecal samples from non-randomized healthy breastfed infants, which were age-matched with CMA infants at wk 8 of intervention, were collected as a reference group. Primary outcomes were bifidobacteria and the Eubacterium rectale/Clostridium coccoides group (ER/CC) as percentage of total faecal bacteria determined by fluorescent in situ hybridization. To monitor intestinal inflammation and immune status secondary and exploratory outcomes included faecal Calprotectin (FC) and Eosinophilic Cationic Protein (ECP) and faecal α1-antitrypsin (A1A).

**Results**: Average age (±SD) of CMA infants (n = 71) was 6.00 ± 2.98 months at inclusion of the study and 7.84 ± 3.25 months in the reference group (n = 51). Of the CMA subjects, 90% presented predominantly GI symptoms and 10% dermatological symptoms; stratification was based on these manifestations. Sixty CMA infants completed the 8 weeks intervention (control n = 32; test n = 28). Using the intention-to-treat data set and ANCOVA technique, levels of bifidobacteria at 8 weeks were significantly higher in the test (35.6%) vs. control group (14.7%) (p < 0.001) and ER/CC was significantly lower in the test (12.1%) vs. control group (26.6%) (p < 0.001). Determined bacterial proportions in the test group were close to levels observed in the healthy reference group. Median FC and A1A were lower in week 8 compared to week 0, but were not significantly different between groups at week 8 (p = 0.815 and p = 0.717, respectively). Additional outcomes, including healthy reference data, will be presented.

**Conclusions**: This study shows that an AAF with specific synbiotics significantly affects the gut microbiota composition of non-IgE CMA infants. Determined bacterial proportions in the test group were close to levels observed in the healthy reference group. Median levels of FC and A1A decreased in both study groups at week 8 of intervention.

### PP069 Travelling with food allergy: are the commercial airlines prepared?

#### Ines Padua^1^, Andre Moreira^2^, Patricia Padrao^1,3^, Pedro Moreira^1,4^, Renata Barros^1^

##### ^1^Faculty of Nutrition and Food Sciences, University of Porto, Porto, Portugal; ^2^Faculty of Medicine, University of Porto, Porto, Portugal; ^3^Institute of Public Health, University of Porto, Porto, Portugal; ^4^Research Centre in Physical Activity, Health and Leisure, University of Porto, Portugal

**Correspondence**: Ines Padua - inespadua@gmail.com

*Clinical and Translational Allergy* 2017, **7(Suppl 1)**:PP069

**Introduction**: Food allergy (FA) has a significant impact on the patient’s quality of life, namely on travelling. We aimed to assess the ability of the commercial airlines to support passengers with FA.

**Methods**: A worldwide commercial airlines on-line survey, including 841 companies, from 216 countries, addressing their preparation to support passengers with FA was performed. A total of 721 airlines were reached by 2 reminders (3 refuse to participate and contact failed with 117). Additionally, the Top 100 Airlines, according to the World Airlines Awards 2015, were contacted to reply to a simulated reservation for a passenger with FA addressing staff training, the availability of special meals for FA on board, and the possibility to travel with their own food and medication. The information available on the airline’s website was also analysed. Statistical analysis included descriptive statistics.

**Results**: 3 out of 721 companies completed the survey and reported to have trained staff to deal with emergencies and no restrictions for travelling costumers with FA concerning to food or medication carrying. From the remaining, 713 failed to reply and 5 reported not being able to provide requested information. Considering the simulated reservation, 22 out 100 replied. Six reporting to have the information on their website, 8 having special food allergy menus, 2 staff training for emergencies, 9 allowed passengers to carry their epinephrine injector; 11 allowed to bring food and 8 highlighted that cannot guarantee allergen-free flights, regarding the risk of cross-contact. Concerning the Top 100 website information, only 4% mention that the flight crew are trained, 67% have special FA menus, 40% recommend the carriage of medication and 26% refer it is possible to carry food on board.

**Conclusions**: Most of the commercial airlines are not prepared or even warned about the impact of an in-flight allergic reaction. Our results aware for a priority on airline’s education and training in FA order to increase the family’s confidence to travel and afford the life safety on board.

### PP073 Management of life threatening reactions to food allergy in school aged children of Sharjah UAE

#### Hanan Sharif, Manzoor Ahmed, Nehad Gomaa

##### Pediatric Department, University Hospital Sharjah, Sharjah, United Arab Emirates

**Correspondence**: Manzoor Ahmed - Manzoor.Ahmed@uhs.ae

*Clinical and Translational Allergy* 2017, **7(Suppl 1)**:PP073

**Introduction**: Food allergy is a growing problem in the school aged children It is a common cause of anaphylaxis [1], with an estimation of 1 in 25 children at school affected [2]. Surveys indicate that 16 to 18% of children with food allergy experience a reaction in school [3,4]. In UAE, 8% of children suffer from food allergy [5].

The objectives of this study was to look for School readiness to treat anaphylaxis to food allergy, how are they managed, and to identify any scope for further improvement.

**Method**: Data was collected retrospectively from schools in Sharjah, A validated questioner (on epinephrine administration) was distributed in English and Arabic to all Schools which was filled by the School clinic nurse /doctor for those children’s who encountered life threatening reactions to food and required epinephrine administration during 2013–2014. 33 out of 80 schools in Sharjah completed the epinephrine administration form. An official approval from Ministry of Education, Sharjah was obtained for this study.

**Results**: Eighty schools in emirate of Sharjah have been invited to participate in the survey 33 schools responded by filling up the questioner. The survey showed the following results:

- Number of the schools that have Epi pen available: 3 schools (9%).
- Number of the schools that have emergency response activated team: 3 schools (9%).
- Number of food allergy cases reported 14 cases; 2 cases were managed with Epi pen adminstration, and 12 cases were managed with antihistamines and transferred immediately to hospital as epi pens were not available.

Time required for treatment of the two cases treated with Epi pen was within 3to 5 min, one given by the student and the other by the school doctor.

**Conclusions**: Our survey showed that Anaphylactic reaction in schools are not uncommon. Management of such cases need clear policies to recognize and treat allergic reactions and anaphylaxis. Epi pen availability, food allergy action plans and school nurse training and education are vital to ensure the safety of such children.

**References**

1. Decker WW, Campbell RL, Manivannan V, etal. The etiology and incidence of anaphylaxis in Rochester, Minnesota: a report from the Rochester Epidemiology Project. J Allergy Clin Immunol. 2008;122(6):1161–5.
2. Branum AM, Lukacs SL. Food allergy among children in the United States. Pediatrics. 2009;124(6):1549–55.
3. Nowak-Wegrzyn A, Conover-Walker MK, Wood RA. Food-allergic reactions in schools and preschools. Arch PediatrAdolesc Med. 2001;155(7):790–5.
4. Sicherer SH, Furlong TJ, DeSimone J, Sampson HA. The US Peanut and Tree Nut Allergy Registry: characteristics of reactions in schools and day care. J Pediatr. 2001;138(4):560–5.
5. Al-Hammadi S, Al-Maskari F, Bernsen R Prevalence of food allergy among children in Al-Ain city, United Arab Emirates. Int Arch Allergy Immunol. 2010;151(4):336–42.

### PP075 Supporting anaphylaxis patients through a mobile application

#### Harmieke van Os - Medendorp^1^, Joris Mens^2^, Koen Smit^2^, Frans Timmermans^3^, André Knulst^1^

##### ^1^Department of Dermatology/Allergology, UMC Utrecht, Utrecht, the Netherlands; ^2^Utrecht University Graduate School of Natural Sciences, Utrecht, the Netherlands; ^3^Dutch Anaphylaxis Network, Dordrecht, the Netherlands

**Correspondence**: Harmieke van Os - Medendorp - h.vanosmedendorp@umcutrecht.nl

*Clinical and Translational Allergy* 2017, **7(Suppl 1)**:PP075

**Introduction**: This study was performed to identify the critical success factors (CSFs) of an anaphylaxis app to support patients in an emergency situation.

**Methods**: CSFs were identified through a literature study, by analyzing existing anaphylaxis apps and by surveying a sample of patients who visited the website of the Dutch Anaphylaxis Network. An expert interview was used to further reflect upon the findings.

**Results**: Five existing anaphylaxis apps were found, with different features, most were for the UK or US market, available for free or at low costs. The literature presents the following CSFs that influence the adoption of mobile technology for allergic patients: 1. being context-aware through scanning of physical items; 2. automating the retrieval of product-related allergy information; 3. helping to improve the patient’s understanding of their condition in general; 4. ensuring medical validation of given advice; 5. being tailored to patient’s medications and 6. providing advice in a patient-sensitive manner, based on age and level of experience. The survey yielded 276 responses, 80% was female, mean age of 40.96 (sd = 11.2) years and an average of 9.81 (sd = 7.5) years of experience with anaphylaxis. The desirability of an app to support anaphylaxis patients was rated with an average of 8.34 on a scale of 1 to 10 (sd = 1.8), while the probability of the respondents using such an app was rated with an 8.75 (s = 1.9). Most important features of an app according to the respondents was Clarity of information (6.68); Medical validation (6.62) and Emergency procedure information (6.48). Less important features were Interactivity of the app (4.50); Allergy knowledge quiz (3.33) and use of games (2.66). The expert mentioned that the app should be available at no cost to boost its use.

**Conclusions**: An app that includes medically validated and clear information on allergy symptoms and treatment procedures has great potential to fill a gap in the support of anaphylaxis patients. Relevance for daily practice. An app in Dutch language will be developed to support patients or caregivers in emergency situations. The app will be patient-tailored, so that a patient can import his own emergency medication and personal information. The main feature is a step-wise approach to cope with allergic reactions. Besides general information about the most important causes for anaphylaxis: food allergy or insect sting allergy will be given. The app will be built in cooperation with Allergy patient’s organizations, the Dutch dermatologists’ association and the University Medical Centre Utrecht, the Netherlands.

### PP077 Negative impact of multiple elimination diets on nutritional status in food allergic children

#### Tina Vesel^1^, Tomaž Poredoš^1^, Anja Koren Jeverica^2^, Marjeta Sedmak^1^, Evgen Benedik^1^, Tadej Avčin^1^

##### ^1^University Children’s Hospital, University Medical Center, Ljubljana, Slovenia; ^2^University Clinic of Respiratory and Allergic Diseases, Ljubljana, Slovenia

**Correspondence**: Tina Vesel - jana.vesel1@telemach.net

*Clinical and Translational Allergy* 2017, **7(Suppl 1)**:PP077

**Introduction**: Nutritional deficiencies have been reported in food-allergic children. The aim of our study was to assess the actual food intakes and nutritional status of children with suspected or proven multiple food allergies when elimination diet was not supervised by continuing dietitian follow-up.

**Methods**: 22 children (8 girls, 16 boys; mean age 5.0 ± 4.1 years) with proven (19 children) or suspected only (3 children) multiple food allergies were studied retrospectively. Twenty children presented with atopic dermatitis, eight with anaphylaxis, two with hives and four with gastrointestinal symptoms. Two children had additional conditions that influenced nutritional status (one cromosomopathia, one histamine intolerance). Nutritional intakes assessment was passed on exact questioning on actual food avoidance. Children’s weight, height, laboratory data for nutritional parameters were assessed.

**Results**: In 18 (82%) children additional questioning revealed their diet was extended from advised by physician. Fourteen children (64%) did not eat milk, egg and wheat and in addition numerous variable foods were also avoided in their diets- in nine of animal origin and in twelve of plant origin (most often peanuts and tree nuts). Two children did not consume egg and milk and excluded also some plant and/or animal foods, two children avoided numerus plant foods, one child did not eat cow milk and some plant foods and one child had no special food restrictions. In described group of 22 children the means for anthropometric measures were below the average for age (41.P for height and 35.P for weight). Three children were <−3% for relative height and two children were <−3% for relative weight. Lower serum levels of levels of albumin/iron/zinc/selenium/vitamin B12 were found in 5/8/11/10/1 children, respectively. Three children had osteoporosis and one had osteopenia. Eleven children (50%) had multiple nutritional deficits. Combined diet without cow milk, egg and wheat (14 children) was always associated with nutritional deficits.

**Conclusions**: Multiple food elimination diet (e.g. without cow milk, egg and wheat) has negative impact on nutritional status of food-allergic children if not supervised continually by experienced physician and dietitian.

### PP078 Improved diagnostic procedure by ISAC multiplex assay in food and latex allergic children

#### Tina Vesel^1^, Anja Koren Jeverica^1^, Mira Šilar^2^, Meta Accetto^1^, Mirjana Zupančič^1^, Tadej Avčin^1^, Peter Korošec^2^

##### ^1^University Children’s Hospital, University Medical Center, Ljubljana, Slovenia; ^2^University Clinic of Respiratory and Allergic Diseases, Ljubljana, Slovenia

**Correspondence**: Tina Vesel - jana.vesel1@telemach.net

*Clinical and Translational Allergy* 2017, **7(Suppl 1)**:PP078

**Introduction**: One of the most important implications of a multiplex assay such as ISAC assay should be its ability to distinguish between allergy and asymptomatic sensitisation due to cross reactivity which could ex. influence the decision for performing a food/latex challenge in children with multiple allergies.

**Methods**: Test results of food and latex recombinants in 53 ISACs were retrospectively analysed and compared in allergic versus tolerant children. Allergy was confirmed by typical clinical history and determination of specific IgE/skin prick test, by provocation test or by very high values of specific IgE/skin prick tests. When analysing potential cow’s milk/egg/soy/wheat/peanut/hazelnut/fruit/latex allergy 1/2 /8 /1 /14 /15 /3 /3 children were excluded because of unknown clinical allergic status (e.g. waiting/refusing provocation test).

**Results**: 12 egg allergic children had significantly higher specific IgE to nGal d 1, nGal d 2 and nGal d 3 than 39 egg tolerant children (p < 0.0001). 12 cow’s milk allergic children had significantly higher specific IgE to nBos d 4, nBos d 5 and nBos d 8 than 40 cow milk tolerant children (p < 0.0001). 16 peanut allergic children had higher specific IgE to rArah 1, n/rArah 2, n/r Arah 3, nArah 6, rArah 8 and rArah 9 than 23 peanut tolerant children (p from <0.0001 to < 0.05). 14 hazelnut allergic children had higher specific IgE to rCor a 8 and rCor a 9 than 25 hazelnut tolerant children (p = 0.006 and p = 0.0009, respectively) and significantly lower specific IgE rCor a 1 than hazelnut tolerant children (p < 0.0001). Three soy allergic children had significantly higher specific IgE to nGly m 5 and nGly m 6 than 42 soy tolerant children (p < 0.001 and p = 0.003, respectively). Two wheat allergic children differed from 50 wheat tolerant in rTri a 19, rTri a 14, tTri a aA_TI (p from < 0.001 to< 0.05). 6 children with anaphylaxis to different fruit had significant higher n/r Pru p 3 than 25 fruit tolerant children (p = 0.03). 18 children with OAS associated with fruit had significantly higher specific IgE to rPru p 1, rMal d 1 and rApi g 1 than 25 fruit tolerant children (p < 0.0001). Four children allergic to latex had significant higher specific IgE antibodies to rHev b 6.01 than 46 latex tolerant children (p = 0.005).

**Conclusions**: ISAC differentiated allergic from tolerant children when analysing recombinants of cow’s milk egg, peanut, hazelnut, soy, wheat, fruit or latex.

### PP082 Esophageal eosinophilic infiltration during desensitisation treatment: a growing concern

#### Glauce Yonamine, Gustavo Soldateli, Bruna Aquilante, Antonio Carlos Pastorino, Cleonir Lui de Moraes Beck, Andrea Keiko Gushken, Mayra de Barros Dorna, Cristiane Nunes dos Santos, Ana Paula Moschione Castro

##### Child Institute Clinical Hospital, University of São Paulo, São Paulo, Brazil

**Correspondence**: Glauce Yonamine - glaucehy@uol.com.br

*Clinical and Translational Allergy* 2017, **7(Suppl 1)**:PP082

**Introduction**: To describe the frequency and evolution of esophageal eosinophilic infiltration in patients with cow’s milk protein allergy (CMPA) submitted to oral desensitization.

**Methods**: A descriptive study involving pediatric patients with CMPA submitted to desensitization protocol from 2012 to 2016. This protocol included daily intakes of increasing diluted amounts of cow milk until 120–200 ml of non-diluted milk. The mean initial dilution was 10^−7^ and the average time of progression from diluted milk to non-diluted milk was 3 months. Patients reported symptoms daily, and when persistent gastrointestinal symptoms occurred, endoscopy was performed. Abnormal findings (endoscopy and histology) suggestive of eosinophilic infiltration (eosinophils ≥15HPF) demanded appropriated treatment with proton-pump inhibitor (PPI) for 8 weeks. Swallowed topical corticosteroids (STC) was initiated if inadequate response.

**Results**: 19 patients were enrolled (11 F: 8 M), mean age of 10,25 years at baseline. All patients had previous history of anaphylaxis and positive specific IgE to cow milk and casein. Nine patients (9/15) presented specific IgE levels to cow milk and casein higher than 100 kU/L. Five patients developed gastrointestinal symptoms during treatment, all of them receiving non-diluted milk being abdominal pain the most frequent complaint, followed by vomiting and dysphagia. All patients had abnormal macroscopic findings, and increased eosinophils (mean = 20 HPF Eo). Four patients (P1, P2, P3, P4) received PPI as initial therapy, and one patient lost follow up (P5). Endoscopy was repeated and eosinophils were normal in two patients and clinical symptoms disappeared (P1, P2) but symptoms returned in one (P1). Another patient had milk excluded (P3) and P4 is waiting second endoscopy. STC was necessary in two patients with distinct evolution (P1, P3). One patient kept milk ingestion and STC (P1) and the other persisted with endoscopic abnormalities despite milk exclusion and STC treatment (P3).

**Conclusions**: Esophageal eosinophilic infiltration is a described complication in patients submitted to oral desensitization, but it seems more prevalent in patients with severe allergy and presents different outcomes. It is necessary continuous follow up of these patients for long periods to understand the disease and provide the best management.

## POSTER SESSION 3: Anaphylaxis • Experimental and socioeconomic aspects • Molecular mechanisms

### PP084 Vitamin D levels in patients with anaphylaxis with or without asthma compared to the general Saudi population

#### Abdulhadi Al-Qahtani, Rand Arnaout, Agha Rehan Khaliq, Rashid Amin, Farrukh Sheikh

##### King Faisal Specialist Hospital and Research Centre, Riyadh, Saudi Arabia

**Correspondence**: Abdulhadi Al-Qahtani - Dr.abdulhadi@outlook.com

*Clinical and Translational Allergy* 2017, **7(Suppl 1)**:PP084

**Introduction**: Vitamin D is known for its role in the bone metabolism but it also has immune-modulatory properties. Most of the evidence points towards a causal relationship between low vitamin D levels and the development of asthma and allergies but the results are not conclusive. We studied the vitamin D levels of patients who presented to our hospital with anaphylaxis with or without asthma and compared it to previously published vitamin D levels in Saudi population.

**Methods**: All patients given new Adrenaline auto injector prescriptions for anaphylaxis between the periods of 1/1/2010 and 31/12/2011 were included in this study. Their medical records were also screened for diagnosis of asthma.

**Results**: A total of 238 patients were identified. Data about Vitamin D level was available for 121 of those patients. 84 out of these 121 patients were also being treated for asthma. There was no evidence of any difference in vitamin D levels between those with or without asthma presenting with anaphylaxis. Vitamin D levels compared in patients with anaphylaxis to the general population revealed that while there was no significant difference in terms of vitamin D deficiency, patients who presented with anaphylaxis had a higher chance of either having a normal or higher than normal vitamin D level.

**Conclusions**: When compared with the general population, patients with anaphylaxis have more chance to have normal vitamin D level. Vitamin D deficiency was not found to be a significant risk factor for anaphylaxis in our patients compared with the general Saudi population.

### PP085 Quality of triage of children with anaphylaxis at the Pediatric Emergency Department

#### Esozia Arroabarren, Jorge Alvarez, Marta Anda, Miriam Palacios, Montserrat De Prada, Carmen Ponce

##### Complejo Hospitalario De Navarra, Pamplona, Spain

**Correspondence**: Esozia Arroabarren - esoziaa@yahoo.es

*Clinical and Translational Allergy* 2017, **7(Suppl 1)**:PP085

**Introduction**: Early recognition and epinephrine administration are essential in anaphylaxis management since delays in its diagnosis and treatment have been associated with fatal anaphylaxis. Validated 5-level paediatric triage systems prioritize anaphylaxis to Level I (Resuscitation) or II (Emergent). Inadequate triage of these patients may worsen their prognosis. We analysed the quality of the triage of children with anaphylaxis.

**Methods**: Retrospective review of the triage charts (local adaptation of the Canadian Paediatric Triage and Acuity Scale) of a cohort of children attended for anaphylaxis at a Paediatric Emergency Department (PED) between 2012 and 2016. According to their final triage level, we compared: demographics, waiting time for physician, initial triage level according to the initial assessment (Paediatric Assessment Triangle [PAT]), symptomatic patients during medical examination and epinephrine administration at PED and observations recorded by triage staff.

**Results**: We analysed 137 charts. Final triage levels were: Level I: 18.1%, Level II: 15.2%, Level III: 56.9% and IV:9.5%, respectively. Median ages were: 8.83 (IQR: 13.41y); II: 4.45 (IQR:13y); III: 4.5 (IQR:14.5y) and IV: 5.58 (IQR: 5.58y) (p = 0.09). Median waiting times for physician were: Level I: 0 (IQR: 22 min); Level II: 7 (IQR:22 min); III: 10 (111 min) and IV: 17 (IQR: 72 min) (p = 0.01). Patients with initial triage level V (stable PAT): Level I: 68%; II:71.4%, III: 91%; IV:92.3%(p = 0.006). Rates of symptomatic patients during medical examination and epinephrine administration at PED: Level I: 80%/76%; II: 57.1%/61.9%; III:48.6%/51.3% and IV: 53.8%/46.2% (p = 0.056 and p = 0.089). Symptoms compatible with anaphylaxis were recorded by triage staff in 28% of Level I, 33.3 of II, 39.7% of III and in 7.6% of level IV triage charts (p = 0.134)

**Conclusions**: Only 33.3% of the triage patients were triaged correctly. A significant number of the charts contained sufficient information to suspect an anaphylaxis. Inadequate triage delayed medical attention and treatment. Symptom recognition should be stressed out to triage possible anaphylaxis. Specific risk discriminators for anaphylaxis are needed to improve its identification during triage procedure.

### PP086 Pathways of anaphylaxis in an adjuvant-free mouse model

#### Bianca Balbino^1^, Riccardo Sibilano^2^, Philipp Starkl^3^, Thomas Marichal^4^, Nicolas Gaudenzio^2^, Hajime Karasuyama^5^, Pierre Bruhns^1^, Mindy Tsai^2^, Laurent L. Reber^1^, Stephen J. Galli^2^

##### ^1^Institut Pasteur, Paris, France; ^2^Stanford University School of Medicine, Palo Alto CA, USA; ^3^Medical University of Vienna, Vienna, Austria; ^4^University of Liege, Liege, Belgium; ^5^Tokyo Medical and Dental University, Tokyo, Japan

**Correspondence**: Bianca Balbino - bbalbino@pasteur.fr

*Clinical and Translational Allergy* 2017, **7(Suppl 1)**:PP086

**Introduction**: Conflicting results have been obtained regarding roles mast cells and other effector cells in models of active systemic anaphylaxis (ASA). In part, this might reflect the choice of adjuvant used during sensitization, as various adjuvants might differentially influence the production of particular antibody isotypes. Moreover, in humans, sensitization generally occurs in the absence of an artificial adjuvant. We therefore developed an adjuvant-free mouse model of ASA, and compared pathways of anaphylaxis in this model *vs*. in an ASA model using adjuvants for sensitization.

**Methods**: Mice were sensitized intra-peritoneally (i.p.) with ovalbumin (OVA) at weekly intervals for 6 weeks (adjuvant-free ASA model), or once i.p. with OVA together with Alum and *Bordetella pertussis* toxin (ASA model using adjuvants). Mice were then challenged i.p. with OVA two or three weeks later, respectively.

**Results**: Wild-type (WT) mice developed immediate hypothermia in both models, but significant mortality was only observed in the ASA model using adjuvants for sensitization. Depletion of monocytes/macrophages with clodronate liposomes significantly reduced anaphylaxis in both models. Depletion of neutrophils using anti-Gr-1 antibodies reduced anaphylaxis in the ASA model using adjuvants, but had no effect in the adjuvant-free ASA model. By contrast, anaphylaxis developed in mast cell-deficient *Kit* ^*W*-*sh/W*-*sh*^ mice in the ASA model using adjuvants, while it was markedly reduced in both *Kit* ^*W*-*sh/W*-*sh*^ mice and *Cpa3*-*Cre; Mcl*-*1* ^*fl/fl*^ mast cell-deficient mice in the adjuvant-free model. Finally, we found that combined treatment with the anti-histamine triprolidine and the platelet-activating factor (PAF) receptor antagonist CV-6209 almost entirely blocked anaphylaxis in the adjuvant-free ASA model.

**Conclusions**: Our data demonstrate that components of both the “classical” and “alternative” pathways contribute to anaphylaxis in the adjuvant-free ASA model. Monocytes/macrophages contribute to anaphylaxis regardless of the presence or absence of adjuvant during the sensitization, but the use of adjuvant in effect masks any non-redundant contribution of mast cells while revealing a contribution of neutrophils.

### PP087 Systemic neutrophilia during anaphylaxis in humans

#### Fabrícia Carolino, Ana Reis Ferreira, Josefina R. Cernadas

Serviço de Imunoalergologia, Centro Hospitalar São João, E.P.E., Porto, Portugal

**Correspondence**: Fabrícia Carolino - fabricia.c@sapo.pt

*Clinical and Translational Allergy* 2017, **7(Suppl 1)**:PP087

**Introduction**: To assess changes in WBC count (WBC-C) occurring during an episode of anaphylaxis in human subjects.

**Methods**: We conducted a search for hospitalizations in any unit of our institution (tertiary hospital) between Jan/10 and Jul/15 with an ICD9 code of anaphylaxis (995.0, 995.6, or 999.4). Medical records were reviewed and patients not fulfilling the currently accepted diagnostic criteria (Sampson HA *et al.* 2006) were excluded, as well as those without WBC-C performed during the episode.

**Results**: From Jan/10-Jul/15, 35 inpatients had one of the searched ICD9 codes. We excluded 9 (25.7%) patients not fulfilling anaphylaxis criteria, and 5 (14.3%) with no WBC-C, leaving 21 (60.0%) to be included in the analysis (61.9% females, 52.1 ± 25.6 years). Seventeen (81.0%) patients had anaphylactic shock. There were cardiovascular (CV) manifestations in 19 (90.5%) patients; 12 (57.1%) had mucocutaneous involvement and 12 (57.1%) respiratory signs/symptoms. Adrenaline was scarcely used. Drugs were the main suspect (n = 17, 81.0%); only 7 (33.3%) patients were referred to an Allergist. Tryptase levels were determined in 4 (19.0%) patients and were increased in 3. During the anaphylactic episode, 12 (57.1%) patients presented a *de novo* increase in peripheral relative and absolute neutrophil counts (mean ± SD: 85.8 ± 5.9% and 13.8 ± 7.1 × 10^−9^/L, respectively), 8 (66.7%) also with leucocytosis; 5 (23.8%) patients had a fall in blood neutrophils. Two (9.5%) fatal outcomes occurred (1 with neutrophilia). The limited sample size may be explained by the decision of including only hospitalized patients, fact that may justify the greater percentage of anaphylactic shock. Our analysis revealed clear reduced tryptase-dosing rates and adrenaline underuse. Although it is the common understanding that anaphylaxis associates with blood neutropenia, an increase in systemic neutrophils has been previously described in non-human anaphylaxis models and changes in blood-leukocyte populations were pointed as possible markers for severe shock in mice.

**Conclusions**: To the authors’ knowledge this is the first study to describe the occurrence of systemic neutrophilia in humans in the context of anaphylaxis. Despite the limited sample size, our study is ongoing and the authors believe that this is a promising field of research in understanding the complexity of anaphylaxis.

### PP088 Evaluation of knowledge of anaphylaxis in adolescents and implementation of an educational programme in schools in our area

#### Aida del Campo García, Sara Pereiro Fernández, Nerea Sarmiento Carrera, Fernando Bandrés Sánchez-Cruz, José Ramón Fernández Lorenzo

##### Hospital Álvaro Cunqueiro, Vigo, Spain

**Correspondence**: Aida del Campo García - palas89@hotmail.com

*Clinical and Translational Allergy* 2017, **7(Suppl 1)**:PP088

**Introduction**: To establish the knowledge that exists among adolescents in our region about anaphylaxis and its treatment. To determine the usefulness of a theoretical and practical workshop on the management of anaphylaxis and adrenaline auto-injectors among adolescents.

**Methods**: We conducted two methodological approaches: a descriptive study of knowledge about anaphylaxis in a cohort of secondary students through a questionnaire; an analytical quasi-experimental study comparing the previous results with those obtained after conducting a workshop on anaphylaxis. The workshop consisted of a theoretical and practical training of 45 min.

**Results**: We included 115 teenagers from 13.74 years on average, being the 13.9% of them allergic to some food. Before the workshop, the 82.6% of teenagers recognized anaphylaxis symptoms. Later, this percentage reached the 87.2%. The 41.7% of them considered the adrenalin as a treatment, a slightly higher (56%) percentaje among allergic. After the workshop, the 100% of respondents considered the adrenalin as a first choice, which means a statistically significant change. With regard to the correct use of auto-injectors, it was well identified by the 42.6% of students before the workshop and by the 97.2% of them thereafter. The most frequent cause of error was the location of the injection, followed by the device clamping. In the questions formulated as a clinical case before the workshop, the 41.7% of respondents would administer adrenalin to a patient with anaphylaxis. This figure increased among allergic up to 50%; they would then reach the 99.1%, which is a statistically significant change. It is common for adolescents to develop their activities accompanied by other teenagers, without the presence of their family and caregivers. Hence the importance of a universal education in anaphylaxis in school. We plan to carry out our study on a pilot basis, in order to assess its expansion to other schools in our region. We observed a statistically significant increase in adolescents that identify anaphylaxis and properly use adrenaline.

**Conclusions**: There is a significant lack of knowledge about anaphylaxis among teens in our sample. The development of a theoretical and practical workshop on Anaphylaxis and the management of self-injectable epinephrine in their education significantly increases their knowledge and makes us consider its universalization.

### PP089 Anaphylaxis in 939 patients aged 65 or more: data from Germany, Austria and Switzerland

#### Stephanie Claus^1^, Sabine Dölle^2^, Linus Grabenhenrich^3^, Claudia Pföhler^4^, Franziska Ruëff^5^, Kathrin Scherer^6^, Margitta Worm^2^, Regina Treudler^1^, Network for Online Registration of Anaphylaxis (NORA)

##### ^1^Department of Dermatology, Venereology and Allergology, Comprehensive Allergy Center, University Hospital, Leipzig, Germany; ^2^Department of Dermatology and Allergology, Comprehensive Allergy Center, Charité - Universitätsmedizin Berlin, Berlin, Germany; ^3^Institute for Social Medicine, Epidemiology and Health Economics, Charité - Universitätsmedizin Berlin, Berlin, Germany; ^4^Department of Dermatology and Allergology, Saarland University Hospital, Homburg, Germany; ^5^Department of Dermatology and Allergology, Ludwig-Maximilians-University, Munich, Germany; ^6^Allergy Unit, Department of Dermatology, University Hospital, Basel, Switzerland

**Correspondence**: Sabine Dölle - sabine.doelle@charite.de

*Clinical and Translational Allergy* 2017, **7(Suppl 1)**:PP089

**Introduction**: Anaphylaxis in children and adults differs with regard to elicitors and clinical picture. Little is known about anaphylaxis in elderly patients. We aimed at characterizing typical features of anaphylaxis in this group of patients.

**Methods**: Data from the Network for Online Registration of Anaphylaxis (NORA) were analyzed for German speaking countries (Germany, Switzerland and Austria). We compared data from patients aged ≥65 (elderly) with data from patients aged 18–64 (main adult group) in terms of elicitors, clinical symptoms, comorbidities and emergency treatment.

**Results**: Between July 2007 and March 2016, anaphylaxis was registered in 939 elderly and 4665 of other adult patients. Insect venom was the most frequent trigger in both groups (elderly: 60% vs. adults: 52%), followed by drugs (24% vs. 21%) and food items (10% vs. 17%). Within the group of insects yellow jacket (72% vs. 73%) and in the group of drugs analgesics (38% vs. 40%) were the most common elicitors. For food anaphylaxis hazelnut (16%) was the most frequent elicitor in the elderly, and wheat (16%) in the other adults. Cardiovascular symptoms were slightly more prevalent in the elderly (77% vs. 73%) and the reactions are more severe with 46% (36% compared to other adults) experiencing a grade III/IV reaction. Noteworthy, 60% of the elderly had a preexisting cardiovascular comorbidity compared with 18% of the other adults. First line treatment by professionals included mainly corticosteroids (89%) and antihistamines (82%) in both groups. Epinephrine was only used in 24% (elderly) versus 19% (other adults).

**Conclusions**: Compared to adults aged below 65 years, the symptom pattern in the elderly was characterized by cardiovascular symptoms and more severe reactions. Epinephrine was used more frequently in the elderly compared to younger adults but was still only used in less than one out of four patients.

**Acknowledgements**: Network for Online Registration of Anaphylaxis (NORA) participating centers can be found under www.anaphylaxie.net.

### PP090 Improvement of the knowledge of teachers about anaphylaxis after training workshops

#### Mercedes Escarrer Jaume^1^, Agustin Madroñero^1^, Maria Teresa Guerra Perez^2^, Juan Carlos Julia^3^

##### ^1^Juaneda Hospital, Palma de Mallorca, Spain; ^2^Jerez, Spain; ^3^Valencia, Spain

**Correspondence**: Mercedes Escarrer Jaume - m.escarrer@gmail.com

*Clinical and Translational Allergy* 2017, **7(Suppl 1)**:PP090

**Introduction**: Improvement of the knowledge of teachers about anaphylaxis after training workshops.

**Methods**: In 2014 we conducted a questionnaire to 2481 teachers, where it became evident that the knowledge about anaphylaxis was scarce [1]. Only 40.5% knew what anaphylaxis was, and only 11% said that they knew how to administer adrenaline auto-injector, for this reason we conducted training teachers with theory and practice, with demonstration videos and developing action protocols in schools.

**Results**: After a year we made training to 296 teachers of the Balearic Islands, with a theoretical practical course of 4 h, at the end we had a questionnaire on knowledge of anaphylaxis and all passed the test. According to the EAACII 1 in 300 Europeans suffer anaphylaxis at some point in their life and most European schools have at least one child at risk of anaphylaxis food allergy. It is very important that teachers are trained in the recognition and early treatment of an anaphylactic reaction

**Conclusions**: The training of teachers is important along with videos and protocols which explain simply how to recognize and treat an anaphylactic reaction.

**Reference**

1. https://www.youtube.com/watch?v=ea9kPsHBv_s

### PP091 Validation of a targeted mass spectrometry method for confirmation of peanut allergens in serum

#### Charlotte Hands^1^ Plovdiv, Lee Gethings^2^, Jim Langridge^2^, Karine Adel-Patient^3^, Hervé Bernard^3^, Ivona Barcievic-Jones^1^, Rebekah Sayers^1^, Clare Mills^1^

##### ^1^Manchester Institute of Biotechnology, University of Manchester, Manchester, United Kingdom; ^2^Waters Corporation, Wilmslow, United Kingdom; ^3^INRA-CRJJ, Jouey-en-Josas, France

**Correspondence**: Charlotte Hands - charlotte.hands@postgrad.manchester.ac.uk

*Clinical and Translational Allergy* 2017, **7(Suppl 1)**:PP091

**Introduction**: The iFAAM project is investigating whether the passage of allergen into the circulation may contribute to determining the severity of an allergic reaction by comparing uptake in healthy individuals and individuals undergoing oral food challenge. A mass spectrometry based method to detect the presence of peanut allergens in serum samples is being developed which will allow confirmation of peanut allergens in serum identified by other methods such as immunoassay and mediator release.

**Methods**: A set of heavy labelled tryptic peptide targets for detection of peanut allergens Ara h 1, Ara h 3 and Ara h 2, 6 and 7 have been used to develop a targeted mass spectrometry (MS) method using multiple reaction monitoring, for detection of peanut in serum. Using serial isotopic dilution (SID) series and blank serum spiked with a peanut protein extract, initial validation studies have been undertaken exploring the use of different depletion methods to remove the most abundant serum proteins. MS analysis has made use of different TOF and triple quadrupole platforms.

**Results**: Undepleted serum samples had a marked matrix effect on SIDs, likely due to the presence of other serum peptides causing suppression effects on peanut peptide ionisation. These effects were less marked in depleted serum samples. Analysis of peanut spiked into blank serum and analysed after depletion showed differential matrix effects, with some peanut peptides having been lost during the depletion process. The general limit of detection of peanut peptide targets was achieved in the femtomolar range, on column.

**Conclusions**: Further method development is required to optimise serum depletion, trypsin digestion and chromatographic separation steps to allow detection of peanut at levels likely to be found in human subjects after ingestion of peanut. This mass spectrometry method shows promise as a complimentary tool to immunoassay methods to detect the presence of peanut in serum.

### PP092 Anaphylaxis, acute urticaria and angioedema in patients with diabetes mellitus type 2

#### Raditsa Sokolova^1^, Rumyana Yankova^1^, Mariya Ivanovska^2^, Marianna Murdjeva^2^, Tatyana Popova^2^, Svetlan Dermendzhiev^3^

##### ^1^Department of Dermatology and Venereology, Medical University Plovdiv, Plovdiv, Bulgaria; ^2^Department of Microbiology and Immunology, Medical University Plovdiv, Plovdiv, Bulgaria; ^3^Department of Allergology and Occupational Medicine, Medical University Plovdiv, Plovdiv, Bulgaria

**Correspondence**: Mariya Ivanovska - marijaku87@yahoo.com

*Clinical and Translational Allergy* 2017, **7(Suppl 1)**:PP092

**Introduction**: The aim of this study is to determine the incidence of anaphylaxis, acute urticaria and angioedema during of years 2012–2015 in patients with diabetes mellitus type 2.

**Methods**: We performed a prospective study of the frequency of anaphylaxis, acute urticaria and angioedema during of years 2012–2015 in patients with diabetes mellitus type 2. A total of 83 patients with diabetes mellitus type 2 of the district of Plovdiv were included in the study.

**Results**: With a history of anaphylaxis were 4.8% from diabetics. With history of acute urticaria and angioedema were 26.5% diabetics. In all diabetics with a history of anaphylaxis, the symptoms were due to drugs (antibiotics and general anesthetics). In all diabetic patients with a history of urticaria and angioedema, the symptoms also due to drugs (antibiotics 36.3%, sulfonamide 13.6%, ACE inhibitors 13.6%, alpha-lipoic acid 13.6%, general anesthetics 9.1% and other) except one who reported insect bites. 9.1% of patients with a history of urticaria and angioedema were reported symptoms of anaphylaxis. Comorbidity and increased incidence of infections requiring antibiotic and concomitant treatment, most likely causes an increase in the risk of developing hypersensitivity reactions induced by drugs.

**Conclusions**: The most frequent causes of anaphylaxis, acute urticaria and angioedema in patients with diabetes mellitus type 2 are antibiotics, general anesthetics, sulfonamide, ACE inhibitors and alpha-lipoic acid.

### PP093 Retrospective database analysis on anaphylaxis and patient concordance in the United States

#### Martin Karjalainen^1^, Ulrike Lehnigk^1^, Duncan Brown^2^, Julie C. Locklear^3^

##### ^1^Allergopharma GmbH & Co. KG, Reinbek, Germany; ^2^Xcenda, LLC., Palm Harbor FL, USA; ^3^EMD Serono, Inc., Rockland MA, USA

**Correspondence**: Ulrike Lehnigk - ulrike.lehnigk@allergopharma.com

*Clinical and Translational Allergy* 2017, **7(Suppl 1)**:PP093

**Introduction**: The objective was to understand and evaluate patient characteristics, concordance with post discharge care, health care resource utilization and repeated events among adults and children with an inpatient or Emergency Department (ED) claim for food or non-food induced anaphylaxis in the United States.

**Methods**: For retrospective analysis the Truven Healthcare MarketScan^®^ Commercial and Medicare Supplemental and COB Databases was used to (1) identify the patient profiles with inpatient or ED claims for anaphylactic shock, stratified to food-based allergic reactions and to allergic reactions due to non-food or unknown causes; (2) examine the event characteristics and healthcare resource use (HCRU) related to the event and (3) assess Epinephrine Auto-Injector (EAI) prescriptions pre- and post-event and the HCRU associated with these prescriptions.

**Results**: The study comprised 10,189 adults (age >18) and 3891 pediatric patients (age <18). Those patients treated in the ED present with acute respiratory failure (3.8%), hypotension (5.0%), and in rare cases cardiac arrest (0.3%). While in the ED, intervention included resuscitations (25.4%), intubation (1.6%), tracheostomy (2.0%), epinephrine administration (13.2%), and cardiopulmonary resuscitation (0.1%). 11.7% of patients seen in the ED were admitted to inpatient care and spent 2.7 days on average in the hospital. At the time of the index event 83.3% of patients did not have a prescription for an EAI filled. Only 12.1% of patients had the minimum of 1 EAI prescription refill. The mean number of days from last prescription to anaphylactic event was 2350 days. A significant portion of patients are transitioned to inpatient care from the ED resulting in greater healthcare utilization. The data indicate that patients demonstrate suboptimal maintenance of EAI prescriptions placing them at risk for life threatening events and questioning the preparedness of patients and caregivers for the management of an anaphylactic event. Given the low active prescription level it appears patients are not proactively prepared for an anaphylactic event which may lead to costly ED visits and hospital admissions.

**Conclusions**: Current educational programs and safety information delivered to patients and caregivers regarding the use of EAIs to treat anaphylaxis are not effective.

### PP094 Weaknesses of treatment guidelines for the management of anaphylaxis and healthcare utilization following an anaphylaxis event

#### Ulrike Lehnigk^1^, Martin Karjalainen^1^, Duncan Brown^2^, Julie C. Locklear^3^

##### ^1^Allergopharma GmbH & Co. KG, Reinbek, Germany; ^2^Xcenda, LLC., Palm Harbor FL, USA; ^3^EMD Serono, Inc., Rockland MA, USA

**Correspondence**: Ulrike Lehnigk - ulrike.lehnigk@allergopharma.com

*Clinical and Translational Allergy* 2017, **7(Suppl 1)**:PP094

**Introduction**: Intramuscular epinephrine (adrenaline) is classified as first-line treatment of choice for anaphylaxis in international and national guidelines. Patients at risk are recommended to be prescribed 2 epinephrine auto-injectors (EAIs) which they should carry with them at all times. A prospective, web-based survey was conducted to obtain insight into adherence to treatment recommendations according guidelines, and post-anaphylaxis behavior in patients at risk for anaphylaxis and their caregivers.

**Methods**: 505 patients aged 18–65 years (mean = 30.4; 26.1% male) and 448 caregivers of individuals under aged 18 years (mean = 24.4; 33.3% male) were recruited in the United States between the 15th and 30th of November, 2015. All participants had been prescribed an EAI for self-administration or for administration as a caregiver.

**Results**: At home, 16.8% of patients and 14.3% of caregivers had no EAI and 61.0% of patients and 58.5% of caregivers indicated 1 EAI was available. At their workplace, 81.8% of patients did not have access to at least 1 EAI and 32.6% of caregivers reported that their child did not have access to at least 1 EAI at school. Only 9.6% of caregivers identified that 2 EAIs were available for their children at school. Of those who received emergency care, 4.6% received their first dose of epinephrine in the Emergency Department. 6.0% of patients reported having to call emergency services due to the lack of a secondary EAI with 2.8% being admitted to inpatient care. Following utilization of an EAI, 23.8% of patients and 21.4% of caregivers did not seek follow-up medical attention. Given the lifesaving ability of EAIs, the effect of treatment according to guidelines is imperative to successful manage anaphylactic events. The data call into question the efficacy of current educational programs and safety information delivered to patients and caregivers regarding the access to and the use of EAIs to treat anaphylaxis.

**Conclusions**: This survey revealed poor guideline adherence by patients and caregivers: most patients did not carry the guideline recommended number of 2 EAIs and did not seek emergency treatment after using an EAI. Reasons for this behavior should be investigated in further studies to improve the utilization of this life saving treatment.

### PP095 Patient and caregiver reported problems in the utilisation of epinephrine auto-injectors for management of severe allergic reactions

#### Ulrike Lehnigk^1^, Martin Karjalainen^1^, Duncan Brown^2^, Julie Locklear^3^

##### ^1^Allergopharma GmbH & Co. KG, Reinbek, Germany; ^2^Xcenda, LLC., Palm Harbor FL, USA; ^3^EMD Serono, Inc., Rockland MA, USA

**Correspondence**: Ulrike Lehnigk - ulrike.lehnigk@allergopharma.com

*Clinical and Translational Allergy* 2017, **7(Suppl 1)**:PP095

**Introduction**: A prospective, web-based study was conducted to understand causes for utilization problems of epinephrine auto-injectors (EAIs) among patients and caregivers of patients with anaphylaxis.

**Methods**: 505 patients aged 18–65 years (mean = 30.4; 26.1% male) and 448 caregivers of individuals under aged 18 years (mean = 24.4; 33.3% male) were recruited in the United States. All participants had been prescribed an EAI for self-administration or for administration by a caregiver. The survey took place between the 15^th^ and 30^th^ of November, 2015.

**Results**: The most common administration problems included: EAI was pressed too firmly against the skin (11.1%), needle was bent during injection (7.4%), and did not allow enough time for the injection to complete (8.7%). When asked to identify factors contributing to the cause of injection problems, caregivers were more likely to attribute problems to improper training (p = 0.01), lack of understanding on how to use the EAI after training (p = 0.03), and difficulty in use (p = 0.003) as compared to patients. Only 16.4% of all participants reporting a problem immediately realized there was an issue with the EAI injection while 6.0% of participants realized the EAI did not work properly upon the worsening of symptoms. Results indicate that the causes of injection problems were similar among patients and caregivers. The findings call into question the efficacy of current educational programs and safety information delivered to patients and caregivers regarding the use of EAIs to treat anaphylaxis.

**Conclusions**: Education of patients and caregivers about current anaphylaxis treatment guidelines and the safety and easy use of an EAI that is directed at health care professionals may not be working well. Reasons for non-adherence to anaphylaxis treatment guidelines should be further assessed in future studies.

### PP096 ED management of pediatric anaphylaxis in different hospital settings in Ireland

#### Ioana Maris, Jonathan Hourihane

##### Cork University Hospital, University College Cork, Cork, Ireland

**Correspondence**: Ioana Maris - imaris@ucc.ie

*Clinical and Translational Allergy* 2017, **7(Suppl 1)**:PP096

**Introduction**: Anaphylaxis is a medical emergency and professional first-line treatment is administered mainly by emergency physicians in most studies.

Our aim was to compare emergency management of paediatric anaphylaxis in three different hospital settings—a joint adult and paediatric ED associated with the national referral centre for paediatric allergy versus specialized paediatric ED versus general ED.

**Methods**: As part of the NORA European Anaphylaxis initiative, we undertook a project on the incidence of anaphylaxis in Irish children. Hospital emergency response was assessed looking at early use of i.m. adrenaline, use of second-line medication, and discharge planning/prescription of emergency drugs.

**Results**: 133 cases of paediatric anaphylaxis were reported to attend EDs in Ireland between August 2013—May 2015: 51 cases (38.3%) presented to the national referral centre for paediatric allergy with a tertiary hospital general ED, 46 cases (34.6%) to a children’s hospital with a paediatric ED, and 36 (27.1%) to a regional hospital with general ED. There was no statistically significant difference in first line administration of i.m. adrenaline between centres (55.6% of cases overall, Fisher’s Exact Test p = 0.48). All cases were prescribed AAIs in the national allergy centre, compared to 62.8% in the paediatric ED, and 77.7% in the general ED.

**Conclusions**: Attendance to the ED associated with an allergy centre improves AAI prescription but does not improve adrenaline use for the presenting emergency. Usage of i.m. adrenaline as first line treatment needs to be improved.

### PP097 Theoretical and clinical dose built-up in oral immunotherapy using IFN-gamma by calibration during oral challenge test for anaphylactic food allergy

#### Geunwoong Noh

##### Department of Allergy, Jeju Halla General Hospital, Jeju Halla, Korea Republic

**Correspondence**: Geunwoong Noh - admyth@naver.com

*Clinical and Translational Allergy* 2017, **7(Suppl 1)**:PP097

**Introduction**: Oral immunotherapy using IFN-gamma has been done for more 10 years successfully. The dosage modulation of treatment including initial dose and incremental dose for the treatment is absolutely necessary in the clinical field. Dosage modulation is also the issue during the treatment. For these issues, the theoretical and clinical backup is also needed. The aims of this study are the built up the dosage modulation by dosage calibration during oral challenge test and during the treatment for the precise oral immunotherapy using IFN-gamma for anaphylactic food allergy. Also, the theoretical principle is also suggested.

**Methods**: Patients who had anaphylactic food allergy for milk, eggs, soybean and wheat were selected. Basic allergic laboratory tests were done including allergen-specific IgE and skin prick tests. Oral food challenge tests were conducted. Especially, the minimal dose and clinical severity were checked during oral immunotherapy. The initial dose was determined by the minimal dose and incremental dose was determined by the clinical severity. During the treatment, the incremental doses were escalated according to the patient’s responses to dosage. The duration and effectiveness of treatment is compared and evaluated between the classic methods and the advanced methods applying the concepts of calibration and modulation of dosage.

**Results**: The trial of calibration is proper for oral immunotherapy using IFN-gamma for anaphylactic food allergy. By dosage calibration, the duration and the effectiveness of oral immunotherapy using IFN-gamma was much improve, significantly. By dosage increment, the incremental dose was escalated exponentially. The basic theory of oral immunotherapy using IFN-gamma is depending on the tolerogenic effects of IFN-gamma. The allergy provocation strength should be within the range of tolerogenic effects of IFN-gamma. During oral immunotherapy using IFN-gamma for anaphylactic food allergy, patients were getting tolerance for last therapeutic dose. The meaning of allergy provoking dose and strength did not follow the absolute dose. The allergy provoking dose seems to be the dose of difference between the therapeutic dose and tolerable dose.

**Conclusions**: For more precise and effective treatment of oral immunotherapy for anaphylactic food allergy, the dosage calibration during oral challenge test is absolutely necessary for the determination of initial dose and incremental dose for the treatment. Also, modulation of dosage should follow the coverage range of tolerogenic effects of IFN-gamma. From the results of clinical application of dosage modulation, the framework for the dosage modulation was built up with several terminologies for the theoretical and clinical setting.

### PP098 Follow-up of patients admitted for anaphylaxis – A tertiary hospital experience

#### Cristina Ornelas, Joana Caiado, Manuel Branco Ferreira, Manuel Pereira Barbosa

##### Imunoallergology Department, Centro Hospitalar Lisboa Norte, Hospital Santa Maria, Lisbon, Portugal

**Correspondence**: Cristina Ornelas - cristina.ornelas@gmail.com

*Clinical and Translational Allergy* 2017, **7(Suppl 1)**:PP098

**Introduction**: Anaphylaxis is a severe and pottentialy life-threatening systemic hypersensitivity reaction and patients (pts) should be closely monitored. At discharge it’s important to assure follow-up in order to assess culprit(s) agent(s).

Our aim was to characterize follow-up of pts admitted for anaphylaxis’s surveillance at the Imunoallergology Department.

**Methods**: Retrospective analysis of clinical files of pts admitted for anaphylaxis between January 1, 2005 and April 30, 2016.

**Results**: 144 pts were admitted for anaphylaxis in our department (57% female sex; mean age 44.8 years). 129 pts were referred for follow-up (remaining 15 had already follow-up in other centers). Investigation was started in 92 (37 missed first appointment). Suspected etiologies were: drugs (43; 3 during drug challenge or desensitization; >1 drug suspected in 2 pts), foods (36; 1 during oral food challenge; >1 food involved in 5 pts), foods or drugs (3), hymenoptera venom (1) and iodinated contrast media (ICM) (1). Etiology was unknown in 8 cases. Suspected drugs were betalactams (15), other antibiotics (6), nonsteroidal anti-inflammatory drugs (15), acetaminophen (4), local anesthetics (1), proton pump inhibitors (1) and angiotensin 2 receptor antagonists (1). The suspected drug was confirmed in 6 cases and excluded in 3; 12 pts are still under investigation and 22 were lost to follow-up. Suspected foods were fresh fruits (13), seafood (11), fish (3), nuts (4), milk (2), seeds (4), spice (2), wheat (2) and honey (1)—suspected etiology was confirmed in 24 cases, excluded in 9 and under investigation in 3; 7 cases were lost to follow-up. In 3 pts with both etiologies suspected, only one of them was confirmed for each (food-2; drug-1). The hymenoptera suspected by clinical history was wasp but we confirmed sensitization to polistes. The investigation in ICM reaction was negative. Regarding the 8 pts with unknown etiology, 4 are being studied, 3 were lost to follow-up and 1 revealed food allergy (LTP sensitization). Overall, from the 92 pts that came to a first appointment after hospital discharge, 42 have a confirmed anaphylaxis’s etiology; 32 pts were lost to follow-up.

**Conclusions**: Long-term management of patients who experienced anaphylaxis may be long and time-consuming but it’s crucial to identify the etiologic agent in order to avoid the culprit and minimize the risk of further reactions. In our cohort more than half of the pts doesn’t have a confirmed culprit allergen, mainly due to a high dropout rate.

### PP099 Analysis of 150 anaphylaxis episodes

#### Yolanda Puente^1^, Juan Carlos Daza^1^, Francisco Javier Monteseirin^2^

##### ^1^Hospital Universitario Virgen Macarena, Seville, Spain; ^2^University of Seville, Seville, Spain

**Correspondence**: Yolanda Puente - doctorapuente@gmail.com

*Clinical and Translational Allergy* 2017, **7(Suppl 1)**:PP099

**Introduction**: Anaphylaxis is the most severe manifestation of an allergic reaction and recent data indicates a continuous increase of cases. The aim of this study was to evaluate the characteristics of anaphylaxis in 150 patients who had been studied by allergists in a geographical area.

**Methods**: This descriptive study involved a retrospective review of records of 150 patients who had suffered from anaphylaxis and had been carefully studied in our center. All of them belonged to the same geographical area. The median age was 31.59 years, from 0 to 80 years. 81 of them were female.

**Results**: 52 of them were caused by food, 40 cases were due to drug allergy and diagnosis agents or immunotherapy. In addition, 29 patients had anaphylaxis because of hymenoptera allergy. Only 18 of them were related to exercise-induced, idiophatic etiology or *Anisakis simplex*. On the other hand, the majority of patients had only suffered from one anaphylactic reaction. The 72.01% of successive episodes were related to food allergy. The average of episodes by patient was 1.44 with a range from 1 to 10. 44.29% suffered from pollen allergy. Most of them happened in the springtime. The average of serum basal tryptase levels was 1.39 µg/l, range 0.1–40. More than 50% of patients carried their epinephrine auto-injector with them at all times.

**Conclusions**: Anaphylaxis is a relatively common problem and it is quite necessary to improve the current understanding would be through. In our opinion, food allergy is the most important trigger of anaphylaxis including recurrent episodes. Unfortunately a lot of patients do not carry their self-administered epinephrine injector, underusing it in the treatment of anaphylactic reactions. On the other hand, an improved understanding of this disorder would aid ongoing efforts to reduce it and could provide clues for its prevention.

### PP100 Paracetamol: a rare culprit in anaphylaxis

#### Ana Rodolfo, Fabrícia Carolino, Eunice Dias de Castro, Josefina R. Cernadas

##### Immunoallergology Department, Centro Hospitalar de São João, E.P.E., Porto, Portugal

**Correspondence**: Fabrícia Carolino - fabricia.c@sapo.pt

*Clinical and Translational Allergy* 2017, **7(Suppl 1)**:PP100

**Introduction**: Anaphylaxis to paracetamol is rare and the underlying mechanisms are poorly understood. We aimed to review the cases of anaphylaxis to paracetamol in our Drug Allergy Unit (DAU) in the last five years.

**Methods**: The authors did a retrospective analysis of all cases of anaphylaxis to paracetamol studied in our DAU between January 2011 and December 2015. The data were analysed using SPSS, version 21.

**Results**: Twelve adult patients with anaphylactic reaction to paracetamol were studied (67% males, aged 18–72y), 25% had allergic respiratory disease. All patients were medicated with oral paracetamol for pain relief. Six patients also had anaphylaxis to nonsteroidal anti-inflammatory drugs (NSAID’s): acetylsalicylic acid (n = 4), ibuprofen (n = 3), naproxen (n = 1), nimesulide (n = 1) and diclofenac (n = 1). Nine patients had immediate reactions (75%), and 3 (25%) late reactions (onset 3–12 h). Eleven patients (92%) had mucocutaneous involvement, 11 respiratory manifestations, 3 (25%) cardiovascular and 1 (8%) gastrointestinal. None had anaphylactic shock. Only 8 (67%) patients were admitted to the emergency department (ED), none was treated with adrenaline nor had tryptase determined. Because the *in vivo* work up of these patients is very limited, they all underwent an oral challenge (OC) with an alternative drug (etoricoxib) and tolerance was confirmed. In the 6 cases of multiple-reactors, the authors postulate an underlying COX-1 inhibition mechanism since paracetamol is a weak inhibitor of COX-1.In the cases of single-reactors (SR) IgE-mediated mechanisms may be involved. A possible explanation for the late anaphylaxis (12 h) in a SR is COX-1 inhibition; alternatively, IgE involvement may be considered if this “late anaphylaxis” is in fact the late manifestation of a bifasic response. The *in vitro* study of this patient is in progress.

**Conclusions**: Paracetamol is a widely used drug, available over-the-counter in most countries, but not entirely without risks. The allergy diagnosis work-up in cases of anaphylaxis to paracetamol is difficult and mostly based on a detailed clinical history. Skin tests are not standardized and positive results are doubtful. *In vitro* assays such as specific IgE and Basophil Activation Test have poor specificity and sensitivity. Moreover OC with the culprit drug are contraindicated in anaphylaxis.

### PP101 Recurrent anaphylaxis in a patient with LTP syndrome

#### Natalia Ukleja-Sokolowska^1^, Ewa Gawronska-Ukleja^1^, Magdalena Zbikowska-Gotz^1^, Zbigniew Bartuzi^1^, Lukasz Sokolowski^2^

##### ^1^Department of Allergology, Clinical Immunology and Internal Diseases, L. Rydygier Collegium Medicum, Bydgoszcz NCU, Bydgoszcz, Poland; ^2^Division of Ergonomics and Exercise Physiology, L. Rydygier Collegium Medicum in Bydgoszcz NCU, Bydgoszcz, Poland

**Correspondence**: Natalia Ukleja-Sokolowska - ukleja@10 g.pl

*Clinical and Translational Allergy* 2017, **7(Suppl 1)**:PP101

**Introduction**: Lipid transfer proteins are a wide spread family of food allergens. Sensitization usually occurs through the consumption of LTP containing food and the primary sensitizer usually is peach. The marker of LTP syndrome is an elevated level of peach LTP—Pru p 3, in some cases mugwort LTP—Art v 3. The disease is often variable and unpredictable.

**Case report**: Patient, 38, female, was admitted to the Department of Allergology, Clinical Immunology and Internal Diseases in December 2015 because of recurrent symptoms of anaphylaxis: swelling of the upper and lower limbs, edema of both eyelids, lips and tongue, with shortness of breath and increased heart rate. Only in 2015, she experienced 4 anaphylactic reactions after eating poppy seed, sunflower seed and peach. In 2013 Polycheck found elevated level of IgE specific to peach (5,5 kU/L), apple (10 kU/L), rye flour (2,7 kU/L), wheat flour (0,39 kU/L). During hospitalization we performed skin prick test, spirometry, established the level of asIgE directed against selected allergens and the level of IgE directed against allergen components using ImmunoCap ISAC. SPT were positive to weed, *Artemisia vulgaris*, cat, birch, alder, carp, strawberry, wheat and rye flour. Patient had elevated level of IgE specific to rye flour—1.38 IU/ml and peach—2.02 IU/ml, but the level of specific IgE against wheat flour and apple was <0.35 IU/ml. In ImmunoCap ISAC we found elevated levels of IgE directed against nsLTP from different plant sources: rAra h 9—7 ISU-E, rCor a 8—2 ISU-E, nJug r 3—4,7 ISU-E, rPru p 3—6,3 ISU-E, nArt v 3—1,4 ISU-E, nOle e 7—1,4 ISU-E, rPla a 3—3,4 ISU-E. Thye level of IgE directed against all other allergen components available in ISAC were not elevated.

**Conclusion**: Results of ImmunoCap ISAC changed the way we interpret this case. Before the results it was possible to suspect pollen-food syndrome, although it was not consistent with the clinical picture. In ImmunoCap high levels of IgE specific to Pru p 3 and Art v 3, markers of LTP syndrome, allow as to diagnose patient with this disease. We explained the nature of the disease and the need to carry rescue kit containing epinephrine in auto-syringe. This patient could benefit from Pru p 3 immunotherapy.

**Consent to publish**: Patient consented to the publication of this abstract.

### PP102 Anti-allergy effects of whey protein hydrolysates in human peripheral blood mononuclear cells

#### Aine Adams, Bernard Mahon, Karen English

##### Food for Health Ireland (FHI), Cellular Immunology Lab, Department of Biology, Institute of Immunology, Maynooth University, Maynooth, Ireland

**Correspondence**: Aine Adams - aine.adams@gmail.com

*Clinical and Translational Allergy* 2017, **7(Suppl 1)**:PP102

**Introduction**: 20 to 30% of infants in Europe are diagnosed with an atopic (allergic) disease. The majority of first atopic responses are directed towards food proteins that are consumed during the first months of life. There is increasing evidence that certain milk-derived components have positive effects on infant health. Ongoing research in this area has identified immunomodulating ingredients (milk-derived hydrolysates) which have potential to support the immune system in an allergy setting. Hydrolysing the proteins in infant formula is one approach in the management of allergenic responses in infants. These hydrolysed proteins which lack allergenic IgE binding sites can modulate T-cell differentiation away from a Th2 response and decrease inflammation. Our research focus is on identifying hydrolysates that can be added to infant formula to ameliorate cow’s milk allergy.

The aim of this study was to identify milk protein hydrolysates (peptides) with (anti-inflammatory/anti-allergy) properties using an *in vitro* approach.

**Methods**: To that end, hydrolysates were screened for their immunomodulatory properties in 4 cellular models, human peripheral blood mononuclear cells (PBMC), human monocyte dendritic cells (DC), T helper 1 (Th1) polarised and Th2 polarised cells. Specifically, T cell proliferation was analysed, before and after the addition of hydrolysates using a CFSE assay by flow cytometry and cytokine levels, released in the culture supernatants were measured by ELISA. DC maturation and cytokine production were also examined by flow cytometry and ELISA. Naïve human CD4^+^ T cells were activated with plate-bound anti-CD3 and anti-CD28 and then cultured under Th1 or Th2 polarising conditions with or without hydrolysates for 4 days. Transcription factor expression was then assessed by flow cytometry.

**Results**: A number of hydrolysates significantly decreased T cell proliferation driven by anti CD3/CD28 beads and several downregulated the maturation marker CD86 in DCs. Select hydrolysates increased the expression of T-bet and Gata-3, decreased production of the pro-inflammatory cytokines IFN-gamma and IL-6 and increased anti-inflammatory IL-10.

**Conclusions**: Thus far we have identified a number of hydrolysates which promote an anti- inflammatory /anti-allergy T cell and DC phenotype. Further work will examine these hydrolysates in an *in*-*vivo* humanised mouse model of allergy in order to confirm their protective impact.

**Acknowledgements**: Funding code: TC2013-001.

### PP104 Serum tryptase measurement is useful to assess clinical positivity of oral food challenge to hazelnut in children

#### Nelly Gourdon-Dubois^1^, Laetitia Sellam^2^, Bruno Pereira^3^, Elodie Michaud^2^, Khaled Messaoudi^4^, Bertrand Evrard^4^, Jean-Luc Fauquert^2^

##### ^1^CHU Clermont-Ferrand, INSERM CIC 1405, Clermont-Ferrand, France; ^2^Pediatric Allergy Unit, CHU Clermont-Ferrand, CHU Estaing, Clermont-Ferrand, France; ^3^Unité de Biostatistiques, Direction de la Recherche Clinique (DRCI), CHU Clermont-Ferrand, Clermont-Ferrand, France; ^4^Department of Immunobiology, UdA-INRA - CHU Montpied, CHU Clermont-Ferrand, Clermont-Ferrand, France

**Correspondence**: Nelly Gourdon-Dubois - ngourdondubois@chu-clermontferrand.fr

*Clinical and Translational Allergy* 2017, **7(Suppl 1)**:PP104

**Introduction**: Oral Food challenge (OFC) is the milestone to access to a definitive diagnosis of hazelnut allergy (HA). We evaluated variation of serum tryptase levels during blinded OFC.

**Methods**: 31 OFC were performed in children (20 M/11F) aged 10.5 ± 4.9 y, referred to the allergy outpatient paediatric Unit. All underwent a reaction after hazelnut ingestion and were therefore suspected for allergy. Single blind placebo controlled OFC was performed according to usual recommendations: increments of native hazelnut were added every 30 min (0.1, 0.2, 0.5, 1, 2, 5, and 20 grams). Challenge was declared positive when a single objective symptom occurred. Sensitization to hazelnut was confirmed previously to OFC (positive SPT or specific IgE measurements over 0.10 IU/mL) and serum Tryptase was measured in µg/L (ImmunoCAP Tryptase, Phadia^®^) before (T^b^) and one hour after the last increment of the challenge (T^f^). We calculated Tryptase variation ΔT = T^f^ − T^b^ and Tryptase relative variation ΔT/T^b^. Comparisons between the groups were performed using the Chi square tests for categorical variables, and by the Student t-test or Kruskal-Wallis test for quantitative parameters.

**Results**: Among the 31 patients, 8 had a positive OFC to hazelnut (n = 8 OFC+) and 23 negative (n = 23 OFC-). At baseline the two groups of patients (OFC+ and OFC-) were similar in terms of sex, age, and reaction occurred when the diagnosis was settled. T^b^ dosage did not differ significantly between OFC+ patients (4.83 ± 5.57 µg/L) and OFC- patients (3.26 ± 1.39 µg/L). T^f^ was significantly higher in OFC+ patients (9.78 ± 11.13 µg/L) than in OFC- patients (3.30 ± 1.41 µg/L) (p = 0.03). Variation of serum Tryptase (ΔT) was significantly higher (p < 0.001) in OFC+ patients (4.95 ± 6.67) than OFC- patients (0.04 ± 0.57). Moreover the relative variation of serum Tryptase ΔT/T^b^ was significantly higher (p < 0.001) in OFC+ patients (102 ± 145%) than in OFC- patients (3.74 ± 21.8%). Range of ΔT/T^b^ varied from +11 to +450% in OFC+ patients and from −37 to +70%. Among the OFC- patients, all except 4 had a negative variation of Tryptase whereas none of the OFC+ had a negative variation of ΔT/T^b^, thus confirming the good negative value of ΔT/T^b.^ ΔT/T^b^ was higher in patients with positive SPT (64.76% ± 121.70%) than in patients with negative SPT (3.53% ± 23.51%) (p = 0.03). Specific IgE to hazelnut did not differ in OFC+ and OFC- patients, neither the level of recombinant specific IgE to cor a 1, cor a 8, cor a 9 and cor a 14.

**Conclusions**: Unlike the baseline level of serum Tryptase, the relative variation of serum Tryptase during OFC (ΔT/T^b^) is correlated to the clinical results of OFC in children suspected of HA. This ratio could be used to detect subclinical reactions during food challenge or to manage patients with subjective symptoms during OFC to hazelnut.

### PP106 Sublingual immunotherapy for peach allergy regulates the Th2 response by increase of Th1/TReg response

#### Francisca Palomares, Francisca Gomez, Gador Gomez, Maria Jose Rodriguez, Luisa Galindo, Ana Molina, Inmaculada Doña, Esther Barrionuevo, Maria Jose Torres, Cristobalina Mayorga

##### IBIMA-HRUM, Malaga, Spain

**Correspondence**: Francisca Palomares - francis.palomares@gmail.com

*Clinical and Translational Allergy* 2017, **7(Suppl 1)**:PP106

**Introduction**: Pru p 3 is the primary sensitizer of plants fruit and responsible for severe reactions in the Mediterranean area. Sublingual immunotherapy (SLIT) using peach extract enriched in Pru p 3 (Prup3-enriched-SLIT) brings a new perspective to treat patients with severe reactions to peach. However, there is a lack of knowledge regarding immunological response during the immunotherapy.

Our aim was to evaluate the lymphocyte modulation from a Th2 pattern to a Th1/Treg profile during one year of Prup3-enriched-SLIT.

**Methods**: We studied three groups: peach allergic patients who received Prup3-enriched-SLIT for 1 year, peach allergic untreated patients, and healthy controls who tolerated peach. Monocyte-derived dendritic cells (DCs) maturation and lymphocyte proliferation were assessed by flow cytometry from peripheral blood mononuclear cells obtained before treatment, and 1, 6 and 12 months during SLIT, and cultured with Prup3.

**Results**: We found statistically significant differences in DCs activation and maturation between allergic patients and controls at the basal state. When we analyzed the effect of Pru p 3-SLIT at different times, we found a significant reduction of activation and maturation markers (CCR7, CD40, CD80, CD83 and CD86) at the first month of treatment that was maintained after 1 year. Concerning lymphocytes, we observed a significant decrease of effector cells Th2, Th9, NK^Dim^ and IgE producing plasma cells (IgE-PCs) and an increase in Th1, NK^Bright^ and Treg cells subpopulations. These changes were only observed in the Pru p 3-SLIT group. These results were similar to those obtained in the specific proliferative response to Pru p 3 in lymphocyte subpopulations. Most of these changes demonstrated to be significant from the first month of treatment.

Our results showed for the first time the immunological changes induced by Pru p 3-SLIT in peach allergic patients with severe reactions that include significant decreases of Th2/Th9/(IgE-PCs)/NK^Dim^ and increases of Th1/NK^Bright^/Treg cells.

**Conclusions**: Patients treated with Pru p 3-SLIT showed immunological differences compared to untreated patients from a Th2 towards a Th1/Treg response. These changes could be used as biomarkers of therapy evolution during SLIT.

### PP107 Effects of different dietary treatments on epigenetic mechanisms in children with IgE-mediated cow’s milk allergy

#### Lorella Paparo, Rita Nocerino, Maurizio Mennini, Linda Cosenza, Carmen Di Scala, Antonio Amoroso, Rosita Aitoro, Roberto Berni Canani

##### University of Naples, Naples, Italy

**Correspondence**: Rita Nocerino - ritanocerino@alice.it

*Clinical and Translational Allergy* 2017, **7(Suppl 1)**:PP107

**Introduction**: We showed that the acquisition of oral tolerance in children with cow’s milk allergy (CMA) is driven by a different DNA methylation pattern of Th1 and Th2 cytokines. In this study we evaluated comparatively the effect of different dietary treatments on DNA methylation pattern of cytokine genes involved in Th2 (IL-4) and Th1 (IL-10) response in children with IgE-mediated CMA.

**Methods**: Prospective randomized study in subjects with IgE-mediated CMA randomized into two groups of dietary treatment: (1) extensively hydrolyzed casein formula containing the probiotic *L.rhamnosus* GG (LGG); (2) soy formula. At enrollment and after 6 and 12 months of treatment a venous blood sample was collected to perform DNA methylation analysis of the CpGs in the promoter region, and the determination of serum concentrations of IL-4 and IL-10 by ELISA. At 12 months, the possible acquisition of oral tolerance to cow’s milk proteins was evaluated by oral food challenge.

**Results**: 16 children (11 males, ages 6–12 months) were prospectively randomized: 8 in Group 1 and 8 in Group 2. At enrollment DNA methylation rate of CpGs in the promoter region of IL-4 and IL-10 cytokines and respective serum levels were similar between the two groups. After 6 and 12 months of dietary treatment subjects treated with extensively hydrolyzed casein formula containing LGG showed significant higher rate of IL-4 and lower rate of IL-10 gene methylation associated with significant differences in serum levels. In addition, after 12 months of exclusion diet, the number of patients acquiring oral tolerance resulted double in Group 1 (50 vs 25%).

**Conclusions**: Dietary treatment with extensively hydrolyzed casein formula containing LGG induces a more rapid and pronounced epigenetic effect on IL-4 and IL-10 DNA methylation pattern. These epigenetic mechanisms might be involved in the positive effects of this dietary strategy on the acquisition of oral tolerance.

### PP109 The tree nut components IgE profile in patients suspected of food allergy

#### Adam Wawrzeńczyk, Magdalena Żbikowska-Gotz, Michał Przybyszewski, Anna Wawrzeńczyk, Zbigniew Bartuzi

##### Department of Allergology, Clinical Immunology and Internal Diseases, L. Rydygier Collegium Medicum, Bydgoszcz NCU, Bydgoszcz, Poland

**Correspondence**: Adam Wawrzeńczyk - adanw23@gmail.com

*Clinical and Translational Allergy* 2017, **7(Suppl 1)**:PP109

**Introduction**: According to the EuroPrevall (The prevalence, cost and basis of food allergy across Europe) report tree nut tree nut allergy is one of the most common food allergens. It is connected mainly with cross-reactions with tree pollen-based Central Europe and North- Eastern Europe, but also of severe anaphylactic reaction related LTP- Mediterranean Countries. Tree nut allergy include allergy to: Almond, Brazil nut Cashew nut, Hazelnut, Chestnut, Macadamia, Pecan, Pistachio and Walnut. So far, everyday diagnostics of tree nut allergy were skin prick tests and determination of the level of allergen-specific IgE. New possibilities of food allergy diagnostics provides molecular diagnostics, in particular ImmunoCap ISAC. ImmunoCap ISAC is an excellent method of diagnosis of tree nuts it detects up to 8 different antibodies against components of nuts: hazelnut (Cor a 1.0401, Cor a 8, Cor a 9), walnut (Jug r 1, Jug r 2, Jug r 3), cashew nut (Ana o 2) and Brazilian walnut (Ber e 1).

**Methods**: The study was conducted at the Department of Allergology, Clinical Immunology and Internal Medicine in Bydgoszcz. The study was retrospective. 136 adult, living in Poland were classified to study. The ImmunoCap ISAC test was performed to all patients.

**Fig. 7:**
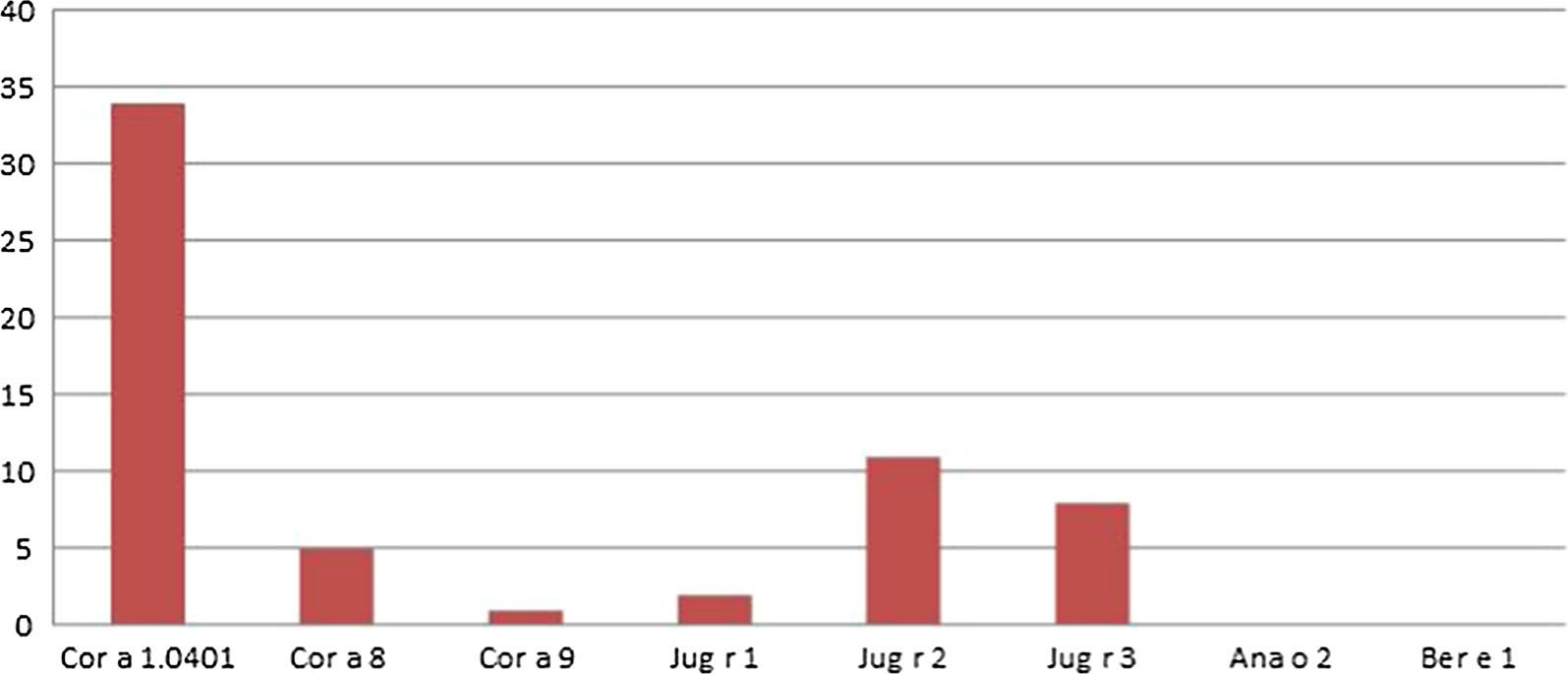
See text for description

**Results**: The antibodies against tree nut components were detected in 1/3 patients. In 81% of patients antibodies against components responsible for cross-reaction with birch pollen were detected. In 37% of patients antibodies against components responsible for anaphylaxis were detected and 78% of detected proteins belonged to walnut. Ana o 2 and Ber e 1 were not detected (Fig. 7).

### PP110 The families’ knowledge about allergic daily problems – Investigation of the mother’s depression and anxiety by a questionnaire

#### Hulya Ercan Sarıcoban^1^, Meltem Ugras^2^, Zerrin Yalvac^3^

##### ^1^Department of Pediatric Allergy and Immunology, Yeditepe University Faculty of Medicine, Istanbul, Turkey; ^2^Department of Pediatric Gastroenterology, Yeditepe University Faculty of Medicine, Istanbul, Turkey; ^3^ Department of Pediatrics, Yeditepe University Faculty of Medicine, Istanbul, Turkey

**Correspondence**: Hulya Ercan Sarıcoban - hulya_ercan@yahoo.com

*Clinical and Translational Allergy* 2017, **7(Suppl 1)**:PP110

**Introduction**: We aimed to search; the awareness of the families about food allergy; the families knowledge about allergic life threatening events; the daily problems the families are experiencing; and to investigate the mother’s depression and anxiety by a questionnaire.

**Methods**: This study is done between 2014 and 2016 in Yeditepe University Medical Faculty Departments of Pediatric Allergy and Pediatric Gastroenterology, to the children who have been following for food allergy and who were still on diet to the allergen food by evaluating the data from the patient files retrospectively. Among 51 patients with allergy 51 mothers fulfilled STAI-1 and STAI-2 questionnaries and Beck’s depression scale. There were 51 children with food allergy, their mothers fulfilled the questionnaire. There were 51 children in the control group and their mother also fulfilled the questionnaire.

**Results**: 51 mothers of those 114 children fulfilled the questionnaire and among those 51 mothers 21 children (41.2%) were girls and 30 (58.2%) were boys. STAI 1 and 2 anxiety points were higher among 51 mothers having children with food allergy when compared to the 51 mothers having children without any food allergy. Mothers with children with food allergy who fulfilled Becks depression scale had higher points than control group. Professional carrier of 30%, social life 39% and economic status of 45.1% was affected among mothers having anxiety. 37.2% of mothers with anxiety had difficulty attending family activitiesand 23.5% had problems with her partner because of the child’s health problems. 45.1% of mothers having either anxiety or depression could not send her child to school/kindergarten.

**Conclusions**: Mothers having children with food allergy experience daily life difficulties, have concerns while sending their children to school, picnic or outdoor. Those mothers are more anxious and depressed and their social life and professions are negatively affected.

### PP111 Determination of peanut-specific health-related quality of life (HRQL) items in the food allergy quality of life questionnaire (FAQLQ)

#### Bertine M. J. Flokstra - de Blok^1^, J. L. van der Velde^1^, Andrea Vereda^2^, Anthony E. J. Dubois^1^

##### ^1^UMCG, Groningen, the Netherlands; ^2^Fundazion Dimenez Diaz, Madrid, Spain

**Correspondence**: Bertine M.J. Flokstra - de Blok - b.m.j.flokstra@umcg.nl

*Clinical and Translational Allergy* 2017, **7(Suppl 1)**:PP111

**Introduction**: The food allergy health related quality of life questionnaires (FAQLQs) were developed and validated to measure the impact of food allergy on health-related quality of life (HRQL) irrespective of the causative allergenic food. At present it is unknown whether all items of these instruments are relevant for peanut allergic patients. The aim of this study was to identify whether there are peanut-specific HRQL items in the FAQLQ-Child Form (CF), Teenager Form (TF) and Adult Form (AF).

**Methods**: In order to identify FAQLQ items that are reported most frequently and have the highest impact for peanut allergic patients, we used previous data from the development of the FAQLQs. We calculated frequency, percentage, mean importance and overall importance of individual FAQLQ items in peanut allergic patients and non-peanut allergic patients. In order to identify peanut-specific FAQLQ items that are sensitive to change, the data of the longitudinal validation of the FAQLQs (HRQL following a double blind placebo controlled food challenge) were used in a comparison of peanut allergic and non-peanut allergic patients and tested for significance (p < 0.05).

**Results**: In general, peanut allergic patients reported a higher overall impact for each item than non-peanut allergic patients. There was 1 item in each FAQLQ that significantly changed following DBPCFC in the non-peanut allergic group compared to the peanut allergic children (“Don’t get anything when someone is giving treats at school”, −1.06 vs 0.04, p = 0.044), teenagers (“Being careful about touching certain foods”, 0.82 vs −0.59, p = 0.016) and adults (“Able to eat fewer products”, −1.00 vs 0.30, p = 0.011). Since peanut allergic patients reported higher overall impact scores and only one item differed between non peanut allergic patients and peanut allergic patients for each FAQLQ, this study did not result in peanut-specific FAQLQ instruments.

**Conclusions**: All parts of the FAQLQ-CF, -TF and –AF are considered to be relevant for peanut allergic patients.

### PP112 “Allergen Hunt”: an educational experience for primary school children in Turin, Italy

#### Clara Ippolito, Amaranta Traversa, Daniela Adriano, Daniela Manila Bianchi, Silvia Gallina, Lucia Decastelli

##### Istituto Zooprofilattico Sperimentale Piemonte, Liguria e Valle d’Aosta – SC Controllo Alimenti, Turin, Italy

**Correspondence**: Clara Ippolito - clara.ippolito@izsto.it

*Clinical and Translational Allergy* 2017, **7(Suppl 1)**:PP112

**Introduction**: Around 8% of children suffer from food allergy, worldwide. Children who become aware about the good food safety practices via practical experiences are more likely to perform long-term protective behaviours and may disseminate knowledge to families.

Here we describe our workshops aimed to engage primary schools to join an educational experience about allergenic food. The activities were included in the “EXPO 2015 events” host by the municipality of Turin.

**Methods**: We designed the “Allergen Hunt” Workshop as follows:

- Welcome telling “The fairy tale about Sabrina” who discovers to be allergic to hazelnut and learns how to manage her allergy.
- Presentation of the main allergenic foods.
- “Guess the allergen here inside”- children have to select the right allergen-card that can be hidden in some proposed foods.
- “Special cookies”- children are divided in two groups: group A cooks hazelnut cookies (using play dough and green glitter powder as hazelnut flour) and group B cooks coconut cookies (using play dough and gold glitter powder as coconut flour); then few cooks of group A go to help group B and *vice versa*.
- Dissemination of rules on behaviour to prevent cross-contaminations.

**Results**: From 11th to 15th May 2015, ten primary schools (216 children) joined our workshops: 29.6% of the first class (age: 6 y-o), 40.3% of the second (7 y-o), 18.5% of the third (8 y-o), and 11.6% of the fifth class (10 y-o). All of them knew the word “allergy” for environmental allergens, and many of them had already heard about milk, peach and hazelnut as food allergens. We presented the most frequent food allergens (milk, eggs, wheat, nuts, soybeans, crustaceans, molluscs, fish) among the ones reported by EU Regulation 1169/2011; we chose to add to the list peach, kiwifruit, and sesame seeds because they are usually eaten by children and may cross-reacted with environmental allergens. Younger children showed great interest in the “Special cookies” handling game. “Guess the allergen here inside” did unexpectedly excite older and younger children: most of them answered correctly and were aware about the presence of milk in ham and eggs in hamburgers.

**Conclusions**: Children were interested in learning more about food allergens and in joining the practical experiences we proposed. Participants were often competent, sensitive to food allergy topic and interested to know the rules to manage food allergy and to reduce contamination during food handling and preparation.

### PP113 Psychological impact of open food challenges in adults

#### Melina Makatsori, Anne Miles

##### Department of Psychological Sciences, Birkbeck, University of London, London, United Kingdom

**Correspondence**: Melina Makatsori - makatsorim@yahoo.co.uk

*Clinical and Translational Allergy* 2017, **7(Suppl 1)**:PP113

**Introduction**: Despite food challenge testing being the gold standard for food allergy diagnosis, there is only minimal research published on its psychological impact and effect on health related quality of life (HRQoL) in adults. The aims of this study were to assess whether undergoing a clinically indicated open food challenge affects the quality of life of adults with suspected food allergy and whether HRQoL gains are higher among people testing negative than those testing positive for food allergy. In addition, we aimed to evaluate whether undergoing a food challenge test leads to a reduction in anxiety.

**Methods**: A prospective cohort study involving completion of a survey prior to the challenge test, on the day, and three months after the test was conducted. The inclusion criteria were: age 18 or older and a clinical decision that a food challenge was indicated. The Food Allergy Quality of Life Questionnaire-Adult Form (FAQLQ-AF) was used to assess change in HRQoL before and after the challenge. State anxiety was assessed with the 6-item version of the Spielberger State Trait Anxiety Inventory.

**Results**: 53 individuals were included. The mean age was 33.5 years (SD = 12.5). The majority were female (71.7%). A variety of foods were tested with the most common being tree nuts, peanut, shellfish and fish. Most participants tested negative on challenge (84.9%). There was a significant improvement in HRQoL three months post-challenge compared to baseline, F (1,52) = 15.346; p < 0.001 and this was unrelated to challenge outcome. A significant reduction in state anxiety was observed following the food challenge, F (2,104) = 15.75; p < 0.001. Undergoing a food challenge test leads to improvement in HRQoL of adults with suspected food allergy despite whether they test positive or negative.

**Conclusions**: By making these tests more widely available in clinical practice and clarifying whether an individual is allergic or not, any uncertainty can be dispelled, unnecessary food restrictions can be avoided, HRQoL can be improved and anxiety reduced.

### PP114 Evaluation of the Anaphylaxis Post-graduate Teacher Intervention Programme

#### Sonja Posega Devetak^1^, Iztok Devetak^2^, Tina Vesel^3^

##### ^1^Department of Pediatrics, General and Teaching Hospital Izola, Izola, Slovenia; ^2^Faculty of Education, University of Ljubljana, Ljubljana, Slovenia; ^3^Department of Allergology, Rheumatology and Clinical Immunology, University Medical Center, University Children’s Hospital, Ljubljana, Slovenia

**Correspondence**: Sonja Posega Devetak - sonja.posega@gmail.com

*Clinical and Translational Allergy* 2017, **7(Suppl 1)**:PP114

**Introduction**: Studies showed that teachers’ competences for managing children at risk of anaphylaxis or children with food allergy in kindergartens and schools are poorly developed. The purpose of this study was to explore how short theoretical and practical intervention programme influence on pre-service teachers’ knowledge about allergy and anaphylaxis.

**Methods**: 62 post-graduate pre-service primary and lower secondary school teachers (all female; median age 24.5) participated in the study. 27.4% of pre-service teachers reported that they are allergic themselves. Participants were exposed to 90 min theoretical (about allergy and anaphylaxis) and practical (using adrenalin auto-injector) educational intervention. Participants answered the Teachers’ Health Competences Development–Anaphylaxis Management Questionnaire (THCDAMQ) which comprised knowledge items about anaphylaxis (Max 7 points) and attitude items on managing children’s anaphylaxis three times, before intervention, immediately after and 14 days after the intervention.

**Results**: Pre-service teachers showed positive attitudes towards learning more about different children’s health issues (91.9%). All of them expressed that child health topics were very important for each teacher and all wanted to increase their health competences. 90.3% thought that teacher is responsible for pupils’ health issues during school time. 71% reported that they haven’t been exposed to any activities that would promote their health competences development. The results of the Friedman Test indicated that there was a statistically significant difference in THCDAMQ scores across the three time points (pre-intervention, post-intervention, 14-days follow-up χ^2^ (2, *N* = 37) = 48.127, *p* ≤ .000). Inspection of the median values showed an increase in total scores on items that test post-graduate pre-service teachers’ knowledge about anaphylaxis from pre-intervention (*Md* = 3; IQR 2–4.5) to post-intervention (*Md* = 6; IQR 6–6) and a follow-up (*Md* = 6; IQR 6–6) scores.

**Conclusions**: Intervention anaphylaxis programme had positive effect on students’ knowledge and attitudes towards school child allergy. Students retained their knowledge after intervention. Pre-service teachers would manage a child with anaphylaxis more efficiently when intervention anaphylaxis programme is available to them.

### PP115 N-3, N-6 polyunsatured fatty acids effect on the local anaphylactic response and intestinal integrity in a murine model of allergy

#### Abir Haddi, Malika Guendouz, Soraya Ainad Tabet, Youcef Bouferkas, Omar Kheroua, Djamel Saidi

##### LPNSA, Biology, Faculty of Natural and Life Sciences, University of Oran 1 Ahmed Ben Bella, Oran, Algeria

**Correspondence**: Abir Haddi - abira85@yahoo.fr

*Clinical and Translational Allergy* 2017, **7(Suppl 1)**:PP115

**Introduction**: This study was carried out in order to investigate a possible preventive n-3 and n-6 PUFA effect against alterations due to immunization with β-lactoglobulin (β-Lg) in Balb/c mice.

**Methods**: Four to five weeks old female Balb/c were divided into 3 groups (n = 9 each). Mice were supplemented by gavage at a dose of 0.6% V/W for 15 consecutive days with either: (1) fish oil, rich in PUFA n-3, (2) corn oil, rich in PUFA n-6 and slight amount of PUFA n-3 or (3) a solution of PBS 1/10 as the control group. Mice of all groups were then immunized with intraperitoneal injections of 10 µg β-Lg absorbed onto 2 mg of Al(OH)_3_. At the end of the experimentation, mice were killed and jejunums were removed for histological study and local anaphylactic study in Ussing chamber by the measurement of the electrophysiological parameters: short current circuit Isc (µA/cm^2^) and tissue conductance G (mmho/cm^2^).

**Results**: In Ussing chamber stimulation with β-Lg of jejunal fragments of the control group induced an increase in the Isc and G values (ΔIsc = 17.40 ± 2.39 µA/cm^2^, ΔG = 6.41 ± 1.67 mmho/cm^2^). This increase is very significantly diminished after n-3 PUFA supplementation (ΔIsc = 7.94 ± 1.46 µA/cm^2^, ΔG = 1.81 ± 0.62 mmho/cm^2^ p < 0.01). A non significant decrease of the Isc and G values (ΔIsc = 10.73 ± 1.55 µA/cm^2^, ΔG = 3.65 ± 0.62 mmho/cm^2^) was observed in the group supplemented with n-6 PUFA compared to the control group. The altered intestinal architecture observed in the control group was improved in the groups supplemented with both PUFA n-3 and n-6. The beneficial effect exerted by (n-3, n-6) PUFA was evidenced by a very significant increase (p < 0.01) of the villis length.

**Conclusions**: Local intestinal anaphylactic response and intestinal damages are some of IgE mediated food allergy symptoms. Although contradictory, studies showed a decrease in sera IgE levels after supplementation by fish oil (n-3) or sunflower oil (n-6). In our experiment, n-3 PUFA seems to reduce the intestinal anaphylactic response and both n-3 and n-6 PUFA improved the intestinal architecture. The mechanism by which these PUFA exert their effect cannot be explained by this study and need to be further investigated. PUFA supplementation especially n-3 PUFA decreases considerably some of the damages resulting from sensitization with β-Lg in Balb/c mice and could be considered as a preventive strategy against allergy to cow’s milk proteins.

### PP116 Effect of taurine on modulation of intestinal anaphylactic response in β-Lactoglobulin sensitised Balb/c mice

#### Soraya Ainad Tabet, Abir Haddi, Omar Kheroua, Djamel Saidi

##### LPNSA, Biology, Faculty of Natural and Life Sciences, University of Oran 1 Ahmed Ben Bella, Oran, Algeria

**Correspondence**: Soraya Ainad Tabet - haddisoraya@yahoo.fr

*Clinical and Translational Allergy* 2017, **7(Suppl 1)**:PP116

**Introduction**: This study was led in order to evaluate taurine effect on modulation of intestinal anaphylactic response induced by beta-lactoglobulin (β-Lg), major allergen of bovine milk in Balb/c mice used as an animal model of allergy to cow’s milk proteins (ACMP).

**Methods**: 42 Balb/c female mice were divided into 3 groups (n = 14 each). The first group was untreated and represents the negative control (CL−), the second group was intraperitoneally sensitized with β-Lg and represents the positive control (CL+), the third group received for 15 days 100 mg/kg/day of taurine administrated intraperitoneally (Tau+) then sensitized intraperitoneally with β-Lg. Specific IgG anti-β-Lg were determined in the mice sera by an enzyme-linked immunosorbent assay (ELISA), and local anaphylactic responses were performed ex-vivo in Ussing chamber by intestine challenge with β-Lg. All animals were subjected to the challenge test in-vivo by i.p. injection (1 mg/mouse) of β-Lg.

**Results**: Compared with sensitized mice, those treated with taurine have lower anti-β-Lg-specific IgG (1/16,750 th) (p < 0.0001).The addition of β-Lg to the serosal side of the mouse intestinal epithelium in Ussing chamber produced electrogenic chloride (Cl-) secretion as shown by Isc stimulation (ΔIsc = 7.50 ± 3.34 µA/cm2) (ΔIsc = 17.54 ± 1.04 µA/cm^2^) respectively (p < 0.001). Sensitized mice demonstrated also an enhanced epithelial permeability as measured by conductance (p < 0.0001). Taurine treatment resulted in a significant decrease in secretory response. Signs of allergic reaction were less important in (Tau+) group, but it should be noted that 50% of the mice reached stade 4 after 30 min.

**Conclusions**: A net increase in sera IgG anti-β-Lg titers was observed in (CL+) indicating a high immunogenicity of this protein. Also, a low production of IgG anti-β-Lg was observed in the (Tau+) suggesting that taurine plays an immunomodulation role. The stimulation with β-Lg in the mucosal side of tissue in the Ussing chamber was significantly decrease tissues of (Tau+) compared with (CL+), suggesting a decrease in local intestinal anaphylactic response. These findings provide evidence for the first time suggesting that taurine appears to reduce intestinal anaphylactic response, indicating protection from β-Lg-induced epithelial permeability increase.

### PP117 Anaphylaxis - In situ simulation training in an out-patient allergy clinic

#### Jeanette Fisker Trandbohus^1^, Pernille Winther^2^, Hans-Jørgen Malling^2^, Kirsten Skamstrup Hansen^1,2^, Lene Heise Garvey^1^

##### ^1^Department of Pediatrics, Herlev and Gentofte Hospital, Copenhagen University Hospital, Copenhagen, Denmark; ^2^Allergy Clinic, Herlev and Gentofte Hospital, Copenhagen University Hospital, Copenhagen, Denmark

**Correspondence**: Jeanette Fisker Trandbohus - jeanette.fisker.trandbohus@regionh.dk

*Clinical and Translational Allergy* 2017, **7(Suppl 1)**:PP117

**Introduction**: Anaphylaxis is a medical emergency, requiring rapid recognition and treatment. Simulation team-training is an effective method in other emergencies such as cardiac arrest. The Allergy Clinic and Department of Pediatrics evaluate and treat children and adults with allergic diseases. Procedures with a risk of inducing anaphylaxis e.g. allergen challenge tests and immunotherapy are performed daily. Recently, staff members have undergone *in situ* simulation team-training with focus on diagnosis and treatment of anaphylaxis. Training took place in the departments in collaboration with the Simulation Unit. The aim of this investigation was to evaluate the attitude to *in situ* simulation team-training among staff.

**Methods**: Participants answered a questionnaire 1–3 months after training.

*Questions:*

1. Has the *in situ* training increased your focus on closed-loop communication in emergencies?
2. Has the *in situ* training increased your focus on the importance of team work when treating anaphylaxis?
3. Has the *in situ* training increased your focus on the use of the ABCD-algorithm when treating anaphylaxis?
4. Has the *in situ* training increased your knowledge of the anaphylaxis treatment algorithm?
5. Has your knowledge regarding treatment of emergencies in general increased after training?
6. Did training in your own department add to the learning experience?
7. How confident do you feel in treating anaphylaxis after the in situ training?

Possible answers question 1–6: □ Yes, definitely □ Yes, partly □ I don’t know/neutral □ No, I doubt it □ No, definitely not

Possible answers question 7: □ Very confident □ Confident □ Almost confident □ Not confident □ Very unconfident

**Results**: In all, 35 staff members completed training and 23 (66%) answered the questionnaire. Respectively 96, 100, 83, 82, 86% answered “Yes, definitely” or “Yes, partly” to question 1–5. Twenty-two of 23 stated, that training within the department had added to the learning experience. One did not answer. After *in situ* training, 96% were ’Very confident’ or ’Confident’ in treating patients with anaphylaxis.

**Conclusions**: *In situ* simulation team-training was perceived to add to the learning experience and highlight the importance of team work and awareness of managing emergencies locally within the departments. These preliminary data support the value of *in situ* simulation training in departments performing procedures and treatments with a high risk of inducing anaphylaxis.

### PP118 Di-(2-ethylhexyl)phthalate disturbed food allergic responses in ovalbumin-sensitized mice

#### Chia-Chi Wang^1^, Yin-Hua Cheng^1^, Chun-Wei Tung^1^, Tong-Rong Jan^2^

##### ^1^Kaohsiung Medical University, Kaohsiung, Taiwan; ^2^National Taiwan University, Taipei, Taiwan

**Correspondence**: Chia-Chi Wang - chiachiwang@kmu.edu.tw

*Clinical and Translational Allergy* 2017, **7(Suppl 1)**:PP118

**Introduction**: Food safety is threatened by environmental toxicants which were contaminated from industrial processes. Di-(2-ethylhexyl) phthalate (DEHP) is among the most-produced phthalates used as plasticizer in industry to increase the flexibility of polyvinyl chloride (PVC) products. DEHP was intentionally blended into emulsifier in Taiwan and has been ubiquitously detected in numerous drinks and food. The incident of phthalate-contaminated foodstuffs raises the concerns of exposure of DEHP-tainted foods on public health. Epidemiological studies revealed a positive association between DEHP exposure and the development of asthma, allergies and atopic disorders. Although several toxicology studies indicate the adjuvant effects of DEHP to disturb the T cell functionality systemically or in the lung tissues of allergic animals, however, little is known regarding the effects of DEHP on intestinal immunity and its underlying mechanisms. As the primary source of DEHP exposure for most people is through gastrointestinal tract and one of the major health concerns of DEHP is its deteriorated effects on immune system, the objective of this study is to evaluate the effects of DEHP on intestinal immunity.

**Methods**: The allergen-induced diarrhea mice model was applied to study the adjuvant effects of DEHP on the incidence of allergic symptoms and pathological changes.

**Results**: The present data demonstrated that exposure of DEHP dose-dependently induced the severity of OVA-induced allergic responses. Several aspects were affected by DEHP including acceleration of the occurrence of OVA-induced diarrhea, increased serum level of OVA-specific IgE, infiltration of IL-4-secreting T cells in the villus and the degranulated mast cells with the crypt of duodenum.

**Conclusion**: Collectively, exposure of DEHP will exacerbate antigen-specific allergic responses in the food allergic BALB/c model.

### PP119 Identification of immunoreactive proteins derived from different strains of *Lactobacillus acidophilus*

#### Anna Szyc, Lidia Markiewicz, Agata Szymkiewicz, Mariola Dietrich, Barbara Wróblewska

##### Institute of Animal Reproduction and Food Research of Polish Academy of Sciences, Olsztyn, Poland

**Correspondence**: Anna Szyc - a.szyc@pan.olsztyn.pl

*Clinical and Translational Allergy* 2017, **7(Suppl 1)**:PP119

**Introduction**: The main aim of the study was comparison and identification of the Lactobacillus acidophilus derived proteins which have the capacity to bind to humans class E antibodies.

**Methods**: To detect the immunoreactive bacterial proteins of Lactobacillus acidophilus 145 and Lactobacillus acidophilus La5 the individual protein fractions were isolated from MRS (18 h/37 °C) bacterial cultures. Bacterial protein fractionation was performed as in Bäuerl (2010). The Lactobacillus acidophilus cell fractionation of proteins have been subjected separation with SDS-PAGE and 2DE method followed by immunoblotting with human sera with determined increased IgE level. Identification of obtained spots was carried out by MALDI-TOF MS/MS method.

**Results**: Obtained results indicate that lactic acid bacteria commonly used in the production technology of fermented milk beverages also have in their structure a protein capable of binding to the antibodies of class E. The profile of isolated bacterial proteins differs considerably depending on analyzed strain. It has been proven that some bacterial proteins connected with surface layer are prone to bind nonspecifically to human IgE but this part was excluded using specific anti-A and anti-G antibodies. The comparison of IgE-immunoreactive proteins profile show difference between the strains and between used human serum. Identification of this proteins revealed that they belong mainly to enzymatic group and are located both in cytoplasmic and membrane fraction of proteins.

**Conclusions**: Immune system in the initial stage of life in most organisms develops tolerance and identifies commensal microbial protein as self-consistent. On the other hand bacterial cells are a reach source of proteins that can exhibit immunomodulatory properties. It is known that bacterial proteins have determinants that are recognized by human immunoglobulins A, M and G but also E. Part of IgE reaction can be nonspecific like this induced by S-layer proteins (A and G) but there are also enzymatic proteins with proven immunoreactive potential. These results indicate that bacterial proteins depending on the strain and localization have varied profile and reveal different determinants with different affinity to human IgE antibodies.

### PP120 The impact of filaggrin mutations in a large pediatric population with food allergy

#### Ingo Marenholz^1,2†^, Birgit Kalb^1,2,3†^, Sarah Grosche^1,2^, Katharina Blümchen^4^, Rupert Schlags^5^, Mareike Price^6^, Sylke Rietz^7^, Jorge Esparza-Gordillo^1,2^, Thomas Keil^8,9^, Susanne Lau^3^, Bodo Niggemann^3^, Kirsten Beyer^3^, Young-Ae Lee^1,2^

##### ^1^Max-Delbrück-Center (MDC) for Molecular Medicine, Berlin, Germany; ^2^Clinic for Pediatric Allergy, Experimental and Clinical Research Center, Charité - Universitätsmedizin Berlin, Berlin, Germany; ^3^Department of Pediatric Pneumology and Immunology, Charité - Universitätsmedizin Berlin, Berlin, Germany; ^4^Department of Allergy, Pulmonology and Cystic Fibrosis, Children’s Hospital, Goethe University, Frankfurt am Main, Germany; ^5^Children’s Hospital, Wangen, Germany; ^6^Clinic for Pediatric Pneumology, Allergology and Neonatology, University Children’s Hospital Hannover, Hannover, Germany; ^7^Center for Pediatric and Adolescent Medicine, Elisabeth Children’s Hospital, Oldenburg, Germany; ^8^Institute of Social Medicine, Epidemiology and Health Economics, Charité - Universitätsmedizin Berlin, Berlin, Germany; ^9^Institute for Clinical Epidemiology and Biometry, University of Würzburg, Würzburg, Germany

^†^Ingo Marenholz and Birgit Kalb equally contributed to this work

**Correspondence**: Ingo Marenholz - i.marenholz@mdc-berlin.de

*Clinical and Translational Allergy* 2017, **7(Suppl 1)**:PP120

**Introduction**: Loss‐of‐function‐mutations in the epidermal barrier gene filaggrin (*FLG*) are the major genetic risk factor for eczema and eczema‐associated allergic airways diseases. We aimed to investigate the role of *FLG* mutations in food allergy, the importance of which is still unclear.

**Methods**: We have recruited 523 children with food allergy diagnosed by double‐blind placebo controlled food challenge. All children were extensively characterized including allergic responses to specific foods. The four most common *FLG* loss‐of‐function mutations were genotyped in all cases and controls. Association of *FLG* mutations with food allergy was analyzed by logistic regression using the Multicenter Allergy Study (MAS) as control population.

**Results**: *FLG* mutations were associated with allergies to diverse foods (hen’s egg, peanut, cow’s milk) with similar risk estimates (odds ratios between 2.5 and 2.7, P‐values <10^−5^). Interestingly, this effect remained significant after adjusting for the eczema status. We did not observe an association of *FLG* mutations with polyvalent compared to monovalent food allergy. Mucocutaneous, gastrointestinal, respiratory, and cardiovascular responses during double‐blind placebo controlled food challenge were documented in a standardized fashion. The *FLG* effect increased with the number of organ systems involved suggesting an effect on severity of the allergic response.

**Conclusions**: Since *FLG* mutations cause a skin barrier defect, our results suggest a pathway for food allergy similar to that for eczema‐associated asthma, where transcutaneous sensitization through the impaired epidermal barrier is the initializing event leading to localized or systemic allergic responses that may affect distant organ systems. Co‐occurrence of food allergy and eczema in early childhood is a common phenomenon. Using our large, well characterized food allergy cohort we demonstrated that *FLG* mutations confer risk for food allergy beyond their known association with eczema. They predispose to multi‐organ involvement during allergic reactions, and should thus be considered when assessing anaphylaxis risk.

## POSTER SESSION 4: Food allergens

### PP121 Epidemiology of food allergy and associated atopic disorders in Saudi Arabia (single referral center experience)

#### Ali Almontasheri^1^, Mohammad Al Bahkali^1^, Sahar Elshorbagi^2^, Abdullah Alfhaid^2^, Mashary Altamimi^2^, Eman Madbouly^2^, Hassan Al-Dhekri^3^, Rand K. Arnaout^1^

##### ^1^Department of Adult Allergy and Clinical Immunology, King Faisal Specialist Hospital and Research Centre, Riyadh, Saudi Arabia; ^2^King Faisal Research Center, King Faisal Specialist Hospital and Research Centre, Riyadh, Saudi Arabia; ^3^Department of Pediatric Allergy and Clinical Immunology, King Faisal Specialist Hospital and Research Centre, Riyadh, Saudi Arabia

**Correspondence**: Ali Almontasheri - dr.ali-999@hotmail.com

*Clinical and Translational Allergy* 2017, **7(Suppl 1)**:PP121

**Introduction**: Food allergy affect up to 8% of children and 3% of adult in the United State and several studies have noted racial discrepancies in prevalence. There is great lack of data about epidemiology of food allergy and associated atopic disorder in Saudi Arabia.

Our objective is to explore Epidemiology of food allergy and associated atopic disorder in Saudi Arabia.

**Methods**: This is a retrospective study that is still in progress. We collected so far 128 patients evaluated for presumed food allergy in eight allergy clinics out of 1360 patient seen in the clinics in one referral Center in Riyadh/Saudi Arabia, between 1^st^ January and 30 April, 2016. We assessed patient’s demographic data, offending food allergens, presenting manifestation, associated atopic disorder, diagnostic measures and treatment plans.

**Results**: The study included 103 patients out of 128 who had complete data, on their clinical symptoms and objective diagnostic measures of food allergy (RAST, ASPT, oral challenge). The pediatric patients were (79%) of the total number of patients, Male (63%) of all patients. Prevalence of Food allergy among adult (>14yrs) patients was 1.5%, while among pediatric patients 6%. Overall the most common offending food allergens were Egg (40%), sesame (39.8%), Tree nuts (39.7%), Milk(28%), Peanut (24%), Fish (21%) Wheat (13%), Shell fish (9%), Soy (7%), Legume (6%). Regarding the presenting manifestation Mucocutaneous were the most common symptoms affecting (43%) of patients followed by anaphylaxis in (32%), pulmonary in (13%) and Gastro-intestinal (6%). Overall (60%) of patients with food allergy had other associated atopic disorder namely bronchial asthma, Allergic rhinitis and atopic dermatitis.

**Conclusions**: This is the very first study exploring the epidemiology and Prevalence of food allergy among Saudi patients who visited allergy clinics in a referral centerPreliminary results revealed the food allergy affects 1.5% of adults and 6% pediatric population Egg followed by sesame, nuts, milk, peanut are the more common offending food allergens in Saudi Arabia. Most presenting feature are mucocutanous followed by anaphylaxis. Majority of the cases associated with other atopic disorder.This study is still in progress, and data will be collected from other centers around the country to have a more accurateand complete picture.

### PP123 Flax seed allergy

#### Maria Basagaña^1^, Sira Miquel^1^, Borja Bartolomé^2^

##### ^1^Allergy Unit, Hospital Universitari Germans Trias i Pujol, Universitat Autònoma de Barcelona, Badalona, Spain; ^2^ Bial-Aristegui Laboratories, Bilbao, Spain

**Correspondence**: Maria Basagaña - maria.basagana.torrento@gmail.com

*Clinical and Translational Allergy* 2017, **7(Suppl 1)**:PP123

**Introduction**: We report the case of a 46 year-old woman, with personal history of bronchial asthma and rhino conjunctivitis, who attended our Unit in September 2012 to study an anaphylaxis appeared immediately after eating multicereals biscuits (oat, rice, flax seeds, egg, almonds and raisins).

**Case report**: Skin-prick tests (SPT) were positive for dog dander exclusively and negative for house dust mites, fungi, cat and dog epithelia, latex and pollen (*Olea europea*, *Parietaria Judaica, Cupressus arizonica, Platanus acerifolia, Graminae family, Artemisia vulgaris*); SPT with food allergens were positive to nut, peanut, mustard, tomato, corn, peach and apple and SPT with cereals commercial extracts (corn, wheat, rice, oat, barley, rye, gluten and gliadin) were negative. Prick by prick with golden and toasted linseed was positive. Total IgE (UniCAP, Thermofisher) was 282 kU/L, specific IgE to dog dander 3.26 kU/L, peach 1.63 kU/L, wheat 0.54 kU/L, rye 0.38 kU/L, apple 1.14 kU/L, Pru p 3 1.97 kU/L, omega-5 gliadin 0- kU/L, tomato 2.22 KU/l. The microarray ISAC (Termofisher) showed polysensitization to both cross-reactivity and species-specific allergens: Can f 5 35 ISU, Cry j 1 0.4 ISU, Cup a 1 1.8 ISU, Ara h 9 0.3 ISU, Jug r 3 0.4 ISU, Pru p 3 0.4 ISU, Art v 3 0.5 ISU, other determinations were negative. Sodium dodecyl sulfate polyacrylamide gel electrophoresis (SDS-PAGE) immunoblotting was performed with gold and toasted flax seed extract. IgE immunoblotting with the patient’s serum, under reducing conditions, showed an IgE binding band < 14 kDa both in the gold flax seed and in the toasted linseed extract. We present a case of a patient with anaphylaxis due to flax seed allergy with a LTP*s* sensitization and asthma and rhinitis due to dog dander allergy with monosensitization to kallicrein. The patient remains asymptomatic with a free diet without flaxseed.

**Conclusion**: The prevalence of allergy to seeds is highly influenced by the geographical area and eating habits. In Spain, according to Alergologica 2005, seeds allergy represents 1.6% of food allergy. The cases reported of flax seed allergy in pubmed are anecdotal but linseed is recognized as an increasingly important food allergen since its introduction in diet for its nutritional benefits and its use as a laxative. To date, no flaxseed allergen has been characterized yet.

**Consent to publish**: The patient has consented to publication of our findings.

### PP125 Value of specific IgE against storage proteins for the diagnosis of tree nut and peanut allergy

#### Bettina Brix^1^, Sabine Dölle^2^, Stefanie Rohwer^1^, Sandra Brandhoff^1^, Alena Berger^1^, Waltraud Suer^1^, Alf Weimann^1^, Margitta Worm^2^

##### ^1^EUROIMMUN AG, Lübeck, Germany; ^2^Department of Dermatology and Allergology, Comprehensive Allergy Center Charité, Charité Universitätsmedizin Berlin, Berlin, Germany

**Correspondence**: Margitta Worm - margitta.worm@charite.de

*Clinical and Translational Allergy* 2017, **7(Suppl 1)**:PP125

**Introduction**: Double blind placebo controlled food challenge (DBPCFC) is the gold standard in food allergy diagnosis, reflecting the clinical outcome of food allergic patients. Studies have shown that component resolved specific IgE (sIgE) testing can aid in the diagnosis of food allergies by the detection of sIgE against distinct proteins. Here, we investigated the advantage of component based multiplex sIgE testing in diagnosing subjects with suspected tree nut and peanut allergy.

**Methods**: Patients from Germany with suspected tree nut and/or peanut allergy were included. Four clinical symptom groups based on a clinical questionnaire, skin prick testing (SPT), occasionally sIgE measurement and DBPCFCs were defined and referred as no symptoms, no symptoms/tolerant, oral allergy syndrome (OAS), and grade I-IV anaphylaxis (ANA). Subsequently sera from all subjects were investigated for sIgE using EUROLINE sIgE multiparameter tests (EUROIMMUN, Luebeck, Germany) with extracts and components of distinct tree nuts (hazelnut, walnut, macadamia nut, cashew nut, pecan nut, Brazil nut, and pistachio) and peanut.

**Results**: SPT indicated sensitization to tree nut and/or peanut, and pollen in 68 subjects. 41% and 59% of these subjects presented with OAS and ANA, respectively. As determined by EUROLINE tests, subjects from both symptom groups were sensitized to PR-10 proteins from hazelnut (Cor a 1: OAS 89%; ANA 95%), almond (Pru du 1: OAS 36%; ANA 43%), and peanut (Ara h 8: OAS 18%; ANA 23%). The sensitization to storage proteins from tree nuts and peanut was almost exclusively shown in anaphylactic patients, 33% of which were positive for sIgE against storage proteins from more than one tree nut and/or peanut. Moreover, among anaphylactic patients, sIgE against Ara h 6 was most frequently (38%) detected.

**Conclusions**: In this cohort, sensitization to storage proteins from tree nut and/or peanut was highly associated with ANA, indicating that the detection of sIgE against storage proteins can improve the assessment of clinical outcome in allergic patients. In contrast, anti-PR-10 IgE gave no indication on clinical outcome since the majority of subjects with tree nut and/or peanut allergy was sensitized to PR-10 protein due to cross-reactivity to birch pollen. Component based multiplex sIgE tests including storage proteins can aid in assessing the risk of ANA that provides additional value for the diagnosis of serious allergic reactions in tree nut and peanut allergy.

### PP126 Isolation and characterisation of 2S albumins: developing clinical tools for allergy diagnosis

#### Cristina Bueno^1^, Laura Martín-Pedraza^1^, Sara Abián^1^, Pablo San Segundo-Acosta^1^, Juan Carlos López-Rodríguez^1^, Rodrigo Barderas^1^, Eva Batanero^1^, Javier Cuesta-Herranz^2^, María Teresa Villalba^1^

##### ^1^Departamento de Bioquímica y Biología Molecular, Facultad de Ciencias Químicas, Universidad Complutense de Madrid, Madrid, Spain; ^2^Departamento de Alergología, Fundación Jiménez Díaz, Madrid, Spain

**Correspondence**: Cristina Bueno - crbueno@ucm.es

*Clinical and Translational Allergy* 2017, **7(Suppl 1)**:PP126

**Introduction**: 2S albumins have been described as food allergens constituting important clinical diagnostic tools in food allergies. Despite their relatively low sequence similarity, there are studies focused to their potential role in cross-reactivity reactions, so they can be used as a tool for their prediction.

The aim of this study is to identify ten 2S albumins from seeds and nuts, as well as to characterize its structural parameters and assay them for IgE reactivity with patients’ sera.

**Methods**: Isolation of 2S albumins from extracts using chromatographic methods. After identification by mass-spectrometry, molecular characterization was conducted by electrophoretic methods (1- and 2-dimensional electrophoresis); its secondary structure and thermal stability were studied by circular dichroism spectroscopy. Finally, immunoassays were performed to reveal their allergenic capacity, using allergic mustard-allergic patients’ sera.

**Results**: Proteins have been isolated and purified from extracts by means of two chromatographic steps, a gel filtration and a reverse phase in HPLC. They were identified as 2S albumins by mass-spectrometry of digested peptides (MALDI-TOF). They generally show a helicoidal secondary structure, stable at 85 °C and possess a wide range of pI and heterogeneous polypeptide size and composition.

**Conclusions**: The fingerprint analysis confirmed the purified proteins as 2S albumins, storage proteins already characterized as allergens. Purified proteins shown similar characteristics than those described for this family of proteins. The molecular characterization revealed low molecular masses and acid and basic pI, some of them exhibit the typical two chains-pattern and others, such as the one from sunflower seed, are constituted by one polypetide chain. Circular dichroism studies reveal their high resistance to thermal treatment except the one from pistachio. The characterized 2S albumins will be used as clinical tools in Component-Resolved Diagnosis (CRD) in a big Spanish population of patients allergic to vegetable foods using the potent high-throughput screening technology with ADVIA-Centaurus, to elaborate a more accurate diagnosis and therefore a more effective treatment. This diagnosis is especially relevant having in account the severe symptoms caused by this allergenic family.

### PP127 A systematic approach to identifying food allergens of public health importance

#### Bushra Javed^1,2^, Phil Padfield^1,2^, Matt Sperrin^3^, Angela Simpson^1,2^, E. N. Clare Mills^1,2^

##### ^1^Manchester Institute of Biotechnology, University of Manchester, Manchester, United Kingdom; ^2^Institute of Inflammation and Repair, University of Manchester, Manchester, United Kingdom; ^3^Institute of Population Health, University of Manchester, Manchester, United Kingdom

**Correspondence**: Bushra Javed - bushra.javed@postgrad.manchester.ac.uk

*Clinical and Translational Allergy* 2017, **7(Suppl 1)**:PP127

**Introduction**: Food safety authorities recognise food allergy as a significant public health concern and have defined a list of “priority foods” that must be labelled irrespective of their level of inclusion in a recipe. Allergen molecules of those priority foods are the actual hazard for sensitive individuals but data are often lacking regarding their capacity to cause an allergic reaction. A systematic approach is being taken to assess this for peanut and tree nut allergens.

**Methods**: Legislation from across the world has been searched to compile a list of tree nuts that have to be labelled and from this a search stratgey developed. This was implemented in a pilot study and data used to develop a modified PECO approach to the full systematic review. In addition a set of gradings has been developed to allow data gathered to be critically appraised in a consitent manner.

**Results**: A search strategy and search terms, based on allergen labelling requirements across the world for peanut and tree nuts, has been developed. This was implemented in a pilot study and the retrieved articles were systematically reviewed and an inclusion list generated. Articles included were subject to analysis uing grading crieteria spanning the quality of the patient population used to the quality of an allergen preparation used in a given study.

**Conclusions**: This framework along with scientific grading criteria provides the basis to conduct a systematic literature review to identify the quality of evidence supporting inclusion of particular tree nut species in allergen labelling lists and quality of evidence that certain protein molecules can be classified as an allergen. Such data will allow future development of curated sequence sets of verified allergen molecules which are needed for development of analytical methods for determining allergens in foods.

### PP129 Fish allergy in an aquarium worker

#### Magna Correia, Filipe Benito-Garcia, Cristina Arêde, Susana Piedade, Mário Morais-Almeida

##### Allergy Center, CUF Descobertas Hospital, Lisbon, Portugal

**Correspondence**: Magna Correia - magnacorreia01@hotmail.com

*Clinical and Translational Allergy* 2017, **7(Suppl 1)**:PP129

**Introduction**: Fish is one of the most frequent causes of IgE-mediated food allergy in children in Portugal and rarely begins in adults. Allergy to fish in adulthood can be caused by an occupational exposure.

**Case report**: The authors describe the case of a 29 years old women, with an unusual work (since age of 23): animal handler at an Aquarium, she prepares raw fish (capelin) to feed amphibious without use of personal protection measures (gloves) and also dives in the tanks for cleaning. She has personal history of rhinoconjunctivitis since adolescence and hand eczema since three years ago. In the last two years she started having episodes of facial/lip angioedema and/or nausea/vomiting and the last one with stridor and dyspnea, always after the ingestion of cooked fish meals. Contact urticaria to jellyfish and corals was also reported. Skin prick tests (Bial - Aristegui, Bilbao, Spain) were positive to: codfish, axillary seabream, striped red mullet, pilchard, gilt-head bream and seatrout; negative to: tuna fish, sole, hake, morkfish and salmon.

*Prick by prick* tests were positive to: raw hake, raw and cooked gilthead seabream and raw capelin (the food fish); negative to: cooked hake. Total serum IgE of 727 UI/mL. Specific serum IgE (UniCAP^®^, Thermo Fisher Scientific, Uppsala, Sweden) were weakly positive to: codfish (0.19 KU/L), hake (0.11 KU/L), salmon (0.16 KU/L), pilchard (0.13 KU/L) and sole (0.29 KU/L); negative to: swordfish. She tolerates tuna fish and salmon and eats them regularly. Nevertheless, avoidance of ingestion of other fishes as well as direct fish handling only with personal protection measures were recommended in order to keep on the actual job.

**Conclusion**: Allergic sensitization to fish in adulthood is unusual. There are reports suggesting that sensitization to food allergens may occur outside the intestinal tract, namely through the skin. It is likely that in this case, the fish sensitization has occurred by repeated skin contact with raw fish in the context of occupational exposure.

**Consent to publish**: Consent was obtained for publication of this case report.

### PP134 Quantitative immunoassays for native and denatured major milk allergens, Bos d 5 (β-lactoglobulin) and Bos d 11 (β-casein)

#### James Hindley^1^, Ross Yarham^1^, Anna Kuklinska-Pijanka^1^, David Gillick^1^, Karine Patient^2^, Herve Bernard^2^, Martin D. Chapman^1^

##### ^1^Indoor Biotechnologies Ltd, Cardiff, United Kingdom; ^2^INRA-CRJJ, Jouey-en-Josas, France

**Correspondence**: James Hindley - hindleyjp@indoorbiotech.co.uk

*Clinical and Translational Allergy* 2017, **7(Suppl 1)**:PP134

**Introduction**: Allergy to milk is one the most prevalent allergies affecting around 3% of children. At present there is no effective treatment for milk allergy and therefore strict avoidance is recommended. This however can be difficult as milk is an ingredient in many foods. The development of diagnostics and therapeutics for milk allergy depends on accurate and reliable methods for standardisation. Our aim was to develop quantitative immunoassays that could be used to accurately measure specific milk allergens.

**Methods**: Monoclonal antibody pairs recognising the major milk allergens Bos d 11 (β-casein) and Bos d 5 (β-lactoglobulin) in its native and denatured state and were obtained. Subsequently two-site ELISAs and Luminex xMAP assays were developed using highly purified, IgE validated allergens as standards. The assays were used to measure milk allergens in different types of foods (powdered milk, chocolate mousse, chocolate bar, and cookie) and in diagnostic/therapeutic preparations. They were also used to monitor potential milk contamination in products that claim to be ‘milk free’. The Luminex assays for Bos d 5 and Bos d 11 were “multiplexed” to allow for simultaneous quantification of both allergens in a single sample.

**Results**: All assays showed wide standard dynamic range. The Lower Limit of Detection (LLOD) for ELISA was 7.8 ng/ml for both native and denatured Bos d 5 and 15.6 ng/ml for Bos d 11. The Luminex assays proved to be even more sensitive with LLOD up to 40-fold lower compared to ELISA (0.2 ng/ml, 2.0 ng/ml and 2.4 ng/ml for native Bos d 5, denatured Bos d 5 and Bos d 11 respectively). Concentration of Bos d 5 in tested samples ranged from 12.3 mg/g in milk powder to 0.279 µg/g in a placebo “milk free” chocolate bar developed for research.

**Conclusions**: These data demonstrate that these immunoassays are suitable for the quantification of specific milk allergens, in their native and denatured forms, as well as in food samples containing relatively low levels of milk allergen. Immunoassays for quantification of specific milk allergens have been developed. The assays can be used separately (as ELISA) or be “multiplexed” (Luminex assays) allowing simultaneous quantification of multiple allergens in a single sample. The immunoassays will allow standardization of milk protein levels in diagnostic and therapeutic extracts and detection of milk allergens in foods.

### PP136 Gluten, enzymatic or acid hydrolysed gluten does not induce sensitisation by the oral route in contrast to i.p. dosing: a study in gluten-tolerant Brown Norway rats

#### Charlotte Madsen, Katrine L. Bøgh

##### National Food Institute, Technical University of Denmark, Søborg, Denmark

**Correspondence**: Charlotte Madsen - charm@food.dtu.dk

*Clinical and Translational Allergy* 2017, **7(Suppl 1)**:PP136

**Introduction**: Hydrolysed wheat proteins have provoked IgE mediated symptoms after skin exposure or ingestion in subjects tolerant to wheat. Acid hydrolysed wheat protein in a facial soap has been described to break tolerance to wheat. We have previously investigated the sensitising capacity of wheat gluten (G), enzymatic hydrolysed (EHG) and acid hydrolysed gluten (AHG) in naïve Brown Norway rats (Kroghsbo et al. 2014). The aim was to study the influence of wheat tolerance on the sensitising capacity of G, EHG and AHG.

**Methods**: Rats were bred for at least two generations on ordinary wheat-containing rat chow. After weaning the rats were kept on chow and then moved to a gluten free diet. Blood was drawn before dosing day 0 and a week after termination of dosing. Oral study: Rats were dosed by gavage with 20 mg G, EHG or AHG daily for 35 days. I.p. study: Rats were dosed with G, EHG or AHG 320 µg protein day 0, 14 and 28. Sera were analysed for specific IgG1 and IgE to the respective protein by ELISAs. Cross-reactivity was measured by inhibition ELISA’s and avidity by KSCN ELISA.

**Results**: At baseline >50% of the rats had low IgG1 titre to G, but no detectable IgE to G. Oral dosing with G, EHG or AHG did not induce significant changes in the level of specific IgG1 and IgE to either the product used for dosing or to G. I.p. dosing resulted in a significant increase in IgG1 antibodies to the respective proteins and in IgE to G and AHG. The G dosed rats had IgG1 with the highest avidity. IgG1 from AHG immunised rats showed a higher avidity to G than to the AHG. The inhibition ELISA’s showed a high level of similarity within and between the groups.

**Conclusions**: In contrast to our study in naïve rats, oral dosing in wheat tolerant rats could not induce a specific immune response above baseline. In the i.p. dosed animals the high level of cross-reactivity indicates a response dominated by antibodies to epitopes that are similar on all three products. The lower avidity to AHG (containing novel epitopes) compared to G in AHG dosed animals suggests a role for affinity maturation in the wheat tolerant animals. Exposure by the oral route to EHG or AHG is very unlikely to break an already established tolerance to wheat and induce sensitisation. In the i.p. study prior affinity maturation to common epitopes on G and AHG makes it possible to induce a high avidity immune response to G by dosing with AHG. This may illustrate how AHG in a facial soap could break tolerance to wheat.

### PP138 Food-dependent exercise-induced anaphylaxis – A case report

#### Ana Miranda, Eugénia Matos, Anna Sokolova

##### Hospital Prof. Doutor Fernando Fonseca, Amadora, Portugal

**Correspondence**: Anna Sokolova - asokolova@netcabo.pt

*Clinical and Translational Allergy* 2017, **7(Suppl 1)**:PP138

**Introduction**: Food-dependent exercise induced anaphylaxis (FDEIA) is a distinct form of food allergy induced by physical exercise. The ingestion of specific food are usually tolerated if not followed by exercise. FDEIA patients generally have not experienced symptoms in response to warmth (such as a hot bath), or other condition that increases the core body temperature, and they have symptoms only in association with an ingestion of specific food(s). A variety of foods have been described as causal allergy inducing foods in FDEIA. Skin tests and in vitro serum food-specific IgE assays are currently used, but the results are frequently negative. A challenge test consisting of ingestion of assumed food followed by intense physical exercise is the only reliable method to diagnose the disease, but this is not always safe to perform. Monosodium glutamate is a common added ingredient to savoury foods, namely in Chinese food, and it has been previously linked to urticaria and angioedema.

**Case report**: We present a case of a 16-year-old white boy that developed within a few minutes from onset of physical activity: periorbital edema, urticaria, generalized pruritus and cough. In the emergency department, he was hypotensive (79/50 mmHg). He was treated with intramuscular epinephrine, clemastine and methylprednisolone and symptoms resolved completely. 4 h before the onset of physical activity he eats Chinese food, including pasta, shrimp and pork meat, which were previously tolerated by him. He regularly exercises without any problem. There was no past personal history of anaphylaxis, angioedema, food allergy, atopic dermatitis, drug allergy or vaccine allergy. He had no concurrent illness that day. He was not exposed to other foods, alcohol or medication (including NSAIDs, aspirin) several hours prior to exercise. There was no exposure to extreme temperature changes. Skin prick testing, serum-specific immunoglobulin E level and ImmunoCAP ISAC were negative. The challenge test was not performed due to severity of clinical presentation.

**Conclusion**: This case is an important reminder that although rare, food-dependent exercise-induced anaphylaxis exists. Making a diagnosis can be difficult but it can lead to life-saving preventative strategies. Besides some food additives, like monosodium glutamate, may be implicated in some of these reactions. In this case there is indication to avoid concomitant ingestion of the food and exercise.

**Consent to publish**: Informed consent to use the data was obtained from the patient.

### PP140 Immunogenic and allergenic properties of egg white proteins as affected by different denaturation processes

#### Alba Pablos-Tanarro, Daniel Lozano-Ojalvo, Rosina López-Fandiño, Elena Molina

##### Instituto de Investigación en Ciencias de la Alimentación, Madrid, Spain

**Correspondence**: Alba Pablos-Tanarro - a.pablos@csic.es

*Clinical and Translational Allergy* 2017, **7(Suppl 1)**:PP140

**Introduction**: 70–80% of children with egg allergy can eat baked foods containing highly heated egg, showing that denaturation of egg proteins greatly modifies their allergenic potential. However, little is known about the effects of moderate denaturation treatments on the allergenicity of egg white proteins. We sought to investigate to what extent different technological processes that lead to a limited denaturation of the egg proteins can affect their immunogenic capacity. Thus, in this study, we compared the ability to sensitize and trigger allergic responses of raw egg white (R-EW), pasteurized egg white (H-EW, 80°C-10 min) and high-pressure treated egg white (P-EW, 400 mPa-10 min).

**Methods**: BALB/c mice were orally sensitized during 7 weeks with 5 mg of either raw, heated or pressurized egg white (R-EW, P-EW or H-EW). One week after the last sensitization dose, mice were orally challenged with 50 mg of the egg white protein preparation used for sensitization and the anaphylactic responses were evaluated. During the sensitization protocol, EW-specific IgG1 and IgE were quantified weekly. Passive cutaneous anaphylaxis (PCA) tests were performed in order to know whether the immune response was IgE-mediated or both IgG1- and IgE-mediated. Cytokine production was also measured in allergen-stimulated spleen cell cultures.

**Results**: The highest liberation of mast cell protease, as well as the most severe anaphylactic signs and body temperature drop, were observed in P-EW sensitized and challenged mice, followed by R-EW sensitized and challenged mice. Moreover, splenocytes from mice sensitized to P-EW produced more Th2 cytokines, IL-4 and IL-5, than those from mice from the other groups in response to stimulation with the allergen. Regarding the humoral response, R-EW and P-EW promoted the highest levels of specific IgG1 at the end of the sensitization protocol, while H-EW stimulated the highest production of IgE. PCA tests confirmed that IgG1 plays an important role on the allergic reactions to R-EW and P-EW, whereas reactions to H-EW were mediated by IgE to a higher extent.

**Conclusions**: Denaturation of egg white proteins with high-pressure increases their sensitizing and eliciting capacity. Egg white heated to pasteurization temperatures exhibits a reduced capacity to induce anaphylactic responses despite the fact that it actively induces the production of specific IgE.

### PP141 The effect of the food matrix on the bioavailability and IgE reactivity of peanut of allergens

#### Huan Rao^1,2^, Ivona Baricevic-Jones^1^, Frances Smith^1^, Rebekah Sayers^1^, Phil Padfield^1^, Angela Simpson^3^, E. N. Clare Mills^1^, Wentong Xue^2^

##### ^1^Institute of Inflammation and Repair, Manchester Institute of Biotechnology, University of Manchester, Manchester, United Kingdom; ^2^College of Food Science and Nutritional Engineering, China Agricultural University, Beijing, China; ^3^Allergy Centre, University Hospital of South Manchester, Manchester, United Kingdom

**Correspondence**: Huan Rao - huan.rao@postgrad.manchester.ac.uk

*Clinical and Translational Allergy* 2017, **7(Suppl 1)**:PP141

**Introduction**: Food processing and interactions involved in different food components affect the bioavailability and immunoreactivity of allergens. However, the impacts of different food matrices on protein digestibility and IgE reactivity of peanut of allergens are poorly understood. We are using an in vitro digestion model to evaluate the effects of digestion on protein release and immunoreactivity of allergens from peanut flour, and the same peanut flour incorporated in to the EuroPrevall dessert and iFAAM cookie matrices used for oral food challenges in the iFAAM project.

**Methods**: Digestibility of roasted peanut flour, peanut dessert and peanut cookie was assessed using an in vitro digestion system which includes a model chew, followed by simulated gastric and duodenal digestion system. Protein digestion was monitored by SDS-PAGE, and immunoreactivity was specifically analyzed by immunoblotting using a serum panel from peanut-allergic subjects from the ManARTS biobank, using a combination of immunoblotting and immunoassay methods.

**Results**: Roasted peanut flour proteins proved highly digestible following gastric-duodenal digestion with only low molecular weight peptides of Mr < 6 kDa being visible. Gluten containing baked matrices are slower to digest and hence release and digestibility from the cookie matrix is expected to be slower than that from the water continuous EuroPrevall chocolate dessert matrix. Undertaking gastric digestions at higher pH (6.5) reflecting the affect of taking ant-acids also reduced the rate of peanut allergen digestion.

**Conclusions**: Food matrices can have complex effects on the release and digestion of allergens during simulated in vitro digestion. Future studies will apply mass spectrometry profiling methods to determine how both the food matrices and increased pH affect the patterns of proteolysis, especially given the pH-dependent nature of pepsin specificity.

### PP144 Peanut components causing peanut allergy in Iceland

#### Sigurveig Sigurdardottir^1^, Helga Magnusdottir^1,2^, Anna G. Vidarsdottir^1^, Michael Clausen^3^, Sigrun Lund^2^, Anders Blom Jensen^4^, Bjorn R. Ludviksson^1^

##### ^1^Department of Immunology, Landspitali University Hospital, Reykjavik, Iceland; ^2^University of Iceland, Reykjavik, Iceland; ^3^Children’s Hospital, Landspitali University Hospital, Reykjavik, Iceland; ^4^Thermo Fisher Scientific, Copenhagen, Denmark

**Correspondence**: Sigurveig Sigurdardottir - veiga@lsh.is

*Clinical and Translational Allergy* 2017, **7(Suppl 1)**:PP144

**Introduction**: Sensitivity to peanut allergens varies between countries, reflecting environmental factors such as local pollen and genetics. Arah1, 2 and 3 are considered the major peanut allergens whereas Arah8 is a homologue of the birch pollen allergen Betv1 and is not associated with severe allergic reaction. Ara h 6 is cross-reactive with Arah2 but may also be a single sensitizer. The aims were to determine the component pattern of peanut sensitization in Iceland and relate to the history of allergic reaction to peanuts as well as to pollen allergy.

**Methods**: Serum samples from 220 individuals obtained during a 25 month study period and were positive for peanut specific IgE (Pn-IgE) were used to measure by ImmunoCAP (Thermo Fisher Scientific, Uppsala, Sweden); Arah1-, Arah2-, Arah3-, Arah8- and Betv1-IgE. Information on age, gender, personal- and family history of atopy and skin prick tests were obtained from medical records. Arah2-IgE negative individuals were interviewed and invited to an open peanut challenge. Those who reacted to peanuts were evaluated for Arah6-IgE by ISAC (Thermo Fisher Scientific, Uppsala, Sweden).

**Results**: The main atopic findings within the study cohort of 220 Pn-IgE positive individuals included history of eczema (75.5%), asthma (65.5%), and allergic rhinitis (65.5%). Arah2-IgE was negative in 52.3% (115/220). Of those, 24.3% (28/115) were already consuming peanuts, 29.6% (34/115) had a negative challenge, 5.2% (6/115) had a positive challenge and 2,6% (3/115) had an inconclusive one. Forty four (38.3%) rejected or were unable to undergo a challenge. Of the Arah2 negative and challenge positive individuals, three were sensitive to Arah6, (one single-sensitive), two were sensitive to Arah1 and 3 and one was borderline sensitive to Arah1, 2 and 3. Two inconclusive cases were positive to Arah6. Those who were challenge positive had a higher Arah1-IgE.

**Conclusions**: Peanut sensitized individuals in Iceland are highly atopic and half of them are not sensitized to the major allergen Arah2. This is only partly explained by birch sensitization and sensitization to other components such as Arah1 and Arah6 is important.

### PP145 The stability of peanut allergens in AR101, an oral immunotherapeutic for the treatment of peanut allergy, in two representative food matrices

#### Reyna Simon^1^, Robert Elfont^1^, Sean Bennett^1^, Robert Voyksner^2^

##### ^1^Aimmune Therapeutics, Brisbane CA, USA; ^2^LCMS Ltd., Durham NC, USA

**Correspondence**: Reyna Simon - rsimon@aimmune.com

*Clinical and Translational Allergy* 2017, **7(Suppl 1)**:PP145

**Introduction**: Peanut allergy is an increasingly common health problem. While there are currently no approved treatments specifically for peanut allergy, oral immunotherapy (OIT) has shown promising results. In OIT, peanut allergens (often in the form of peanut flour) are typically delivered by mixing into vehicle foods and then consuming. Also, in OIT clinical trials, patients undergo food challenges, where the peanut allergens are mixed into vehicle food. To insure consistent dosing during OIT and during food challenges, it is important to show that the food matrices do not compromise the integrity of the allergens. Aimmune Therapeutics is currently enrolling patients in a Phase 3 OIT study (PALISADE) to demonstrate the efficacy and safety of AR101, a formulated drug product containing peanut protein at different dosage strengths. It is taken orally following mixing with food. The aim of this study was to determine the stability of peanut allergens over a 24 h period following mixing with foods used in the PALISADE food challenge, applesauce and chocolate pudding, foods also commonly used as vehicles for OIT dosing.

**Methods**: Peanut flour (PF), Peanut-Food Challenge Material (PFCM; peanut flour mixed with food-grade flavorings and bulking agents), and AR101 (100 mg) were each mixed with food and sampled at 0, 4, and 24 h. Proteins were extracted from the food mixtures and analyzed by peanut specific ELISA and LCMSMS. Total peanut allergen was quantitatively measured in an ELISA (R-Biopharm Ridascreen Fast-Peanut). LCMSMS identified two marker peptides each for Ara h 1, h 2, and h 6 following protein extraction, digestion, and alkylation. These were compared over the course of 24 h to a no-food-matrix control.

**Results**: ELISA: Ranges of peanut protein (ppm peanut) recovered with AR101 extraction from applesauce (2 samples analyzed in duplicate) were: 3.3–4.2 (t = 0 h), 3.9–4.3 (4 h), 3.3–3.8 (24 h). Ranges for AR101 extracted from chocolate pudding were: 3.2–3.6 (0 h), 4.0–5.7 (4 h), 3.6–4.5 (24 h). Data were similar for PF and PFCM. LCMSMS: For all marker peptides, peak areas were within 30% of t = 0 h for all food matrices (single samples, triplicate measurement).

**Conclusions**: When mixed into applesauce or chocolate pudding, there was no observable degradation of the samples over 24 h, insuring sufficient stability to accommodate any reasonable variability in time taken to consume a dose.

### PP146 Severe banana allergy

#### Anna Sokolova^1^, Maria de Lurdes Torre^1^, Borja Bartolomé^2^

##### ^1^Hospital Professor Doutor Fernando Fonseca EPE, Amadora, Portugal; ^2^BIAL Industrial y Farmacéutica S.A., Zamudio, Spain

**Correspondence**: Anna Sokolova - asokolova@netcabo.pt

*Clinical and Translational Allergy* 2017, **7(Suppl 1)**:PP146

**Introduction**: Food allergy is the most common cause of anaphylaxis in children. Banana fruit appears to have become an important cause of fruit allergy in children. The allergens involved are poorly characterized.

**Case report**: We report a clinical case of an 18-year-old boy with generalized urticaria and oropharyngeal pruritus with dyspnoea immediately after banana ingestion at the age of 12. Since then he has eliminated this fruit from his diet and has no complaints with any other foods, namely of rosaceae family. Skin prick tests performed with commercial extracts were positive for pollen from Olea europeae 10 mm and banana 7 mm. The past medical history is remarkable for Crohn’s disease. Specific IgE measured by EAST technique was 1.94 kU/L for Olea europeae pollen extract and 0.78 kU/L for banana pulp extract, 1.2 kU/L for banana peel extract and 17.5 kU/L for Pru p 3 (LTP). The molecular mass of the IgE binding bands was calculated by SDS PAGE immunoblotting in reducing and non-reducing electrophoretic conditions. The apparent molecular mass of the IgE -binding bands were: 20 kDa in banana pulp extract and 9 kDa for banana peel extract (with some less intense bands of 66, 50, 40, 30, 29 kDa). The immunoblotting-inhibition method was used to study if the 9 kDa binding band was an LTP protein. Banana peel extract was used as a solid phase and Pru p 3 as inhibition phase. Pru p 3 was able to produce a partial IgE-binding inhibition on the 9 kDa band. Oral food challenge was not performed due to Crohn’s disease.

**Conclusion**: Some of the detected IgE-binding bands in banana extracts show similar molecular mass to the known allergens, namely Mus a 4- thaumatin-like protein (20 kDa in banana pulp extract), Mus a 5- beta 1.3 glucanase (30 kDa), Mus a 2- chitinase (33 kDa) in banana peel extract. Most likely, the main IgE-binding band of 9 kDa from banana peel extract is the Mus a 3 LTP protein. High sensitization to Pru p 3 allergen has no clinical manifestations in our patient. We speculate about the association between food allergy and inflammatory bowel disease in this particular case.

**Consent to publish**: Informed consent to use the data was obtained from the patient.

### PP147 Who is at risk for severe reactions during oral food challenges?

#### Songül Yürek, Kirsten Beyer, Bodo Niggemann

##### Department of Pediatric Pneumonology and Immunology, Charité Universitätsmedizin Berlin, Berlin, Germany

**Correspondence**: Songül Yürek - songuel.yuerek@charite.de

*Clinical and Translational Allergy* 2017, **7(Suppl 1)**:PP147

**Introduction**: Oral food challenges (OFC) are the gold standard for the diagnosis of food allergy in children. Using a standardized approach with semi-log dose increments every 30 min OFC usually represent a safe procedure without requiring intensive care. Here we describe three cases of severe anaphylactic reaction during OFC in order to identify potential risk factors in children.

**Case report**: Among the last 1500 DBPCFC performed in our clinic we had three cases of severe anaphylactic reactions requiring ICU transferral. Surprisingly the reactions in our cases were not caused by peanut but by cow’s milk (CM), hen’s egg (HE) and hazelnut (HN). Three male patients (9, 12 and 16 years of age) each with a medical history of atopic eczema and bronchial asthma showed severe anaphylactic reactions with pulmonary obstruction and recurrent drop in blood pressure requiring two or more intramuscular adrenaline injections, administration of volume boluses, inhalation of salbutamol and oxygen, and in two cases application of continuous intravenous adrenaline. All patients started to react at higher titration steps: The eliciting doses were 0.34 g of CM, 0.20 g of hazelnut and 1.55 g of HE protein, respectively.

**Conclusion**: Severe anaphylactic reactions requiring intensive care during oral food challenges are rare and seem to occur most often in older children suffering from bronchial asthma. We postulate the following assumptions:

- Severe reactions are not limited to the well-known potentially life-threatening peanut allergen but may also occur with CM, HE and hazelnut. Especially older children around school age who did not outgrow their CM or HE allergy may be at a higher risk for anaphylaxis.
- Next to respiratory problems, arterial hypotension seems to play an important role in children and adolescents, which indicates that the administration of intravenous volume boluses in combination with intramuscular adrenaline is a part of severe anaphylaxis treatment that should not be underestimated.
- Since severe reactions are not foreseeable, OFC should be performed under medical surveillance in a controlled clinical setting with access to an intensive care unit.

**Consent to publish**: Written informed consent to use the data was obtained for all patients.

### PP148 Effects of avoidance of hazelnut upon IgE reactivity profiles and basophil responsiveness in hazelnut allergic and sensitized children

#### Margaretha A. Faber^1^, Annick Bastiaensen^1^, Evelyne Mangodt^1^, Athina van Gasse^1^, Ine Decuyper^1,2^, Vito Sabato^1^, Margo M. Hagendorens^1,2^, Chris H. Bridts^1^, Luc S. De Clerck^1^, Didier Ebo^1^

##### ^1^Department of Immunology-Allergology-Rheumatology, Faculty of Medicine and Health Sciences, University of Antwerp, Antwerp University Hospital, Antwerp, Belgium; ^2^Department of Pediatrics, Antwerp University Hospital, Antwerp, Belgium

**Correspondence**: Athina van Gasse - athinavg@hotmail.com

*Clinical and Translational Allergy* 2017, **7(Suppl 1)**:PP148

**Introduction**: Hazelnut allergy shows distinct clinical patterns depending on the sensitization profile, which shows variations according to age. This study aims at determining whether alterations in the sensitization profile to hazelnut occurs in early childhood.

**Methods**: Twenty-one infants with atopic dermatitis demonstrating an early sensitization to hazelnut (AD-group) and 9 children (<4 years old) with a history of hazelnut allergy (GR-group) were selected. At baseline (T0) children were recommended to avoid hazelnut. At T0 and follow-up consultation (T1) history taking and specific immunoglobulin E (sIgE) measurement to hazelnut extract and its components were performed. A basophil activation test (BAT) with hazelnut was performed at T0 and T1 in children of the GR-group and at T1 in children of the AD-group.

**Results**: Median follow-up was 6.2 years. At T1, a significant increase in sensitization to Cor a 1.04, Cor a 8, Cor a 11, Cor a 14 and Bet v 1 was observed in the AD-group. Six (29%) infants from the AD-group had uneventful accidental exposure to hazelnut, all of them showed a negative BAT despite positive sIgE results. Children out of the GR-group were significantly more frequently sensitized to Cor a 1.04 and Bet v 1 at T1. Sensitization to other components and BAT results did not significantly differ between T0 and T1.

**Conclusions**: Despite avoidance of hazelnut, sensitization profiles can broaden during early childhood. However, in the absence of a generalized hazelnut allergy, the clinical relevance of these serological changes remain elusive as the majority of these children demonstrate negative BATs.

### PP149 A standardised protocol for double-blind placebo-controlled food challenges with heated hen’s egg that is easy to perform

#### Susanne Schwarz, Mandy Ziegert, Saskia Albroscheit, Valerie Trendelenburg, Bodo Niggemann, Kirsten Beyer

##### Department of Pediatric Pneumology and Immunology, Charité Universitätsmedizin Berlin, Berlin, Germany

**Correspondence**: Susanne Schwarz - susanne.schwarz2@charite.de

*Clinical and Translational Allergy* 2017, **7(Suppl 1)**:PP149

**Introduction**: Hen’s egg (HE) allergy is one of the most common food allergies in early childhood. However, previously it has been shown that about 80% of children allergic to raw HE can tolerate heated HE. As double-blind placebo-controlled food challenges (DBPCFC) are the gold standard in food allergy diagnostic, we intended to establish a standardized DBPCFC-protocol for heated HE that is simple in regard to preparation, blinding and titration and well accepted even by young children.

**Methods**: For the DBPCFC with heated HE, 55 g raw pasteurized HE (Wiesenhof) that equals 1 HE (7,095 g HE protein) was heated for 1 min in a microwave (800 W) leading to 45 g heated HE. As a blinding matrix applesauce with cinnamon, chocolate pudding or vegetable puree was added. The mixture was blended and titrated in seven incremental dose steps containing 0.03, 0.1, 0.3, 1, 3, 10 and 30 g of heated HE. For placebo the equivalent blinding matrix was used and rice powder was added to match the texture. If no reaction occurred during the titrated DBPCFC, a cumulative dose of 45 g heated HE was given another day. Children with allergic reaction to raw HE who tolerated at least 0.4 g of raw HE during the challenge were eligible for a challenge with heated HE.

**Results**: 50 children with allergic reaction to raw HE performed a DBPCFC with heated HE according to the protocol described above within 3 month after their positive DBPCFC to raw HE. The challenge meal was well accepted by every child. 43/50 children (86%) showed no allergic reaction to heated HE whereas 7/50 (14%) were allergic to raw and heated HE.

**Conclusions**: Our standardized protocol for a DBPCFC with heated HE showed a high acceptance even in young children in regard to volume and taste. Using this protocol allows a simple, less time-consuming preparation method compared to baked egg protocols, especially if no oven is available on site. Furthermore it allows a more precise titration and masking of the DBPCFC. Our data on DBPCFC with heated HE confirm published data from other countries that the majority of children with HE allergy tolerate heated HE. Preparation of heated HE in a microwave is a simple method for a DBPCFC with heated HE. A high proportion of children with raw HE allergy can tolerate heated HE, therefore standardized DBPCFC with heated HE should be performed in order to improve quality of life of HE allergic children.

### PP150 Association of peanut oleosins with severe allergic symptoms: new marker for clinical severity of peanut allergy?

#### Christian Schwager^1^, Skadi Kull^1^, Jochen Behrends^2^, Niels Röckendorf^3^, Frauke Schocker^1^, Andreas Frey^3^, Arne Homann^1^, Wolf-Meinhard Becker^1^, Uta Jappe^1,4^

##### ^1^Division of Clinical and Molecular Allergology, Research Center Borstel, Priority Area Asthma and Allergy, Airway Research Center North (ARCN), German Center for Lung Research (DZL), Borstel, Germany; ^2^Core Facility Fluorescence Cytometry, Research Center Borstel, Borstel, Germany; ^3^Division of Mucosal Immunology and Diagnostics, Research Center Borstel, Priority Area Asthma and Allergy, Airway Research Center North (ARCN), German Center for Lung Research (DZL), Borstel, Germany; ^4^Interdisciplinary Allergy Outpatient Clinic, Department of Internal Medicine, University of Luebeck, Luebeck, Germany

**Correspondence**: Christian Schwager - cschwager.fz-borstel@gmx.de

*Clinical and Translational Allergy* 2017, **7(Suppl 1)**:PP150

**Introduction**: Allergic reactions to peanut are on the rise in Western countries. In vitro diagnostic tests applying aqueous extracts or water-soluble single allergens are investigated as an alternative to costly and potential life-threatening oral food challenges (OFC). However, their diagnostic accuracy to predict the degree of clinical severity is still inferior to OFCs which might be based on the absence of lipophilic allergens such as oleosins. Recently, sensitization to oleosins has been associated with more severe allergic symptoms in allergy to sesame and hazelnut. Hence, we sought to investigate oleosins as possible new candidates for routine diagnostic measurements in peanut allergy.

**Methods**: Oleosins from raw and in-shell roasted peanuts were isolated by flotation centrifugation and purified by preparative electrophoresis. Protein identification was carried out by N-terminal sequencing and mass spectrometry. Sensitization prevalence to oleosins from raw and in-shell roasted peanuts was investigated by western blot. The ability of oleosins to trigger type I hypersensitivity reactions was evaluated by basophil activation test (BAT).

**Results**: A number of eight oleosins were isolated and identified from peanut. So far, these molecules showed IgE binding exclusively to sera from patients suffering from severe peanut allergy. Moreover, IgE binding to oleosins obtained from in-shell roasted peanuts was increased compared to raw ones. Both, oleosins from raw and in-shell roasted peanuts were able to stimulate basophils from peanut-allergic individuals having severe symptoms.

**Conclusions**: Oleosins are important lipophilic allergens that are able to trigger allergic reactions but have been overlooked so far due to their low solubility in aqueous buffers. Sensitization to these proteins seems to be solely associated with severe allergic symptoms. Peanut oleosins are clinically relevant peanut allergens. As they are most likely associated with more severe allergic symptoms they might be new maker candidates for the clinical severity of peanut allergy. Furthermore, in-shell roasting increases the IgE binding potency of oleosins which is in line with previous results for other peanut allergens.

### PP151 Cyp c 1 or Gad c 1 for fish allergy component-resolved diagnostics: is this a question?

#### Caroline Klingebiel^1^, Nesrine Zaabat^2,3^, Sylvia Osscini^1^, Chantal Agabriel^4^, Benoît Sterling^5^, Ania Carsin^6^, Valérie Liabeuf^7^, Isabelle Cleach^8^, Jean-Louis Mège^8,9^, Joana Vitte^8,9^

##### ^1^Laboratoire Montgrand, LBM Multisite SELDAIX-BIOPLUS, Marseille, France; ^2^Unité Allergologie et Exploration du Complément, Laboratoire d’Immuno-chimie et de Neuro-immunologie, Département d’Immunologie, Institut Pasteur d’Algérie, Dély-Brahim, Algeria; ^3^Laboratoire d’Immunologie, Département de Pharmacie, Faculté de Médecine d’Alger, Ben Aknoun, Algeria; ^4^Service de Pédiatrie Multidisciplinaire, Hôpital de la Timone, Assistance Publique Hôpitaux de Marseille, Marseille, France; ^5^Service de Pédiatrie, Hôpital Nord, Assistance Publique Hôpitaux de Marseille, Marseille, France; ^6^Service de Pneumologie Pédiatrique, Hôpital de la Timone, Assistance Publique Hôpitaux de Marseille, Marseille, France; ^7^Service de Dermatologie et Vénéréologie, Hôpital de la Timone, Assistance Publique Hôpitaux de Marseille, Marseille, France; ^8^Laboratoire d’Immunologie, Hôpital de la Conception, Assistance Publique Hôpitaux de Marseille, Marseille, France; ^9^Aix-Marseille Université, Marseille, France

**Correspondence**: Joana Vitte - jvitte@hotmail.fr

*Clinical and Translational Allergy* 2017, **7(Suppl 1)**:PP151

**Introduction**: To compare the performance of rGad c 1 and rCyp c 1 in fish sIgE component-resolved diagnostics.

**Methods**: Retrospective analysis of laboratory reports for 78 outpatients (median age 12 years, range 0.8–66; 40 males) having undergone fish sIgE component-resolved diagnostics between November 2011 and July 2016. Comparison of ImmunoCAP 250 results for sIgE to rCyp c 1 and rGad c 1 was done for 46 patients (22 males; median age 13 years, range 0.8–66). Comparison of ImmunoCAP 250 and ISAC 112 results was done for 46 patients (26 males; median age 13; range 3–58; 17 rCyp c 1 + rGad c 1+ ISAC; 29 rCyp c 1 or rGad c 1 + ISAC).

**Results**: 44/46 (96%) singleplex assays yielded concordant pos/neg results (18 double negative, 26 double positive). Both discordant results were rescued by rCyp c 1 testing (0.44 and 0.11 kUA/L). Among double positive results, median levels of sIgE to rCyp c 1 were slightly higher (3.1 vs 2.5 kUA/L, not significant). 39/46 (85%) ISAC 112 and ImmunoCAP 250 assays yielded concordant pos/neg results (9 double negative, 30 double positive). All discordant results (7/46 = 15%) were negative with ISAC 112 and positive with ImmunoCAP 250 (sIgE to rCyp c 1 0.18–12.4 kUA/L; sIgE to rGad c 1 0.18–0.65 kUA/L). Among double positive results, median levels of sIgE to rGad c 1 were slightly higher with ISAC 112 (median 6.1 ISU, range 0.8–89) than with ImmunoCAP rGad c 1 (5.1 kUA/L, range 0.67–68) and ImmunoCAP rCyp c 1 (4.2 kUA/L, range 0.56–156), but this difference was not significant.

**Conclusions**: Currently, two recombinant beta-parvalbumins are available for fish sIgE component-resolved diagnostics. Both can be used for singleplex ImmunoCAP assays, while ISAC 112 only contains rGad c 1. Data on rCyp c 1 and rGad c 1 performances are scarce and have not been updated for ISAC 112. In our hands, singleplex rCyp c 1 and rGad c 1 display very similar performances in a vast majority of patients. rCyp c 1 may offer better detection for low values of 1 kUA/L or less. rGad c 1 microarrayed on ISAC 112 lacks sensitivity for low values (15% false negative), but positive results are well correlated with singleplex rGad c 1 and rCyp c1. Singleplex assay of sIgE to rCyp c 1 offers a very slight increase in sensitivity of detection compared with rGad c 1. rGad c 1 microarrayed on ISAC 112 is prone to false negatives (up to 15%) for low levels, but when positive it is well correlated to singleplex results.

### PP152 Knowledge of parents about the elimination diet and food allergens

#### Monica Maćków^1^, Alina Zbróg^1^, Monica Bronkowska^1,2^

##### ^1^Faculty of Health Science and Physical Education, Witelon State University of Applied Sciences, Legnica, Poland; ^2^Faculty of Human Nutrition, University of Environmental and Life Sciences, Wroclaw, Poland

**Correspondence**: Monica Maćków - dietmonica@gmail.com

*Clinical and Translational Allergy* 2017, **7(Suppl 1)**:PP152

**Introduction**: The aim of this pilot study was to assess the knowledge of parents of children with food allergies on the elimination diet and allergens, and knowledge of the sources of information about food allergy.

**Methods**: The pilot study was conducted from May to August 2016 among 86 parents in Lower Silesian in Poland, whose children have food allergies. Surveys were distributed to parents in hospitals and nursery school. They were a group of women and men in the age range 18–50 years. Parents’ knowledge was verified by a questionnaire, which has been specially developed for the study.

**Results**: The product most often in Polish children invoke allergic reactions were cow’s milk and eggs. The vast majority of respondents showed knowledge of the definition of the elimination diet and they knew that, when a food allergy elimination diet and you should carefully read the labels of food products. However, the greater part of the interviewees believed that the allergy to cow’s milk should be eliminated from the diet always and only cow’s milk and milk products.

**Conclusion**: Compared to other studies of Polish parents also have basic than parents from other countries. Also among the other respondents to the primary sources of information was Internet and doctor.

Parents of children, who have food allergies, have a basic knowledge on the elimination diet and individual allergens. However, this does not always indicate correctly, which foods you should avoid elimination diet.

This may be due to the fact that the main sources of information were the Internet, the doctor and books about allergies.

### PP153 Hunting for new wheat allergens: a 2D Immunoblot and mass spectrometry approach

#### Justine Courtois^1^, Romy Gadisseur^2^, Catherine Bertholet^2^, Pierre Lukas^2^, Etienne Cavalier^2^, Philippe Delahaut^3^, Birgit Quinting^4^

##### ^1^CRIG Liège, Liège, Belgium; ^2^CHU Liège, Liège, Belgium; ^3^CER Group Marloie, Marloie, Belgium; ^4^HELMo Liège, Liège, Belgium

**Correspondence**: Justine Courtois - j.courtois@crig.be

*Clinical and Translational Allergy* 2017, **7(Suppl 1)**:PP153

**Introduction**: Wheat is a complex allergenic food containing a lot of proteins that are difficult to isolate and to identify. Hence we aimed to develop a diagnostic method linking specific allergenic 2D western blot profiles to a particular clinical symptom in wheat allergy. Afterwards, we used mass spectrometry (LC-MS/MS) to identify molecular allergens.

**Methods**: A total protein extract of wheat seeds was separated on the basis of the isoelectric point and the molecular weight of the proteins. Twenty-five patients presenting positive specific IgE (sIgE) for wheat were classified into 3 different phenotypes: wheat dependent exercise induced anaphylaxis (WDEIA), atopic dermatitis (AD) and pollen rhinitis (PR). Their sera were analyzed by 2D immunoblotting on a standardized wheat seeds extract in order to evaluate their sIgE reactivity against the protein spots. Their sIgE sensitization profiles were compared and protein spots of interest were identified by LC-MS/MS.

**Results**: Specific sensitization profiles were identified for each phenotype group. For WDEIA, protein spots around 37 kDa (pH 6–9) and 37–50 kDa (pH 5–6) were identified. For AD, spots were observed around 50 kDa (pH 9), 10 kDa (pH 9) and 20 to 75 kDa (pH3). For PR, specific spots were situated around 90 kDa (pH 9). The LC-MS/MS analysis of these identified spots pointed out several potential interesting allergens: tri a 26, tri a bA, tri a 34, tri a tritin.

**Conclusion**: Our study answers to the request of many allergists wishing to get an accurate diagnosis of wheat allergy in order to determinate the risk of cross-reaction, to adapt the diet and to limit the risk of anaphylactic choc. Nevertheless, it is necessary to consider the 2D immunoblot results with the medical history of each patient. Moreover, there are different clinical manifestations of wheat allergy depending on the involved allergen and the way of exposure: WDEIA, AD, PR or baker’s asthma. The present project pointed out new wheat allergens that could be associated to a specific phenotype. The identification of further protein spots is still under investigation. At this stage, specific sensitization profiles were identified for the 3 phenotype groups (WDEIA, DA, PR). The protein spots of interest detected by sIgE concern one or more allergens. Some wheat allergens were identified by LC-MS/MS. At the end of the study, it will be possible to establish a link between a specific symptomatology and the responsible allergens newly identified.

## POSTER SESSION 5: Case reports

### PP154 Goat’s milk allergy with an unusual presentation

#### Filipe Benito-Garcia, Magna Correia, Cristina Arêde, Susana Piedade, Mário Morais-Almeida

##### Immunoallergy Department, CUF Descobertas Hospital, Lisboa, Portugal

**Correspondence**: Filipe Benito-Garcia - filipe.benito.garcia@gmail.com

*Clinical and Translational Allergy* 2017, **7(Suppl 1)**:PP154

**Introduction**: Cow’s milk (CM) protein allergy is the most prevalent food allergy in children. Patients with CM allergy usually do not tolerate goat’s (GM) or sheep’s (SM) milk, since there is a high cross-reactivity between all of them. There are few case reports of patients with allergy to GM tolerant to CM.

**Case report**: A 14 years old girl with IgE-mediated CM allergy since 6 months of age, with CM eviction till 12 years old. At this age an oral food challenge (OFC) has been performed, being negative. She started a regular ingestion of CM proteins during the last two years. She was referred to our Immunoallergy department, one week after an episode of lip angioedema and generalized urticaria with intense pruritus after eating a meal containing goat cheese, parsley, tomato, onion, poached egg and pepper. Two weeks before she had already an episode of oral pruritus after eating a small portion of goat cheese. Skin prick tests (SPT) (Bial—Aristegui, Bilbao, Spain) to milk and CM proteins (casein, α-lactoalbumin, β-lactoglobulin), egg and egg’s proteins, pepper and parsley, were negative. *Prick by prick test* (PPT) were positive to goat cheese (19 × 8 mm), GM (11 × 7 mm) and SM (7 × 9 mm). In order to validate these results, PPT with goat’s cheese and milk and SM have been performed to 10 healthy adult controls. Specific IgE (UniCAP^®^, Thermo Fisher Scientific, Uppsala, Sweden) was positive to GM (8.7 kU/L) and SM (9.3 kU/L) and negative for CM and proteins. Total IgE 241 kU/L. Two weeks after the episode, an OFC with CM was performed, being negative, and daily ingestion of CM proteins has been advised with good tolerance.

**Conclusions**: The majority of patients with allergy to CM proteins do not tolerate GM or SM. We describe a rare case of an adolescent girl who started to react to goat’s cheese after outgrown a CM allergy, probably due to sensitization to proteins without homology to CM proteins.

**Consent to publish**: The authors confirm that the individual described has authorized them to publish the findings related with the case.

### PP155 Food challenge in two patients with asthma and Ara h2 verified peanut allergy

#### Margareta Brandt Gertmo, Ewa Ternesten Hasseus, Jenny Van Odijk

##### Sahlgrenska Universityhospital, Gothenburg, Sweden

**Correspondence**: Margareta Brandt Gertmo - margareta.b.gertmo@gmail.com

*Clinical and Translational Allergy* 2017, **7(Suppl 1)**:PP155

**Introduction**: IgE antibodies to peanut storage protein Ara h 2 have high diagnostic accuracy in verifying or excluding symptomatic peanut allergy, and high levels are considered to predict serious allergic reactions. In our experience some adult patients with peanut allergy suffer disabling fear of peanuts, having been taught that they risk a severe allergic reaction upon airborne exposure of, or consumption of peanuts.

**Case report**:

1. Female, 24 years old with asthma. The patient had been told to exclude peanuts from her diet and not to be in a room where peanuts are consumed. She had always carried an adrenaline auto-injector. Current level of Ara h2 was 9.3 kU/L. She had never experienced a severe allergic reaction and was eager to find out how she would react to accidental intake of peanuts.
  Following a strict protocol, she consumed one peanut and experienced instantly some mild oral symptoms. After 20 min she reacted with profuse vomiting and a stuffy nose. No adverse respiratory or circulatory reactions occurred and no adrenaline or other medication was given. The recovery was swift and the patient left the clinic after two hours.
2. Male, 20 years old with asthma. At the age of seven he reacted with oral symptoms, nausea and vomiting after eating peanuts. Hence he developed a fear of all nuts and had been prescribed an adrenaline auto-injector. Current level of Ara h2 was >100 kU/L. He wanted to know if his fear of nuts was relevant and if there still was a need to always bring the adrenaline auto-injector.
  According to a strict protocol he ate three peanuts. Initially he experienced transient mild symptoms from the throat, followed by abdominal pain and flushing 10 min later. After another 10 min he developed hives and profuse vomiting. Antihistamine and cortisone was given intravenously, and the patient recovered quickly. He left the clinic after being observed for four hours.

**Conclusion**: Both patients expressed great relief to have undergone a peanut challenge. Knowing that eating small amounts of peanuts did not affect their ability to breathe or their circulatory functions has helped them replace the fear of what might happen into a relevant respect for peanuts. Oral challenge with peanuts in patients with peanut allergy could be justified in some patients, even with elevated levels of Ara h2, when it is important for the patient to know not only *that* he/she will react but also *how*.

**Consent to publish**: Patients have consented to the publication of this abstract.

### PP156 Pemetrexed anaphylaxis – An unusual suspect

#### Leonor Carneiro-Leão^1^, Vladyslava Barzylovych^2^, Júlio Oliveira^3^, Josefina Cernadas^1^

##### ^1^Serviço de Imunoalergologia, Centro Hospitalar de São João, Porto, Portugal; ^2^Drug Allergy Diagnostics Centre, IPOG of NAMS, Kiev, Ukraine; ^3^Clínica do Pulmão, Instituto Português de Oncologia do Porto, Porto, Portugal

**Correspondence**: Leonor Carneiro-Leão - leonorcarneiroleao@gmail.com

*Clinical and Translational Allergy* 2017, **7(Suppl 1)**:PP156

**Introduction**: Pemetrexed is a folate antimetabolite, approved for treatment of mesothelioma and non-squamous non-small cell lung cancer. It’s usually used in combination with platinum salts, gemcitabine or docetaxel. Pemetrexed presents a good safety profile, with myelosuppression being the most common dose-limiting toxicity. However, hypersensitivity reactions to pemetrexed have been described.

**Case report**: We report the case of a 52 year old woman, with stage IV metastatic adenocarcinoma of the lung, on palliative chemotherapy (QT) with pemetrexed plus carboplatin; for nausea control she was also receiving fosaprepitant immediately before QT. On her first treatment with these drugs, 3 min after starting pemetrexed perfusion, the patient developed dyspnea, cyanosis of the extremities, generalized pruritic exanthema and hypersudoresis. QT was immediately stopped and the patient was treated with hydrocortisone and clemastine. However, her status rapidly progressed to anaphylactic shock, with loss of consciousness, sphincters control, bradypnea, bradycardia, non-measurable blood pressure) and tachycardia. She received IV adrenaline and aggressive fluid resuscitation, with rapid improvement and was discharged 24 h later, fully recovered. Serum tryptase during the episode was elevated at 67.5ug/L (normal range: <11.4ug/L). The patient was referred to our drug allergy unit for study. Skin prick tests performed 6 months later were positive to pemetrexed 25 mg/ml (7 mm; histamine 10 mg/ml = 5 mm) and negative to carboplatin 10 mg/ml and fosaprepitant 1 mg/ml; intradermal tests were also positive to pemetrexed (1/1000) and negative to carboplatin (1/1000–1/10) and fosaprepitant (1/1000–1/10). As the oncologist decided for an alternative treatment, a desensitization protocol to the culprit drug, pemetrexed, was not considered.

**Conclusion**: Reports of hypersensitivity reactions to pemetrexed are very rare. This drug is almost exclusively used in combination with other chemotherapeutic agents, like platinum salts, which might underestimate pemetrexed as a cause of immediate hypersensitivity reactions. To our knowledge, this is the third report of documented anaphylaxis to pemetrexed, and the first to support the diagnosis of an IgE dependent mechanism through skin tests.

**Consent to publish**: Consent for publication was obtained.

### PP157 Barnacle allergy – Report of 2 cases

#### Leonor Carneiro-Leão, Diana Silva, Alice Coimbra

##### Serviço de imunoalergologia, Centro Hospitalar de São João, Porto, Portugal

**Correspondence**: Leonor Carneiro- Leão - leonorcarneiroleao@gmail.com

*Clinical and Translational Allergy* 2017, **7(Suppl 1)**:PP157

**Introduction**: Barnacles (*Pollicipes pollicipes),* a type of seafood found on sea coasts worldwide, are a delicacy consumed in Portugal, Spain, France and South America. Barnacle allergy is rare, with few published cases [1].

**Case report**: *Case 1:* A 4-year-old boy with allergic rhinitis, sensitized to pollens and house dust mites, reported 2 suspected food-related allergic reactions. In the first episode, he complained of pruritus and facial erythema immediately after eating barnacles and fish. The second episode occurred 6 months later and he presented with dyspnea, cough, urticaria and facial edema immediately after eating hake. He had a specific IgE to hake = 0.34 kU/L and positive skin prick-prick tests (SPPT) (raw hake: 7 mm; cooked hake: 5 mm; histamine: 4 mm) performed at another hospital. No further tests were conducted and he was instructed by his previous physician to avoid all fishes. A more detailed history at our out-patient clinic revealed that he continued to ingest and tolerate fish. Also, the parents were eating barnacles in the second episode and they could not guarantee the absence of cross contamination with the son’s food. One year after the last episode, SPPT with barnacle were performed and they were positive (raw meat: 13 mm, cooked meat: 13 mm, histamine: 4 mm). Parents refused oral provocation test (OPT). Comment: This case illustrates the importance of hidden allergens, cross contamination and a detailed history in order to establish a correct diagnosis and unnecessary dietary restrictions. *Case 2:* A 9-year-old boy, with allergic rhinitis undergoing allergen immunotherapy with *D. pteronyssinus*, was referred to our Allergy Department for an episode of dyspnea, nausea, and vomiting, oral and nasal pruritus immediately after eating 4 barnacles. He was rushed to the ER and successfully treated with corticosteroids, antihistamines and nebulized salbutamol. Four months later, SPPT were positive to cooked barnacle (meat: 7 mm; histamine 10 mg/ml: 5 mm); OPT with barnacle was not performed.

**Conclusion**: Barnacle allergy is a rare, but its allergenic potential is well established. Molecular allergens and cross-reactivity with mites have been described [1].

**Statement of consent**: Consent for publication was obtained.

**Reference**

1. Marinho S, et al. Barnacle allergy: allergen characterization and cross-reactivity with mites. J Investig Allergol ClinImmunol. 2006;16(2):117–22.

### PP158 Anaphylactic shock after contact with a betaine-polyhexanide antiseptic solution

#### Fabrícia Carolino, Josefina R. Cernadas

##### Serviço de Imunoalergologia, Centro Hospitalar São João, E.P.E., Porto, Portugal

**Correspondence**: Fabrícia Carolino - fabricia.c@sapo.pt

*Clinical and Translational Allergy* 2017, **7(Suppl 1)**:PP158

**Introduction**: Prontosan^®^ is an irrigation solution indicated for cleansing, moisturising and decontamination of acute and chronic wounds, and contains 0.1% undecylenamidopropyl betaine plus 0.1% polyaminopropyl biguanide (polyhexanide [PHMB]) in purified water.

**Case report**: We report the case of a 72-year-old woman, non-atopic, that developed manifestations of an anaphylactic shock, with generalized urticaria, stridor, dyspnoea, nausea, abdominal pain and severe hypotension in about 20 min after nurse treatment of a chronic vascular ulcer with Prontosan^®^ and petrolatum, at primary care; she was immediately medicated with hydrocortisone but no adrenaline and was transferred to the hospital emergency. The ulcer has been treated with the same antiseptic compound twice weekly before the described severe reaction although in the two previous treatments she presented with generalized urticaria, stridor and dyspnoea in 30 min after the ulcer care. Following the episode of anaphylactic shock, treatment did no longer include Prontosan^®^, and no more reactions occurred after that. The patient was referred to an Allergist for evaluation. A skin prick test with the implicated antiseptic was performed inducing a 6 × 5 mm wheal at 15-minute reading (identical to histamine 10 mg/ml); however, at 30 min a significant wheal increase was observed, to 14 × 10 mm. The patient’s daughter offered to be a control with a negative skin test result. At this point it was not possible to test each antiseptic component separately, but this will be the next step, along with an *in vitro* assay.

**Conclusion**: To the authors’ best knowledge this would be the forth case of anaphylaxis to a polyhexanide irrigation solution. No reports of severe immediate hypersensitivity reactions to topical betaine formulations were found in the literature.

**Consent to publish**: The authors state they have obtained patient’s informed oral consent for presentation and publication of the present clinical case.

### PP159 Galactose-α-1,3-galactose (alpha gal) allergy without anaphylaxis: a case report in Brazil

#### Renata R. Cocco, Luis F. Ensina, Carolina S. Aranda, Dirceu Solé

##### Federal University of São Paulo, São Paulo, Brazil

**Correspondence**: Renata R. Cocco - renata.cocco72@gmail.com

*Clinical and Translational Allergy* 2017, **7(Suppl 1)**:PP159

**Introduction**: Several studies suggest that tick bites are a cause of IgE antibody responses to alpha-gal in the United States, Europe, Asia and Australia. Delayed-onset reactions, especially anaphylaxis, have been reported to happen 3 to 6 h after ingestion of mammalian food products. Hereby we describe the first case of alpha gal allergy in Brazil in a male farmer, who presents with no other symptoms than urticaria.

**Case report**: A 55 year-old cattle breeder from North of Brazil (Rondonia) with no previous allergies refer to presenting with a daily scattered and pruritic papules for five years. Symptoms used to show up mostly in the late afternoon and were closely related to the ingestion of meat (lamb, bovine, pork, chicken) during lunch time. When questioned about tick bites, he clearly described multiple lesions in his body secondary to his job with animals and farm. Specific serum IgE revealed: bovine meat: 38.80 kU/L; pork meat: 28.60 kU/L; cow’s milk: 9.7 kU/L; serum bovine albumin: 1.51 kU/L; alpha gal: 70.7 kU/L; total IgE 888UI/mL. After orientation to restrict all meats, his symptoms have disappeared.

**Conclusions**: Although most of reports about alpha-gal allergy involve anaphylaxis, this patient presented only with urticaria. The only route of sensitization was through tick bites, since he never had contact to cetuximab. Chronic urticaria in farmers or environment with ticks, which can be very often in Brazil, should be investigated for alpha gal allergy.

**Consent to publish**: Patient consented to the publication of this abstract.

### PP160 Anaphylaxis to fenugreek

#### Leire Dopazo^1^, Rebeca Lopez^1^, Borja Bartolome^2^, Raquel Perez^1^, Laura Santos-Diez^1^, Agurtzane Bilbao^1^, Juan Miguel Garcia^1^

##### ^1^Hospital Universitario Cruces, Bilbao, Spain; ^2^R&D Department, Bial-Aristegui, Spain

**Correspondence**: Leire Dopazo - leiredop@hotmail.com

*Clinical and Translational Allergy* 2017, **7(Suppl 1)**:PP160

**Introduction**: Fenugreek (Trigonellafoenum-graecum), belonging to the Fabaceae plant family (legumes), has been used for culinary (as a spice in curry) and medical purposes since ancients times, especially in Greece, Egypt and India.

**Case report**: A 16 years old male, with previous anaphylaxis after intake peanuts, presented anaphylaxis after intake fenugreek. Skin prick test with extracts: Peanut 10 × 20 mm, natural Fenugreek 6 × 8 mm, Lentil 2 mm, Soy 2 mm, Bean negative, Histamine 5 mm. Serum specific IgE determinations (ImmunoCAP; *EAST): Peanut 84 kU/L (Ara h 1 34.5 kU/L, Ara h 2 60.9 kU/L, Ara h 3 2.41 kU/L, Ara h 8 0.03 kU/L and Ara h 9 0.05 kU/L), Fenugreek >17.5 kU/L*, Curry 16.9 kU/L, Lentil 1.67 kU/L, Soy 2.65 kU/L, Bean 0.04 kU/L. IgE-binding-protein molecular mass study (SDS-PAGE-Immunoblotting): Peanut extract (raw and toasted): 75, 63, 21 and 19 kDa. Fenugreek extract: IgE-binding bands between > 97–30, 21 and 19 kDa. Cross-reactivity study using SDS-PAGE-Immunobloting-inhibition with fenugreek extract as solid phase: Raw and toasted peanut extracts inhibit completely IgE binding to fenugreek extract. Study interpretation: 63 kDa-IgE binding band in peanut extract could correspond to 7S globulin Ara h 1. 21 kDa and 19 kDa detected bands in peanut extract could correspond to 2S albumin Ara h 2.01 and Ara h 2.02, bands of same molecular mass are observed in fenugreek extract. sIgE cross-reactive to fenugreek and peanut proteins are presents in patient’s serum.

**Conclusions**: We describe a case of fenugreek anaphylaxis. Cross-reactivity between fenugreek and peanut is demonstrated. We consider necessary to rule out fenugreek sensitization in peanut or other legumes allergic patients. Possibility of allergic reactions with curry in peanut and legumes allergic patients.

**Consent to publish**: Local institutional review board approved study procedures, and written informed consent was obtained. The authors declare that they have no relevant conflicts of interest.

### PP161 Rectal bleeding in a breastfed infant

#### Andreia Forno, Alexandra Rodrigues, António Jorge Cabral, Rute Gonçalves

##### Department of Pediatrics, Hospital Central do Funchal, Funchal Madeira, Portugal

**Correspondence**: Andreia Forno - aforno_@hotmail.com

*Clinical and Translational Allergy* 2017, **7(Suppl 1)**:PP161

**Introduction**: Rectal bleeding in infants can be a diagnostic challenge. In children younger than one year of age, anal fissures are the most common cause of rectal bleeding. However, anal fissures can hide and delay other rare diagnoses.

**Case report**: We report a twenty-four days old female admitted with rectal bleeding since she was two weeks old. There were no other symptoms, as fever, vomiting or diarrhea. The child was exclusively breastfed. The physical examination revealed an anal fissure at 6am in dorsal position, with no other changes on examination. The mother of the patient excluded dairy products of her diet, with no improvement of symptoms. The laboratory tests revealed peripheral blood eosinophilia (1.2 × 10^3^/μL eosinophils) with no anemia (Hb 10.3 g/dL) or other analytical alterations. No virus (rotavirus and adenovirus) was detected in stools and stool culture was negative. Colonoscopy showed discreet erythema of the sigmoid colon mucosa, without ulcers, fibrin or aphthous injuries. Biopsies revealed high number of eosinophils in the lamina propria of sigmoid colon. The patient started an extensively hydrolyzed cow’s milk formula, with symptomatic improvement. However, after four days, there was recurrence of the rectal bleeding, associated with exuberant perianal erythema. Since the rectal bleeding persisted, the patient started an amino acid-based formula. In just a few days, there was a symptomatic improvement. At six months of age the child gradually reintroduced cow’s milk formula, with tolerance. This clinical presentation, with the results of colonoscopy, is a striking feature of food protein-induced proctocolitis (FPIP).

**Conclusions**: FPIP is a rare cause of rectal bleeding in healthy infants, sometimes mistaken with anal fissures. It is characterized by inflammation of the distal colon due to a non-IgE-mediated reaction to food proteins, most commonly cow’s milk proteins. In exclusively breastfed infants, mother exclusion of cow’s milk and dairy products should be sufficient to stop the symptoms. However food restriction may be difficult to accomplish for mothers. Additionally there are a small number of infants that not respond to food restriction of their mothers or extensively hydrolyzed cow’s milk formula, being necessary to do an amino acid-based formula. This case report pretends to draw attention to a rare diagnosis of rectal bleeding in infants, where clinical suspicion is extremely important.

**Consent to publish**: Written informed consent to use the data was obtained for the patients.

### PP162 Fish anaphylaxis: case report and studies performed

#### Ignacio García Núñez^1^, María Ángeles Algaba Mármol^2^, María José Barasona Villarejo^3^, José Antonio Bácter Martos^2^, Marina Suárez Vergara^1^, José María Ignacio García^1^

##### ^1^Allergy and Pneumology Department, Hospital Quirón Campo de Gibraltar, Cádiz, Spain; ^2^DCCU Écija, Osuna Primary Care Unit, Seville, Spain; ^3^Allergy Department, Hospital Universitario Reina Sofía, Córdoba, Spain

**Correspondence**: Ignacio García Núñez - h62ganui@hotmail.com

*Clinical and Translational Allergy* 2017, **7(Suppl 1)**:PP162

**Introduction**: Every year food allergies are most frequent in Allergy deparments, being more typical to fruits, eggs, milk and fish. *Anisakis simplex* sensitization is very common at Mediterranean blue fishes, producing symptoms from digestive ones to anaphylaxis. Our aim is to show a patient with fish anaphylaxis, and the in vivo and in vitro studies we performed, and the obligation of performing a Food Provocation Test (FPT) with the culprit fish.

**Methods**: A 35 years-old patient came to our clinic referring arms urticaria with facial angioedema and disnea without hypotension 15 min after eating smoked salmon, needing adrenaline treatment. Good tolerance before this episode. We performed skin prick test with the main aeroallergens and panallergens, typical fishes and anisakis, and we studied basal tryptase, total IgE and specific IgE, and ISAC^®^ to evaluate a molecular diagnose.

**Results**: Skin prick test was negative to aeroallergens and fishes, and very positive to A*nisakis simplex*. Total IgE was 80 KU/L, being the basal tryptase 3,5. Specific IgE to *Anisakis* was 10,2, and negative to tuna and salmon, with a positive result using ISAC^®^ to Ani s1 and Ani s3. With these results, we performed a FPT with frozen salmon, being negative during 2 h of observation as an inpatient and during 48 h at home

**Conclusions**:

- We present a patient with moderate anaphylaxis after eating salmon, being the *Anisakis simplex* parasitation the main reason of it.
- A good clinical report and in vivo/in vitro studies are very important to demonstrate the etiology
- A FPT is a very good tool to improve our diagnosis and to avoid mistakes.

**Consent to publish**: The patient has consented to publication of our findings.

### PP163 Food-dependent exercise-induced anaphylaxis (FDEIA) – A case study

#### Ewa Gawronska-Ukleja, Agata Michalska, Natalia Ukleja-Sokolowska, Grzegorz Sergiejko, Magdalena Zbikowska-Gotz, Kinga Lis, Zbigniew Bartuzi, Robert Zacniewski

##### Department of Allergology, Clinical Immunology and Int. Diseases, L. Rydygier Collegium Medicum, Bydgoszcz NCU, Bydgoszcz, Poland

**Correspondence**: Ewa Gawronska-Ukleja - ntsia@go2.pl

*Clinical and Translational Allergy* 2017, **7(Suppl 1)**:PP163

**Introduction**: Food-dependent exercise-induced anaphylaxis (FDEIA) is a rare. Particularly important clinical problem is wheat-dependent exercise induced anaphylaxis (WDEIA), caused by sensitization to omega-5 gliadin.

**Case report**: Patient, 55, male, was admitted due to recurrent generalized urticaria, which often proceeded loss of consciousness. First episode of urticaria, followed by numbness of the lower limbs and syncope, appeared in 2007, without any apparent reason. Patient was hospitalized due to those symptoms. Next episode occurred in 2012—patient ate pizza, than performed physical exercise, during which he lost consciousness and due to those symptoms required hospitalization. Most recent episode of anaphylaxis occurred in 2015, also after physical exercise (shoveling coal). In general episodes of urticaria occur every 2–3 months. During diagnosis we performed skin prick tests (SPT) with inhalant and food allergen. We also established the level of total IgE, specific IgE to Dermatophagoides pteronyssinus, Dermatophagoides farinae, bee and wasp venom, gluten, wheat and rye flour and ImmunoCap ISAC. Patient also performed exercise provocation test on an empty stomach and after eating bread. SPT were positive with wheat flour (4/25 mm) and rye flour (4/5 mm). The concentration of allergen-specific IgE against wheat flour (before exercise provocation test) was 0.18 IU/ml and rye four 1.06 IU/ml, after provocation test respectively 0.18 IU/ml and 1.21 IU/ml. ImmunoCap ISAC reveled elevated level of IgE specific to wheat allergen component rTri a 19 (omega-5-gliadin)—2.4 ISU-E and to timothy nPhl p 4 (berberine bridge enzyme)—2,5 ISU-E. Exercise provocation test on an empty stomach—negative, with no disturbing symptoms. Exercise provocation test after consumption of bread: before the test blood pressure 120/70 mmHg, HR 74/min, immediately after the test (on a treadmill): 128/75 mmHg, HR 120/min. 10 min after the test patient experienced generalized urticaria and hypotension 95/70 mmHg. Patient was administered intravenous steroids, antihistamines and intravenous fluids with improvement.

**Conclusions**: Based on the clinical history of the disease, results of provocation test and immunological examination patient was diagnosed with wheat-dependent exercise induced anaphylaxis. The episodes of anaphylaxis could be prevented by avoidance of food (especially wheat and rye flour) ingestion in relation to exercise.

**Consent to publish**: Patient consented to the publication.

### PP164 A case of Food-Dependent Exercise Induced Anaphylaxis (FDEIA) – Cholinergic urticaria overlap syndrome in a 20-year-old football player

#### Ileana-Maria Ghiordanescu^1^, Cristina Deaconu^2^, Mihaela Popescu^1^, Roxana Silvia Bumbacea^1^

##### ^1^Elias Emergency University Hospital, Bucharest, Romania; ^2^Novo Medica Clinic, Bucharest, Romania

**Correspondence**: Ileana-Maria Ghiordanescu - ileana.ghiordanescu@gmail.com

*Clinical and Translational Allergy* 2017, **7(Suppl 1)**:PP164

**Introduction**: Exercise-induced anaphylaxis (EIA) is a rare disorder in which anaphylaxis occurs after physical exercise. In a subset of exercise-induced anaphylaxis, named food-dependent exercise-induced anaphylaxis (FDEIA), anaphylaxis only developes if exercise is performed within a few hours after eating a specific food. Cofactors, other than food are also described. Primarily differential diagnosis is made with cholinergic urticaria, a micropapular type of urticaria. To complicate things exercise induced anaphylaxis can sometimes aggravate pre—existing cholinergic or cold urticaria and there is also a variant—type of FDEIA that resembles cholinergic urticaria and shares pathogenic mechanisms with it.

**Case report**: We present the case of a 20 years old football player who experienced six episodes of acute urticaria in the last 6 months prior to the presentation. Most episodes where related to phisical exercise (jogging) but he also reported an episode with intens pruritus and micropapular urticaria during sauna bathing. Intrerestingly he described pruritus that developed every time he went running (daily). The last episode showed different clinical aspect, associated tachicardia, dyspnea and colicky pain and occured while exercising (jogging), 6 h after eating pizza. A suspicion of exercise—induced anaphylaxis with possible associated food co-factor was raised. Paraclinical work up showed high levels of Total Ig E (440 IU/mL; cut off limit 100 IU/mL and raised levels of omega 5 gliadin (FEIA: 3.33 kU/L, for a cut off value of 0.35 kU/L). A diagnostic of FDEIA, in an atopic patient was made. The patient was adviced to avoid eating food that contained gluten and an emergency kit was prescribed. During the follow up period the patient did not experience other anaphylactic episodes although he continued to report mainly pruritus after jogging occasionally associated with minor micropapular urticaria. An effort test was performed.

**Conclusions**: In the present case, although a diagnostic of FDEIA was made guided by the presence of raised omega 5 gliadin sIg E and complete avoidance of gluten protected against anaphylaxis, the patient continued to experience pruritus and sometimes mild eruption during jogging—suggesting a FDEIA—cholinergic urticaria overlap syndrome.

**Consent to publish**: Informed constent for presentation and publication was obtained.

### PP165 Fish induced anaphylaxis with different underlying causes and mechanisms

#### Alkerta Ibranji^1^, Elida Nikolla^2^, Gjustina Loloci^3^

##### ^1^Catholic University “Our Lady of Good Counsel”, Tirana, Albania; ^2^Regional Hospital, Saranda, Albania; ^3^“Mother Theresa” School of Medicine, Tirana, Albania

**Correspondence**: Alkerta Ibranji - alkertaibranji@gmail.com

*Clinical and Translational Allergy* 2017, **7(Suppl 1)**:PP165

**Introduction**: Anaphylaxis is a severe, potentially life-threatening systemic hypersensitivity reaction. Foods are by far the most common anaphylaxis trigger in infants, children, teens, and young adults. De novo food (fish and shellfish) allergy is most common within young adult population. Despite the mechanism involved for anaphylaxis elicitation, adrenaline is the first line of treatment and an action plan/management plan should be instructed to the patients or their care givers.

**Case report**: We report three anaphylaxis cases presented in our clinic during 2014–2016, all initially referred by the ED professionals as fish induced anaphylaxis. They all had none co-morbidities and no previous allergic reactions of any kind.

- 23 years old female patient developed generalised rush and urticaria, angioedema of the lips, difficulty with swallowing and breathing, abdominal cramps about 40 min after cod-fish ingestion.
- 27 years old female patient developed angioedema of the lips, generalised urticaria, difficulty with breathing and swallowing about10 min after fish ingestion.
- 24 years old female patient developed urticaria and angioedema, abdominal cramps, mild breathing difficulty about 20 min after fish ingestion.
- Biochemical and full blood count resulted normal. Mast cell disorder was excluded (Table 5).

**Table 5:** See text for description

	Symptoms	Onset after ingestion	Skin prick test sIgE for food panel	Co-factors	Diagnosis
Patient 1	Airway, cutaneous, GI	40 min	Negative/Negative	None	Scombroid
Patient 2	Airway, cutaneous, GI	10 min	Negative/Positive sIgE	Iduprofen 20 min before	Probable Codfish Allergy Induced by NSAIDS
Patient 3	Airway, cutaneous	20 min	Positive for CodFish/Positive for sIgE	None	Codfish Allergy

Epipen was prescribed to patient 2 and 3 in case of future potential anaphylaxis and a written anaphylaxis action plan

**Conclusions**:

- Scombroid food poisoning and fish allergy are often confused interchangeably and in vivo and in vitro allergy tests as well as a careful history are needed to make the right diagnosis.
- The role of co-factors is important in eliciting anaphylaxis.
- De novo food allergy related to fish and shellfish in adults should always be investigated by the allergist.

**Consent to publish**: Written informed consent was obtained from the patient’s parent or guardian for publication of this abstract and any accompanying images. A copy of the written consent is available for review by the Editor of this journal.

### PP166 Clinical allergy induced by repeated challenges or by exposure to a cross-reactive food?

#### Nanna Juel-Berg^1^, Kirsten Skamstrup Hansen^1,2^, Lau Fabricius Larsen^1^, Lars Kjaergaard Poulsen^1^

##### ^1^Allergy Clinic, Copenhagen University Hospital, Gentofte, Denmark; ^2^Department of Pediatrics, Herlev, Denmark

**Correspondence**: Nanna Juel-Berg - njuelberg@gmail.com

*Clinical and Translational Allergy* 2017, **7(Suppl 1)**:PP166

**Introduction**: Sequence homology in IgE binding epitopes of proteins from different foods may cause cross-sensitivity and hereby also clinical cross-reactivity. A recent study investigating allergy towards pistachio nut and cashew nut found that 25/25 patients were double-allergic to the nuts. The levels of specific IgE (sIgE) may increase with age and new allergies may occur towards previously tolerated foods. Sensitization, on the contrary, can be present though the patient is tolerant to the food item, why diagnosis relies on oral food challenges (OFCs). A study has shown that up to 13% of OFCs can be negative, with a clinical reaction upon re-exposure. This can be due to priming or maturation of the immune system. But the course of events leading to reactivity or cross-reactivity towards a previously tolerated food item is not fully clarified.

**Fig. 8:**
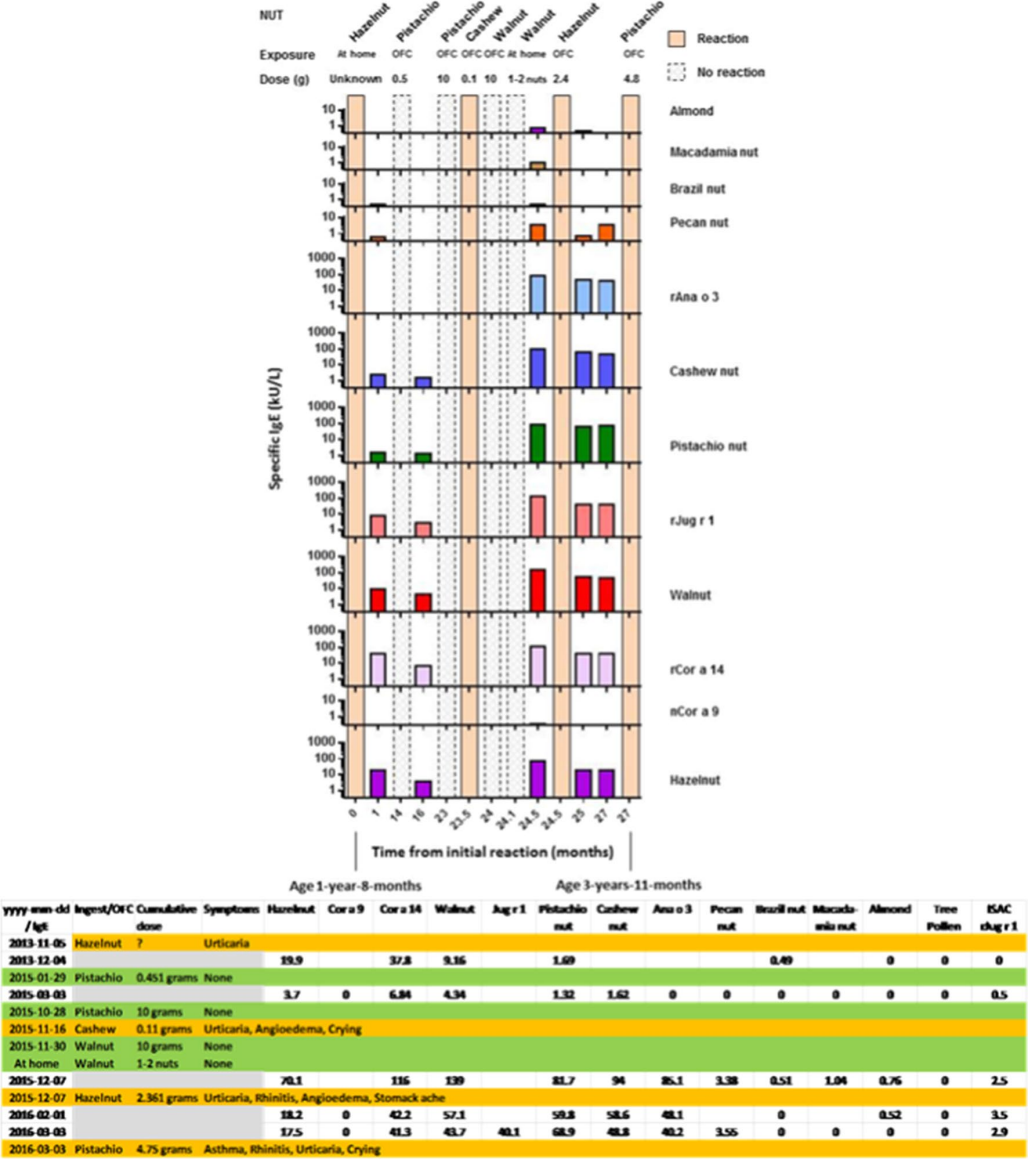
Summary of clinical and serological testing. OFCs were terminated at appearance objective symptoms or after tolerating 10 grams (g) of nut (the first pistachio-OFC was stopped at 0.45 g, due to time-shortage). IgE (detection limit ≥0.35 kU/L) towards all listed nuts and components were measured on each blood sample. IgE is depicted on a logarithmic scale

**Case report**: A 1-year-8-months-old girl was referred to an allergy center after an allergic reaction to hazelnuts. She had sIgE (Thermo Fisher Scientific, Uppsala, Sweden) towards other tree nuts that she had never ingested, and kept a nut-free diet until subsequent OFCs (Fig. 8). After two negative pistachio-OFCs, one positive cashew-OFC and one negative walnut-OFC, a 20 fold increase in sIgE was observed towards all nuts challenged with including hazelnuts (later hazelnut-OFC was positive). Regular intake of the food tolerated in an OFC is normally recommended. But due to the increase in sIgE it was regarded unsafe. When re-challenged with pistachio nut, the patient reacted with grade-2-anaphylaxis (asthma, rhinitis and urticaria) to a dose much lower than the dose previously tolerated. Due to safety and ethical reasons the patient was not re-challenged with walnuts.

**Conclusions**: The report suggests that allergy or cross-allergy towards pistachio nut may be induced by repeated exposure. It also stresses the importance of IgE testing close to the date before and shortly after an OFC. The natural course of allergic disease should be taken into consideration. But the timing of OFCs could be of importance to the challenge outcome.

**Consent to publish**: A written consent for presentation and publication is obtained from the parents.

### PP168 Suicide attempt by food anaphylaxis – Case report

#### João Marcelino^1^, Ricardo Prata^2^, Ana Célia Costa^1^, Manuel Branco Ferreira^1^, Fátima Duarte^1^, Marta Neto^1^, Jennifer Santos^2^, Luís Câmara Pestana^2^, Daniel Sampaio^2^, Manuel Pereira-Barbosa^1^

##### ^1^Immunoallergology Universitary Department, Santa Maria Hospital, Lisbon Medical Academic Center, Lisbon, Portugal; ^2^Psychiatric Department, Santa Maria Hospital, Centro Hospitalar Lisboa Norte, Lisbon, Portugal

**Correspondence**: João Marcelino - jlam_1987@sapo.pt

*Clinical and Translational Allergy* 2017, **7(Suppl 1)**:PP168

**Introduction**: Anaphylaxis is a severe, potentially life-threatening systemic hypersensitivity reaction, being a medical emergency. Trigger identification, patient education and establishing emergency action plans are essential steps in the management of this condition. However, in rare cases, patients deliberately expose themselves to the trigger to attempt self-harm.

**Case report**: We report a case of a 39-year-old woman, with a history of multiple sclerosis, borderline personality disorder, depression (with 10 previous emergency department visits with suicidal thoughts) and documented hypersensitivity to nuts and fresh fruits (rosaceae). Following a fifth discharge from a Psychiatric Department, she stopped taking her psychiatric medication, had a worsening of her depression and ingested peach juice as a suicide attempt. Within minutes, she developed generalized pruritus, hand edema, wheezing and desaturation. She was rushed to the Emergency Department where she was promptly medicated with im adrenaline, iv corticosteroids, iv anti-histamines and inhaled bronchodilators, which stabilized the patient and reversed the anaphylactic reaction. In the emergency room she confessed she had taken the juice as a suicide attempt. During the first three days she remained dependent on oxygen and inhaled bronchodilators to prevent desaturation. On the seventh day, completely recovered from her anaphylaxis, she was transferred to the Psychiatric Department for further treatment.

**Conclusions**: Food allergens account for a large part of anaphylaxis triggers. However, their intentional use is very uncommon. Suicide attempts by means of anaphylaxis are rare; however, they occur and should be documented. This documentation is essential, so the true prevalence of the problem can be known. Immunoalergologists and Psychiatrists following patients with depression and a history of anaphylactic reaction should be aware of this possibility and have a more stringent follow-up.

**Consent to publish**: All case details or other personal information presented in this case report are published with the consent of the patient.

### PP169 Alfa-gal syndrome: five new Italian cases

#### Paola Minale^1^, Paola Dignetti^2^, Donatella Bignardi^1^

##### ^1^IRCSS San Martino, IST Genova UOC Allergologia, Genova, Italy; ^2^Emergency Department, San Paolo Hospital Savona, Savona, Italy

**Correspondence**: Paola Minale - paola.minale@hsanmartino.it

*Clinical and Translational Allergy* 2017, **7(Suppl 1)**:PP169

**Introduction**: Alfa -Gal syndrome, an IgE-mediated immediate-type allergy to the disaccharide galactose–1,3-galactose is relevant as food, drugs, and tick bites allergy. The majority of cases have been found in the southeast area of the United States, but their occurrence in Europe is nonetheless so frequent as to be recognized as a new challenge for allergyst.

**Case report**: We present clinical characteristics of 5 Italian Patients with a history of reactions (rash, itching, urticaria) of typically delayed onset following consumption of pork, beef, lamb meat and/or innard. An association with tick bites (Ixodes ricinus) was confirmed in all subjects. As the phenomenon of a delayed-onset immediate-type allergy with a latency of 3–6 h following ingestion of mammalian meat is considered pathognomonic for alfa-Gal syndrome, a Prick-to-prick tests using fresh meat (pork, beef, lamb) and kidney preparations was performed in all Patients. Five Patients showed positive results to pork and beef meat, two of them also to lamb meat. Allergen-specific IgE was measured by ImmunoCAP^®^ alpha-Gal; all patients were sensitized to alpha-Gal (range 2.2–124 kUA/l). All Patients were male, three of them with job exposition to tick bites (1 veterinary, 2 forest guardian), two of them exposed only during holiday on open air or hunting. Interestingly, the veterinary experienced a systemic reaction during cow labor, possibly for cutaneous contact with vagina and placenta.

**Conclusions**: Epidemiological studies put the prevalence of alfa-Gal sensitization in Europe at 5.5–8.1% (29), but sensitization rates vary considerably from region to region. As geographically local phenomena may be helpful in our understanding of alfa-gal syndrome, studies on the prevalence are ongoing in Ligurian Region, extending to the populations exposed to tick bites to recognize new cases.

**Consent to publish**: We consent for presentation and publication.

### PP170 Anaphylaxis to fish vapors – Case report

#### Irena Nedelea, Diana Deleanu

##### UMF CLUJ, Cluj-Napoca, Romania

**Correspondence**: Irena Nedelea - irena.manea@gmail.com

*Clinical and Translational Allergy* 2017, **7(Suppl 1)**:PP170

**Introduction**: Food allergy accounts for a significant social and economic impact worldwide. An increasing number of severe life-threatening reactions have been linked to food allergies. Anaphylactic reactions to foods are frequently attributed to exposure to fish allergens, mainly in the adult population, and respiratory symptoms occur in approximately 50% of fish allergic patients. Prevalence rates of self-reported fish allergy reaches 8% among fish processing workers.

**Case report**: We report the case of a 47-year-old male patient, with a personal history of atopy (sensitization to Dermatophagoides farinae and Dermatophagoides pternonyssinus), known to suffer from asthma and moderate/severe persistent allergic rhinitis, working as a chef assistant, who refers to the Allergy Service of the Regional Institute of Gastroenterology and Hepatology “Professor Doctor Octavian Fodor”, Cluj-Napoca, Romania, with a history of asthma exacerbations and one anaphylactic episode triggered by occupational exposure to fish vapors. The clinical history reveals contact urticaria to uncooked fish, and two episodes of facial angioedema and acute generalized urticaria after ingestion to lobster and salmon, respectively. Further clinical and paraclinical workup confirmed the atopic diathesis. In vivo sensitization to Der f and Der p, as well as to fish (salmon, tuna, merlucius, herring, and fish mix) was documented.

**Conclusions**: Hypersensitivity reactions to fish present with a wide range of clinical symptoms. In this paper, we aim to highlight the interrelation between asthma and food allergies, by presenting the case of a young male patient with asthma exacerbations and a systemic life-threatening hypersensitivity reaction to fish vapors, as well as contact urticaria after handling uncooked fish, and systemic hypersensitivity reactions triggered by ingestion of salmon and lobster.

**Consent to publish**: With the submission of this abstract, I would like to undertake that the authors give their consent for publication and presentation.

### PP174 IgE sensitisation to Bet v 1 in a birch pollen- allergic adult patient with oral allergy syndrome to hazelnuts, Rosaceae fruits and soy drink

#### Florin-Dan Popescu^1^, Mariana Vieru^1^, Florin-Adrian Secureanu^2^, Carmen Saviana Ganea^2^

##### ^1^Carol Davila University of Medicine and Pharmacy, Bucharest, Romania; ^2^Nicolae Malaxa Clinical Hospital, Bucharest, Romania

**Correspondence**: Florin-Dan Popescu - florindanpopescu@allergist.com

*Clinical and Translational Allergy* 2017, **7(Suppl 1)**:PP174

**Introduction**: Patients from Southern Romania with allergy to birch pollen sometimes report oral allergy syndrome, a contact urticaria of the oropharyngeal sites, to *Rosaceae* fruits and hazelnuts, but no report of soy allergy in these cases was published.

**Case report**: We present a 21-year-old female patient with seasonal allergic rhinoconjunctivitis in spring and convincing history of oral allergy syndrome to hazelnuts, fresh *Rosaceae* fruits (apple, pear, quince, peach, apricot, plum, sweet cherries, strawberries), and soymilk. The skin prick testing was performed with commercial extracts, the prick-prick testing with some fresh edible *Rosaceae* fruits, serum specific IgE sensitization profile was assessed by line blot test system to native aeroallergen extracts and cross-reactive foods, while a multi-parameter line blot test system was used for pollen defined partial allergen diagnosis. The patient presented positive skin prick tests to birch (6 mm wheal) and hazel (7 mm wheal) pollen commercial extracts, negative to soy extract, and positive prick-prick tests with fresh fruits: apple (4 mm wheal), pear and peach (each 3 mm wheal). Serum specific IgE levels were found increased for birch pollen (72 kU/L, EAST class 5), hazel pollen (21 kU/L, EAST class 4), alder pollen (25 kU/L, EAST class 4), but also, to a lesser extent, for hazelnut (0.43 kU/L, EAST class 1). Specific IgE antibodies to apple, apricot, strawberries, celery and carrot were not found (<0.35 kU/L). IgE sensitization profile to recombinant allergen components revealed sensitization to rBet v 1 (65 kU/L, EAST class 5), while specific IgE to profilin biomarker rBet v 2, polcalcin biomarker rBet v 4 and isoflavone reductase rBet v 6 were not detected (<0.35 kU/L). Serum IgE level to cross-reactive carbohydrate determinant marker was below detection (<0.35 kU/L).

**Conclusions**: A multiplex line blot assessment of recombinant *Betula* pollen allergen components can be used in clinical practice to determine whether IgE sensitization to Bet v 1 in patients with birch pollen allergy is involved in cross-reactivity reactions with foods containing Bet v 1-like allergen components: *Rosaceae* fruits (apple, pear, peach, apricot, plum, cherry, strawberry, raspberry), exotic fruits (kiwi, persimmon, Jack fruit), *Betulaceae* (hazelnut) and *Rosaceae* (almond) nuts, *Apiaceae* vegetables (celery, carrot), *Fabaceae* legumes (peanut, soybean, chickpea) and tomato.

**Consent to publish**: Written informed consent was obtained for presentation and publication.

### PP176 Recurrent anaphylaxis in shrimp and cocktail-tomato allergic patient

#### Natalia Ukleja-Sokolowska^1^, Ewa Gawronska-Ukleja^1^, Magdalena Zbikowska-Gotz^1^, Zbigniew Bartuzi^1^, Lukasz Sokolowski^2^

##### ^1^Department of Allergology, Clinical Immunology and Internal Diseases, L. Rydygier Collegium Medicum, Bydgoszcz NCU, Bydgoszcz, Poland; ^2^Division of Ergonomics and Exercise Physiology, L. Rydygier Collegium Medicum, Bydgoszcz NCU, Bydgoszcz, Poland

**Correspondence**: Natalia Ukleja-Sokolowska - ukleja@10 g.pl

*Clinical and Translational Allergy* 2017, **7(Suppl 1)**:PP176

**Introduction**: Most important tomato allergens are: profilin Lyc e 1 and nsLTP Lyc e 3. López-Matas et al. [2011] analyzed the allergen content of 6 varieties of tomatoes. It was found that there are significant differences between the concentrations of allergens depending on the variety of tomato. Pen m 4 is calcium-binding protein is a minor allergen, sensitizing only 10–15% of Italian shrimp-allergic patients, but it is clinically relevant and may be the cause of anaphylaxis.

**Case report**: Patient, 25, female, in 2013 after consumption of shrimp, experienced swelling of the larynx, shortness of breath and generalized urticaria. Similar episodes occurred in May 2014 and June 2014, also due to shrimps. In March 2015 patient experienced angioedema of lips after consumption of cocktail tomatoes in a salad before physical activity (visit to the gym). She ate cocktail tomatoes before and afterwards without any symptoms. During diagnosis SPT were positive with allergens of cat, shrimp, hazel, alder, birch and Artemisia vulgaris. We performed prick by prick tests with fresh shrimp (positive 13/23 mm), slicing tomato (0/0) and cocktail tomato (4/4). In 5 min after prick by prick tests patient experienced angioedema of lips and itchiness and swelling of hands and feet. She was administered steroids with good clinical effect. Open-food challenge with cocktail tomato was negative. Afterwards exercise provocation test was performed**—**without any immediate allergy symptoms. Within 4 h patient experienced severe angioedema of lips, which was treated with systemic steroids. ImmunoCap ISAC reveled elevated level of IgE specific to shrimps calcium-binding protein nPen m 4 (4,3 ISU-E), birch PR-10 rBet v 1 (1,2 ISU-E), Artemisia defensin Art v 1 (1,2 ISU-E), cat Fel d 1 (20 ISU-E) and Fel d 4 (4,4 ISU-E).

**Conclusions**: Patient was diagnosed with exercise induced allergy to cocktail tomato. ImmunoCap ISAC found elevated level of IgE specific to cat allergens and birch allergens. Patient had anaphylactic reaction due to allergy to shrimps calcium-binding protein nPen m 4, which is a minor allergen. In case of anaphylaxis this patient has to carry a rescue set, including antihistamines, oral steroids and adrenaline in an auto-injector.

**Consent to publish**: Patient consented to the publication of this case report.

### PP177 Occupational seafood allergy in a chef: a case report

#### Miguel Vieira, Ana Reis Ferreira, José Pedro Moreira Silva

##### Immunoallergology Department - Centro Hospitalar de Vila Nova de Gaia/Espinho (CHVNG/E), Vila Nova de Gaia, Portugal

**Correspondence**: Miguel Vieira - miguel.santos.vieira@gmail.com

*Clinical and Translational Allergy* 2017, **7(Suppl 1)**:PP177

**Introduction**: Seafood plays an important role in nutrition and world economy, and nowadays it is a common cause of food allergy. Although reactions to seafood have been documented mainly among consumers, immune-mediated reactions have also been reported at work, and Chefs are among the most exposed occupational groups.

**Case report**: A 31 years old male, who is working as a Chef on a cruise ship, was referred to our Allergy Dept for a 6 months history of hand dermatitis initially, and later located hives after handling seafood at work. Three months after the symptoms began, he experienced 2 episodes of anaphylaxis minutes after the ingestion of a small portion of shellfish and sea bass, and after the ingestion of codfish with shellfish sauce (angioedema, stridor, dyspnea, conjunctivitis, nausea, vomiting and diarrhea). The allergic reactions subsided at home after the administration of oral hidroxizine. Skin symptoms started 9 years after working as a cook, exacerbated during his workdays and he got temporary relief during absence of work. Skin prick test with common inhalants were positive to house dust mites, *parietaria judaica* pollen and to a fish allergen panel (rooster fish, monkfish, sea bass, salmon, sardine, tuna, trout, mullet, hake and sole); Prick-prick were positive to raw and boiled shrimp, hake, salmon, sea bass and raw cod. Patch tests were positive to raw shrimp, hake and raw and boiled sea bass. Specific IgEs (UK/l) were positive to cod (5.27), salmon (3.92), tuna fish (2.47), squid (1.16), sardine (3.78), shrimp (22.30) and cod parvalbumin rGad c1 (5.37). Patient was advised to avoid contact with seafood and always carry an emergency kit, with an epinephrine autoinjector in order to prevent lethal outcome if accidental exposure occurs.

**Conclusions**: Occupational exposure to seafood can be an important seafood allergy trigger, by other means than gastrointestinal exposure. Allergic contact dermatitis to seafood is a clinically relevant condition that should be included in the differential diagnosis of chronic dermatitis affecting the hands or forearms of patients at high occupational risk. Disruption of the skin barrier seems to be an important added risk factor to the development of IgE mediated food allergy.

**Consent to publish**: Written informed consent was obtained for publication of this abstract.

### PP178 A rare case of food-induced urticaria to almond butter with tolerance to almond nuts

#### Timothy Watts^1^, Sophia Watts^2^

##### ^1^Guy’s Hospital, London, United Kingdom; ^2^East Surrey Hospital, Redhill, United Kingdom

**Correspondence**: Timothy Watts - timwatts1984@yahoo.co.uk

*Clinical and Translational Allergy* 2017, **7(Suppl 1)**:PP178

**Introduction**: Tree nut allergy is one of the most well recognised and investigated food allergies. Skin prick, Specific IgE (with components) and oral challenge tests are frequently used to evaluate and eventually confirm nut allergy with high diagnostic certainty. There is a growing recognition however, of atypical unexpected allergy to foods despite reassuring investigations and diagnostic challenges.

**Case report**: We report a case of a 23 year old male with previously diagnosed childhood primary peanut and sesame seed allergy. He had generally avoided all tree nuts since but reports oral itching symptoms on occasion when accidentally consuming almond. We investigated his almond allergy status. Almond skin prick test was 4 mm (positive) and specific IgE was 0.88 kU/L (positive); however the birch pollen Bet v1 (PR10) component was >100 kU/L consistent with oral allergy syndrome. We proceeded to an oral challenge with almond nuts where he received over 15 g in a supervised graded protocol and did not demonstrate any immediate reaction; effectively ruling out primary or IgE mediated allergy. However, he subsequently consumed almond butter at home with a quantity of around 5 g and developed immediate generalised urticaria within 5 min of consumption. No other allergenic ingredients were identified in the almond butter and he was advised to avoid the almond butter but continue consuming the whole nuts.

**Conclusions**: Despite demonstrating tolerance to whole almond nuts in a supervised challenge this patient unexpectedly reacted to almond butter with features of true IgE-mediated immediate hypersensitivity. There is limited literature on these type of atypical reactions. It is hypothesised that the oils released in the cooking process of making almond butter contain a highly concentrated allergenic epitope that was previously hidden. It is advised to consider atypical reactions to nuts even if deemed safe, especially when in modified forms such as butter or oil.

**Consent to publish**: The authors confirm that consent has been given for presentation and publication.

### PP180 Clinical case of food allergy formation on the background of Staph infection

#### Svitlana Zubchenko, Marta Lomikovska

##### Department of Clinical Immunology and Allergology of Danylo Halytsky, Lviv National Medical University, Lviv, Ukraine

**Correspondence**: Svitlana Zubchenko - maruniak.stepan@gmail.com

*Clinical and Translational Allergy* 2017, **7(Suppl 1)**:PP180

**Introduction**: General and specific researches do not always enable the accurate diagnosis of alimentary allergic causes.Sometimes we need to perform the additional bacteriological researches.

**Case report**: On consultation appointment we had a girl 10 year’s oldwith manifestations of allergic dermatitis, which arose 6 months ago and was associated with the consumption of various foods (as parents said). We know that another relative has no allegropathology. After helminthiasis have been treated and stayed on the elimination diet withthe exclusion of certain products led to the brief improvement. Last 6 months she is suffering on constipation. Objectively: on the face, thorax and extremities is patchy erythema, exfoliative displays, cracks, which accompanied with itching. Laboratory: in blood we see the absolute eosinophil (2.3 g/l), general serum IgE-1980 Iu/ml, increased levels of specific Ig E (ELISA > 0.35 kU/I) to all vegetables, fruits, nuts and cereals. The results of component diagnostics (ImmunoCAP, Phadia) (Pru p3 rBet v1, rBet v2, rBet v4, nART v3, Tri a19, rPhl p7, rPhl p12) are negative. The bacteriological research of skin, throat and feces- a large number of S. Aureus (10^8^). The specific IgE to the Staph enterotoxin B, C, TSST are increased (ImmunoCAP, Phadia). We verified the diagnosis: Chronic IgE-dependent urticaria, sensitization to Staphyloccocal toxin, the food allergy. Recommendation: the antistaphylococcal immunoglobulin with transition to the antistaphylococcal lysate, antihistamines.

**Conclusions**: The Staphylococcal toxin can cause polyvalent sensitization to food allergens, which requires phased diagnostic and specific antistaphylococcal treatment.

**Consent to publish**: Consent for publication was obtained.

### PP181 Specific aspects of the pollen-related food allergy during ASIT

#### Marina Peredelskaya^1^, Natalia Nenasheva^2^

##### ^1^State Hospital 52, Kursk, Russia; ^2^RMAPO, Moscow, Russia

**Correspondence**: Marina Peredelskaya - concy1984@gmail.com

*Clinical and Translational Allergy* 2017, **7(Suppl 1)**:PP181

**Introduction**: A male patient 27 years old applied to the clinic of allergology with complaints of stuffiness in nose, ocular itching, rhinorrhea, episodes of paroxysmal dry cough with wheezing in the chest, difficulty breathing out, chest tightness in the spring time (April and May).

**Case report**: From the medical history: symptoms of rhinitis and conjunctivitis developed in the age of 17 years and returned every year in the spring. Three years later the patient described itching in the mouth cavity after apples intake; three years later similar symptoms started to appear after pears, cherries, peaches intake, not only in the pollen season but all year round. Nuts intake began to provoke swelling of lips, feeling of a lump in the throat and a hoarseness of voice. At the age of 25, in spring, in the setting of rhinitis and conjunctivitis symptoms the marked episodes of difficulty breathing appeared; the patient sought medical advice from the allergologist; the diagnosis of bronchial asthma was established. The patient was given a treatment with bronchial spasmolytics, antihistamines, intranasal corticosteroids during the pollen season, with a positive result. An allergy testing was accomplished, that revealed sensitivities to tree pollen (birch, alder, hazel, and oak). ASIT was recommended.

In the first year of treatment the injections of repository tree pollen allergen (Fostal, Stalallengenes), was administrated, once a week. The preparatory course of therapy was sufficiently well tolerated; after a maintaining dose has been attained, the patient began to observe an infiltrate, hyperemia and pruritis at the injection site appearing on the third day after injection; all the symptoms were reversed within 24 h without treatment. To the end of the second month of maintaining therapy the patient transgressed his elimination diet: he ate an apple, but without any symptoms appearing. However two days after that diet transgression a hard itching in the perianal region appeared. Starting from that point the patient began to observe a pruritis in the perianal region on the third day after each injection of allergen. Topic antihistamine creams were administrated and antihistamine tablets; that treatment gave a positive effect. Dermatological and proctologic evaluation did not reveal any abnormality, as well as examination for helminthic invasion. A detailed inquiry showed that the diet transgression did not provoke any abdominal pain or borborygmus or any altered defecation pattern. The patient has a regular daily stool, does not report any diarrhea or constipation symptoms. EGDS did not reveal any abnormality. After the first year of the ASIT injections a positive trend in the course of disease was noted, with no need in bronchial spasmolitics and intranasal corticosteroids in pollen seasons, reduced need in antihistamines. However the patient reported itching in the perianal region in the pollen season, with the most marked symptoms on the 3rd day after the transgression of elimination diet. For the second course of the ASIT a sublingual drug administration way were chosen, a sublingual birch pollen allergen (Staloral, Stallergenes). The preparatory period of ASIT is well tolerated without any adverse side events. During the period of maintaining therapy episodes were documented of swelling in the region of lips and tong, as well as hyperemia in the infrahyoid lobe, that cut off without treatment 15–30 min later. The patient occasionally transgressed his elimination diet and noted delayed (on the 3rd day) symptoms of perianal itching; however, with the sublingual ASIT they gradually improved.

**Conclusion**: Symptoms of perianal pruritis are considered to be an manifestations of the polyvalent pollen-related food allergy to raw fruits (apples an and others) that first appeared during the injection ASIT therapy in a patient suffering with the pollen allergy to birch pollen and then reduced during his second course of the ASIT with the sublingual birch allergen.

**Consent to publish**: Written informed consent was obtained for publication of this abstract.

### PP185 A case report of high risk atopic children with positive family history of anaphylactic reaction to buckwheat

#### Ivana Filipovic^1^, Zorica Zivkovic^2^, Djordje Filipovic^3^

##### ^1^Faculty of Medical Science, Kragujevac, Serbia; ^2^Children’s Hospital for Lung Diseases and Tuberculosis, Medical Center “Dr Dragiša Mišović”, Belgrade, Serbia; ^3^Belgrade Institute for Emergency Medicine, Belgrade, Serbia

**Correspondence**: Ivana Filipovic - drivanica@yahoo.com

*Clinical and Translational Allergy* 2017, **7(Suppl 1)**:PP185

**Introduction**: Buckwheat, which has been abundantly consumed in Asian countries and has been increasingly popular in the United States, Canada, and Europe, can be a potent allergen when ingested or inhaled. Common buckwheat (*Fagopyrum esculentum*) is known to cause severe anaphylactic reactions in adult individuals. However, type I allergy to buckwheat is rarely seen in children.

**Case report**: Our case is reported a 45-year-old man developed life-threatening anaphylaxis after eating food containing buckwheat on 3 different occasions. He had tolerated all kinds of wheat for many years. Few minutes after eating buckwheat home-baked bread experienced urticaria, dyspnoea, wheezing, as well as symptoms of gastrointestinal tract nausea and vomiting. After circulatory collapse he admitted to the ER requiring restitution. It is interesting to mention that the third case of anaphylaxis was after intake of buckwheat flour as the hidden allergen in pastry. Prior to the first episode of anaphylaxis he has a positive history of respiratory allergies (asthma and allergic rhinitis) as well as positivity to multiple inhalator allergens (mixture of different grass pollen, ragweed, and tree pollens). Skin testing by the prick technique revealed positive reaction to buckwheat with negative reactions to other foods including wheat, egg white, and milk. A prick-to-prick test with buckwheat flour was also positive. His daughter suffered from the respiratory allergies (asthma and allergic rhinitis), atopic dermatitis since birth and complains of mouth itching while consuming hazelnut and walnuts. Her *in vitro* tests were positive to alpha lactalbumine, casein, peanuts, acarus, dogs’ and cats’ hairs, horse’s and cow’s epithelium as well as to the rodent epithelium, mixed grass pollens, tree pollens (birch, beech, ragweed). Total serum IgE were extremely high (1065).

**Conclusion**: According to the literature 11 s globulins in buckwheat have the potential to induce IgE antibodies cross-reactive with 11S globulins in other, botanically unrelated foods and may induce anaphylactic reactions. In developing countries sophisticated diagnostic methods are not available, so we need to make a detail plan for preventing anaphylactic reaction in children with positive family history of anaphylactic reaction to food allergens without severe restrictive diet.

**Consent to publish**: Written informed consent was obtained from the patient and her father for publication of his abstract.

### PP187 A secondary school case study project using a whole school approach, to develop a practical toolkit for disseminating EAACI food allergy guidelines to all UK schools (Whole-School Allergy Awareness & Practical Action Management)

#### Jennette Higgs^1^, Amena Warner^2^, Carla Jones^2^, Audrey Dunn Galvin^3^

##### ^1^Food To Fit Ltd, London, United Kingdom; ^2^Allergy UK, Sidcup, United Kingdom; ^3^University College Cork, Cork, Ireland

**Correspondence**: Jennette Higgs - jennette@foodtofit.com

*Clinical and Translational Allergy* 2017, **7(Suppl 1)**:PP187

**Introduction**: Safeguarding pupils with allergy whilst at school is a growing need. EAACI guidelines [1] provide a sound basis for directing school allergy policy, but for effective risk minimisation, schools need them in a readily accessible format. This project bridges the gap between clinical guidance and practical policies secondary schools can adopt, using a stepwise, whole-school, risk assessment/management approach, with per-school unique outcomes.

**Fig. 9:**
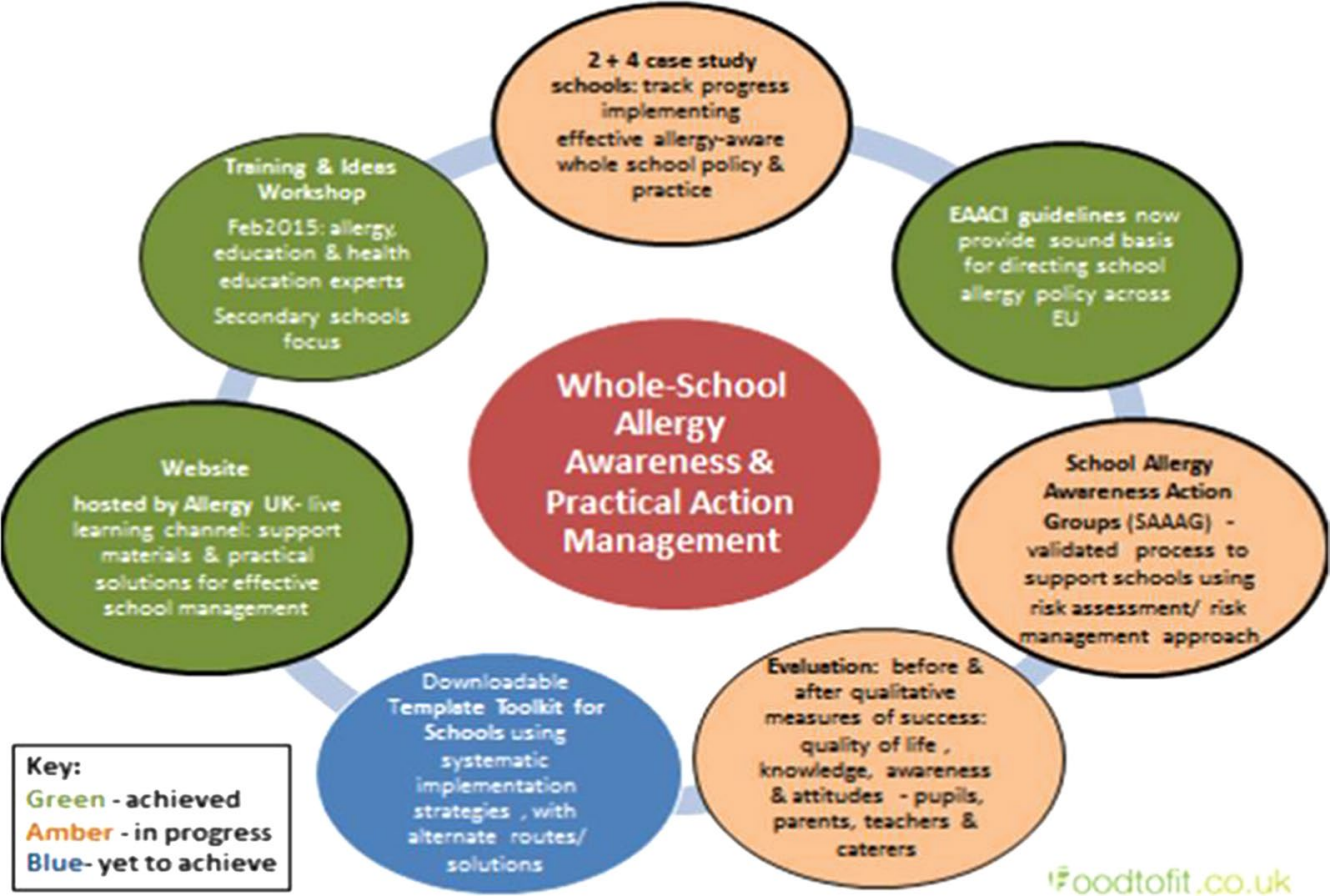
Concept

**Methods**: Using a multidisciplinary case study model to ultimately produce a free, accessible, online toolkit schools can adapt to their own whole-school allergy policies (Fig. 9). Central to success is a School-based Allergy Awareness Group (SAAG), to include School management, Teachers, Pupils, Parents, Caterers & School Nurse. 6 meetings over 1 yr will guide this working group to assess needs, implement, test and review policy with automatic reappraisal. Integral evaluation tools assess change over time: baseline telephone survey; self-efficacy & attitudes, validated questionnaires; SAAG in-house survey; online self-audit.

**Fig. 10:**
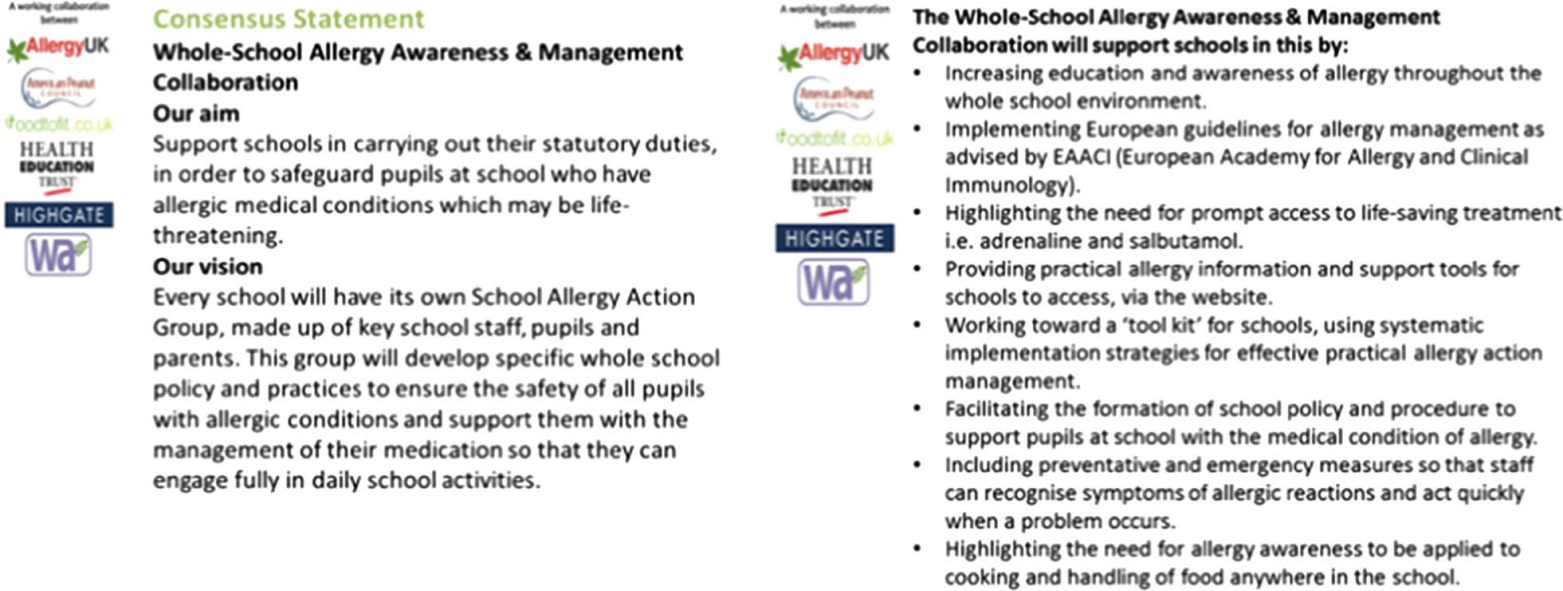
Consensus statement

**Results**: Consensus statement with expert collaboration to assure credibility for schools (Fig. 10).

Kick-off workshop provided content for website hosted by Allergy UK. 2 schools supported to pilot SAAG; each worked systematically to implement bespoke best practice policy. Case study evaluations analysis underway. Self-audit completed by 52 schools.

The SAAG process enables schools to adapt a structured and thorough template to develop a bespoke school allergy policy that best suits their needs. We plan to test different school systems via 3 new case studies in England and Ireland. In parallel will recruit schools for minimal facilitation SAAG test, to simulate independent use of toolkit. Qualitative and quantitative feedback from project evaluations will feed final review process prior to making toolkit available via Allergy UK website.

**Conclusion**: Transposing from clinical to practical solutions can be achieved with beneficial results. School feedback indicates this is a worthwhile time and resource investment for the school. Our work to date demonstrates that implementing allergy awareness best practice in UK secondary schools is a slow process. Raising the profile via a live website and targeted communications programme will help schools to safeguard pupils with allergy and ensure the whole school is appropriately allergy aware, so that inclusion becomes the norm for school culture.

**Consent to publish**: Subjects have given consent for presentation and publication of the case study.

**Reference**

1. Muraro A, Agache I, Clark A, Sheikh A, Roberts G, Akdis CA, Borrego LM, Higgs J, Hourihane JO’B, Jorgensen P, Mazon A, Parmigiani D, Said M, Schnadt S, van Os-Medendorp H, Vlieg-Boerstra BJ, Wickman M. EAACI Food Allergy and Anaphylaxis Guidelines: managing patients with food allergy in the community. Allergy 2014; 69: 1046–57.

